# Global, regional, and national quality of care of gallbladder and biliary tract cancer: a systematic analysis for the global burden of disease study 1990–2017

**DOI:** 10.1186/s12939-021-01596-y

**Published:** 2021-12-18

**Authors:** Javad Khanali, Mohammad-Reza Malekpour, Mohammadreza Azangou-Khyavy, Sahar Saeedi Moghaddam, Negar Rezaei, Ali-Asghar Kolahi, Mohsen Abbasi-Kangevari, Esmaeil Mohammadi, Nazila Rezaei, Moein Yoosefi, Mohammad Keykhaei, Yosef Farzi, Fateme Gorgani, Bagher Larijani, Farshad Farzadfar

**Affiliations:** 1 Non-Communicable Diseases Research Center, Endocrinology and Metabolism Population Sciences Institute, Tehran University of Medical Sciences, Tehran, Iran; 2 Social Determinants of Health Research Center, Shahid Beheshti University of Medical Sciences, Tehran, Iran; 3 Endocrinology and Metabolism Research Center, Endocrinology and Metabolism Clinical Sciences Institute, Tehran University of Medical Sciences, Tehran, Iran

**Keywords:** Global burden of disease, Quality of Care index, Gallbladder cancer, Biliary tract cancer, Principal component analysis, Health status indicators, Quality of healthcare

## Abstract

**Background:**

To improve health outcomes to their maximum level, defining indices to measure healthcare quality and accessibility is crucial. In this study, we implemented the novel Quality of Care Index (QCI) to estimate the quality and accessibility of care for patients with gallbladder and biliary tract cancer (GBBTC) in 195 countries, 21 Global Burden of Disease (GBD) regions, Socio-demographic Index (SDI) quintiles, and sex groups.

**Method:**

This cross-sectional study extracted estimates on GBBTC burden from the GBD 2017, which presents population-based estimates on GBBTC burden for higher than 15-year-old patients from 1990 to 2017. Four secondary indices indicating quality of care were chosen, comprising Mortality to incidence, Disability-Adjusted Life Year (DALY) to prevalence, prevalence to incidence, and years of life lost (YLL) to years lived with disability (YLD) ratios. Then, the whole dataset was analyzed using Principal Component Analysis to combine the four indices and create a single all-inclusive measure named QCI. The QCI was scaled to the 0–100 range, with 100 indicating the best quality of care among countries. Gender Disparity Ratio (GDR) was defined as the female to male QCI ratio to show gender inequity throughout the regions and countries.

**Results:**

Global QCI score for GBBTC was 33.5 in 2017, which has increased by 29% since 1990. There was a considerable gender disparity in favor of men (GDR = 0.74) in 2017, showing QCI has moved toward gender inequity since 1990 (GDR = 0.85). Quality of care followed a heterogeneous pattern among regions and countries and was positively correlated with the countries’ developmental status reflected in SDI (r = 0.7; CI 95%: 0.61–0.76; *P* value< 0.001). Accordingly, High-income North America (QCI = 72.4) had the highest QCI; whereas, Eastern Sub-Saharan Africa (QCI = 3) had the lowest QCI among regions. Patients aged 45 to 80 had lower QCI scores than younger and older adults. The highest QCI score was for the older than 95 age group (QCI = 54), and the lowest was for the 50–54 age group (QCI = 26.0).

**Conclusions:**

QCI improved considerably from 1990 to 2017; however, it showed heterogeneous distribution and inequity between sex and age groups. In each regional context, plans from countries with the highest QCI and best gender equity should be disseminated and implemented in order to decrease the overall burden of GBBTC.

**Supplementary Information:**

The online version contains supplementary material available at 10.1186/s12939-021-01596-y.

## Introduction

In order to improve health care delivery, it is essential to put deliberate emphasis on the quality of care, which means delivering timely, equitable, integrated, and efficient care that is effective, safe, and people-centered [1]. Fortunately, the urgency of improving care quality is beginning to be recognized by providers, health care systems, governments, and payers [2]. However, to achieve better quality, defining consistent metrics and indices for assessing healthcare quality and accessibility is a crucial yet challenging cornerstone that helps policymakers to evaluate health care systems’ progresses and shortcomings [2, 3].

World Health Organization defines the quality of care as “the degree to which health services for individuals and populations increase the likelihood of desired health outcomes” [1]. To conclude, an objective measurement of health outcomes can indicate the quality of care in each healthcare system. Before, few indices have been developed for assessing the quality and accessibility of care, such as the universal health coverage tracer index of 11 interventions [4], coverage index of three primary healthcare interventions [5], and the Healthcare Access and Quality Index (HAQI) [3, 6]. Another example is the attempt of WHO in the World Health Report 2000 to rank the health systems’ performance on “level of health,” represented by disability-adjusted life expectancy (DALE) and an “index of overall health system performance,” calculated as a weighted average of scores on five distinct dimensions [7]. All these studies use coverage of some interventions, deaths from some amenable causes of death or health performances and outcomes as proxies for the quality and accessibility of care of the whole health system. However, none of these indices carry out a disease-specific approach to assess the quality and accessibility of care. Therefore, alternative indices should be developed to determine the quality of care of patients with each kind of disease. In this regard, the group has developed the novel quality of care index (QCI) by combining four indices reflecting patients’ good health outcomes and applied the index to investigate the quality of care in various types of malignancies and ischemic heart disease [8–12].

For several reasons, Gallbladder and biliary tract cancer (GBBTC) patients are another group that the quality and accessibility of care they receive need to be assessed thoroughly and worldwide. As the first reason, although GBBTC is a rare cancer with an average incidence rate of 2.8 per 100,000 people in 2017 [13], it possesses a unique geographical and epidemiological distribution [14, 15]. Central and Northern Europe, India, Japan, and Chile have a much higher incidence of this cancer than the global average [16]. Besides, the incidence is higher in females and some ethnic groups [17]. As the second reason, this cancer has a poor prognosis and low 5-year survival rate and overall survival (e.g., a 5-year survival rate of 5% and overall survival of 6 months for gallbladder cancer) [17, 18]. Such poor prognosis is due to its asymptomatic nature in the early stages, making it difficult for healthcare systems to diagnose it in the early stages when the prognosis is much better [18, 19]. Third, the only potentially curative treatment in patients with the early-stage disease is complete surgical resection. However, only less than 25% worldwide have access to safe, timely, and high-quality cancer surgery [19, 20]. Finally, the largest burden of GBBTC is in the “less-developed” regions of the world, where early diagnosis and curative treatment are much harder to be achieved [18]. All these reasons theoretically could cause global inequities in the quality of care for GBBTC patients.

Considering the poor prognosis, surgical needs of patients, the unique geographical, sex, and ethnic distribution of GBBTC, and its high incidence in less-developed regions of the world, there should be a vast heterogeneity in the quality of care for the disease. This study has used the QCI to compare different healthcare systems by their care quality and accessibility for GBBTC patients and show global inequities in this regard. Here, comprehensive and comparative estimates of GBBTC burden provided by the Global Burden of Disease (GBD) study were represented and employed to calculate QCI in 195 countries, 21 GBD regions, and Socio-demographic Index (SDI) quintiles from 1990 to 2017.

## Materials & methods

### Calculating quality of Care index (QCI)

To create a proxy for the quality and accessibility of care, we have implemented six primary indices, including incidence, prevalence, mortality, years of life lost (YLL), years lived with disability (YLD), and disability-adjusted life years (DALYs) to form four secondary indices. These four secondary indices that each could indicate the quality and accessibility of care comprises:

$${\displaystyle \begin{array}{cc}\mathrm{Mortality}\kern0.5em \mathrm{to}\kern0.5em \mathrm{incidence}\ \mathrm{ratio}=\frac{\# Mortality}{\# Incidence}& \mathrm{DALY}\kern0.5em \mathrm{to}\kern0.5em \mathrm{prevalence}\kern0.5em \mathrm{ratio}\kern0.5em =\kern0.5em \frac{\# DALY}{\#\mathit{\Pr} evalence}\\ {}\mathrm{Prevalence}\kern0.5em \mathrm{to}\kern0.5em \mathrm{incidence}\ \mathrm{ratio}\kern0.5em =\kern0.5em \frac{\#\mathit{\Pr} evalence}{\#Incidence}& \mathrm{YLL}\kern0.5em \mathrm{to}\kern0.5em \mathrm{YLD}\kern0.5em \mathrm{ratio}\kern0.5em =\kern0.5em \frac{\# YLL}{\# YLD}\end{array}}$$

Then, the Principal Component Analysis (PCA) was performed, to combine four indices to form a single all-inclusive index named QCI [21]. PCA is a mathematical approach that reduces large data sets’ dimensionality by transforming plentiful variables into a smaller number while retaining most of the information in the larger data [22]. Therefore, the QCI could be implemented as a single index for assessing the quality of care that retains most of the information of the four mentioned secondary indices. The detailed protocol of computing QCI for various types of diseases can be found elsewhere [23]. The Additional File 1 presents details of the mathematical calculation of QCI for GBBTC (Additional File 1). In this study, all primary indices were used as age-standardized measures, and all QCI scores (except those that specify an age group) are age-standardized QCI scores.

Here, QCI was determined and studied in different countries and regions in 1990–2017 across ages, sexes, and the last estimates of Socio-demographic Index (SDI) quintiles [24, 25]. QCI scores were scaled to the 0–100 range, with 100 indicating the best quality of care in the samples. Besides, we categorized countries into five levels based on the QCI quintiles of 1990 data, where Level 1 indicates countries with the highest quality of care. To represent the change in QCI in the 1990–2017 period, the percent change in QCI score was implemented and calculated by the following formula.

$$\kern4.5em \mathrm{QCI}\ \mathrm{percent}\ \mathrm{change}=\frac{\left(2017\ QCI-1990\ QCI\right)}{1990\ QCI}\times 100$$

### Gender and age disparity

Gender Disparity Ratio (GDR) was defined as a sub-index for QCI as follows:

$$\mathrm{GDR}=\frac{\ QCI\ for\ females}{\ QCI\ for\ males}$$

The QCI of males and females was pooled and calculated separately. This index could speed up comparing QCI for males and females and could show gender equity and inequities throughout the countries studied. GDR values closer to one indicate better gender equity; whereas, far higher values than one show inequity in favor of women, and far lower values than one show vice versa.

For assessing age disparity, QCI for each age group was calculated separately in global, regional, and SDI scales. Since the GBBTC data has been available for higher than 15-year-old of age, all data presented and analyzed in the study is limited to this age group. Besides, the age of patients was classified into five-year intervals starting from the 15–19 group ending to the + 95 group.

### Data source

The gallbladder and biliary tract cancer data were derived from the Global Burden of Diseases, Injuries, and Risk Factors Study 2017 (GBD 2017). This study provides comprehensive, comparable estimates of incidence, death, prevalence, YLL, YLD, DALY for 354 diseases and injuries and 282 causes of death through 195 countries and territories, seven super-regions, and 21 regions from 1990 to 2017. Besides, the latest SDI estimates, provided by GBD, were used as a summary measure that identifies countries’ development status, expressed on a scale of 0 to 1 [24]. The codes included in the study comprise the GBD code of B.1.8 and the International Statistical Classification of Diseases and Related Health Problems 10th Revision (ICD-10) code of C23-C24.9, D13.5. The data could be accessed on the Institute for Health Metrics and Evaluation (IHME) website [26] and is visualized in the GBD compare online tool [27]. Additional File 2 represents all primary indices used in the study through countries, regions, and SDI quintiles (Additional File 2).

### Statistical analysis

To validate the QCI proxy, we examined the correlation between the QCI and the Healthcare Access and Quality (HAQ) index, which has previously been introduced by the Institute of Health Metrics and Evaluation (IHME) as a proxy of care quality and access [3, 6]. This assessment was performed by applying a mixed-effect regression model of QCI as a dependent variable, mean outpatient visits and inpatient admissions per capita [28], and GBBTC prevalence as independent variables while considering countries as random effects. The Pearson correlation coefficient between the HAQ Index and the predicted values was 0.59. Previous studies we performed on QCI show even a higher correlation, indicating that QCI and HAQI are essentially assessing similar components when evaluating health quality [8–11].

Primary measures were reported as point estimations and the 95% uncertainty intervals (95% UIs). The standardized rates were calculated using the direct method of standardization to the world population. The *P* < 0.05 was considered as the level of significance. As mentioned above, The QCI of different regions, countries, and age and sex groups were calculated. The whole GBD dataset was analyzed, and there were no missing data. The correlation between SDI and QCI was examined using Pearson’s correlation analysis. All statistical analyses were performed using R statistical packages v3.4.3 (http://www.r-project.org, RRID: SCR_001905) [29]. Data visualizations were conducted using Python programming language (version 3.6. Available at www.python.org) via Altair version 4.1.

## Results

### Global and National Incidence, mortality and DALY of gallbladder and biliary tract Cancer in 1990–2017

Although the global incidence of GBBTC have increased by 125% in 1990–2017 (from 199,943 to 210,878 people), the global age-standardized incidence rate of GBBTC have decreased from 3.1 (95% CI, 3 to 3.5) to 2.7 (95% CI, 2.4 to 2.9) per 100,000 people (Additional File 2). However, there was heterogeneity in the 2017 incidence rate and its changes in 1990–2017 among countries. The 2017 age-standardized incidence rate varied from 10.8 per 100,000 people in Chile to 0.5 per 100,000 people in Iraq. Chile, Japan, and South Korea were the three countries with a higher than 8 per 100,000 incidence rate. Concerning the rise in age-standardized incidence rate, Armenia, Georgia, and India were the countries that showed the highest increase, with 77, 75, and 33% increase, respectively.

A 22% decrease was also observed in both age-standardized mortality rate and age-standardized DALYs per 100,000 people in 1990–2017 (from 2.8 to 2.2 deaths per 100,000 people, and from 55.2 to 43.2 DALYs per 100,000 people, respectively). Chile, Uruguay, and South Korea were the three countries that had the highest age-standardized death rate in 2017, with 10.4, 7.7, and 5.9 deaths per 100,000 people, respectively. The first two mentioned countries, Chile and Uruguay, and Argentina, also had the highest age-standardized DALYs in 2017. Armenia, Georgia, and India were also the countries that showed the highest increase in age-standardized death rates and age-standardized DALYs in 1990–2017. The increase in the age-standardized death rate was 81% for Armenia, 76% for Georgia, and 34% for India. These countries also showed a 71, 80, and 32% increase in age-standardized DALYs, respectively (Additional File 2).

### Global, regional and National Pattern in quality of Care index (QCI) and Progress on it in 1990–2017

QCI scores for GBBTC were 29.1 for women, 39.3 for men, and 33.5 for both sexes in 2017. QCI also followed a heterogeneous geographical pattern among 21 GBD regions, and 195 countries studied. High-income North America (QCI = 72.4), High-income Asia Pacific (QCI = 56.4), and Western Europe (QCI = 52.2) were the three regions with the highest QCI score in 2017. On the opposite side of the spectrum, Eastern (QCI = 3.0), Central (QCI = 3.1), and Western (QCI = 4.4) Sub-Saharan Africa regions had the lowest scores. Among countries, the USA (QCI = 77.3), Norway (QCI = 65.3), Spain (QCI = 64.6), Germany (QCI = 62.1), and Japan (QCI = 59.2) had the highest; and Central African Republic (QCI = 1.8), Eritrea (QCI = 2.0), Pakistan (QCI = 2.0), Somalia (QCI = 2.1), and Madagascar (QCI = 2.3) had the lowest QCI scores in 2017 (Additional Files 3, 4).

The global QCI score for GBBTC patients has increased by 29% from 1990 to 2017 (from 25.9 to 33.5). This increase also occurred for both sexes, with a 17% rise for women and a 34% rise for men (Additional File 5). However, such a rise did not happen in all GBD regions. The Caribbean, Central Asia, and Southern Sub-Saharan Africa were the three GBD regions with a negligible change in QCI score (less than 1% change). Conversely, East Asia, Southeast Asia, Southern Latin America, and Eastern Europe were the regions with a higher than 100% increase in QCI of GBBTC. Among countries, South Korea (495% increase), China (380% increase), and Maldives (366% increase) had the highest increases, whereas Sweden (68% decrease), Georgia (30% decrease), and Zimbabwe (28% decrease) had the highest decrease in QCI score for GBBTC.

**Fig. 1:**
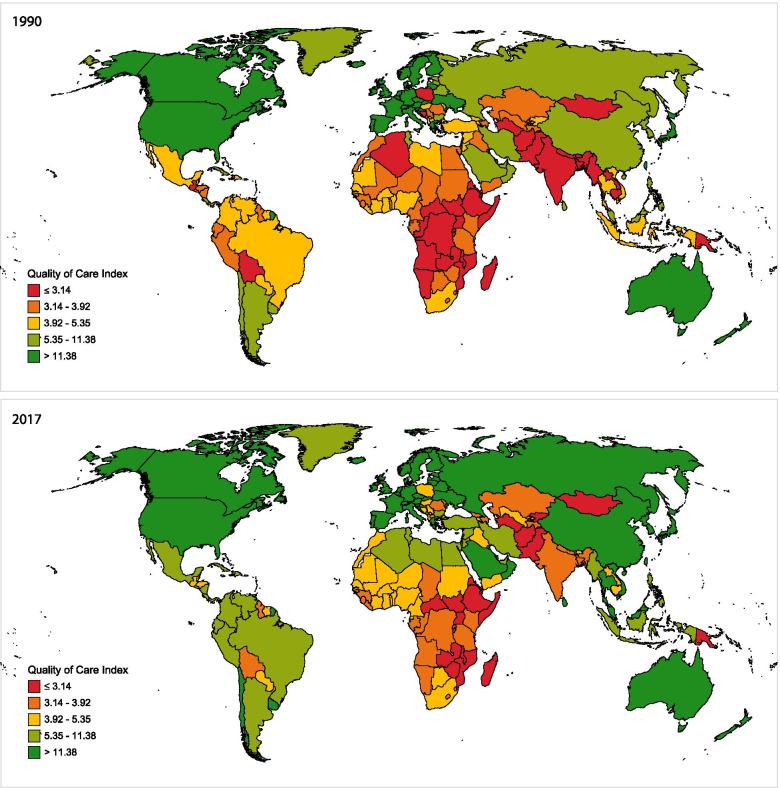
Map of Age-standardized Quality of Care Index for gallbladder and biliary tract cancer by quintiles of 1990 in **A**) 1990 and **B**) 2017

Most of the 39 countries in the highest quintile of the QCI score for GBBTC in 1990 were clustered in Western Europe, High-income North America, and Australasia regions (Fig. 1a). These countries (Level 1 countries) had a QCI score of higher than 11.38. From 1990 to 2017, not only none of these 39 countries dropped down from this score, but also other 33 countries passed this threshold score, mostly from Southeast Asia, Eastern Europe, East Asia, North Africa and Middle East, and Southern Latin America regions. Of the 39 level-5 countries (QCI score < 3.14) in 1990, only 19 countries remained at such low scores in 2017; however, Kyrgyzstan and Zimbabwe dropped below the QCI score of 3.14 (dropped to level 5) from higher scores (Fig. 1b).

### The relationship between QCI and SDI of countries

**Fig. 2:**
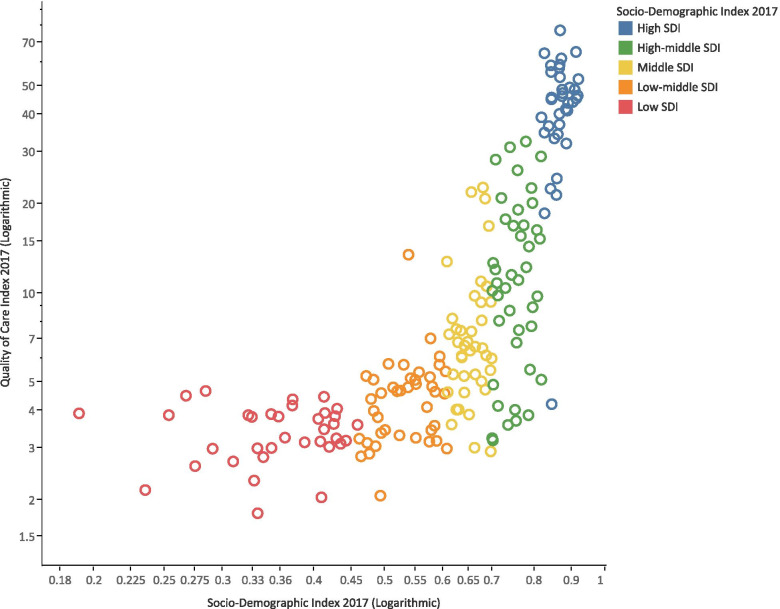
Age-standardized Quality of Care Index for gallbladder and biliary tract cancer by SDI score of countries in 2017

QCI values were generally higher in countries with higher SDI (Fig. 2), and QCI showed a positive correlation with SDI (r = 0.7; CI 95%: 0.61–0.76; *P* value< 0.001). The QCI score was 55.4 for high, 23.9 for high-middle, 12.0 for middle, 3.7 for low-middle, and 3.1 for low SDI quintiles (Additional File 3). Notably, the spread of QCI scores was wider in higher SDI quintiles. The interquartile range (IQR) of QCI scores was 16.17 for the countries in the high SDI quintile; however, it was 0.89 for the countries in the low SDI quintile.

**Fig. 3:**
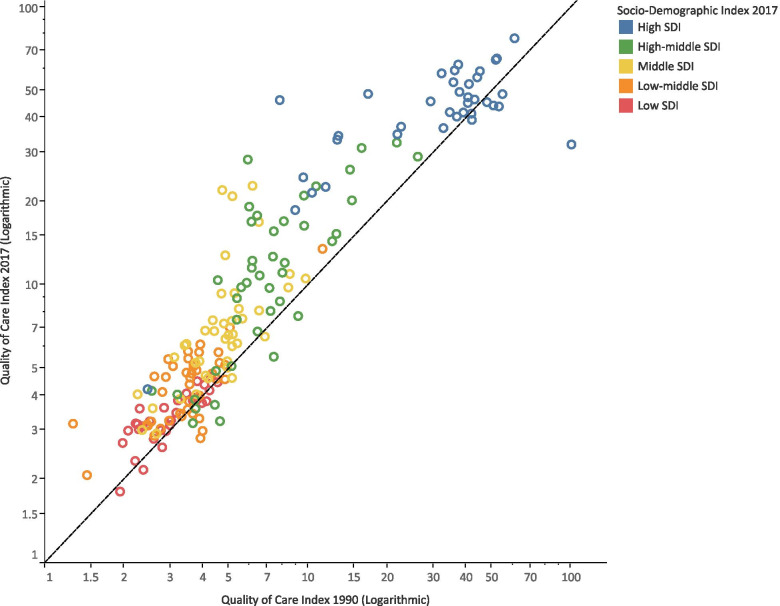
Comparison of 1990 and 2017 Age-standardized Quality of Care Index estimates among countries in different SDI levels

The rise in quality of care between 1990 and 2017 occurred in most of the countries of all SDI quintiles (Fig. 3). This rise was 34% for high, 225% for high-middle, 159% for middle, 16% for low-middle, and 14% for low SDI quintiles. Among the first quintile of countries with the highest rises in QCI, only Bolivia, Eritrea, Myanmar, and Guatemala were from low and low-middle SDI quintiles.

### QCI score in different age groups

**Fig. 4:**
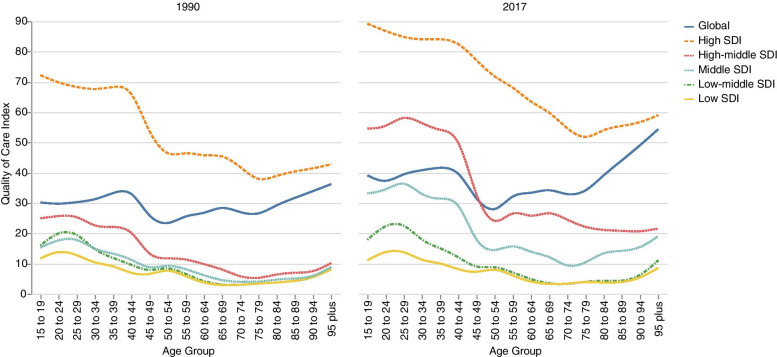
Age pattern of Quality of Care Index for gallbladder and biliary tract cancer among different SDI levels in **A**) 1990 and **B**) 2017

Global QCI of GBBTC in 2017 was lower in patients aged 45 to 80 (Fig. 4, Additional File 6). The highest QCI score was for the older than 95 age group (QCI = 54), and the lowest was for the 50–54 age group (QCI = 26.0). The higher QCI scores in higher SDI quintiles were also observed in all age groups. The only exception was the low-middle SDI quintile in 70–74 and 75–79 age groups, which had lower QCI scores than the low SDI quintile.

Global QCI scores in all age groups were higher in 2017 than in 1990. The top three highest increases were occurred in older than 95, 90–94, and 85–89 age groups, with 49, 44, and 38% increase, respectively. Notably, this increase was much higher than the global average increase of 29% for the all-ages group.

### Gender disparity ratio (GDR)

There was considerable gender disparity in quality of care for GBBTC in 2017, and Only 33 countries had a GDR value between 0.8 and 1.2 in this year (Additional File 4). Besides, most countries showed gender inequity in favor of men (only 23 countries had GDR > 1), and the global GDR was 0.74. The only regions with worse quality of care for men than women were Eastern and Central Europe regions (GDR was 1.34 and 1.14, respectively). Conversely, South Asia (GDR = 0.34), Andean Latin America (GDR = 0.46), and Eastern Sub-Saharan Africa (GDR = 0.51) had the worst quality of care scores for women compared with men. The countries with the highest gender disparity in favor of women were Slovenia (GDR = 6.07), Croatia (GDR = 1.89), Lithuania (GDR = 1.86), Bulgaria (GDR = 1.71), and Estonia (GDR = 1.60), which all are countries from Eastern and Central Europe regions. The highest 13 countries in GDR, of which 11 were Eastern and Central European countries, had a GDR more than one and a half interquartile range over the third quartile (GDR > 1.11). On the opposite side of the spectrum, countries with the highest gender disparity in favor of men were Pakistan (GDR = 0.22), India (GDR = 0.36), Azerbaijan (GDR = 0.38), Afghanistan (GDR = 0.41), and Bolivia (GDR = 0.41). In the 1990–1017 period, 114 out of 195 countries progressed toward equity, however global GDR moved toward inequity (from 0.85 in 1990 to 0.74 in 2017).

**Fig. 5:**
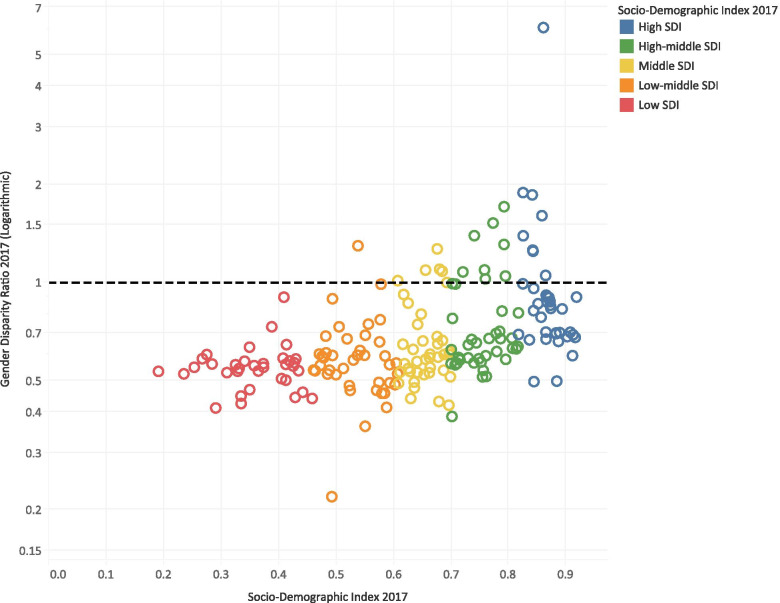
Gender Disparity Ratio for Quality of Care Index of the gallbladder and biliary tract cancer by SDI score of countries in 2017

All SDI quintiles showed better quality of care for men than women; nevertheless, higher SDI quintiles showed much fewer inequities. GDR was 0.88 for high, 0.86 for high-middle, 0.75 for middle, 0.42 for low-middle, and 0.39 for low SDI quintiles (Fig. 5). Only high-middle and middle SDI quintiles progressed toward gender equity in the 1990–2017 period.

## Discussion

Inferring the GBD 2017, we have implemented a novel index –QCI- to estimate the quality and accessibility of care for patients with GBBTC worldwide. The study revealed considerable heterogeneities regarding 2017 QCI scores and how quickly regions and countries have improved since 1990. However, considering all heterogeneities among countries, the global QCI score increased from 25.9 to 33.5 in 1990–2017. Besides, the QCI showed a positive correlation with SDI, along with showing vast age and gender disparities.

There were considerable heterogeneities among regions and countries regarding 2017 QCI scores and how greatly their QCI has increased since 1990. Referring to our results, the variable that was significantly correlated with QCI and could create such heterogeneity was countries’ developmental status, which is reflected in SDI [24]. Accordingly, Regions like High-income North America, High-income Asia Pacific, and Western Europe, and countries like the USA, Norway, and Spain, which have sat ahead along the development spectrum, had higher QCI scores in 2017 [25]. Besides, the high percent increase of QCI in Korea, China, and the Maldives was a rationale result since these countries are known for their striking development in the past few decades [25]. This positive correlation between QCI and SDI was expected because early diagnosis, high-quality cancer surgery, and public care accessibility, which are more available in countries with higher SDI, could significantly improve QCI scores in the case of GBBTC [17, 18, 30]. In concrete, it is proved that GBBTC treatment in a multidisciplinary setting at experienced centers increases the patients’ overall survival [19]. However, the “less developed” regions of the world that have the most burden of GBBTC suffer from low awareness of cancer, delay in seeking treatment in the general population, an inadequate number of health care workers, scarce resources, and lack of high-quality treatments [18, 31].

Nevertheless, the study also showed a big difference in QCI scores among countries at a similar SDI quintile, which carries a hoping and a warning message. The hoping one is that the countries that lagged behind could improve their QCI score as their frontier counterparts with a similar SDI did. However, the warning message is that quality of care is not an inevitable effect of countries’ development, as we see Poland as a high SDI country having QCI scores lower than many of the high-middle and middle SDI countries [3]. Budget constraints within the health sector and persistent subnational inequalities in access to care due to high out-of-pocket payments are some reasons for such low quality and accessibility of care in Poland [32]. However, further detailed and case-by-case studies may need to understand why some countries are lagging behind in the quality of care despite their high developmental status and how they can pursue improving quality and accessibility of care.

The 29% rise in QCI of GBBTC in 1990–2017, plus projected world’s development and SDI improvement, keep hopes alive that the GBBTC mortality will continue to be declining. This decline would be particularly important to achieve one of the health-related sustainable development goals, reducing premature mortality from non-communicable diseases by one-third by 2030 [33]. However, the world should be aware of the consequences of population aging and growth, which resulted in a 125% increase in GBBTC incidence in 1990–2017 [13]. Considering projected further growth and aging of the world population [34], further notice to quality of care for the disease would be mandatory. Equitable and widespread providing of facilities such as sonograms and Computed Tomography scanners; along with human resources, drugs, and devices for high-quality cancer surgery, chemotherapy and radiotherapy would decrease the mortality rate of the disease [18]. Besides, fighting against obesity, non-healthy eating patterns, physical inactivity, and excessive alcohol drinking are amongst the established preventive measures. However, the major burden of GBBTC is in the less developed regions of the world where have a minimum health expenditure and react to problems rather than respond to them. This epidemiological distribution may distract attention to the prevention and quality of care in the countries [18].

Besides having plans that benefit all parts of the health system by improving healthcare quality and moving toward universal health coverage, countries could launch specific programs to improve care quality for GBBTC. Having such programs are essential for countries with high incidence and mortality from the disease. In this regard, some countries should be introduced as role models for preventing premature deaths due to GBBTC. Chile, a high-middle SDI country with one of the highest incidence rates for GBBTC worldwide (14.02 new cases per 100,000 in 2017), could be assumed as an example [13]. The high incidence in Chile is believed to be majorly due to the high prevalence of gallstone disease as the strongest risk factor of GBBTC [35, 36]. The Chilean government has created a specific program to facilitate the early diagnosis of symptomatic gallstones and provide rapid treatment for patients at a higher risk of developing gallbladder cancer [18, 36]. The 178% increase in QCI score for Chile in the 1990–2017 period could be partly due to this program, in which one of its main goals is decreasing the number of patients with advanced metastatic GBBTC [37].

The QCI score of GBBTC also showed considerable age and gender disparity. The higher QCI for adults older than 80 in comparison with adults aged 45 to 80 could be due to lower years which is lost by death in this age group. The lower loss of years decreases YLLs and DALYs in proportion to the high age-specific incidence and prevalence of GBBTC in the age group [13, 26]. In explaining gender disparity showed by the study, GDR and SDI did not show considerable correlation (r = 0.32); however, countries with higher QCI for women than men were majorly from high and high-middle SDI quintiles. High GDR in favor of men in low SDI countries could be partly justified considering delayed healthcare seeking in women due to cultural, socio-economic, and educational barriers [38, 39]. This problem is well-documented in Pakistan as the country with the highest GDR in favor of men, referring to the study results [39, 40]. Other potential reasons for higher QCI in men than women remained unexplained and should be further studied in the future.

Although most of the study results were anticipated, an unexpected result was obtained in the 2017 QCI of GBBTC for Sweden. The QCI score of this country was 100 in 1990 but declined to 32 in 2017. Such a decrease in QCI happened despite a 46% decline in incidence and a 32% decline in mortality of GBBTC in Sweden during 1990–2017 [26]. The reason was the high mortality to incidence proportion (higher than 1) in 2017 GBD data, which had been repeated in 2016 and 2015. However, the cause of such high mortality to incidence proportion for Sweden remained unknown to us.

Another notable result was the USA which had the highest QCI in 2017. Considering disparities in insurance coverage, access to care, and health costs in the US, the result was not expected for us [41–44]. However, the top care quality in the US for individuals who have good access and insurance choice may mask the shortcomings and disparities. A closer assessment of this country in the subnational and ethnic levels may be necessary to show the strengths and shortcomings that led to the result.

The study was also subject to some limitations. First, the study’s data was derived from GBD 2017; therefore, any GBD 2017 study limitations are also applicable here [45, 46]. Second, QCI does not capture all aspects of healthcare quality. For instance, QCI does not represent estimates for subnational level quality and inequalities; however, the inequality within countries could spark constructive debates on resource allocation by health systems. Therefore, providing more detailed data that include subnational levels would be a focus of efforts in future works. As the third limitation, a lead-time bias occurs when countries with different screening schedules and protocols for gallbladder cancer are compared, and a country detects the disease at an earlier time point and has a falsely increased overall survival due to the bias. In the QCI methodology, YLD is affected by the bias, and DALY is minimally affected due to the small share of YLD in DALY in GBBTC. The bias should be considered and corrected in future studies [47]. As the fourth limitation, there is a lack of data on the intervention coverage in GBBTC patients in many countries of the world. Therefore, it was not possible to incorporate intervention coverage into the QCI estimations on quality of care, and incorporating the variables would be the subject of future researches. Despite these limitations, QCI presents a holistic view regarding the quality of care for many types of diseases based on geographical distribution, sex, and age groups. In terms of validity, the QCI showed an acceptable correlation with the Healthcare Access and Quality (HAQ) index previously implemented to assess the quality of care for some death causes [3, 6]. Besides, QCI might have some unique features that were not offered by the HAQ index. First, the HAQI uses the mortality from 32 amenable causes of death as a proxy for the healthcare quality of the whole health system. In contrast, QCI assesses the quality of care for each disease apart, which makes a judgment about the quality of care more case-by-case and straightforward. This feature enables to determine different countries’ performance and strategies encountering each health problem. However, the disease-specific approach in QCI and its methodology do not allow to compare the quality of care for different diseases within countries. As the second unique feature and unlike HAQI, QCI captured estimates for quality of care in different sex groups and GBD regions, which enabled better interpreting discrepancies in care quality.

## Conclusion

Here, an objective assessment of care quality for GBBTC patients has been provided, which could be used to compare healthcare systems in providing facilities for GBBTC patients in terms of early diagnosis, patient care and treatment, and care accessibility. The considerable difference in QCI and GDR among countries, even countries at a similar SDI quintile, shows that progress remains to be made for QCI and filling inequity gaps. Even in countries having almost similar income levels, the disadvantage of one race, ethnicity, gender or gender identity, class, and sexual orientation, and the unequal allocation of power and resources, including goods, services, and societal attention can hinder progress toward quality of care. To achieve quality, health systems should invest in new skills, facilities, and equipment, as well as maintain their existing infrastructures. Moreover, the balanced investment must be maintained over time and between different geographical areas.

## Supplementary Information

**Additional file 1.** Details of the mathematical calculation of QCI for GBBTC.

**Additional file 2.** Age-standardized Incidence, Death, years of life lost (YLL), years lived with disability (YLD), and disability-adjusted life years (DALYs) for gallbladder and biliary tract cancer along 21 GBD regions, 195 countries, and SDI quintiles.

**Additional file 3.** Age-standardized Quality of Care Index in 21 GBD regions, 195 countries, and SDI quintiles in 1990 and 2017.

**Additional file 4.** Sex-specific age-standardized Quality of Care Index, and Gender Disparity Ratio in 21 GBD regions, 195 countries, and SDI quintiles in 1990 and 2017.

**Additional file 5.** The global trend of QCI in female, male, and both from 1990 to 2017.

**Additional file 6.** Age-specific Quality of Care Index in Global and SDI quintiles’ level in 1990 and 2017.

## Data Availability

The data that support the findings of this study are available from Non-Communicable Diseases Research Center, Endocrinology and Metabolism Population Sciences Institute, Tehran University of Medical Sciences, Tehran, Iran, but restrictions apply to the availability of these data, which were used under license for the current study, and so are not publicly available. However, data are available from the authors upon reasonable request and with permission of the Institute of Health Metrics and Evaluation.

## Abbreviations

DALYs: Disability-adjusted life years
GDR: Gender disparity ratio
GBD: Global Burden of Disease
HAQ: Healthcare Access and Quality
ICD-10: International Statistical Classification of Diseases and Related Health Problems 10th Revision
IHME: Institute for Health Metrics and Evaluation
GBBTC: Gallbladder and biliary tract cancer
PCA: Principal Component Analysis
QCI: Quality of care index
SDI: Socio-Demographic Index
YLDs: Years lived with disability
YLLs: Years of life lost
