# Supplementary material for: Global, regional, and national quality of care of gallbladder and biliary tract cancer: a systematic analysis for the global burden of disease study 1990–2017
Source: Int J Equity Health. 2021 Dec 18;20:259. doi: 10.1186/s12939-021-01596-y (PMC8684179; doi:10.1186/s12939-021-01596-y)
Supplement: Supplementary file 1 — Additional file 1. Details of the mathematical calculation of QCI for GBBTC. [file 12939_2021_1596_MOESM1_ESM.docx]

**Quality of Care Index (QCI) construction**

PCA is a mathematical approach that uses an n-dimensional transformation of data points to achieve an eigenvector that resembles and covers the largest data span on its axis. In our case, we had four secondary measures comprising Mortality to incidence, Disability-Adjusted Life Year (DALY) to prevalence, prevalence to incidence, and years of life lost (YLL) to years lived with disability (YLD) ratios; that we wanted to composite into one characteristic. Therefore, it is a four-dimension contemplate with +200 location data x in 27 years (from 1990 to 2017). After running the PCA code on the GBD 2017 dataset, we investigate the amount of variance among data points that can be explained by different eigenvectors (components). Conventionally, the one vector with the most discrimination ability (explaining the largest amount of variability and variance of data points) is defined as the first component of PCA and considered a composite characteristic. This approach was then reiterated for each age-group x sex-groups. The PCA scores (=loading factors) were implemented and rescaled into 0-100 and named QCI. We used a post-hoc investigation on QCI calculations, and it has grabbed 97.1% of variation [in both-sex age-standardized population calculation] of the four input parameters. On Average, PCA has retrieved 96.3% of the variation in the calculation for different subpopulations (consisting of a different combination of sex and age groups) ranging between 88.26% and 99.17%. The below figures show the properties of PCA calculation. Also, checked for correlation of QCI and each of the four entry variables, all |correlation coefficients| were greater than 0.97. A large amount of grabbed variability and association with either input variable made us confident that QCI can be a good quality indicator in GBBTC cancers.


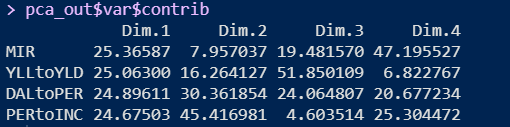


Figure 1: Percent of contribution of each of four secondary variables (as entries) with different components [=dimensions] of PCA on age-standardized both-sex subpopulation of GBBTC cancers from 1990 to 2017.


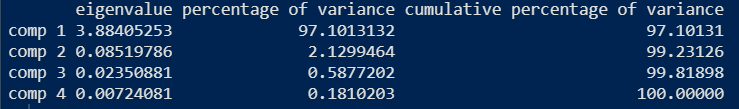


Figure 2: The eigenvalue of the first component was close to 4 (as the largest possibility in a 4-dimension PCA) and has grabbed 97.1% of variability and information of total data points in age-standardized both-sex population. Also, it revealed a mean variance of 96.3% on average in different subpopulations.


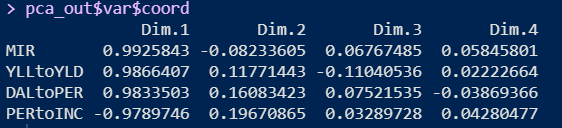


Figure 3: The slopes of each of four entry variables in each of dimensions. Notably, all |correlation coefficients| were greater than 0.97 in the dimension #1 (first component).
