# Supplementary material for: Global, regional, and national quality of care of gallbladder and biliary tract cancer: a systematic analysis for the global burden of disease study 1990–2017
Source: Int J Equity Health. 2021 Dec 18;20:259. doi: 10.1186/s12939-021-01596-y (PMC8684179; doi:10.1186/s12939-021-01596-y)
Supplement: Supplementary file 2 — Additional file 2. Age-standardized Incidence, Death, years of life lost (YLL), years lived with disability (YLD), and disability-adjusted life years (DALYs) for gallbladder and biliary tract cancer along 21 GBD regions, 195 countries, and SDI quintiles. [file 12939_2021_1596_MOESM2_ESM.html]

|  | | 1990 | | | | | | | | | | 2017 | | | | | | | | | |
| --- | --- | --- | --- | --- | --- | --- | --- | --- | --- | --- | --- | --- | --- | --- | --- | --- | --- | --- | --- | --- | --- |
|  | | Incidence | | Deaths | | YLLs (Years of Life Lost) | | YLDs (Years Lived with Disability) | | DALYs (Disability-Adjusted Life Years) | | Incidence | | Deaths | | YLLs (Years of Life Lost) | | YLDs (Years Lived with Disability) | | DALYs (Disability-Adjusted Life Years) | |
| Location | Sex | Number | Rate | Number | Rate | Number | Rate | Number | Rate | Number | Rate | Number | Rate | Number | Rate | Number | Rate | Number | Rate | Number | Rate |
| Afghanistan | Both | 155 (86 to 246) | 2.2 (1.3 to 3.4) | 159 (91 to 248) | 2.4 (1.4 to 3.6) | 3832 (2031 to 6362) | 51.7 (28.1 to 84.6) | 34 (17 to 57) | 0.5 (0.3 to 0.8) | 3867 (2048 to 6419) | 52.2 (28.4 to 85.2) | 216 (147 to 301) | 2.2 (1.5 to 3) | 216 (149 to 299) | 2.3 (1.6 to 3.2) | 5775 (3805 to 8285) | 49.6 (33.8 to 68.9) | 48 (29 to 73) | 0.5 (0.3 to 0.7) | 5824 (3836 to 8347) | 50.1 (34.1 to 69.6) |
|  | Female | 86 (44 to 137) | 2.9 (1.5 to 4.2) | 88 (46 to 136) | 3.1 (1.7 to 4.3) | 2168 (1086 to 3694) | 65.2 (33.6 to 106.4) | 19 (9 to 34) | 0.6 (0.3 to 1) | 2187 (1095 to 3726) | 65.8 (33.9 to 107.6) | 133 (83 to 194) | 2.7 (1.7 to 4) | 134 (83 to 196) | 2.9 (1.9 to 4.4) | 3567 (2137 to 5537) | 60.7 (37.8 to 88.9) | 30 (17 to 48) | 0.6 (0.3 to 0.9) | 3597 (2155 to 5579) | 61.3 (38.2 to 89.7) |
|  | Male | 69 (28 to 126) | 1.8 (0.8 to 3.2) | 70 (30 to 129) | 1.9 (0.9 to 3.4) | 1664 (625 to 3183) | 40.8 (15.9 to 77.7) | 15 (6 to 30) | 0.4 (0.2 to 0.8) | 1679 (632 to 3210) | 41.2 (16.1 to 78.4) | 83 (47 to 133) | 1.6 (1 to 2.6) | 82 (49 to 131) | 1.7 (1.1 to 2.8) | 2208 (1182 to 3733) | 38.3 (22 to 62.8) | 19 (9 to 34) | 0.4 (0.2 to 0.6) | 2227 (1192 to 3762) | 38.7 (22.2 to 63.3) |
| Albania | Both | 22 (19 to 30) | 1 (0.9 to 1.4) | 23 (20 to 32) | 1.1 (0.9 to 1.5) | 533 (470 to 676) | 23.3 (20.5 to 30.6) | 5 (3 to 7) | 0.2 (0.2 to 0.3) | 538 (475 to 684) | 23.5 (20.7 to 31) | 45 (35 to 63) | 1.1 (0.8 to 1.5) | 49 (38 to 68) | 1.2 (0.9 to 1.6) | 977 (759 to 1338) | 23.6 (18.3 to 31.7) | 10 (6 to 15) | 0.2 (0.2 to 0.4) | 987 (767 to 1352) | 23.8 (18.4 to 32) |
|  | Female | 11 (9 to 16) | 0.9 (0.8 to 1.4) | 11 (10 to 17) | 1 (0.8 to 1.5) | 251 (214 to 356) | 21.6 (18.3 to 31.3) | 2 (2 to 4) | 0.2 (0.1 to 0.3) | 253 (216 to 360) | 21.8 (18.5 to 31.6) | 28 (20 to 38) | 1.3 (0.9 to 1.7) | 30 (21 to 41) | 1.3 (0.9 to 1.8) | 596 (415 to 814) | 27.7 (19.2 to 38) | 6 (4 to 9) | 0.3 (0.2 to 0.4) | 602 (418 to 823) | 28 (19.4 to 38.4) |
|  | Male | 11 (9 to 16) | 1.1 (0.9 to 1.7) | 11 (9 to 16) | 1.2 (1 to 1.8) | 283 (221 to 370) | 25.4 (20.6 to 35) | 3 (2 to 4) | 0.3 (0.2 to 0.4) | 285 (223 to 373) | 25.7 (20.8 to 35.4) | 17 (12 to 32) | 0.9 (0.6 to 1.6) | 19 (13 to 35) | 1 (0.6 to 1.7) | 381 (249 to 685) | 19.2 (12.7 to 33.7) | 4 (2 to 7) | 0.2 (0.1 to 0.4) | 384 (252 to 694) | 19.4 (12.9 to 34.1) |
| Algeria | Both | 552 (462 to 695) | 4.5 (3.7 to 5.8) | 564 (472 to 721) | 4.7 (3.9 to 6.2) | 13634 (11515 to 16687) | 101.3 (85.3 to 125.5) | 118 (77 to 166) | 0.9 (0.6 to 1.3) | 13752 (11606 to 16851) | 102.2 (85.8 to 126.9) | 1099 (912 to 1302) | 3.4 (2.9 to 4.1) | 1135 (945 to 1343) | 3.6 (3.1 to 4.3) | 25623 (20713 to 30264) | 75 (61.2 to 88.3) | 240 (157 to 330) | 0.7 (0.5 to 1) | 25863 (20896 to 30535) | 75.7 (61.8 to 89.3) |
|  | Female | 425 (347 to 546) | 6.7 (5.5 to 9) | 432 (352 to 568) | 7.1 (5.8 to 9.7) | 10613 (8604 to 13119) | 154.2 (125.6 to 194.6) | 89 (56 to 129) | 1.4 (0.9 to 2) | 10702 (8669 to 13237) | 155.6 (126.5 to 196.3) | 798 (654 to 977) | 5 (4.2 to 6.2) | 822 (681 to 1015) | 5.3 (4.4 to 6.7) | 18936 (15032 to 22865) | 111.8 (90.9 to 134.7) | 172 (112 to 242) | 1.1 (0.7 to 1.5) | 19108 (15179 to 23026) | 112.9 (91.7 to 136.1) |
|  | Male | 127 (98 to 170) | 2.2 (1.7 to 2.8) | 131 (100 to 174) | 2.3 (1.8 to 3) | 3021 (2311 to 4123) | 46.2 (35.5 to 62.2) | 28 (19 to 41) | 0.5 (0.3 to 0.7) | 3050 (2337 to 4157) | 46.7 (36 to 62.8) | 301 (212 to 365) | 1.9 (1.3 to 2.3) | 313 (220 to 375) | 2 (1.4 to 2.4) | 6687 (4810 to 8176) | 39.2 (27.7 to 47.7) | 68 (43 to 94) | 0.4 (0.3 to 0.6) | 6755 (4863 to 8233) | 39.6 (28 to 48.1) |
| American Samoa | Both | 0 (0 to 0) | 1.4 (1.1 to 1.7) | 0 (0 to 0) | 1.5 (1.2 to 1.8) | 7 (6 to 9) | 29.6 (23.6 to 35.7) | 0 (0 to 0) | 0.3 (0.2 to 0.4) | 7 (6 to 9) | 29.9 (23.9 to 36) | 0 (0 to 1) | 1.1 (0.9 to 1.4) | 0 (0 to 1) | 1 (0.9 to 1.4) | 10 (9 to 13) | 22.3 (19.1 to 28) | 0 (0 to 0) | 0.2 (0.2 to 0.3) | 10 (9 to 13) | 22.5 (19.3 to 28.3) |
|  | Female | 0 (0 to 0) | 1.7 (1.3 to 2.3) | 0 (0 to 0) | 1.7 (1.3 to 2.4) | 4 (3 to 6) | 36.4 (28.1 to 48.8) | 0 (0 to 0) | 0.4 (0.2 to 0.6) | 5 (4 to 6) | 36.8 (28.4 to 49.2) | 0 (0 to 0) | 1.3 (1.1 to 1.8) | 0 (0 to 0) | 1.3 (1.1 to 1.8) | 7 (6 to 8) | 28.4 (23.6 to 36.4) | 0 (0 to 0) | 0.3 (0.2 to 0.4) | 7 (6 to 9) | 28.7 (23.9 to 36.8) |
|  | Male | 0 (0 to 0) | 1.1 (0.7 to 1.4) | 0 (0 to 0) | 1.2 (0.8 to 1.5) | 3 (2 to 4) | 23.2 (15.4 to 28.9) | 0 (0 to 0) | 0.2 (0.1 to 0.4) | 3 (2 to 4) | 23.4 (15.5 to 29.2) | 0 (0 to 0) | 0.8 (0.6 to 1.1) | 0 (0 to 0) | 0.8 (0.6 to 1.1) | 4 (3 to 5) | 16 (12.2 to 21.8) | 0 (0 to 0) | 0.2 (0.1 to 0.3) | 4 (3 to 5) | 16.2 (12.3 to 22) |
| Andean Latin America | Both | 1001 (882 to 1116) | 4.9 (4.3 to 5.5) | 1045 (927 to 1167) | 5.2 (4.7 to 5.9) | 24089 (21029 to 26625) | 110.4 (96.7 to 122.3) | 215 (145 to 285) | 1 (0.7 to 1.4) | 24304 (21229 to 26864) | 111.4 (97.7 to 123.5) | 1785 (1569 to 2182) | 3.3 (2.9 to 4.1) | 1891 (1666 to 2329) | 3.6 (3.1 to 4.4) | 39404 (34430 to 47672) | 72.6 (63.5 to 87.9) | 389 (263 to 534) | 0.7 (0.5 to 1) | 39793 (34820 to 48131) | 73.3 (64.1 to 88.7) |
|  | Female | 716 (592 to 802) | 6.7 (5.6 to 7.6) | 745 (618 to 834) | 7.2 (6 to 8.1) | 17218 (14058 to 19217) | 154 (126.3 to 171.3) | 151 (101 to 206) | 1.4 (0.9 to 1.9) | 17369 (14202 to 19385) | 155.4 (127.5 to 172.9) | 1222 (1040 to 1547) | 4.4 (3.7 to 5.6) | 1294 (1102 to 1642) | 4.7 (4 to 5.9) | 27172 (22966 to 33982) | 97.3 (82.2 to 121.6) | 263 (174 to 370) | 0.9 (0.6 to 1.3) | 27436 (23159 to 34307) | 98.2 (83 to 122.8) |
|  | Male | 285 (236 to 364) | 2.9 (2.4 to 3.7) | 300 (248 to 383) | 3.2 (2.6 to 4) | 6871 (5635 to 8825) | 64.5 (53.2 to 82.9) | 63 (42 to 88) | 0.6 (0.4 to 0.9) | 6935 (5688 to 8886) | 65.2 (53.7 to 83.5) | 563 (472 to 736) | 2.2 (1.8 to 2.9) | 597 (501 to 788) | 2.4 (2 to 3.1) | 12232 (10211 to 15933) | 46.4 (38.7 to 60.6) | 125 (82 to 179) | 0.5 (0.3 to 0.7) | 12357 (10332 to 16079) | 46.9 (39.2 to 61.2) |
| Andorra | Both | 2 (1 to 3) | 3.1 (2.4 to 4.3) | 1 (1 to 1) | 2.1 (1.7 to 2.7) | 23 (18 to 29) | 38.2 (30.8 to 48.4) | 0 (0 to 1) | 0.8 (0.5 to 1.2) | 23 (19 to 29) | 39 (31.5 to 49.3) | 3 (2 to 4) | 2.2 (1.7 to 3.1) | 2 (2 to 3) | 1.6 (1.3 to 2.2) | 38 (31 to 54) | 29.2 (23.1 to 41) | 1 (0 to 1) | 0.5 (0.3 to 0.8) | 39 (31 to 55) | 29.7 (23.5 to 41.6) |
|  | Female | 1 (1 to 1) | 3 (2 to 4.5) | 1 (0 to 1) | 2.2 (1.5 to 3) | 11 (8 to 16) | 39.2 (26.8 to 55.9) | 0 (0 to 0) | 0.7 (0.4 to 1.2) | 12 (8 to 16) | 39.9 (27.5 to 57.1) | 2 (1 to 2) | 2.1 (1.4 to 3.3) | 1 (1 to 2) | 1.7 (1.2 to 2.6) | 20 (14 to 31) | 29.9 (21.5 to 46.4) | 0 (0 to 1) | 0.5 (0.3 to 0.8) | 20 (15 to 31) | 30.4 (21.9 to 47.3) |
|  | Male | 1 (1 to 2) | 3.3 (2.3 to 5.2) | 1 (0 to 1) | 2 (1.6 to 2.7) | 12 (9 to 15) | 37.3 (28.2 to 48.9) | 0 (0 to 0) | 0.9 (0.5 to 1.5) | 12 (9 to 16) | 38.2 (28.7 to 50.1) | 2 (1 to 2) | 2.3 (1.5 to 3.7) | 1 (1 to 2) | 1.6 (1.1 to 2.5) | 19 (13 to 29) | 28.4 (20.1 to 44.4) | 0 (0 to 1) | 0.6 (0.3 to 1) | 19 (14 to 30) | 29 (20.7 to 45.2) |
| Angola | Both | 56 (38 to 75) | 1.6 (1.1 to 2.1) | 57 (39 to 76) | 1.7 (1.2 to 2.2) | 1494 (987 to 2038) | 35.6 (24.5 to 47.3) | 13 (8 to 19) | 0.3 (0.2 to 0.5) | 1506 (997 to 2057) | 35.9 (24.7 to 47.8) | 127 (99 to 163) | 1.3 (1.1 to 1.7) | 130 (102 to 166) | 1.5 (1.1 to 1.9) | 3326 (2587 to 4249) | 29.9 (23.4 to 38.1) | 28 (18 to 41) | 0.3 (0.2 to 0.4) | 3354 (2607 to 4292) | 30.2 (23.6 to 38.5) |
|  | Female | 34 (21 to 50) | 1.9 (1.2 to 2.7) | 35 (21 to 50) | 2.1 (1.3 to 2.9) | 910 (544 to 1384) | 43.9 (27 to 63.3) | 8 (4 to 12) | 0.4 (0.2 to 0.6) | 918 (549 to 1394) | 44.4 (27.3 to 64) | 84 (59 to 114) | 1.6 (1.1 to 2.2) | 85 (61 to 117) | 1.7 (1.2 to 2.4) | 2172 (1535 to 2988) | 35.8 (25.3 to 48.8) | 19 (11 to 28) | 0.4 (0.2 to 0.5) | 2191 (1547 to 3011) | 36.2 (25.6 to 49.3) |
|  | Male | 22 (13 to 30) | 1.2 (0.8 to 1.6) | 22 (14 to 31) | 1.3 (0.9 to 1.8) | 584 (355 to 835) | 27.4 (17.2 to 37.9) | 5 (3 to 8) | 0.3 (0.2 to 0.4) | 588 (358 to 843) | 27.7 (17.4 to 38.3) | 44 (32 to 60) | 1 (0.7 to 1.4) | 44 (32 to 60) | 1.1 (0.8 to 1.5) | 1154 (838 to 1574) | 22.7 (16.6 to 31) | 10 (6 to 14) | 0.2 (0.1 to 0.3) | 1163 (845 to 1586) | 22.9 (16.7 to 31.3) |
| Antigua and Barbuda | Both | 2 (1 to 2) | 3.6 (2.7 to 4) | 2 (1 to 2) | 3.7 (2.8 to 4.1) | 41 (30 to 45) | 79.5 (59.1 to 87) | 0 (0 to 1) | 0.8 (0.5 to 1.1) | 41 (30 to 45) | 80.3 (59.7 to 88.2) | 1 (1 to 2) | 1.2 (1.1 to 1.7) | 1 (1 to 2) | 1.3 (1.2 to 1.8) | 27 (24 to 38) | 26.1 (23.3 to 36.9) | 0 (0 to 0) | 0.3 (0.2 to 0.4) | 27 (24 to 38) | 26.3 (23.6 to 37.3) |
|  | Female | 1 (1 to 1) | 3.6 (2.9 to 4.1) | 1 (1 to 1) | 3.7 (3 to 4.1) | 23 (19 to 25) | 82 (67.7 to 91.6) | 0 (0 to 0) | 0.8 (0.5 to 1.1) | 23 (19 to 26) | 82.8 (68.3 to 92.5) | 1 (1 to 1) | 1.5 (1.3 to 2) | 1 (1 to 1) | 1.5 (1.4 to 2.1) | 17 (15 to 24) | 31.2 (27.3 to 43.2) | 0 (0 to 0) | 0.3 (0.2 to 0.5) | 17 (15 to 24) | 31.5 (27.6 to 43.6) |
|  | Male | 1 (0 to 1) | 3.6 (1.8 to 4.1) | 1 (0 to 1) | 3.8 (1.9 to 4.3) | 18 (9 to 20) | 77.2 (39 to 87.9) | 0 (0 to 0) | 0.8 (0.4 to 1.1) | 18 (9 to 20) | 78 (39.4 to 88.8) | 0 (0 to 1) | 1 (0.8 to 1.7) | 0 (0 to 1) | 1 (0.8 to 1.7) | 10 (8 to 17) | 20.3 (16.9 to 35.3) | 0 (0 to 0) | 0.2 (0.1 to 0.4) | 10 (8 to 17) | 20.5 (17.1 to 35.7) |
| Argentina | Both | 2744 (2594 to 2895) | 8.4 (8 to 8.9) | 2843 (2687 to 2997) | 8.8 (8.3 to 9.3) | 59548 (56253 to 62464) | 179.2 (169.3 to 187.9) | 575 (391 to 761) | 1.8 (1.2 to 2.3) | 60123 (56727 to 63139) | 180.9 (170.5 to 189.9) | 2819 (2492 to 3251) | 5.3 (4.7 to 6.1) | 2903 (2577 to 3345) | 5.4 (4.8 to 6.2) | 57370 (50982 to 65317) | 110.2 (97.9 to 125.4) | 613 (414 to 844) | 1.2 (0.8 to 1.6) | 57983 (51503 to 66072) | 111.4 (98.8 to 126.9) |
|  | Female | 1900 (1763 to 2031) | 10.4 (9.6 to 11.1) | 1986 (1835 to 2117) | 10.8 (10 to 11.5) | 40758 (37926 to 43418) | 224 (208.6 to 238.3) | 393 (261 to 533) | 2.1 (1.4 to 2.9) | 41151 (38294 to 43858) | 226.2 (210.3 to 240.8) | 1756 (1486 to 2093) | 5.8 (4.9 to 7) | 1841 (1563 to 2177) | 5.9 (5 to 7) | 35636 (30179 to 42279) | 125.6 (106.3 to 149.3) | 377 (252 to 536) | 1.3 (0.8 to 1.8) | 36014 (30480 to 42740) | 126.9 (107.3 to 150.9) |
|  | Male | 844 (769 to 922) | 5.9 (5.4 to 6.5) | 857 (785 to 914) | 6.1 (5.6 to 6.6) | 18789 (17050 to 19992) | 125.1 (113.4 to 133.3) | 182 (123 to 252) | 1.3 (0.9 to 1.7) | 18972 (17186 to 20193) | 126.4 (114.3 to 134.6) | 1063 (899 to 1305) | 4.6 (3.9 to 5.6) | 1062 (907 to 1263) | 4.6 (4 to 5.5) | 21733 (18415 to 25988) | 92.7 (78.5 to 111) | 236 (152 to 330) | 1 (0.7 to 1.4) | 21969 (18593 to 26277) | 93.7 (79.3 to 112.2) |
| Armenia | Both | 21 (17 to 31) | 0.8 (0.6 to 1.2) | 22 (18 to 32) | 0.9 (0.7 to 1.2) | 493 (396 to 766) | 17.4 (14 to 26.5) | 5 (3 to 8) | 0.2 (0.1 to 0.3) | 497 (400 to 773) | 17.6 (14.1 to 26.7) | 59 (44 to 64) | 1.4 (1.1 to 1.5) | 65 (48 to 70) | 1.6 (1.1 to 1.7) | 1250 (933 to 1354) | 29.9 (22.3 to 32.2) | 13 (8 to 17) | 0.3 (0.2 to 0.4) | 1263 (943 to 1368) | 30.2 (22.6 to 32.6) |
|  | Female | 12 (10 to 20) | 0.8 (0.7 to 1.3) | 13 (11 to 21) | 0.9 (0.7 to 1.4) | 273 (224 to 440) | 17.1 (14.1 to 27.7) | 3 (2 to 5) | 0.2 (0.1 to 0.3) | 276 (226 to 445) | 17.3 (14.2 to 28) | 40 (27 to 44) | 1.6 (1.1 to 1.8) | 44 (29 to 48) | 1.8 (1.2 to 2) | 809 (554 to 893) | 33.9 (23.2 to 37.4) | 9 (5 to 12) | 0.4 (0.2 to 0.5) | 817 (559 to 903) | 34.2 (23.4 to 37.8) |
|  | Male | 9 (6 to 16) | 0.8 (0.6 to 1.4) | 9 (6 to 16) | 0.9 (0.6 to 1.5) | 220 (157 to 408) | 17.7 (12.7 to 32.3) | 2 (1 to 4) | 0.2 (0.1 to 0.3) | 222 (158 to 411) | 17.9 (12.8 to 32.6) | 19 (15 to 22) | 1.1 (0.8 to 1.2) | 21 (15 to 23) | 1.2 (0.9 to 1.3) | 441 (342 to 493) | 24.3 (18.8 to 27.1) | 4 (3 to 6) | 0.2 (0.2 to 0.3) | 446 (345 to 499) | 24.6 (19 to 27.3) |
| Australasia | Both | 799 (689 to 967) | 3.3 (2.9 to 4) | 586 (547 to 606) | 2.4 (2.3 to 2.5) | 11061 (10303 to 11450) | 46.2 (42.9 to 47.8) | 192 (130 to 266) | 0.8 (0.5 to 1.1) | 11253 (10462 to 11642) | 47 (43.5 to 48.6) | 1213 (993 to 1571) | 2.4 (2 to 3.1) | 845 (764 to 936) | 1.7 (1.5 to 1.8) | 13687 (12310 to 15203) | 29.4 (26.4 to 32.7) | 296 (203 to 413) | 0.6 (0.4 to 0.8) | 13983 (12547 to 15535) | 30 (27 to 33.5) |
|  | Female | 462 (389 to 606) | 3.4 (2.9 to 4.4) | 351 (337 to 364) | 2.5 (2.4 to 2.6) | 6346 (6088 to 6609) | 48.9 (46.8 to 51) | 107 (71 to 153) | 0.8 (0.5 to 1.1) | 6453 (6195 to 6718) | 49.7 (47.7 to 51.8) | 680 (522 to 978) | 2.5 (1.9 to 3.4) | 474 (413 to 541) | 1.7 (1.5 to 1.9) | 7432 (6450 to 8508) | 30.3 (26.2 to 34.7) | 161 (108 to 236) | 0.6 (0.4 to 0.9) | 7593 (6581 to 8668) | 30.9 (26.7 to 35.4) |
|  | Male | 336 (268 to 433) | 3.2 (2.6 to 4.2) | 235 (200 to 247) | 2.3 (2 to 2.4) | 4715 (4006 to 4960) | 43.2 (36.5 to 45.4) | 85 (54 to 125) | 0.8 (0.5 to 1.1) | 4800 (4064 to 5067) | 44 (37.1 to 46.3) | 533 (407 to 739) | 2.4 (1.8 to 3.3) | 372 (322 to 427) | 1.6 (1.4 to 1.8) | 6255 (5442 to 7268) | 28.5 (24.7 to 33) | 134 (85 to 205) | 0.6 (0.4 to 0.9) | 6390 (5560 to 7414) | 29.1 (25.3 to 33.8) |
| Australia | Both | 676 (578 to 837) | 3.4 (2.9 to 4.2) | 500 (475 to 518) | 2.5 (2.4 to 2.6) | 9437 (8892 to 9823) | 47.3 (44.3 to 49.2) | 162 (110 to 226) | 0.8 (0.5 to 1.1) | 9599 (9015 to 9977) | 48.1 (45.1 to 50) | 1019 (806 to 1374) | 2.4 (1.9 to 3.2) | 726 (648 to 816) | 1.7 (1.5 to 1.9) | 11668 (10346 to 13229) | 29.8 (26.3 to 33.8) | 247 (169 to 355) | 0.6 (0.4 to 0.8) | 11915 (10562 to 13487) | 30.4 (26.9 to 34.4) |
|  | Female | 387 (319 to 532) | 3.4 (2.8 to 4.6) | 298 (285 to 311) | 2.6 (2.5 to 2.7) | 5384 (5139 to 5636) | 49.9 (47.6 to 52.2) | 90 (58 to 132) | 0.8 (0.5 to 1.2) | 5473 (5221 to 5727) | 50.7 (48.3 to 53.1) | 557 (411 to 853) | 2.4 (1.8 to 3.5) | 407 (346 to 472) | 1.7 (1.5 to 2) | 6321 (5363 to 7394) | 30.6 (25.8 to 35.9) | 130 (83 to 200) | 0.6 (0.4 to 0.8) | 6451 (5469 to 7544) | 31.2 (26.3 to 36.6) |
|  | Male | 288 (228 to 384) | 3.3 (2.6 to 4.4) | 202 (176 to 213) | 2.4 (2.1 to 2.5) | 4054 (3467 to 4288) | 44.5 (38.1 to 47.1) | 72 (46 to 109) | 0.8 (0.5 to 1.2) | 4126 (3523 to 4370) | 45.3 (38.7 to 47.9) | 462 (339 to 671) | 2.4 (1.8 to 3.5) | 319 (272 to 374) | 1.6 (1.4 to 1.9) | 5347 (4554 to 6375) | 28.9 (24.6 to 34.3) | 116 (72 to 184) | 0.6 (0.4 to 1) | 5463 (4641 to 6513) | 29.5 (25 to 35.1) |
| Austria | Both | 671 (552 to 799) | 5.4 (4.5 to 6.4) | 558 (489 to 584) | 4.4 (3.8 to 4.6) | 9510 (8268 to 9946) | 79.5 (69.3 to 83.2) | 151 (101 to 214) | 1.2 (0.8 to 1.7) | 9661 (8383 to 10115) | 80.7 (70.1 to 84.6) | 617 (509 to 782) | 3.3 (2.8 to 4.2) | 412 (372 to 446) | 2.2 (1.9 to 2.3) | 6491 (5853 to 7050) | 38.2 (34.5 to 41.4) | 153 (105 to 212) | 0.9 (0.6 to 1.2) | 6644 (5989 to 7217) | 39 (35.4 to 42.4) |
|  | Female | 479 (397 to 594) | 6.1 (5.1 to 7.6) | 407 (368 to 429) | 5 (4.5 to 5.3) | 6652 (5912 to 7034) | 92.2 (81.3 to 97.6) | 105 (68 to 153) | 1.4 (0.9 to 2) | 6757 (6022 to 7136) | 93.6 (82.9 to 99.1) | 336 (261 to 483) | 3.1 (2.5 to 4.2) | 230 (206 to 260) | 2.1 (1.8 to 2.3) | 3426 (3061 to 3862) | 36.7 (32.7 to 41.4) | 81 (52 to 120) | 0.8 (0.5 to 1.1) | 3507 (3140 to 3950) | 37.5 (33.5 to 42.4) |
|  | Male | 192 (135 to 241) | 4.2 (3 to 5.3) | 152 (107 to 163) | 3.4 (2.4 to 3.6) | 2858 (2081 to 3078) | 61.5 (44.6 to 66.1) | 46 (29 to 66) | 1 (0.6 to 1.4) | 2904 (2113 to 3122) | 62.5 (45.3 to 67.2) | 281 (189 to 391) | 3.6 (2.4 to 4.9) | 181 (129 to 204) | 2.3 (1.7 to 2.6) | 3065 (2226 to 3472) | 40.1 (29.4 to 45.4) | 73 (42 to 111) | 0.9 (0.5 to 1.4) | 3137 (2283 to 3568) | 41 (30.2 to 46.6) |
| Azerbaijan | Both | 68 (55 to 94) | 1.4 (1.1 to 1.9) | 70 (55 to 98) | 1.4 (1.1 to 2.1) | 1657 (1384 to 2154) | 31.3 (25.7 to 41.5) | 15 (10 to 22) | 0.3 (0.2 to 0.4) | 1672 (1395 to 2174) | 31.6 (25.9 to 42) | 127 (106 to 151) | 1.4 (1.2 to 1.7) | 130 (109 to 155) | 1.5 (1.3 to 1.8) | 3273 (2633 to 3877) | 32.8 (27.2 to 38.6) | 28 (18 to 39) | 0.3 (0.2 to 0.4) | 3301 (2654 to 3917) | 33.1 (27.5 to 39) |
|  | Female | 46 (35 to 67) | 1.6 (1.2 to 2.3) | 48 (36 to 72) | 1.6 (1.2 to 2.5) | 1097 (877 to 1505) | 36.2 (28.6 to 50.5) | 10 (6 to 16) | 0.3 (0.2 to 0.5) | 1107 (885 to 1523) | 36.5 (28.9 to 51) | 80 (58 to 97) | 1.5 (1.2 to 1.9) | 81 (60 to 99) | 1.6 (1.2 to 2) | 2072 (1451 to 2580) | 37.3 (26.8 to 45.9) | 18 (11 to 25) | 0.3 (0.2 to 0.5) | 2090 (1464 to 2604) | 37.6 (27.1 to 46.4) |
|  | Male | 21 (17 to 31) | 1.1 (0.8 to 1.6) | 22 (17 to 32) | 1.1 (0.9 to 1.7) | 560 (452 to 789) | 24.6 (19.7 to 35.7) | 5 (3 to 7) | 0.2 (0.1 to 0.4) | 565 (457 to 795) | 24.8 (19.9 to 36) | 47 (36 to 65) | 1.3 (1 to 1.9) | 49 (37 to 68) | 1.4 (1.1 to 2.1) | 1201 (898 to 1647) | 28.2 (21.7 to 38.8) | 11 (7 to 16) | 0.3 (0.2 to 0.4) | 1211 (908 to 1662) | 28.5 (21.9 to 39.2) |
| Bahrain | Both | 2 (2 to 2) | 1.2 (0.9 to 1.5) | 2 (2 to 2) | 1.3 (1 to 1.6) | 53 (40 to 62) | 26.1 (19.1 to 31.1) | 0 (0 to 1) | 0.3 (0.2 to 0.4) | 53 (40 to 62) | 26.3 (19.3 to 31.5) | 6 (4 to 7) | 0.7 (0.5 to 0.8) | 6 (4 to 7) | 0.8 (0.6 to 0.9) | 153 (110 to 183) | 14.5 (10.9 to 17.1) | 1 (1 to 2) | 0.2 (0.1 to 0.2) | 155 (110 to 185) | 14.7 (11 to 17.3) |
|  | Female | 1 (1 to 1) | 1.3 (0.9 to 1.5) | 1 (1 to 1) | 1.3 (1 to 1.6) | 23 (17 to 28) | 26.9 (18.8 to 31.7) | 0 (0 to 0) | 0.3 (0.2 to 0.4) | 23 (17 to 28) | 27.1 (19 to 32.1) | 3 (2 to 3) | 0.8 (0.5 to 1) | 3 (2 to 3) | 0.9 (0.6 to 1) | 62 (40 to 75) | 16.5 (10.7 to 19.9) | 1 (0 to 1) | 0.2 (0.1 to 0.3) | 63 (41 to 75) | 16.7 (10.8 to 20.2) |
|  | Male | 1 (1 to 2) | 1.2 (0.7 to 1.6) | 1 (1 to 2) | 1.3 (0.7 to 1.8) | 30 (18 to 38) | 25.5 (14.3 to 34.1) | 0 (0 to 0) | 0.3 (0.1 to 0.4) | 30 (18 to 38) | 25.7 (14.4 to 34.4) | 4 (3 to 4) | 0.7 (0.5 to 0.8) | 3 (2 to 4) | 0.7 (0.5 to 0.9) | 91 (65 to 118) | 13.1 (9.4 to 16.3) | 1 (1 to 1) | 0.1 (0.1 to 0.2) | 92 (66 to 119) | 13.2 (9.5 to 16.5) |
| Bangladesh | Both | 1153 (846 to 1927) | 2.5 (1.8 to 4.2) | 1191 (876 to 1990) | 2.7 (2 to 4.4) | 29629 (21681 to 49377) | 58.1 (42.6 to 97.4) | 257 (157 to 458) | 0.5 (0.3 to 1) | 29887 (21808 to 49764) | 58.6 (43 to 98.2) | 2217 (1745 to 2863) | 1.9 (1.5 to 2.4) | 2304 (1816 to 2959) | 2 (1.6 to 2.5) | 55369 (42901 to 72097) | 43.1 (33.6 to 55.8) | 497 (311 to 710) | 0.4 (0.3 to 0.6) | 55865 (43301 to 72581) | 43.5 (34 to 56.4) |
|  | Female | 661 (449 to 1248) | 3.1 (2.1 to 5.9) | 678 (461 to 1267) | 3.3 (2.3 to 6.3) | 17319 (11549 to 32756) | 74.2 (50.1 to 142.2) | 147 (85 to 288) | 0.7 (0.4 to 1.4) | 17467 (11637 to 33028) | 74.9 (50.5 to 143.4) | 1316 (956 to 1912) | 2.3 (1.7 to 3.3) | 1361 (986 to 1964) | 2.4 (1.8 to 3.5) | 33596 (24340 to 49337) | 53.7 (38.8 to 78.4) | 295 (173 to 458) | 0.5 (0.3 to 0.8) | 33891 (24577 to 49710) | 54.2 (39.2 to 79.1) |
|  | Male | 492 (358 to 758) | 2 (1.4 to 3) | 513 (374 to 786) | 2.1 (1.6 to 3.2) | 12310 (8926 to 18994) | 44.8 (32.5 to 69.1) | 110 (68 to 168) | 0.4 (0.3 to 0.7) | 12420 (9016 to 19218) | 45.2 (32.9 to 69.8) | 901 (598 to 1185) | 1.5 (1 to 1.9) | 943 (625 to 1232) | 1.6 (1 to 2) | 21773 (14002 to 29670) | 33.1 (21.6 to 44.7) | 202 (118 to 304) | 0.3 (0.2 to 0.5) | 21975 (14132 to 29926) | 33.4 (21.7 to 45.1) |
| Barbados | Both | 12 (10 to 14) | 4.1 (3.3 to 4.5) | 13 (10 to 14) | 4.3 (3.3 to 4.6) | 248 (193 to 268) | 87.5 (68.6 to 94.8) | 3 (2 to 4) | 0.9 (0.6 to 1.2) | 250 (196 to 271) | 88.4 (69.3 to 95.6) | 6 (6 to 9) | 1.3 (1.2 to 2) | 7 (6 to 10) | 1.4 (1.2 to 2) | 131 (114 to 190) | 27.8 (24.2 to 40.2) | 1 (1 to 2) | 0.3 (0.2 to 0.4) | 133 (115 to 192) | 28.1 (24.4 to 40.6) |
|  | Female | 8 (7 to 9) | 4.6 (3.7 to 5) | 9 (7 to 9) | 4.7 (3.8 to 5.1) | 157 (128 to 173) | 98.5 (81.6 to 107.9) | 2 (1 to 2) | 1 (0.6 to 1.3) | 159 (130 to 174) | 99.5 (82.7 to 109.1) | 4 (4 to 6) | 1.6 (1.3 to 2.2) | 5 (4 to 6) | 1.7 (1.4 to 2.3) | 87 (73 to 119) | 33.8 (28.6 to 46.6) | 1 (1 to 1) | 0.4 (0.2 to 0.5) | 88 (74 to 121) | 34.1 (28.9 to 47.1) |
|  | Male | 4 (3 to 5) | 3.6 (2 to 4) | 5 (3 to 5) | 3.8 (2.1 to 4.2) | 90 (53 to 100) | 73.8 (43.3 to 82.3) | 1 (1 to 1) | 0.8 (0.4 to 1.1) | 91 (54 to 101) | 74.6 (43.7 to 83.2) | 2 (2 to 4) | 1 (0.8 to 1.9) | 2 (2 to 4) | 1.1 (0.9 to 2) | 44 (36 to 83) | 20.7 (16.9 to 38.9) | 0 (0 to 1) | 0.2 (0.1 to 0.4) | 45 (37 to 84) | 21 (17.1 to 39.4) |
| Belarus | Both | 189 (174 to 208) | 1.4 (1.3 to 1.6) | 195 (181 to 214) | 1.5 (1.4 to 1.6) | 4085 (3778 to 4502) | 30.7 (28.5 to 33.7) | 42 (29 to 57) | 0.3 (0.2 to 0.4) | 4127 (3817 to 4547) | 31.1 (28.9 to 34.1) | 235 (207 to 272) | 1.5 (1.3 to 1.7) | 232 (207 to 262) | 1.4 (1.3 to 1.6) | 4460 (3933 to 5067) | 28.3 (24.9 to 32.1) | 54 (36 to 73) | 0.3 (0.2 to 0.5) | 4514 (3975 to 5128) | 28.6 (25.2 to 32.5) |
|  | Female | 129 (118 to 144) | 1.5 (1.4 to 1.7) | 134 (123 to 146) | 1.5 (1.4 to 1.7) | 2650 (2426 to 2909) | 32 (29.3 to 35.1) | 29 (19 to 40) | 0.3 (0.2 to 0.5) | 2679 (2451 to 2941) | 32.4 (29.6 to 35.5) | 155 (132 to 189) | 1.5 (1.3 to 1.8) | 151 (133 to 173) | 1.4 (1.2 to 1.6) | 2677 (2323 to 3123) | 27.5 (23.9 to 32.1) | 35 (23 to 49) | 0.3 (0.2 to 0.5) | 2712 (2355 to 3161) | 27.8 (24.2 to 32.5) |
|  | Male | 59 (53 to 74) | 1.3 (1.1 to 1.5) | 61 (54 to 76) | 1.3 (1.2 to 1.6) | 1435 (1259 to 1787) | 28.4 (25.1 to 35.5) | 13 (9 to 18) | 0.3 (0.2 to 0.4) | 1448 (1271 to 1808) | 28.6 (25.3 to 35.9) | 81 (66 to 96) | 1.4 (1.1 to 1.6) | 81 (68 to 96) | 1.4 (1.2 to 1.7) | 1783 (1468 to 2128) | 29.2 (24.3 to 34.7) | 18 (12 to 26) | 0.3 (0.2 to 0.4) | 1801 (1485 to 2152) | 29.5 (24.5 to 35.1) |
| Belgium | Both | 595 (491 to 755) | 3.7 (3.1 to 4.7) | 458 (414 to 478) | 2.8 (2.5 to 2.9) | 7639 (6964 to 7992) | 49 (45.1 to 51.4) | 140 (92 to 198) | 0.9 (0.6 to 1.2) | 7780 (7105 to 8147) | 49.9 (45.8 to 52.2) | 428 (363 to 536) | 1.8 (1.6 to 2.3) | 359 (330 to 396) | 1.4 (1.3 to 1.6) | 5389 (4943 to 6021) | 24.9 (22.8 to 28.1) | 102 (68 to 145) | 0.5 (0.3 to 0.6) | 5491 (5024 to 6135) | 25.4 (23.2 to 28.5) |
|  | Female | 337 (271 to 460) | 3.4 (2.8 to 4.5) | 283 (263 to 301) | 2.8 (2.6 to 3) | 4375 (4079 to 4652) | 48.1 (44.8 to 51.1) | 74 (48 to 109) | 0.8 (0.5 to 1.1) | 4450 (4149 to 4726) | 48.9 (45.7 to 52) | 208 (172 to 277) | 1.5 (1.2 to 1.9) | 193 (173 to 216) | 1.3 (1.1 to 1.4) | 2619 (2338 to 2954) | 21.7 (19.2 to 24.8) | 47 (30 to 69) | 0.4 (0.2 to 0.5) | 2666 (2377 to 2999) | 22.1 (19.5 to 25.1) |
|  | Male | 258 (199 to 368) | 4 (3.1 to 5.5) | 174 (146 to 187) | 2.8 (2.3 to 3) | 3264 (2812 to 3520) | 49.8 (42.6 to 53.7) | 66 (41 to 104) | 1 (0.6 to 1.5) | 3330 (2859 to 3593) | 50.9 (43.4 to 54.9) | 220 (174 to 297) | 2.2 (1.8 to 3) | 166 (146 to 191) | 1.6 (1.4 to 1.8) | 2770 (2425 to 3234) | 28.5 (24.9 to 33.4) | 56 (36 to 83) | 0.6 (0.4 to 0.8) | 2825 (2470 to 3288) | 29 (25.4 to 34) |
| Belize | Both | 3 (2 to 3) | 3 (2.5 to 3.2) | 3 (3 to 3) | 3.2 (2.7 to 3.5) | 62 (54 to 68) | 64.7 (55.5 to 70.7) | 1 (0 to 1) | 0.6 (0.4 to 0.9) | 63 (54 to 69) | 65.3 (55.9 to 71.5) | 3 (3 to 5) | 1.3 (1.1 to 1.8) | 3 (3 to 5) | 1.3 (1.2 to 1.9) | 82 (73 to 115) | 29.4 (26.2 to 41.3) | 1 (0 to 1) | 0.3 (0.2 to 0.4) | 83 (74 to 116) | 29.7 (26.4 to 41.7) |
|  | Female | 2 (2 to 2) | 4.1 (3.4 to 4.6) | 2 (2 to 2) | 4.3 (3.6 to 4.8) | 43 (37 to 49) | 91.5 (77.7 to 102.4) | 0 (0 to 1) | 0.9 (0.6 to 1.2) | 44 (37 to 49) | 92.4 (78.5 to 103.4) | 2 (2 to 3) | 1.7 (1.5 to 2.2) | 2 (2 to 3) | 1.7 (1.5 to 2.3) | 53 (46 to 71) | 38.9 (34 to 52) | 0 (0 to 1) | 0.4 (0.2 to 0.5) | 54 (47 to 72) | 39.3 (34.3 to 52.6) |
|  | Male | 1 (1 to 1) | 1.8 (1.3 to 2.1) | 1 (1 to 1) | 2 (1.4 to 2.3) | 19 (13 to 22) | 38.7 (27.6 to 45.5) | 0 (0 to 0) | 0.4 (0.2 to 0.6) | 19 (14 to 23) | 39.1 (27.9 to 46) | 1 (1 to 2) | 0.9 (0.8 to 1.7) | 1 (1 to 2) | 0.9 (0.8 to 1.8) | 29 (25 to 51) | 20.3 (17.4 to 37) | 0 (0 to 0) | 0.2 (0.1 to 0.4) | 29 (25 to 52) | 20.5 (17.6 to 37.4) |
| Benin | Both | 31 (26 to 39) | 1.6 (1.3 to 2) | 33 (27 to 41) | 1.7 (1.4 to 2.2) | 705 (583 to 904) | 34.3 (28.3 to 43.8) | 7 (5 to 10) | 0.4 (0.2 to 0.5) | 712 (588 to 914) | 34.6 (28.7 to 44.3) | 54 (44 to 73) | 1.3 (1 to 1.7) | 57 (46 to 76) | 1.4 (1.1 to 1.9) | 1278 (1015 to 1730) | 27.3 (21.8 to 36.6) | 12 (8 to 18) | 0.3 (0.2 to 0.4) | 1290 (1025 to 1746) | 27.6 (22 to 37) |
|  | Female | 17 (14 to 24) | 1.8 (1.4 to 2.4) | 19 (15 to 25) | 1.9 (1.6 to 2.6) | 394 (312 to 546) | 37.6 (29.9 to 51.9) | 4 (2 to 6) | 0.4 (0.3 to 0.6) | 398 (315 to 550) | 38 (30.3 to 52.4) | 32 (23 to 45) | 1.4 (1.1 to 2) | 34 (25 to 47) | 1.6 (1.2 to 2.1) | 742 (544 to 1044) | 30.1 (22.1 to 42) | 7 (4 to 11) | 0.3 (0.2 to 0.5) | 749 (549 to 1055) | 30.4 (22.3 to 42.5) |
|  | Male | 14 (10 to 18) | 1.4 (1 to 1.9) | 14 (11 to 19) | 1.5 (1.1 to 2) | 310 (230 to 412) | 30.8 (22.9 to 40.7) | 3 (2 to 4) | 0.3 (0.2 to 0.5) | 313 (233 to 416) | 31.1 (23.1 to 41) | 22 (17 to 33) | 1.1 (0.9 to 1.7) | 23 (17 to 34) | 1.2 (0.9 to 1.8) | 537 (397 to 810) | 24 (18 to 35.7) | 5 (3 to 8) | 0.2 (0.2 to 0.4) | 542 (400 to 819) | 24.3 (18.2 to 36.1) |
| Bermuda | Both | 2 (2 to 3) | 3.6 (2.6 to 4) | 2 (2 to 3) | 3.7 (2.7 to 4.1) | 48 (35 to 53) | 74.3 (54.1 to 81.4) | 1 (0 to 1) | 0.8 (0.5 to 1.1) | 49 (36 to 53) | 75.1 (54.7 to 82.3) | 1 (1 to 2) | 0.8 (0.7 to 1.2) | 1 (1 to 2) | 0.8 (0.7 to 1.2) | 18 (15 to 28) | 14.8 (12.7 to 23) | 0 (0 to 0) | 0.2 (0.1 to 0.3) | 18 (16 to 28) | 15 (12.9 to 23.3) |
|  | Female | 1 (1 to 2) | 3.9 (3 to 4.4) | 1 (1 to 2) | 4 (3.1 to 4.4) | 29 (22 to 32) | 81 (62.2 to 90.1) | 0 (0 to 0) | 0.9 (0.6 to 1.2) | 30 (23 to 33) | 81.9 (62.9 to 91) | 1 (0 to 1) | 0.7 (0.6 to 1) | 1 (0 to 1) | 0.8 (0.6 to 1.1) | 9 (8 to 13) | 13.7 (11.3 to 19.2) | 0 (0 to 0) | 0.2 (0.1 to 0.2) | 9 (8 to 13) | 13.9 (11.4 to 19.4) |
|  | Male | 1 (0 to 1) | 3.3 (1.4 to 3.8) | 1 (0 to 1) | 3.4 (1.5 to 3.9) | 19 (8 to 22) | 66.3 (29 to 76.5) | 0 (0 to 0) | 0.7 (0.3 to 1) | 19 (8 to 22) | 67 (29.3 to 77.4) | 0 (0 to 1) | 0.9 (0.7 to 1.6) | 0 (0 to 1) | 0.9 (0.7 to 1.5) | 9 (7 to 16) | 16.1 (13.6 to 30.3) | 0 (0 to 0) | 0.2 (0.1 to 0.4) | 9 (7 to 16) | 16.3 (13.8 to 30.8) |
| Bhutan | Both | 6 (5 to 11) | 2.6 (1.9 to 4.4) | 6 (5 to 11) | 2.8 (2.1 to 4.8) | 160 (116 to 275) | 59 (43.1 to 101) | 1 (1 to 2) | 0.6 (0.3 to 1) | 162 (117 to 277) | 59.6 (43.5 to 101.9) | 15 (11 to 20) | 2.6 (1.9 to 3.5) | 16 (12 to 21) | 2.8 (2.1 to 3.8) | 356 (260 to 479) | 56.6 (41.6 to 75.9) | 3 (2 to 5) | 0.6 (0.4 to 0.8) | 360 (262 to 483) | 57.2 (42 to 76.7) |
|  | Female | 4 (3 to 8) | 3.3 (2.2 to 6.4) | 4 (3 to 8) | 3.6 (2.3 to 6.9) | 102 (68 to 188) | 76.3 (50.8 to 143.2) | 1 (0 to 2) | 0.7 (0.4 to 1.5) | 103 (68 to 190) | 77.1 (51.3 to 144.5) | 9 (6 to 14) | 3.1 (2.2 to 4.7) | 9 (7 to 14) | 3.4 (2.4 to 5.1) | 213 (149 to 315) | 70 (49 to 104.4) | 2 (1 to 3) | 0.7 (0.4 to 1.1) | 215 (151 to 318) | 70.7 (49.4 to 105.3) |
|  | Male | 2 (2 to 4) | 1.9 (1.4 to 2.9) | 2 (2 to 4) | 2 (1.5 to 3.2) | 58 (42 to 90) | 42.3 (31 to 64.9) | 1 (0 to 1) | 0.4 (0.2 to 0.7) | 59 (43 to 90) | 42.8 (31.4 to 65.5) | 6 (3 to 10) | 2.1 (1.2 to 3.3) | 6 (4 to 10) | 2.3 (1.3 to 3.5) | 143 (78 to 228) | 44.5 (24.5 to 70.5) | 1 (1 to 2) | 0.5 (0.2 to 0.8) | 144 (79 to 231) | 45 (24.7 to 71.2) |
| Bolivia | Both | 218 (164 to 277) | 7 (5.3 to 8.7) | 228 (172 to 285) | 7.6 (5.8 to 9.3) | 5353 (3968 to 6884) | 157 (117.5 to 199.7) | 45 (28 to 67) | 1.4 (0.9 to 2.1) | 5398 (3996 to 6939) | 158.4 (118.6 to 201) | 406 (319 to 521) | 4.9 (3.9 to 6.3) | 434 (342 to 552) | 5.3 (4.2 to 6.8) | 9351 (7304 to 12040) | 106.1 (82.8 to 136.7) | 87 (56 to 126) | 1 (0.7 to 1.5) | 9438 (7372 to 12156) | 107.1 (83.6 to 138) |
|  | Female | 156 (106 to 211) | 9.4 (6.4 to 12.3) | 163 (111 to 217) | 10.1 (6.9 to 13.1) | 3836 (2604 to 5291) | 212.6 (144.3 to 288.3) | 32 (19 to 50) | 1.9 (1.1 to 2.9) | 3868 (2623 to 5335) | 214.4 (145.7 to 290.8) | 280 (206 to 377) | 6.3 (4.7 to 8.4) | 298 (221 to 399) | 6.8 (5.1 to 9.1) | 6464 (4666 to 8746) | 139.6 (101.2 to 188.4) | 59 (35 to 91) | 1.3 (0.8 to 2) | 6523 (4699 to 8833) | 140.9 (101.9 to 189.7) |
|  | Male | 62 (39 to 88) | 4.3 (2.8 to 6) | 65 (41 to 92) | 4.7 (3 to 6.5) | 1517 (947 to 2191) | 94.6 (60.1 to 135.2) | 13 (7 to 21) | 0.9 (0.5 to 1.4) | 1530 (955 to 2211) | 95.5 (60.6 to 136.8) | 126 (87 to 181) | 3.3 (2.3 to 4.7) | 136 (95 to 194) | 3.6 (2.6 to 5.2) | 2888 (1987 to 4132) | 69.5 (48.4 to 99.3) | 28 (16 to 42) | 0.7 (0.4 to 1.1) | 2916 (2006 to 4165) | 70.2 (48.8 to 100.1) |
| Bosnia and Herzegovina | Both | 220 (169 to 243) | 5.5 (4.3 to 6.1) | 229 (177 to 252) | 5.9 (4.7 to 6.5) | 5302 (3975 to 5864) | 122.1 (93 to 134.2) | 47 (30 to 65) | 1.1 (0.7 to 1.6) | 5349 (4012 to 5917) | 123.2 (93.9 to 135.7) | 192 (154 to 325) | 3.2 (2.6 to 5.4) | 210 (169 to 356) | 3.5 (2.8 to 5.9) | 4012 (3207 to 6798) | 66.4 (53.4 to 112.4) | 41 (24 to 74) | 0.7 (0.4 to 1.2) | 4053 (3241 to 6866) | 67.1 (53.9 to 113.5) |
|  | Female | 146 (106 to 165) | 6.4 (4.7 to 7.2) | 152 (111 to 170) | 6.8 (5.1 to 7.6) | 3455 (2457 to 3905) | 141.7 (101.8 to 159.3) | 30 (19 to 44) | 1.3 (0.8 to 1.9) | 3486 (2483 to 3951) | 143 (102.8 to 160.8) | 124 (98 to 226) | 3.6 (2.9 to 6.6) | 136 (107 to 247) | 4 (3.1 to 7.2) | 2556 (2013 to 4621) | 77.1 (60.8 to 139.1) | 26 (16 to 49) | 0.8 (0.5 to 1.4) | 2582 (2036 to 4679) | 77.8 (61.3 to 140.4) |
|  | Male | 74 (53 to 89) | 4.3 (3.1 to 5.3) | 77 (55 to 93) | 4.7 (3.4 to 5.8) | 1847 (1286 to 2194) | 96.7 (68.5 to 115.8) | 16 (10 to 23) | 0.9 (0.6 to 1.3) | 1863 (1300 to 2218) | 97.7 (69.2 to 117) | 67 (51 to 125) | 2.6 (2 to 4.8) | 74 (56 to 136) | 2.9 (2.2 to 5.3) | 1457 (1089 to 2726) | 53.9 (40.6 to 100.5) | 15 (8 to 29) | 0.6 (0.3 to 1.1) | 1471 (1100 to 2761) | 54.4 (41 to 101.9) |
| Botswana | Both | 7 (5 to 8) | 1.2 (1 to 1.5) | 7 (5 to 9) | 1.3 (1.1 to 1.7) | 161 (126 to 206) | 26.2 (20.6 to 33.4) | 1 (1 to 2) | 0.3 (0.2 to 0.4) | 162 (127 to 208) | 26.4 (20.9 to 33.8) | 12 (9 to 15) | 1 (0.8 to 1.2) | 13 (10 to 16) | 1.1 (0.9 to 1.3) | 273 (218 to 346) | 19.8 (15.8 to 24.9) | 3 (2 to 4) | 0.2 (0.1 to 0.3) | 275 (221 to 349) | 20 (16 to 25.1) |
|  | Female | 4 (3 to 6) | 1.4 (1 to 1.9) | 4 (3 to 6) | 1.5 (1.1 to 2) | 99 (68 to 138) | 29.3 (20.6 to 40.6) | 1 (1 to 1) | 0.3 (0.2 to 0.5) | 100 (69 to 140) | 29.6 (20.8 to 41) | 8 (6 to 11) | 1.2 (0.9 to 1.5) | 9 (7 to 11) | 1.3 (1 to 1.7) | 185 (140 to 242) | 23.9 (18.1 to 31.1) | 2 (1 to 3) | 0.3 (0.2 to 0.4) | 186 (142 to 244) | 24.1 (18.2 to 31.4) |
|  | Male | 2 (2 to 3) | 1 (0.7 to 1.3) | 2 (2 to 3) | 1.1 (0.8 to 1.4) | 62 (43 to 85) | 22 (15.6 to 29.6) | 1 (0 to 1) | 0.2 (0.1 to 0.3) | 63 (44 to 86) | 22.2 (15.7 to 29.9) | 4 (2 to 5) | 0.7 (0.5 to 1) | 4 (3 to 6) | 0.8 (0.5 to 1.1) | 88 (57 to 134) | 14.4 (9.7 to 21.4) | 1 (0 to 1) | 0.2 (0.1 to 0.3) | 89 (58 to 135) | 14.5 (9.8 to 21.6) |
| Brazil | Both | 2909 (2813 to 3002) | 3.4 (3.2 to 3.5) | 2984 (2871 to 3078) | 3.6 (3.4 to 3.7) | 69716 (67551 to 72096) | 73.1 (70.8 to 75.6) | 637 (436 to 842) | 0.7 (0.5 to 1) | 70353 (68216 to 72895) | 73.8 (71.6 to 76.3) | 4984 (4800 to 5125) | 2.2 (2.1 to 2.3) | 5203 (5020 to 5344) | 2.3 (2.3 to 2.4) | 111380 (107233 to 114508) | 48.1 (46.4 to 49.4) | 1106 (755 to 1462) | 0.5 (0.3 to 0.6) | 112486 (108307 to 115776) | 48.6 (46.9 to 50) |
|  | Female | 2070 (2005 to 2137) | 4.4 (4.3 to 4.6) | 2130 (2068 to 2195) | 4.7 (4.6 to 4.9) | 49145 (47585 to 50790) | 97.4 (94.5 to 100.6) | 449 (311 to 597) | 0.9 (0.7 to 1.3) | 49594 (48062 to 51286) | 98.4 (95.4 to 101.6) | 3397 (3267 to 3505) | 2.7 (2.6 to 2.8) | 3578 (3448 to 3687) | 2.9 (2.8 to 3) | 75056 (72149 to 77439) | 59.7 (57.4 to 61.6) | 747 (509 to 996) | 0.6 (0.4 to 0.8) | 75803 (72810 to 78268) | 60.3 (57.9 to 62.3) |
|  | Male | 839 (783 to 884) | 2.1 (1.9 to 2.2) | 854 (796 to 899) | 2.2 (2.1 to 2.4) | 20571 (19314 to 21835) | 45.5 (42.6 to 48.2) | 189 (128 to 252) | 0.5 (0.3 to 0.6) | 20759 (19490 to 22039) | 46 (43.1 to 48.8) | 1588 (1400 to 1661) | 1.6 (1.4 to 1.7) | 1626 (1428 to 1701) | 1.7 (1.5 to 1.7) | 36323 (31787 to 38108) | 34.3 (30.1 to 36) | 359 (244 to 479) | 0.4 (0.2 to 0.5) | 36682 (32075 to 38489) | 34.7 (30.3 to 36.4) |
| Brunei | Both | 4 (3 to 5) | 4 (3.3 to 5.4) | 4 (3 to 5) | 4 (3.3 to 5.4) | 84 (70 to 106) | 78.5 (64.8 to 101.3) | 1 (1 to 1) | 0.8 (0.6 to 1.2) | 85 (70 to 107) | 79.3 (65.5 to 102.5) | 10 (8 to 12) | 3.6 (2.9 to 4.4) | 9 (7 to 11) | 3.3 (2.8 to 3.9) | 216 (166 to 248) | 65.6 (52.8 to 76.7) | 2 (2 to 3) | 0.8 (0.5 to 1.1) | 218 (168 to 251) | 66.4 (53.4 to 77.9) |
|  | Female | 2 (1 to 2) | 4.1 (3.3 to 5.2) | 2 (1 to 2) | 4.2 (3.3 to 5.4) | 42 (31 to 52) | 82.9 (63 to 101.6) | 0 (0 to 1) | 0.9 (0.6 to 1.3) | 42 (31 to 52) | 83.8 (63.8 to 102.7) | 5 (4 to 6) | 3.4 (2.6 to 4.4) | 5 (3 to 5) | 3.1 (2.4 to 3.8) | 109 (76 to 131) | 64.3 (46.4 to 75.7) | 1 (1 to 2) | 0.8 (0.5 to 1.1) | 110 (77 to 132) | 65.1 (46.9 to 76.8) |
|  | Male | 2 (1 to 3) | 3.9 (2.9 to 5.7) | 2 (1 to 3) | 4 (3 to 5.8) | 42 (31 to 59) | 74.7 (54.6 to 106.9) | 0 (0 to 1) | 0.8 (0.5 to 1.3) | 43 (31 to 60) | 75.5 (55.4 to 108.4) | 5 (4 to 7) | 3.9 (2.9 to 5.2) | 4 (3 to 6) | 3.5 (2.6 to 4.5) | 106 (77 to 134) | 67.6 (49.1 to 86.1) | 1 (1 to 2) | 0.9 (0.5 to 1.3) | 108 (78 to 135) | 68.5 (49.7 to 86.9) |
| Bulgaria | Both | 201 (187 to 243) | 1.6 (1.5 to 1.9) | 201 (189 to 246) | 1.6 (1.5 to 2) | 4465 (4218 to 5166) | 34.2 (32.4 to 39.3) | 45 (30 to 61) | 0.4 (0.2 to 0.5) | 4510 (4260 to 5220) | 34.6 (32.8 to 39.8) | 243 (218 to 289) | 1.6 (1.5 to 1.9) | 249 (228 to 276) | 1.6 (1.5 to 1.8) | 4839 (4422 to 5305) | 34.9 (31.9 to 38.2) | 54 (36 to 72) | 0.4 (0.2 to 0.5) | 4893 (4468 to 5362) | 35.3 (32.2 to 38.6) |
|  | Female | 129 (118 to 150) | 1.8 (1.7 to 2.2) | 125 (116 to 147) | 1.8 (1.7 to 2.1) | 2754 (2575 to 2965) | 39.4 (36.9 to 42.6) | 29 (20 to 39) | 0.4 (0.3 to 0.6) | 2782 (2603 to 2994) | 39.8 (37.3 to 43.1) | 165 (144 to 205) | 1.9 (1.7 to 2.3) | 165 (148 to 187) | 1.9 (1.7 to 2.1) | 3116 (2761 to 3497) | 40.4 (35.8 to 45.3) | 36 (24 to 50) | 0.4 (0.3 to 0.6) | 3153 (2794 to 3542) | 40.9 (36.1 to 45.7) |
|  | Male | 72 (66 to 100) | 1.3 (1.2 to 1.8) | 76 (70 to 107) | 1.4 (1.3 to 1.9) | 1712 (1572 to 2276) | 28.5 (26.3 to 37.7) | 16 (11 to 23) | 0.3 (0.2 to 0.4) | 1728 (1585 to 2298) | 28.8 (26.6 to 38.1) | 77 (68 to 92) | 1.2 (1.1 to 1.4) | 84 (73 to 99) | 1.3 (1.2 to 1.6) | 1723 (1489 to 1980) | 28.4 (24.6 to 32.6) | 17 (12 to 24) | 0.3 (0.2 to 0.4) | 1740 (1504 to 2001) | 28.7 (24.9 to 32.9) |
| Burkina Faso | Both | 75 (60 to 99) | 1.8 (1.5 to 2.4) | 77 (62 to 102) | 1.9 (1.6 to 2.6) | 1765 (1411 to 2354) | 38.7 (31.1 to 51.4) | 17 (11 to 24) | 0.4 (0.3 to 0.6) | 1782 (1427 to 2368) | 39.1 (31.5 to 51.8) | 109 (89 to 146) | 1.4 (1.1 to 1.8) | 112 (92 to 149) | 1.5 (1.2 to 1.9) | 2620 (2123 to 3507) | 29.1 (23.7 to 38.8) | 24 (16 to 36) | 0.3 (0.2 to 0.4) | 2644 (2141 to 3537) | 29.4 (23.9 to 39.2) |
|  | Female | 47 (35 to 66) | 2.2 (1.6 to 3) | 48 (37 to 68) | 2.3 (1.8 to 3.2) | 1108 (830 to 1553) | 46.5 (34.9 to 65.1) | 10 (7 to 16) | 0.5 (0.3 to 0.7) | 1118 (838 to 1569) | 47 (35.2 to 65.8) | 67 (52 to 91) | 1.5 (1.2 to 2.1) | 69 (53 to 94) | 1.7 (1.3 to 2.2) | 1590 (1223 to 2195) | 32.8 (25.4 to 45.1) | 15 (9 to 22) | 0.3 (0.2 to 0.5) | 1605 (1234 to 2210) | 33.2 (25.6 to 45.5) |
|  | Male | 28 (18 to 39) | 1.4 (0.9 to 2) | 29 (19 to 40) | 1.5 (1 to 2.1) | 657 (422 to 919) | 30.3 (19.7 to 42.1) | 6 (4 to 10) | 0.3 (0.2 to 0.5) | 664 (427 to 927) | 30.6 (19.9 to 42.4) | 43 (31 to 65) | 1.1 (0.9 to 1.7) | 43 (32 to 66) | 1.2 (0.9 to 1.8) | 1030 (750 to 1577) | 24.6 (18.1 to 37.7) | 10 (6 to 15) | 0.3 (0.2 to 0.4) | 1039 (756 to 1594) | 24.9 (18.3 to 37.9) |
| Burundi | Both | 26 (19 to 37) | 1.3 (1 to 1.9) | 27 (20 to 38) | 1.5 (1.1 to 2.1) | 671 (492 to 964) | 29.7 (22.3 to 42) | 6 (4 to 9) | 0.3 (0.2 to 0.4) | 677 (497 to 973) | 30 (22.5 to 42.4) | 35 (27 to 47) | 1 (0.8 to 1.3) | 36 (28 to 49) | 1.1 (0.9 to 1.5) | 914 (705 to 1232) | 21.5 (16.8 to 29.1) | 8 (5 to 12) | 0.2 (0.1 to 0.3) | 922 (709 to 1241) | 21.7 (16.9 to 29.4) |
|  | Female | 16 (12 to 24) | 1.5 (1.1 to 2.2) | 17 (12 to 25) | 1.6 (1.2 to 2.4) | 415 (297 to 622) | 33.1 (24.2 to 49.4) | 4 (2 to 6) | 0.3 (0.2 to 0.5) | 418 (299 to 627) | 33.5 (24.5 to 49.9) | 20 (14 to 30) | 1.1 (0.8 to 1.8) | 21 (15 to 32) | 1.3 (0.9 to 2) | 517 (358 to 785) | 25.1 (17.5 to 38.1) | 5 (3 to 7) | 0.3 (0.2 to 0.4) | 522 (361 to 791) | 25.4 (17.7 to 38.5) |
|  | Male | 10 (5 to 15) | 1.1 (0.7 to 1.7) | 10 (6 to 16) | 1.2 (0.8 to 1.9) | 256 (142 to 410) | 25.3 (14.5 to 39.5) | 2 (1 to 4) | 0.2 (0.1 to 0.4) | 258 (143 to 413) | 25.6 (14.6 to 39.8) | 15 (10 to 20) | 0.8 (0.6 to 1.1) | 15 (11 to 21) | 0.9 (0.7 to 1.2) | 397 (276 to 550) | 17.9 (12.8 to 24.3) | 3 (2 to 5) | 0.2 (0.1 to 0.3) | 400 (279 to 555) | 18.1 (13 to 24.5) |
| Cambodia | Both | 99 (72 to 124) | 2.3 (1.7 to 2.8) | 101 (73 to 125) | 2.5 (1.8 to 3) | 2529 (1791 to 3203) | 52.2 (37.4 to 65.3) | 22 (14 to 31) | 0.5 (0.3 to 0.7) | 2551 (1805 to 3231) | 52.7 (37.9 to 65.9) | 193 (155 to 239) | 1.8 (1.5 to 2.3) | 199 (160 to 248) | 1.9 (1.6 to 2.4) | 4636 (3683 to 5724) | 39.6 (31.9 to 49.2) | 43 (28 to 60) | 0.4 (0.3 to 0.6) | 4679 (3728 to 5780) | 40 (32.1 to 49.7) |
|  | Female | 58 (38 to 76) | 2.3 (1.5 to 2.9) | 59 (39 to 77) | 2.5 (1.6 to 3.1) | 1464 (941 to 2005) | 52.8 (34.6 to 70.1) | 13 (7 to 19) | 0.5 (0.3 to 0.8) | 1477 (947 to 2022) | 53.3 (34.9 to 70.7) | 114 (84 to 150) | 1.8 (1.3 to 2.3) | 118 (87 to 155) | 1.9 (1.4 to 2.5) | 2690 (1968 to 3535) | 39.6 (28.9 to 51.8) | 25 (16 to 37) | 0.4 (0.3 to 0.6) | 2715 (1989 to 3577) | 40 (29.2 to 52.3) |
|  | Male | 42 (27 to 60) | 2.3 (1.5 to 3.2) | 43 (28 to 61) | 2.5 (1.7 to 3.5) | 1065 (683 to 1541) | 51.5 (33.5 to 73.9) | 9 (5 to 15) | 0.5 (0.3 to 0.8) | 1074 (689 to 1555) | 52 (33.9 to 74.6) | 79 (59 to 108) | 1.8 (1.4 to 2.5) | 81 (60 to 110) | 2 (1.5 to 2.8) | 1946 (1425 to 2692) | 39.8 (29.7 to 54) | 18 (11 to 27) | 0.4 (0.3 to 0.6) | 1964 (1438 to 2717) | 40.3 (30 to 54.6) |
| Cameroon | Both | 71 (58 to 94) | 1.7 (1.4 to 2.3) | 74 (61 to 98) | 1.9 (1.6 to 2.5) | 1729 (1397 to 2314) | 37.6 (30.7 to 49.8) | 16 (10 to 22) | 0.4 (0.3 to 0.5) | 1745 (1409 to 2338) | 37.9 (31 to 50.3) | 139 (104 to 184) | 1.4 (1 to 1.8) | 145 (109 to 191) | 1.5 (1.1 to 2) | 3366 (2502 to 4479) | 29 (21.8 to 38.4) | 31 (19 to 46) | 0.3 (0.2 to 0.4) | 3397 (2523 to 4523) | 29.3 (22 to 38.8) |
|  | Female | 42 (33 to 56) | 2 (1.6 to 2.7) | 43 (34 to 59) | 2.2 (1.7 to 2.9) | 1003 (777 to 1369) | 43 (33.9 to 57.9) | 9 (6 to 14) | 0.4 (0.3 to 0.6) | 1012 (784 to 1380) | 43.5 (34.2 to 58.5) | 80 (54 to 116) | 1.5 (1 to 2.2) | 84 (57 to 121) | 1.7 (1.2 to 2.4) | 1904 (1264 to 2803) | 32.3 (21.9 to 47.4) | 18 (10 to 28) | 0.3 (0.2 to 0.5) | 1922 (1277 to 2824) | 32.7 (22.1 to 47.9) |
|  | Male | 30 (20 to 40) | 1.5 (1 to 1.9) | 31 (21 to 41) | 1.6 (1.1 to 2.1) | 726 (502 to 984) | 31.9 (22 to 42.7) | 7 (4 to 10) | 0.3 (0.2 to 0.5) | 733 (507 to 991) | 32.2 (22.2 to 43.1) | 59 (39 to 89) | 1.2 (0.8 to 1.7) | 61 (41 to 91) | 1.3 (0.9 to 1.9) | 1462 (945 to 2221) | 25.5 (16.9 to 38.1) | 13 (8 to 21) | 0.3 (0.2 to 0.4) | 1475 (953 to 2240) | 25.7 (17.1 to 38.6) |
| Canada | Both | 1395 (1185 to 1765) | 4.2 (3.5 to 5.3) | 1056 (1018 to 1097) | 3.2 (3 to 3.3) | 19733 (18918 to 20446) | 59.8 (57.3 to 61.9) | 330 (220 to 465) | 1 (0.7 to 1.4) | 20063 (19232 to 20832) | 60.8 (58.1 to 63.1) | 1906 (1620 to 2472) | 2.8 (2.4 to 3.6) | 1483 (1379 to 1649) | 2.1 (1.9 to 2.3) | 24436 (22560 to 27440) | 37.8 (34.9 to 42.4) | 456 (301 to 640) | 0.7 (0.4 to 0.9) | 24892 (22957 to 27954) | 38.5 (35.5 to 43.2) |
|  | Female | 802 (664 to 1086) | 4.2 (3.5 to 5.6) | 633 (607 to 660) | 3.3 (3.1 to 3.4) | 11239 (10766 to 11725) | 62.1 (59.4 to 64.8) | 182 (116 to 275) | 1 (0.6 to 1.4) | 11421 (10936 to 11950) | 63.1 (60.3 to 66) | 1032 (835 to 1416) | 2.7 (2.2 to 3.6) | 865 (774 to 968) | 2.2 (1.9 to 2.4) | 13469 (11962 to 15228) | 39.1 (34.6 to 44) | 235 (152 to 350) | 0.6 (0.4 to 0.9) | 13704 (12205 to 15484) | 39.7 (35.3 to 44.8) |
|  | Male | 592 (476 to 798) | 4.1 (3.3 to 5.5) | 422 (399 to 456) | 3 (2.8 to 3.3) | 8494 (7699 to 8945) | 57.2 (52 to 60.2) | 148 (96 to 225) | 1 (0.6 to 1.5) | 8642 (7798 to 9109) | 58.2 (52.6 to 61.3) | 874 (677 to 1272) | 2.8 (2.2 to 4) | 618 (552 to 748) | 2 (1.8 to 2.4) | 10967 (9752 to 13334) | 36.3 (32.4 to 43.9) | 221 (141 to 340) | 0.7 (0.5 to 1.1) | 11188 (9960 to 13558) | 37.1 (33 to 44.7) |
| Cape Verde | Both | 2 (2 to 2) | 0.8 (0.7 to 1) | 2 (2 to 2) | 0.8 (0.7 to 1) | 40 (34 to 49) | 17.9 (15.1 to 21.7) | 0 (0 to 1) | 0.2 (0.1 to 0.2) | 40 (34 to 49) | 18.1 (15.3 to 21.9) | 4 (3 to 4) | 0.8 (0.7 to 1) | 4 (3 to 5) | 0.9 (0.7 to 1.1) | 77 (63 to 91) | 18 (14.7 to 21.3) | 1 (1 to 1) | 0.2 (0.1 to 0.3) | 77 (63 to 91) | 18.2 (14.8 to 21.5) |
|  | Female | 1 (1 to 2) | 0.9 (0.8 to 1.2) | 1 (1 to 2) | 1 (0.8 to 1.2) | 28 (23 to 35) | 21.8 (17.7 to 26.9) | 0 (0 to 0) | 0.2 (0.1 to 0.3) | 28 (23 to 35) | 22 (17.9 to 27.2) | 2 (2 to 3) | 1 (0.8 to 1.3) | 3 (2 to 3) | 1.1 (0.9 to 1.3) | 53 (41 to 64) | 22.5 (17.4 to 27.4) | 1 (0 to 1) | 0.2 (0.1 to 0.3) | 53 (41 to 65) | 22.7 (17.6 to 27.7) |
|  | Male | 1 (0 to 1) | 0.5 (0.4 to 0.7) | 1 (0 to 1) | 0.6 (0.5 to 0.8) | 12 (9 to 16) | 12.5 (9.8 to 16.7) | 0 (0 to 0) | 0.1 (0.1 to 0.2) | 12 (9 to 16) | 12.6 (9.9 to 16.9) | 1 (1 to 1) | 0.6 (0.4 to 0.8) | 1 (1 to 1) | 0.6 (0.5 to 0.8) | 24 (18 to 32) | 12.3 (9.4 to 16.5) | 0 (0 to 0) | 0.1 (0.1 to 0.2) | 24 (19 to 32) | 12.4 (9.5 to 16.7) |
| Caribbean | Both | 766 (604 to 820) | 2.9 (2.3 to 3.1) | 783 (617 to 833) | 3 (2.4 to 3.2) | 17020 (13685 to 18332) | 63 (50.6 to 67.8) | 169 (112 to 224) | 0.6 (0.4 to 0.9) | 17189 (13842 to 18538) | 63.6 (51.1 to 68.5) | 600 (521 to 856) | 1.2 (1 to 1.7) | 621 (541 to 881) | 1.2 (1.1 to 1.7) | 13266 (11380 to 19019) | 26 (22.3 to 37.3) | 134 (88 to 202) | 0.3 (0.2 to 0.4) | 13400 (11516 to 19174) | 26.3 (22.6 to 37.6) |
|  | Female | 461 (389 to 499) | 3.4 (2.9 to 3.7) | 469 (401 to 504) | 3.5 (2.9 to 3.7) | 10302 (8771 to 11260) | 74.1 (62.8 to 80.8) | 101 (66 to 135) | 0.7 (0.5 to 1) | 10403 (8849 to 11383) | 74.8 (63.4 to 81.7) | 354 (307 to 474) | 1.3 (1.1 to 1.7) | 368 (319 to 491) | 1.4 (1.2 to 1.8) | 7832 (6668 to 10554) | 29.3 (24.9 to 39.5) | 79 (52 to 115) | 0.3 (0.2 to 0.4) | 7910 (6736 to 10665) | 29.6 (25.2 to 39.9) |
|  | Male | 305 (204 to 337) | 2.4 (1.6 to 2.7) | 313 (209 to 343) | 2.5 (1.7 to 2.8) | 6718 (4606 to 7463) | 51.3 (35 to 56.9) | 68 (41 to 91) | 0.5 (0.3 to 0.7) | 6786 (4660 to 7537) | 51.9 (35.4 to 57.4) | 246 (202 to 376) | 1 (0.9 to 1.6) | 253 (208 to 383) | 1.1 (0.9 to 1.6) | 5434 (4400 to 8312) | 22.5 (18.3 to 34.4) | 55 (35 to 89) | 0.2 (0.1 to 0.4) | 5490 (4451 to 8400) | 22.8 (18.5 to 34.8) |
| Central African Republic | Both | 17 (12 to 22) | 1.6 (1.2 to 2) | 17 (12 to 22) | 1.7 (1.3 to 2.2) | 451 (305 to 620) | 36.7 (26.2 to 47.9) | 4 (2 to 6) | 0.4 (0.2 to 0.5) | 455 (308 to 624) | 37 (26.4 to 48.4) | 25 (18 to 34) | 1.3 (1 to 1.7) | 25 (18 to 34) | 1.5 (1.1 to 1.9) | 673 (460 to 943) | 30.6 (22.1 to 41) | 6 (3 to 8) | 0.3 (0.2 to 0.4) | 679 (465 to 950) | 30.9 (22.4 to 41.4) |
|  | Female | 11 (7 to 15) | 1.9 (1.4 to 2.5) | 11 (8 to 14) | 2.1 (1.6 to 2.7) | 284 (193 to 401) | 44.3 (31.1 to 59.4) | 2 (1 to 4) | 0.4 (0.3 to 0.6) | 287 (195 to 404) | 44.7 (31.5 to 59.9) | 16 (10 to 22) | 1.6 (1.1 to 2.2) | 16 (11 to 22) | 1.8 (1.2 to 2.4) | 412 (258 to 608) | 36.5 (23.9 to 50.8) | 3 (2 to 6) | 0.4 (0.2 to 0.5) | 415 (260 to 612) | 36.8 (24.1 to 51.2) |
|  | Male | 6 (3 to 9) | 1.2 (0.7 to 1.8) | 6 (3 to 9) | 1.3 (0.8 to 1.9) | 167 (90 to 265) | 28.1 (16 to 42.8) | 1 (1 to 2) | 0.3 (0.1 to 0.4) | 169 (91 to 267) | 28.4 (16.1 to 43.2) | 9 (5 to 15) | 1 (0.6 to 1.5) | 9 (5 to 15) | 1.1 (0.7 to 1.6) | 261 (141 to 432) | 23.6 (13.8 to 37) | 2 (1 to 4) | 0.2 (0.1 to 0.4) | 263 (142 to 436) | 23.8 (14 to 37.3) |
| Central Asia | Both | 543 (477 to 686) | 1.2 (1 to 1.5) | 562 (492 to 714) | 1.2 (1.1 to 1.6) | 13145 (11551 to 16276) | 26.6 (23.4 to 33.3) | 121 (80 to 173) | 0.3 (0.2 to 0.4) | 13266 (11644 to 16428) | 26.9 (23.6 to 33.6) | 756 (690 to 801) | 1.1 (1 to 1.1) | 786 (721 to 833) | 1.2 (1.1 to 1.2) | 18468 (16674 to 19641) | 24 (21.8 to 25.5) | 169 (115 to 225) | 0.2 (0.2 to 0.3) | 18637 (16809 to 19845) | 24.2 (22 to 25.7) |
|  | Female | 359 (313 to 497) | 1.3 (1.1 to 1.8) | 373 (326 to 520) | 1.3 (1.2 to 1.9) | 8448 (7331 to 11485) | 29.6 (25.7 to 40.3) | 80 (53 to 119) | 0.3 (0.2 to 0.4) | 8527 (7415 to 11602) | 29.9 (26 to 40.6) | 490 (430 to 528) | 1.2 (1.1 to 1.3) | 511 (451 to 551) | 1.3 (1.1 to 1.4) | 11794 (10223 to 12843) | 27.3 (23.8 to 29.6) | 109 (73 to 145) | 0.3 (0.2 to 0.4) | 11903 (10324 to 12948) | 27.6 (24.1 to 29.9) |
|  | Male | 184 (151 to 245) | 1 (0.8 to 1.3) | 189 (154 to 250) | 1.1 (0.9 to 1.4) | 4697 (3866 to 6248) | 22.8 (18.6 to 30.2) | 41 (26 to 61) | 0.2 (0.1 to 0.3) | 4738 (3901 to 6300) | 23 (18.8 to 30.5) | 266 (243 to 302) | 0.9 (0.8 to 1) | 275 (251 to 313) | 1 (0.9 to 1.1) | 6675 (6049 to 7570) | 20 (18.2 to 22.6) | 60 (42 to 80) | 0.2 (0.1 to 0.3) | 6734 (6101 to 7616) | 20.2 (18.4 to 22.8) |
| Central Europe | Both | 6797 (6533 to 7119) | 4.5 (4.4 to 4.8) | 6917 (6671 to 7187) | 4.6 (4.5 to 4.8) | 141312 (135893 to 146251) | 91.8 (88.4 to 95.1) | 1430 (986 to 1888) | 0.9 (0.7 to 1.3) | 142742 (137349 to 147542) | 92.7 (89.4 to 96) | 6834 (6449 to 7283) | 3.1 (2.9 to 3.3) | 6709 (6421 to 7152) | 3 (2.9 to 3.2) | 122213 (116847 to 130010) | 58.4 (55.8 to 62) | 1491 (1034 to 1958) | 0.7 (0.5 to 0.9) | 123704 (118345 to 131431) | 59.1 (56.4 to 62.8) |
|  | Female | 5024 (4820 to 5251) | 5.7 (5.5 to 6) | 5105 (4941 to 5253) | 5.8 (5.6 to 6) | 101164 (97442 to 104124) | 115.8 (111.5 to 119.1) | 1041 (711 to 1383) | 1.2 (0.8 to 1.6) | 102205 (98409 to 105277) | 117 (112.7 to 120.5) | 4634 (4313 to 5070) | 3.6 (3.4 to 3.9) | 4510 (4266 to 4918) | 3.4 (3.3 to 3.8) | 78796 (74354 to 86652) | 67 (63.3 to 73.6) | 1000 (699 to 1329) | 0.8 (0.5 to 1) | 79797 (75428 to 87524) | 67.8 (64.1 to 74.4) |
|  | Male | 1773 (1640 to 1978) | 2.8 (2.6 to 3.2) | 1812 (1689 to 2018) | 3 (2.8 to 3.3) | 40148 (37177 to 44151) | 60.1 (55.7 to 66.2) | 389 (266 to 514) | 0.6 (0.4 to 0.8) | 40537 (37546 to 44571) | 60.7 (56.3 to 66.9) | 2200 (1866 to 2396) | 2.4 (2.1 to 2.7) | 2199 (1880 to 2354) | 2.4 (2.1 to 2.6) | 43417 (37297 to 46693) | 47.7 (40.9 to 51.2) | 490 (334 to 655) | 0.5 (0.4 to 0.7) | 43907 (37741 to 47263) | 48.2 (41.4 to 51.9) |
| Central Latin America | Both | 3414 (3222 to 3520) | 4.1 (3.9 to 4.2) | 3515 (3302 to 3624) | 4.3 (4.1 to 4.5) | 81481 (76526 to 84126) | 90.8 (85.2 to 93.6) | 741 (508 to 978) | 0.9 (0.6 to 1.2) | 82222 (77361 to 84907) | 91.6 (86.2 to 94.6) | 4872 (4610 to 5852) | 2.1 (2 to 2.6) | 5068 (4798 to 6052) | 2.2 (2.1 to 2.7) | 108657 (102973 to 127896) | 46 (43.5 to 54.2) | 1085 (738 to 1479) | 0.5 (0.3 to 0.6) | 109743 (103955 to 129452) | 46.4 (44 to 54.8) |
|  | Female | 2502 (2421 to 2590) | 5.7 (5.5 to 5.9) | 2568 (2486 to 2659) | 6 (5.8 to 6.2) | 60022 (57936 to 62186) | 129 (124.6 to 133.6) | 538 (370 to 711) | 1.2 (0.8 to 1.6) | 60560 (58492 to 62741) | 130.2 (125.9 to 134.9) | 3389 (3176 to 3867) | 2.7 (2.6 to 3.1) | 3531 (3313 to 4058) | 2.9 (2.7 to 3.3) | 75942 (71240 to 86722) | 60 (56.3 to 68.6) | 751 (509 to 1027) | 0.6 (0.4 to 0.8) | 76693 (71906 to 87530) | 60.6 (56.8 to 69.2) |
|  | Male | 912 (779 to 945) | 2.3 (2 to 2.4) | 946 (803 to 978) | 2.5 (2.1 to 2.6) | 21459 (18239 to 22205) | 49.8 (42.2 to 51.5) | 204 (138 to 272) | 0.5 (0.3 to 0.7) | 21663 (18383 to 22424) | 50.3 (42.6 to 52.1) | 1483 (1373 to 1923) | 1.4 (1.3 to 1.8) | 1537 (1423 to 2004) | 1.5 (1.4 to 2) | 32715 (30278 to 41513) | 29.9 (27.6 to 38.1) | 335 (224 to 459) | 0.3 (0.2 to 0.4) | 33050 (30583 to 42015) | 30.2 (27.9 to 38.6) |
| Central Sub-Saharan Africa | Both | 290 (223 to 358) | 1.4 (1.1 to 1.7) | 296 (229 to 367) | 1.5 (1.2 to 1.9) | 7482 (5679 to 9399) | 31.1 (24.1 to 38.7) | 64 (43 to 89) | 0.3 (0.2 to 0.4) | 7547 (5732 to 9477) | 31.4 (24.4 to 39.1) | 548 (451 to 680) | 1.2 (1 to 1.5) | 566 (467 to 705) | 1.3 (1.1 to 1.7) | 14020 (11528 to 17272) | 26.9 (22.2 to 33.5) | 122 (79 to 172) | 0.3 (0.2 to 0.4) | 14143 (11618 to 17415) | 27.2 (22.4 to 33.8) |
|  | Female | 188 (137 to 244) | 1.7 (1.3 to 2.2) | 192 (141 to 247) | 1.9 (1.4 to 2.4) | 4864 (3490 to 6455) | 38.5 (28.3 to 49.2) | 42 (26 to 60) | 0.4 (0.2 to 0.5) | 4906 (3522 to 6512) | 38.9 (28.6 to 49.8) | 362 (279 to 485) | 1.5 (1.2 to 2) | 377 (292 to 505) | 1.6 (1.3 to 2.2) | 9102 (6845 to 12185) | 32.3 (24.9 to 43.3) | 81 (50 to 117) | 0.3 (0.2 to 0.5) | 9183 (6906 to 12288) | 32.7 (25.2 to 43.7) |
|  | Male | 101 (70 to 137) | 1 (0.7 to 1.4) | 104 (72 to 140) | 1.1 (0.8 to 1.5) | 2619 (1799 to 3611) | 23 (16.1 to 30.9) | 23 (14 to 33) | 0.2 (0.1 to 0.3) | 2641 (1817 to 3641) | 23.3 (16.2 to 31.3) | 186 (137 to 253) | 0.9 (0.7 to 1.2) | 189 (141 to 255) | 1 (0.8 to 1.3) | 4918 (3593 to 6816) | 20.3 (15.1 to 27.3) | 42 (26 to 61) | 0.2 (0.1 to 0.3) | 4960 (3613 to 6874) | 20.5 (15.2 to 27.5) |
| Chad | Both | 42 (35 to 54) | 1.5 (1.3 to 1.9) | 45 (37 to 57) | 1.6 (1.4 to 2.1) | 952 (788 to 1263) | 32.6 (27.2 to 42.9) | 9 (6 to 13) | 0.3 (0.2 to 0.5) | 962 (794 to 1275) | 32.9 (27.4 to 43.3) | 68 (53 to 91) | 1.4 (1.1 to 1.8) | 71 (56 to 95) | 1.5 (1.2 to 2) | 1619 (1265 to 2203) | 29.5 (23.1 to 39.8) | 15 (10 to 22) | 0.3 (0.2 to 0.4) | 1634 (1277 to 2218) | 29.8 (23.4 to 40.1) |
|  | Female | 25 (20 to 34) | 1.7 (1.4 to 2.4) | 26 (21 to 36) | 1.9 (1.5 to 2.6) | 563 (445 to 786) | 37.6 (29.8 to 52.6) | 5 (3 to 8) | 0.4 (0.2 to 0.6) | 568 (450 to 794) | 38 (30.1 to 53.2) | 37 (27 to 51) | 1.7 (1.2 to 2.3) | 39 (29 to 54) | 1.8 (1.4 to 2.5) | 890 (643 to 1245) | 35.8 (26.1 to 49.3) | 8 (5 to 12) | 0.4 (0.2 to 0.5) | 898 (649 to 1256) | 36.2 (26.4 to 49.8) |
|  | Male | 17 (12 to 23) | 1.3 (0.9 to 1.7) | 18 (13 to 24) | 1.4 (1 to 1.8) | 390 (280 to 524) | 27.3 (19.7 to 36.6) | 4 (2 to 6) | 0.3 (0.2 to 0.4) | 393 (282 to 529) | 27.6 (19.9 to 36.9) | 30 (23 to 46) | 1.1 (0.8 to 1.7) | 32 (24 to 48) | 1.2 (0.9 to 1.8) | 730 (539 to 1133) | 24.3 (18.3 to 37.2) | 7 (4 to 11) | 0.2 (0.1 to 0.4) | 737 (546 to 1141) | 24.5 (18.4 to 37.5) |
| Chile | Both | 1817 (1667 to 1925) | 18 (16.5 to 19.1) | 1827 (1692 to 1923) | 18.4 (17.1 to 19.4) | 41338 (37759 to 43603) | 391.8 (357.9 to 413.1) | 368 (254 to 493) | 3.6 (2.5 to 4.8) | 41706 (38138 to 43976) | 395.4 (361.6 to 416.9) | 2525 (2207 to 2932) | 10.8 (9.5 to 12.6) | 2435 (2139 to 2792) | 10.4 (9.2 to 12) | 48577 (42355 to 55685) | 208.7 (181.9 to 239) | 532 (356 to 728) | 2.3 (1.5 to 3.1) | 49109 (42777 to 56336) | 211 (183.8 to 241.9) |
|  | Female | 1350 (1242 to 1447) | 24.2 (22.2 to 25.9) | 1359 (1259 to 1437) | 24.6 (22.7 to 25.9) | 31104 (28583 to 32839) | 542.1 (499.2 to 572.6) | 271 (182 to 367) | 4.8 (3.2 to 6.5) | 31375 (28884 to 33153) | 546.9 (503.6 to 577.7) | 1732 (1459 to 2073) | 13.5 (11.3 to 16.1) | 1702 (1453 to 1998) | 13.1 (11.1 to 15.3) | 33954 (28691 to 40284) | 270.4 (227.7 to 321.1) | 358 (235 to 503) | 2.8 (1.8 to 4) | 34312 (28980 to 40723) | 273.2 (230.1 to 324.8) |
|  | Male | 466 (349 to 523) | 10.5 (7.9 to 11.8) | 469 (350 to 515) | 10.9 (8.2 to 11.9) | 10234 (7776 to 11388) | 216.6 (163.5 to 240.4) | 97 (64 to 136) | 2.2 (1.4 to 3) | 10331 (7861 to 11495) | 218.8 (165.2 to 242.7) | 792 (647 to 1006) | 7.7 (6.3 to 9.7) | 733 (613 to 884) | 7.2 (6 to 8.7) | 14623 (12093 to 17835) | 137.7 (114.1 to 167.8) | 174 (111 to 246) | 1.7 (1.1 to 2.3) | 14797 (12257 to 18025) | 139.4 (115.6 to 169.8) |
| China | Both | 11360 (10098 to 16866) | 1.4 (1.2 to 2.1) | 11447 (10128 to 17251) | 1.5 (1.3 to 2.2) | 281273 (251132 to 410073) | 30.6 (27.2 to 45) | 2548 (1673 to 3966) | 0.3 (0.2 to 0.5) | 283821 (253335 to 414562) | 31 (27.5 to 45.5) | 32297 (23370 to 35602) | 1.7 (1.2 to 1.9) | 27967 (21031 to 30625) | 1.5 (1.1 to 1.7) | 574892 (436720 to 629598) | 29.1 (22.1 to 31.9) | 7589 (4804 to 10281) | 0.4 (0.3 to 0.5) | 582481 (442334 to 637348) | 29.5 (22.4 to 32.3) |
|  | Female | 6136 (5393 to 9297) | 1.5 (1.3 to 2.2) | 6229 (5449 to 9655) | 1.5 (1.3 to 2.4) | 147784 (130829 to 214808) | 31.9 (28.1 to 47.4) | 1370 (901 to 2124) | 0.3 (0.2 to 0.5) | 149154 (131974 to 217211) | 32.2 (28.4 to 47.9) | 17356 (11502 to 19676) | 1.8 (1.2 to 2) | 14866 (10067 to 16377) | 1.5 (1 to 1.7) | 297865 (206038 to 328543) | 29.6 (20.6 to 32.6) | 4031 (2457 to 5553) | 0.4 (0.3 to 0.6) | 301896 (208853 to 332944) | 30 (20.8 to 33) |
|  | Male | 5224 (4408 to 8597) | 1.4 (1.2 to 2.2) | 5217 (4415 to 8656) | 1.4 (1.2 to 2.3) | 133489 (112776 to 216057) | 29.5 (24.9 to 48.2) | 1178 (756 to 1918) | 0.3 (0.2 to 0.5) | 134667 (113768 to 217562) | 29.8 (25.2 to 48.9) | 14941 (10306 to 17745) | 1.7 (1.2 to 2) | 13101 (9260 to 15863) | 1.5 (1.1 to 1.8) | 277027 (196123 to 338548) | 28.7 (20.4 to 34.9) | 3558 (2202 to 4962) | 0.4 (0.2 to 0.6) | 280585 (198291 to 342462) | 29.1 (20.6 to 35.3) |
| Colombia | Both | 771 (735 to 820) | 4.4 (4.2 to 4.7) | 786 (752 to 827) | 4.6 (4.4 to 4.9) | 18674 (17810 to 19622) | 98.7 (94.1 to 103.8) | 168 (115 to 224) | 0.9 (0.7 to 1.3) | 18841 (17966 to 19798) | 99.6 (95 to 104.8) | 1183 (1040 to 1454) | 2.2 (1.9 to 2.7) | 1245 (1096 to 1529) | 2.3 (2 to 2.8) | 25439 (22284 to 31189) | 47.1 (41.3 to 57.8) | 264 (177 to 368) | 0.5 (0.3 to 0.7) | 25703 (22504 to 31505) | 47.6 (41.7 to 58.4) |
|  | Female | 584 (550 to 617) | 6.5 (6.1 to 6.8) | 595 (563 to 624) | 6.8 (6.4 to 7.1) | 14138 (13347 to 14873) | 146 (138 to 153.5) | 126 (86 to 170) | 1.4 (0.9 to 1.9) | 14264 (13462 to 15000) | 147.4 (139.3 to 154.8) | 824 (704 to 1000) | 2.8 (2.4 to 3.4) | 869 (742 to 1046) | 2.9 (2.5 to 3.5) | 17656 (15072 to 21553) | 60.5 (51.6 to 73.9) | 183 (122 to 263) | 0.6 (0.4 to 0.9) | 17839 (15232 to 21799) | 61.2 (52.2 to 74.7) |
|  | Male | 186 (173 to 228) | 2.2 (2.1 to 2.7) | 192 (179 to 225) | 2.4 (2.2 to 2.8) | 4536 (4219 to 5249) | 48.9 (45.7 to 56.7) | 42 (29 to 58) | 0.5 (0.3 to 0.7) | 4578 (4266 to 5304) | 49.4 (46.1 to 57.3) | 359 (289 to 537) | 1.5 (1.2 to 2.2) | 377 (306 to 566) | 1.6 (1.3 to 2.3) | 7783 (6238 to 11480) | 31.4 (25.2 to 46.4) | 81 (51 to 119) | 0.3 (0.2 to 0.5) | 7863 (6311 to 11587) | 31.7 (25.5 to 46.9) |
| Comoros | Both | 2 (2 to 3) | 1.3 (0.9 to 1.8) | 2 (2 to 4) | 1.4 (1 to 1.9) | 62 (45 to 88) | 28.7 (21.2 to 40.9) | 1 (0 to 1) | 0.3 (0.2 to 0.4) | 62 (46 to 89) | 28.9 (21.4 to 41.3) | 4 (3 to 6) | 1.1 (0.8 to 1.5) | 5 (4 to 7) | 1.2 (0.9 to 1.7) | 109 (85 to 155) | 23.5 (18.3 to 33.5) | 1 (1 to 2) | 0.2 (0.1 to 0.4) | 110 (85 to 156) | 23.7 (18.5 to 33.8) |
|  | Female | 1 (1 to 2) | 1.5 (1 to 2.5) | 2 (1 to 2) | 1.7 (1.1 to 2.7) | 38 (25 to 61) | 34.9 (22.9 to 56) | 0 (0 to 1) | 0.3 (0.2 to 0.6) | 38 (25 to 62) | 35.2 (23.1 to 56.6) | 3 (2 to 5) | 1.2 (0.9 to 2) | 3 (2 to 5) | 1.4 (1 to 2.1) | 69 (51 to 110) | 27.6 (20.2 to 43.8) | 1 (0 to 1) | 0.3 (0.2 to 0.5) | 70 (51 to 111) | 27.9 (20.5 to 44.3) |
|  | Male | 1 (1 to 1) | 1 (0.7 to 1.4) | 1 (1 to 1) | 1.1 (0.8 to 1.5) | 24 (16 to 34) | 22.3 (15.5 to 31.6) | 0 (0 to 0) | 0.2 (0.1 to 0.3) | 24 (16 to 34) | 22.5 (15.7 to 31.9) | 2 (1 to 2) | 0.8 (0.6 to 1.2) | 2 (1 to 2) | 0.9 (0.6 to 1.3) | 39 (25 to 56) | 18.6 (12.1 to 26.5) | 0 (0 to 1) | 0.2 (0.1 to 0.3) | 40 (26 to 57) | 18.8 (12.3 to 26.8) |
| Congo | Both | 18 (13 to 23) | 1.8 (1.3 to 2.3) | 18 (13 to 24) | 1.9 (1.4 to 2.4) | 447 (310 to 615) | 39.5 (28.1 to 52.6) | 4 (2 to 6) | 0.4 (0.2 to 0.6) | 451 (313 to 620) | 39.8 (28.4 to 53) | 31 (24 to 40) | 1.4 (1.1 to 1.8) | 32 (25 to 41) | 1.6 (1.3 to 2) | 792 (597 to 1075) | 31.3 (24.1 to 40.4) | 7 (4 to 10) | 0.3 (0.2 to 0.5) | 799 (601 to 1085) | 31.6 (24.3 to 40.7) |
|  | Female | 12 (8 to 17) | 2.2 (1.5 to 2.9) | 13 (9 to 18) | 2.4 (1.7 to 3.1) | 309 (200 to 453) | 49.3 (32.8 to 69.3) | 3 (2 to 4) | 0.5 (0.3 to 0.7) | 312 (202 to 457) | 49.7 (33.2 to 69.9) | 21 (15 to 30) | 1.9 (1.3 to 2.5) | 22 (15 to 31) | 2 (1.5 to 2.7) | 535 (354 to 819) | 41 (28.3 to 58.8) | 5 (3 to 7) | 0.4 (0.2 to 0.6) | 540 (357 to 825) | 41.4 (28.6 to 59.3) |
|  | Male | 5 (3 to 8) | 1.2 (0.8 to 1.6) | 5 (3 to 8) | 1.3 (0.8 to 1.7) | 138 (82 to 206) | 26.9 (16.6 to 38.4) | 1 (1 to 2) | 0.3 (0.2 to 0.4) | 139 (83 to 208) | 27.1 (16.8 to 38.8) | 10 (7 to 13) | 0.9 (0.7 to 1.3) | 10 (7 to 14) | 1 (0.7 to 1.4) | 257 (176 to 350) | 20.7 (14.4 to 27.9) | 2 (1 to 3) | 0.2 (0.1 to 0.3) | 259 (178 to 353) | 20.9 (14.5 to 28.1) |
| Costa Rica | Both | 80 (74 to 85) | 4.6 (4.3 to 4.9) | 81 (75 to 86) | 4.8 (4.4 to 5) | 1719 (1580 to 1816) | 94.3 (86.9 to 99.6) | 17 (11 to 23) | 1 (0.7 to 1.3) | 1737 (1594 to 1834) | 95.3 (87.7 to 100.7) | 105 (93 to 147) | 2.2 (1.9 to 3) | 109 (96 to 150) | 2.3 (2 to 3.1) | 2183 (1934 to 3005) | 44.2 (39.2 to 60.8) | 23 (15 to 33) | 0.5 (0.3 to 0.7) | 2207 (1956 to 3036) | 44.7 (39.6 to 61.5) |
|  | Female | 55 (50 to 59) | 6.1 (5.6 to 6.6) | 56 (52 to 59) | 6.3 (5.8 to 6.7) | 1174 (1091 to 1255) | 125.8 (116.7 to 134.4) | 12 (8 to 16) | 1.3 (0.9 to 1.7) | 1186 (1104 to 1266) | 127.1 (117.9 to 135.6) | 69 (59 to 89) | 2.6 (2.2 to 3.4) | 72 (62 to 92) | 2.7 (2.4 to 3.5) | 1419 (1227 to 1861) | 53.7 (46.3 to 70.3) | 15 (10 to 21) | 0.6 (0.4 to 0.8) | 1434 (1238 to 1881) | 54.2 (46.7 to 71) |
|  | Male | 25 (21 to 28) | 3 (2.5 to 3.3) | 26 (21 to 28) | 3.2 (2.7 to 3.5) | 545 (454 to 593) | 61.2 (51.1 to 66.6) | 6 (4 to 8) | 0.7 (0.4 to 0.9) | 551 (459 to 599) | 61.8 (51.6 to 67.4) | 37 (30 to 58) | 1.6 (1.4 to 2.6) | 37 (31 to 60) | 1.7 (1.4 to 2.7) | 764 (638 to 1172) | 33.2 (27.8 to 51.4) | 8 (5 to 13) | 0.4 (0.2 to 0.6) | 773 (645 to 1185) | 33.6 (28 to 52.2) |
| Cote d'Ivoire | Both | 57 (45 to 72) | 1.6 (1.3 to 2) | 57 (46 to 73) | 1.7 (1.4 to 2.1) | 1427 (1126 to 1838) | 33.7 (27.1 to 43) | 13 (8 to 18) | 0.3 (0.2 to 0.5) | 1439 (1136 to 1853) | 34.1 (27.3 to 43.4) | 122 (95 to 167) | 1.4 (1.1 to 1.9) | 125 (98 to 170) | 1.5 (1.2 to 2) | 3009 (2329 to 4150) | 29.1 (22.7 to 39.9) | 27 (17 to 40) | 0.3 (0.2 to 0.4) | 3036 (2355 to 4179) | 29.4 (23 to 40.2) |
|  | Female | 29 (21 to 41) | 1.8 (1.3 to 2.4) | 30 (22 to 42) | 2 (1.5 to 2.7) | 724 (536 to 1053) | 38 (28.4 to 53.9) | 6 (4 to 10) | 0.4 (0.2 to 0.6) | 730 (541 to 1062) | 38.4 (28.7 to 54.3) | 63 (44 to 93) | 1.5 (1.1 to 2.2) | 66 (47 to 98) | 1.7 (1.2 to 2.4) | 1548 (1086 to 2309) | 32.1 (22.5 to 47.4) | 14 (8 to 22) | 0.3 (0.2 to 0.5) | 1562 (1094 to 2330) | 32.4 (22.7 to 47.9) |
|  | Male | 28 (20 to 38) | 1.4 (1 to 1.9) | 27 (19 to 37) | 1.5 (1.1 to 2) | 703 (496 to 951) | 30.1 (21.4 to 40.6) | 6 (4 to 9) | 0.3 (0.2 to 0.5) | 709 (501 to 959) | 30.4 (21.6 to 41) | 58 (44 to 86) | 1.2 (0.9 to 1.8) | 58 (44 to 85) | 1.3 (1 to 1.9) | 1461 (1101 to 2136) | 26.3 (19.8 to 38.4) | 13 (8 to 21) | 0.3 (0.2 to 0.4) | 1474 (1111 to 2153) | 26.5 (20 to 38.8) |
| Croatia | Both | 291 (257 to 361) | 4.6 (4 to 5.8) | 262 (249 to 280) | 4.1 (3.9 to 4.4) | 5148 (4876 to 5526) | 77.7 (73.7 to 83.2) | 62 (42 to 87) | 1 (0.7 to 1.4) | 5210 (4934 to 5587) | 78.7 (74.7 to 84.1) | 348 (285 to 457) | 3.7 (3.1 to 4.8) | 279 (246 to 303) | 3 (2.7 to 3.2) | 4665 (4116 to 5065) | 53.9 (47.8 to 58.5) | 77 (50 to 110) | 0.8 (0.5 to 1.2) | 4742 (4186 to 5148) | 54.7 (48.4 to 59.4) |
|  | Female | 222 (191 to 283) | 5.7 (4.8 to 7.4) | 192 (180 to 205) | 4.9 (4.6 to 5.2) | 3643 (3415 to 3896) | 93 (87.5 to 99.1) | 47 (31 to 67) | 1.2 (0.8 to 1.7) | 3689 (3464 to 3944) | 94.2 (88.6 to 100.3) | 241 (182 to 348) | 4.3 (3.3 to 5.9) | 176 (157 to 196) | 3.1 (2.8 to 3.5) | 2809 (2496 to 3136) | 57.1 (50.4 to 63.8) | 54 (34 to 83) | 1 (0.6 to 1.4) | 2862 (2543 to 3193) | 58.1 (51.4 to 64.7) |
|  | Male | 69 (63 to 82) | 2.8 (2.5 to 3.3) | 69 (64 to 82) | 2.8 (2.6 to 3.4) | 1506 (1389 to 1787) | 55.1 (50.8 to 65) | 15 (11 to 21) | 0.6 (0.4 to 0.8) | 1521 (1402 to 1803) | 55.7 (51.4 to 65.6) | 107 (75 to 132) | 2.9 (2 to 3.5) | 102 (71 to 115) | 2.8 (1.9 to 3.1) | 1856 (1342 to 2094) | 49.9 (36.6 to 56.3) | 24 (14 to 35) | 0.6 (0.4 to 0.9) | 1880 (1364 to 2124) | 50.6 (36.9 to 57) |
| Cuba | Both | 369 (252 to 395) | 3.5 (2.4 to 3.8) | 376 (261 to 397) | 3.6 (2.5 to 3.8) | 7701 (5393 to 8136) | 73.3 (51.3 to 77.4) | 80 (50 to 109) | 0.8 (0.5 to 1) | 7781 (5448 to 8223) | 74.1 (51.8 to 78.3) | 199 (172 to 277) | 1.1 (0.9 to 1.5) | 206 (179 to 286) | 1.1 (0.9 to 1.5) | 3995 (3457 to 5592) | 21.8 (18.9 to 30.6) | 44 (30 to 65) | 0.2 (0.2 to 0.3) | 4039 (3491 to 5650) | 22.1 (19.1 to 31) |
|  | Female | 225 (160 to 246) | 4.2 (3 to 4.6) | 227 (164 to 242) | 4.3 (3.1 to 4.5) | 4764 (3512 to 5074) | 89.6 (66.2 to 95.4) | 49 (30 to 67) | 0.9 (0.6 to 1.3) | 4813 (3564 to 5128) | 90.5 (67.2 to 96.4) | 121 (101 to 163) | 1.2 (1 to 1.6) | 127 (106 to 170) | 1.3 (1 to 1.7) | 2447 (2025 to 3339) | 25.5 (21.1 to 34.9) | 27 (18 to 39) | 0.3 (0.2 to 0.4) | 2474 (2044 to 3373) | 25.8 (21.3 to 35.3) |
|  | Male | 144 (78 to 159) | 2.8 (1.5 to 3.1) | 148 (81 to 161) | 2.9 (1.6 to 3.2) | 2937 (1647 to 3206) | 56.7 (31.7 to 61.9) | 32 (17 to 44) | 0.6 (0.3 to 0.9) | 2969 (1666 to 3244) | 57.3 (32.2 to 62.7) | 77 (61 to 117) | 0.9 (0.7 to 1.3) | 79 (64 to 119) | 0.9 (0.7 to 1.4) | 1548 (1239 to 2318) | 17.8 (14.3 to 26.8) | 17 (11 to 26) | 0.2 (0.1 to 0.3) | 1566 (1252 to 2342) | 18 (14.4 to 27.1) |
| Cyprus | Both | 24 (18 to 29) | 2.8 (2.1 to 3.4) | 23 (17 to 26) | 2.7 (2 to 3.1) | 428 (326 to 496) | 49.5 (37.9 to 57.6) | 6 (4 to 8) | 0.7 (0.4 to 0.9) | 433 (331 to 504) | 50.2 (38.5 to 58.4) | 38 (30 to 48) | 2 (1.5 to 2.5) | 32 (24 to 37) | 1.6 (1.3 to 1.9) | 556 (446 to 659) | 29.4 (23.8 to 35.1) | 9 (6 to 13) | 0.5 (0.3 to 0.7) | 565 (452 to 670) | 29.9 (24.1 to 35.7) |
|  | Female | 13 (9 to 16) | 2.9 (2 to 3.5) | 13 (9 to 15) | 2.8 (1.9 to 3.3) | 244 (169 to 288) | 52.8 (37.1 to 62.2) | 3 (2 to 4) | 0.7 (0.4 to 0.9) | 247 (172 to 292) | 53.4 (37.6 to 62.8) | 16 (13 to 22) | 1.6 (1.3 to 2.1) | 15 (12 to 18) | 1.4 (1.1 to 1.7) | 253 (207 to 330) | 25.6 (20.9 to 33.7) | 4 (2 to 6) | 0.4 (0.2 to 0.6) | 256 (210 to 335) | 26 (21.2 to 34.2) |
|  | Male | 11 (7 to 14) | 2.8 (1.7 to 3.7) | 10 (6 to 12) | 2.6 (1.6 to 3.2) | 184 (117 to 233) | 46.1 (29.3 to 58.4) | 3 (1 to 4) | 0.7 (0.4 to 1) | 186 (118 to 236) | 46.7 (29.8 to 59.2) | 21 (14 to 30) | 2.4 (1.6 to 3.4) | 17 (11 to 21) | 1.9 (1.3 to 2.4) | 303 (203 to 384) | 33.8 (22.6 to 42.4) | 5 (3 to 8) | 0.6 (0.4 to 0.9) | 309 (206 to 391) | 34.4 (23 to 43.2) |
| Czech Republic | Both | 1207 (1073 to 1361) | 8.5 (7.6 to 9.7) | 1178 (1063 to 1223) | 8.3 (7.5 to 8.6) | 22409 (20242 to 23302) | 157.9 (143.2 to 164.2) | 240 (161 to 334) | 1.7 (1.1 to 2.3) | 22649 (20435 to 23539) | 159.6 (144.7 to 165.9) | 1117 (966 to 1360) | 5.2 (4.5 to 6.4) | 916 (838 to 993) | 4.2 (3.9 to 4.6) | 15947 (14591 to 17357) | 77.7 (70.9 to 84.8) | 243 (162 to 328) | 1.2 (0.8 to 1.6) | 16190 (14795 to 17623) | 78.8 (72 to 86.2) |
|  | Female | 879 (795 to 1024) | 10.2 (9.3 to 11.8) | 855 (811 to 890) | 9.8 (9.3 to 10.2) | 15742 (14947 to 16377) | 190.2 (179.7 to 198.2) | 171 (112 to 241) | 2 (1.3 to 2.8) | 15913 (15118 to 16571) | 192.2 (181.7 to 200.3) | 715 (584 to 945) | 5.8 (4.8 to 7.5) | 591 (532 to 656) | 4.7 (4.2 to 5.2) | 9919 (8857 to 11070) | 87.6 (78.3 to 97.9) | 152 (99 to 218) | 1.3 (0.8 to 1.8) | 10071 (8978 to 11243) | 88.8 (79.4 to 99.3) |
|  | Male | 328 (235 to 367) | 5.9 (4.3 to 6.7) | 323 (232 to 347) | 5.9 (4.3 to 6.3) | 6667 (4902 to 7198) | 113.6 (84.1 to 122.1) | 69 (43 to 96) | 1.2 (0.8 to 1.7) | 6736 (4957 to 7275) | 114.8 (85 to 123.5) | 401 (318 to 510) | 4.6 (3.6 to 5.9) | 326 (267 to 366) | 3.6 (3 to 4.1) | 6028 (5042 to 6880) | 66.2 (55.7 to 75.5) | 91 (57 to 130) | 1 (0.6 to 1.5) | 6119 (5097 to 6985) | 67.2 (56.4 to 76.7) |
| Democratic Republic of the Congo | Both | 187 (146 to 241) | 1.3 (1 to 1.6) | 191 (150 to 245) | 1.4 (1.1 to 1.8) | 4800 (3679 to 6170) | 28.7 (22.5 to 36.7) | 42 (27 to 59) | 0.3 (0.2 to 0.4) | 4842 (3711 to 6227) | 29 (22.8 to 37.1) | 347 (265 to 456) | 1.2 (0.9 to 1.5) | 360 (276 to 476) | 1.3 (1 to 1.7) | 8806 (6596 to 11357) | 25.4 (19.4 to 33.6) | 77 (48 to 112) | 0.3 (0.2 to 0.4) | 8883 (6647 to 11479) | 25.7 (19.6 to 33.8) |
|  | Female | 123 (89 to 167) | 1.6 (1.2 to 2.2) | 125 (91 to 170) | 1.8 (1.3 to 2.4) | 3165 (2267 to 4323) | 35.6 (26.4 to 48.4) | 27 (17 to 41) | 0.4 (0.2 to 0.5) | 3192 (2283 to 4357) | 36 (26.7 to 49) | 230 (162 to 336) | 1.4 (1 to 2) | 241 (170 to 351) | 1.5 (1.1 to 2.2) | 5711 (3929 to 8395) | 30.3 (21.3 to 44.2) | 51 (30 to 81) | 0.3 (0.2 to 0.5) | 5762 (3964 to 8462) | 30.7 (21.6 to 44.7) |
|  | Male | 64 (43 to 86) | 1 (0.7 to 1.3) | 66 (45 to 89) | 1.1 (0.7 to 1.4) | 1635 (1082 to 2230) | 21.1 (14.3 to 28.2) | 14 (9 to 21) | 0.2 (0.1 to 0.3) | 1649 (1090 to 2245) | 21.3 (14.4 to 28.4) | 117 (78 to 164) | 0.9 (0.6 to 1.2) | 119 (80 to 167) | 1 (0.7 to 1.3) | 3095 (2022 to 4343) | 19.2 (13 to 26.9) | 26 (15 to 40) | 0.2 (0.1 to 0.3) | 3121 (2041 to 4386) | 19.4 (13.1 to 27.2) |
| Denmark | Both | 285 (255 to 322) | 3.4 (3 to 3.9) | 224 (208 to 234) | 2.6 (2.4 to 2.7) | 4036 (3743 to 4230) | 50.9 (47.1 to 53.4) | 69 (48 to 93) | 0.8 (0.6 to 1.1) | 4105 (3810 to 4299) | 51.7 (47.8 to 54.4) | 312 (260 to 379) | 2.7 (2.3 to 3.3) | 215 (190 to 236) | 1.8 (1.6 to 2) | 3589 (3109 to 3941) | 33 (28.6 to 36.2) | 81 (55 to 110) | 0.7 (0.5 to 1) | 3670 (3182 to 4041) | 33.7 (29.2 to 37.1) |
|  | Female | 169 (153 to 192) | 3.5 (3.2 to 4.1) | 139 (129 to 147) | 2.8 (2.6 to 2.9) | 2437 (2274 to 2583) | 55.9 (51.9 to 59.3) | 40 (27 to 53) | 0.9 (0.6 to 1.2) | 2477 (2303 to 2622) | 56.7 (52.7 to 60.3) | 160 (132 to 202) | 2.5 (2.1 to 3.2) | 116 (103 to 130) | 1.8 (1.6 to 2) | 1833 (1622 to 2060) | 31.7 (28 to 35.8) | 41 (27 to 58) | 0.7 (0.4 to 1) | 1873 (1658 to 2113) | 32.4 (28.6 to 36.5) |
|  | Male | 116 (95 to 149) | 3.3 (2.7 to 4.2) | 85 (72 to 91) | 2.4 (2.1 to 2.5) | 1599 (1356 to 1720) | 45.4 (38.6 to 48.9) | 29 (19 to 43) | 0.8 (0.5 to 1.2) | 1628 (1384 to 1751) | 46.2 (39.3 to 49.9) | 152 (112 to 204) | 2.9 (2.2 to 3.8) | 100 (77 to 113) | 1.9 (1.5 to 2.2) | 1756 (1325 to 2017) | 34.3 (26.1 to 39.4) | 40 (25 to 57) | 0.8 (0.5 to 1.1) | 1796 (1360 to 2059) | 35.1 (26.7 to 40.2) |
| Djibouti | Both | 2 (1 to 2) | 1.3 (0.9 to 1.8) | 2 (1 to 2) | 1.4 (1 to 2) | 45 (31 to 67) | 28 (19.7 to 41) | 0 (0 to 1) | 0.3 (0.2 to 0.4) | 45 (31 to 67) | 28.3 (20 to 41.4) | 5 (4 to 8) | 1 (0.7 to 1.5) | 5 (4 to 8) | 1.1 (0.8 to 1.7) | 136 (91 to 208) | 23 (15.8 to 34.2) | 1 (1 to 2) | 0.2 (0.1 to 0.4) | 137 (92 to 210) | 23.3 (16 to 34.6) |
|  | Female | 1 (1 to 2) | 1.6 (1 to 2.6) | 1 (1 to 2) | 1.8 (1.1 to 2.8) | 28 (17 to 46) | 36.2 (22.7 to 59.2) | 0 (0 to 0) | 0.4 (0.2 to 0.6) | 28 (17 to 47) | 36.6 (22.9 to 59.8) | 3 (2 to 5) | 1.3 (0.9 to 2) | 3 (2 to 5) | 1.4 (0.9 to 2.2) | 77 (50 to 125) | 28.5 (18.7 to 45.6) | 1 (0 to 1) | 0.3 (0.2 to 0.5) | 78 (50 to 126) | 28.8 (18.9 to 46.1) |
|  | Male | 1 (0 to 1) | 0.9 (0.6 to 1.3) | 1 (0 to 1) | 1 (0.7 to 1.4) | 17 (11 to 27) | 20.1 (13.1 to 30.8) | 0 (0 to 0) | 0.2 (0.1 to 0.3) | 17 (11 to 27) | 20.3 (13.2 to 31.1) | 2 (1 to 3) | 0.8 (0.5 to 1.2) | 2 (1 to 3) | 0.9 (0.6 to 1.3) | 58 (36 to 91) | 18.3 (11.7 to 27.8) | 0 (0 to 1) | 0.2 (0.1 to 0.3) | 59 (37 to 91) | 18.5 (11.8 to 28) |
| Dominica | Both | 3 (2 to 4) | 4.4 (3.3 to 4.8) | 3 (3 to 4) | 4.6 (3.6 to 5) | 67 (52 to 73) | 95.9 (74 to 104.1) | 1 (0 to 1) | 0.9 (0.6 to 1.3) | 68 (52 to 74) | 96.9 (74.9 to 105.2) | 1 (1 to 2) | 1.6 (1.4 to 2.4) | 2 (1 to 2) | 1.7 (1.5 to 2.5) | 31 (27 to 46) | 34.6 (30.5 to 51.6) | 0 (0 to 1) | 0.4 (0.2 to 0.5) | 31 (28 to 47) | 34.9 (30.8 to 52) |
|  | Female | 2 (2 to 2) | 4.6 (3.7 to 5) | 2 (2 to 2) | 4.8 (3.9 to 5.3) | 40 (33 to 44) | 100.4 (82.2 to 110.6) | 0 (0 to 1) | 1 (0.6 to 1.3) | 40 (33 to 45) | 101.4 (82.9 to 111.7) | 1 (1 to 1) | 1.7 (1.5 to 2.4) | 1 (1 to 1) | 1.8 (1.6 to 2.5) | 17 (15 to 23) | 37.6 (32.4 to 51.4) | 0 (0 to 0) | 0.4 (0.3 to 0.6) | 17 (15 to 24) | 38 (32.7 to 52.1) |
|  | Male | 1 (1 to 1) | 4.2 (2.4 to 4.7) | 1 (1 to 2) | 4.4 (2.6 to 5) | 27 (16 to 30) | 90.4 (53.3 to 101.1) | 0 (0 to 0) | 0.9 (0.5 to 1.3) | 27 (16 to 31) | 91.3 (53.7 to 102) | 1 (1 to 1) | 1.5 (1.2 to 2.5) | 1 (1 to 1) | 1.6 (1.3 to 2.7) | 14 (12 to 24) | 31.7 (26.6 to 54) | 0 (0 to 0) | 0.3 (0.2 to 0.6) | 14 (12 to 24) | 32 (26.9 to 54.5) |
| Dominican Republic | Both | 72 (48 to 82) | 1.9 (1.3 to 2.2) | 74 (50 to 84) | 2 (1.4 to 2.3) | 1754 (1157 to 2026) | 42.7 (28.6 to 49.1) | 16 (10 to 22) | 0.4 (0.3 to 0.6) | 1770 (1169 to 2041) | 43.1 (28.9 to 49.6) | 85 (62 to 163) | 0.9 (0.7 to 1.8) | 89 (64 to 168) | 1 (0.7 to 1.9) | 1974 (1425 to 3818) | 21 (15.1 to 40.6) | 19 (11 to 38) | 0.2 (0.1 to 0.4) | 1993 (1439 to 3855) | 21.2 (15.3 to 41) |
|  | Female | 40 (27 to 47) | 2.1 (1.4 to 2.5) | 41 (28 to 49) | 2.2 (1.5 to 2.6) | 975 (639 to 1146) | 47.2 (31.4 to 55.5) | 9 (6 to 12) | 0.5 (0.3 to 0.6) | 984 (645 to 1156) | 47.7 (31.7 to 56.1) | 44 (32 to 84) | 0.9 (0.7 to 1.8) | 46 (34 to 88) | 1 (0.7 to 1.9) | 1025 (733 to 1949) | 21.3 (15.2 to 40.8) | 10 (6 to 19) | 0.2 (0.1 to 0.4) | 1035 (739 to 1971) | 21.5 (15.4 to 41.2) |
|  | Male | 32 (19 to 38) | 1.7 (1 to 2) | 32 (19 to 39) | 1.8 (1.1 to 2.1) | 780 (443 to 950) | 38.2 (22.3 to 46.2) | 7 (4 to 10) | 0.4 (0.2 to 0.5) | 787 (448 to 959) | 38.5 (22.5 to 46.6) | 41 (26 to 87) | 0.9 (0.6 to 2) | 42 (27 to 90) | 1 (0.6 to 2.1) | 949 (604 to 2008) | 20.8 (13.2 to 44.2) | 9 (5 to 20) | 0.2 (0.1 to 0.5) | 958 (610 to 2027) | 21 (13.3 to 44.5) |
| East Asia | Both | 12285 (10942 to 18049) | 1.4 (1.3 to 2.1) | 12273 (10881 to 18347) | 1.5 (1.3 to 2.2) | 301431 (269224 to 435968) | 31.1 (27.8 to 45.3) | 2768 (1827 to 4255) | 0.3 (0.2 to 0.5) | 304199 (271834 to 439748) | 31.4 (28 to 45.8) | 34677 (25715 to 38062) | 1.8 (1.3 to 1.9) | 29742 (22670 to 32501) | 1.5 (1.2 to 1.7) | 611543 (468227 to 669786) | 29.4 (22.5 to 32.3) | 8183 (5236 to 11062) | 0.4 (0.3 to 0.6) | 619726 (474276 to 679363) | 29.8 (22.8 to 32.7) |
|  | Female | 6644 (5832 to 9906) | 1.5 (1.3 to 2.2) | 6681 (5825 to 10230) | 1.5 (1.3 to 2.4) | 158463 (140020 to 228402) | 32.4 (28.5 to 47.6) | 1490 (983 to 2288) | 0.3 (0.2 to 0.5) | 159953 (141168 to 230981) | 32.7 (28.7 to 48) | 18611 (12654 to 20956) | 1.8 (1.2 to 2) | 15802 (10970 to 17316) | 1.5 (1.1 to 1.7) | 316221 (222246 to 347328) | 29.7 (21 to 32.6) | 4342 (2732 to 5978) | 0.4 (0.3 to 0.6) | 320563 (225412 to 352267) | 30.2 (21.3 to 33.1) |
|  | Male | 5642 (4795 to 9126) | 1.4 (1.2 to 2.2) | 5592 (4758 to 9118) | 1.5 (1.3 to 2.3) | 142968 (121436 to 229876) | 30 (25.5 to 48.3) | 1278 (825 to 2062) | 0.3 (0.2 to 0.5) | 144246 (122646 to 231648) | 30.3 (25.7 to 48.9) | 16065 (11271 to 18862) | 1.7 (1.2 to 2) | 13940 (10045 to 16851) | 1.5 (1.1 to 1.8) | 295322 (212316 to 358817) | 29.1 (21 to 35.3) | 3841 (2437 to 5313) | 0.4 (0.3 to 0.6) | 299163 (215118 to 362854) | 29.5 (21.3 to 35.7) |
| Eastern Europe | Both | 4521 (4178 to 5094) | 1.6 (1.5 to 1.8) | 4508 (4251 to 5072) | 1.6 (1.5 to 1.8) | 97401 (91294 to 110112) | 33.5 (31.4 to 37.8) | 1023 (692 to 1369) | 0.4 (0.2 to 0.5) | 98424 (92290 to 111023) | 33.8 (31.7 to 38.2) | 5140 (4815 to 5569) | 1.5 (1.4 to 1.6) | 4672 (4465 to 4888) | 1.3 (1.3 to 1.4) | 95556 (91172 to 100352) | 28.3 (27 to 29.7) | 1204 (822 to 1607) | 0.4 (0.2 to 0.5) | 96759 (92262 to 101664) | 28.6 (27.3 to 30.1) |
|  | Female | 3085 (2899 to 3608) | 1.6 (1.5 to 1.9) | 3097 (2956 to 3572) | 1.6 (1.6 to 1.9) | 62914 (59709 to 73061) | 34 (32.1 to 39.7) | 694 (473 to 936) | 0.4 (0.3 to 0.5) | 63608 (60304 to 73844) | 34.4 (32.5 to 40.1) | 3381 (3101 to 3782) | 1.5 (1.4 to 1.7) | 3016 (2912 to 3170) | 1.3 (1.3 to 1.4) | 57600 (55301 to 60856) | 27.8 (26.6 to 29.5) | 787 (535 to 1061) | 0.4 (0.3 to 0.5) | 58387 (56105 to 61748) | 28.2 (27 to 29.9) |
|  | Male | 1435 (1249 to 1701) | 1.5 (1.3 to 1.7) | 1411 (1239 to 1656) | 1.5 (1.3 to 1.7) | 34487 (29968 to 40870) | 32.2 (28.2 to 38) | 329 (211 to 461) | 0.3 (0.2 to 0.5) | 34816 (30251 to 41270) | 32.5 (28.5 to 38.4) | 1760 (1575 to 1886) | 1.4 (1.2 to 1.5) | 1656 (1479 to 1734) | 1.3 (1.2 to 1.4) | 37956 (33557 to 39913) | 28.7 (25.5 to 30.2) | 416 (284 to 558) | 0.3 (0.2 to 0.4) | 38373 (33903 to 40377) | 29 (25.7 to 30.5) |
| Eastern Sub-Saharan Africa | Both | 1038 (824 to 1372) | 1.5 (1.2 to 2) | 1066 (850 to 1411) | 1.6 (1.3 to 2.2) | 26708 (21052 to 34656) | 33.1 (26.3 to 43.6) | 231 (152 to 331) | 0.3 (0.2 to 0.5) | 26939 (21213 to 35042) | 33.4 (26.5 to 44) | 1826 (1511 to 2337) | 1.3 (1 to 1.7) | 1894 (1568 to 2450) | 1.4 (1.1 to 1.8) | 46234 (38006 to 57870) | 28.1 (23.3 to 35.9) | 407 (267 to 562) | 0.3 (0.2 to 0.4) | 46641 (38395 to 58433) | 28.4 (23.5 to 36.2) |
|  | Female | 688 (535 to 918) | 1.9 (1.5 to 2.6) | 704 (551 to 947) | 2.1 (1.6 to 2.8) | 17780 (13759 to 23282) | 43.7 (34.1 to 58.5) | 153 (99 to 224) | 0.4 (0.3 to 0.6) | 17933 (13877 to 23454) | 44.1 (34.4 to 59) | 1181 (937 to 1610) | 1.5 (1.2 to 2.2) | 1224 (969 to 1691) | 1.7 (1.3 to 2.4) | 29802 (23497 to 39567) | 34.7 (27.5 to 46.7) | 263 (166 to 387) | 0.3 (0.2 to 0.5) | 30065 (23690 to 39801) | 35.1 (27.7 to 47.2) |
|  | Male | 350 (254 to 507) | 1 (0.7 to 1.4) | 363 (265 to 527) | 1.1 (0.8 to 1.6) | 8928 (6388 to 12949) | 22.5 (16.3 to 32.6) | 78 (47 to 115) | 0.2 (0.1 to 0.3) | 9006 (6453 to 13084) | 22.7 (16.4 to 33) | 645 (504 to 866) | 0.9 (0.7 to 1.3) | 670 (523 to 906) | 1 (0.8 to 1.4) | 16432 (12706 to 21930) | 21 (16.3 to 28.3) | 144 (92 to 212) | 0.2 (0.1 to 0.3) | 16577 (12805 to 22061) | 21.2 (16.5 to 28.5) |
| Ecuador | Both | 267 (252 to 280) | 5 (4.7 to 5.2) | 280 (265 to 293) | 5.4 (5.1 to 5.6) | 6294 (5981 to 6619) | 111.1 (105.3 to 116.3) | 57 (39 to 76) | 1.1 (0.7 to 1.4) | 6351 (6034 to 6676) | 112.2 (106.4 to 117.5) | 424 (375 to 556) | 2.9 (2.6 to 3.8) | 452 (402 to 586) | 3.1 (2.8 to 4.1) | 9125 (8025 to 12010) | 61.4 (54 to 80.6) | 92 (61 to 131) | 0.6 (0.4 to 0.9) | 9218 (8107 to 12121) | 62 (54.6 to 81.3) |
|  | Female | 189 (179 to 198) | 6.9 (6.5 to 7.2) | 198 (187 to 208) | 7.4 (7 to 7.7) | 4473 (4215 to 4730) | 155.3 (146.4 to 164.1) | 40 (27 to 54) | 1.5 (1 to 2) | 4513 (4259 to 4772) | 156.8 (148.1 to 165.7) | 291 (251 to 370) | 3.8 (3.3 to 4.9) | 311 (268 to 391) | 4.1 (3.6 to 5.2) | 6284 (5372 to 8159) | 81.9 (69.9 to 106.1) | 63 (42 to 92) | 0.8 (0.5 to 1.2) | 6346 (5428 to 8254) | 82.7 (70.7 to 107.3) |
|  | Male | 78 (68 to 83) | 3 (2.6 to 3.2) | 82 (71 to 88) | 3.3 (2.8 to 3.5) | 1821 (1636 to 1961) | 65.4 (57.9 to 70.4) | 17 (12 to 23) | 0.7 (0.4 to 0.9) | 1838 (1648 to 1982) | 66.1 (58.4 to 71) | 133 (113 to 197) | 1.9 (1.6 to 2.8) | 141 (120 to 208) | 2.1 (1.8 to 3.1) | 2842 (2385 to 4216) | 39.5 (33.1 to 58.4) | 30 (19 to 45) | 0.4 (0.3 to 0.7) | 2871 (2411 to 4259) | 39.9 (33.5 to 59) |
| Egypt | Both | 316 (274 to 439) | 1.2 (1 to 1.7) | 311 (270 to 446) | 1.2 (1.1 to 1.9) | 8635 (7386 to 11084) | 27 (23.4 to 37.9) | 71 (48 to 104) | 0.3 (0.2 to 0.4) | 8706 (7440 to 11193) | 27.3 (23.7 to 38.2) | 582 (471 to 791) | 1.1 (0.9 to 1.6) | 567 (457 to 800) | 1.1 (0.9 to 1.7) | 15459 (12441 to 20438) | 23.8 (19.3 to 32.7) | 132 (87 to 199) | 0.2 (0.2 to 0.4) | 15590 (12557 to 20619) | 24 (19.5 to 33) |
|  | Female | 164 (140 to 256) | 1.2 (1 to 2.1) | 160 (137 to 257) | 1.3 (1.1 to 2.2) | 4557 (3915 to 6483) | 28.8 (24.7 to 45.5) | 37 (24 to 56) | 0.3 (0.2 to 0.5) | 4594 (3946 to 6544) | 29 (25 to 46) | 273 (205 to 403) | 1 (0.8 to 1.7) | 269 (198 to 412) | 1.1 (0.8 to 1.9) | 7315 (5493 to 10274) | 23.3 (17.4 to 34.5) | 62 (38 to 97) | 0.2 (0.1 to 0.4) | 7377 (5528 to 10387) | 23.6 (17.6 to 34.8) |
|  | Male | 153 (118 to 211) | 1.1 (0.9 to 1.6) | 151 (118 to 215) | 1.2 (1 to 1.7) | 4078 (2940 to 5399) | 25.4 (19.5 to 35.6) | 34 (22 to 49) | 0.2 (0.2 to 0.4) | 4112 (2972 to 5456) | 25.6 (19.7 to 35.9) | 308 (213 to 429) | 1.1 (0.8 to 1.5) | 298 (209 to 408) | 1.1 (0.8 to 1.6) | 8144 (5371 to 11132) | 24.1 (16.7 to 33.1) | 70 (43 to 106) | 0.2 (0.2 to 0.4) | 8214 (5416 to 11230) | 24.3 (16.9 to 33.6) |
| El Salvador | Both | 99 (90 to 132) | 3.3 (3 to 4.5) | 103 (93 to 137) | 3.5 (3.2 to 4.7) | 2374 (2133 to 3006) | 75.9 (68.5 to 98.3) | 22 (14 to 30) | 0.7 (0.5 to 1) | 2396 (2154 to 3041) | 76.7 (69 to 99) | 165 (132 to 240) | 2.9 (2.3 to 4.2) | 175 (140 to 252) | 3.1 (2.4 to 4.4) | 3600 (2863 to 5188) | 63.8 (50.7 to 92.1) | 36 (23 to 55) | 0.6 (0.4 to 1) | 3636 (2895 to 5245) | 64.4 (51.3 to 93.1) |
|  | Female | 77 (68 to 103) | 4.8 (4.3 to 6.5) | 79 (70 to 108) | 5 (4.4 to 6.9) | 1847 (1620 to 2382) | 111 (97.8 to 145.5) | 17 (11 to 23) | 1 (0.7 to 1.5) | 1864 (1635 to 2404) | 112 (98.9 to 146.5) | 124 (94 to 191) | 3.8 (2.9 to 5.9) | 131 (99 to 203) | 4 (3 to 6.2) | 2731 (2060 to 4180) | 84.5 (63.6 to 129.5) | 27 (17 to 43) | 0.8 (0.5 to 1.3) | 2758 (2086 to 4223) | 85.3 (64.3 to 130.8) |
|  | Male | 23 (19 to 33) | 1.7 (1.4 to 2.4) | 24 (20 to 34) | 1.8 (1.5 to 2.6) | 527 (442 to 739) | 36.1 (30.5 to 50.8) | 5 (3 to 8) | 0.4 (0.2 to 0.6) | 532 (446 to 745) | 36.4 (30.8 to 51.2) | 41 (31 to 60) | 1.7 (1.3 to 2.5) | 43 (33 to 63) | 1.8 (1.4 to 2.6) | 869 (652 to 1270) | 35.9 (26.9 to 52.5) | 9 (6 to 14) | 0.4 (0.2 to 0.6) | 878 (657 to 1286) | 36.2 (27.2 to 53.1) |
| Equatorial Guinea | Both | 3 (2 to 4) | 1.5 (1.1 to 2.1) | 3 (2 to 4) | 1.7 (1.2 to 2.2) | 71 (47 to 106) | 34.7 (24 to 49.2) | 1 (0 to 1) | 0.3 (0.2 to 0.5) | 72 (48 to 107) | 35.1 (24.3 to 49.5) | 5 (3 to 8) | 1.3 (0.8 to 1.9) | 6 (4 to 8) | 1.4 (0.9 to 2.1) | 133 (83 to 200) | 28 (17.8 to 41.8) | 1 (1 to 2) | 0.3 (0.2 to 0.4) | 134 (84 to 202) | 28.3 (17.9 to 42.3) |
|  | Female | 2 (1 to 3) | 1.8 (1.2 to 2.4) | 2 (1 to 3) | 1.9 (1.4 to 2.6) | 44 (29 to 68) | 39.9 (26.6 to 58.4) | 0 (0 to 1) | 0.4 (0.2 to 0.6) | 45 (29 to 69) | 40.3 (26.8 to 58.9) | 4 (2 to 6) | 1.6 (0.9 to 2.5) | 4 (2 to 6) | 1.7 (1 to 2.7) | 95 (52 to 151) | 34.9 (19.5 to 54.8) | 1 (0 to 1) | 0.4 (0.2 to 0.6) | 96 (52 to 152) | 35.3 (19.7 to 55.5) |
|  | Male | 1 (1 to 2) | 1.2 (0.7 to 1.9) | 1 (1 to 2) | 1.3 (0.8 to 2) | 27 (14 to 45) | 28.4 (15.6 to 46) | 0 (0 to 0) | 0.3 (0.1 to 0.5) | 27 (14 to 46) | 28.6 (15.8 to 46.4) | 2 (1 to 2) | 0.9 (0.6 to 1.3) | 2 (1 to 2) | 1 (0.6 to 1.4) | 38 (24 to 56) | 18.6 (11.7 to 27.3) | 0 (0 to 1) | 0.2 (0.1 to 0.3) | 38 (24 to 57) | 18.8 (11.8 to 27.6) |
| Eritrea | Both | 13 (9 to 18) | 1.6 (1.2 to 2.2) | 13 (10 to 19) | 1.7 (1.3 to 2.5) | 358 (262 to 512) | 36 (27 to 51.4) | 3 (2 to 4) | 0.3 (0.2 to 0.5) | 361 (264 to 517) | 36.3 (27.3 to 52) | 29 (22 to 39) | 1.5 (1.1 to 2) | 30 (22 to 40) | 1.6 (1.3 to 2.3) | 792 (593 to 1069) | 33.5 (25.3 to 45.3) | 7 (4 to 10) | 0.3 (0.2 to 0.5) | 798 (598 to 1078) | 33.9 (25.5 to 45.7) |
|  | Female | 7 (5 to 12) | 1.6 (1.2 to 2.5) | 8 (5 to 12) | 1.8 (1.3 to 2.7) | 200 (137 to 326) | 36.4 (25.4 to 58.1) | 2 (1 to 3) | 0.4 (0.2 to 0.6) | 201 (138 to 328) | 36.7 (25.6 to 58.6) | 19 (13 to 28) | 1.6 (1.2 to 2.4) | 19 (14 to 29) | 1.8 (1.3 to 2.7) | 474 (336 to 715) | 35.1 (25.5 to 53.5) | 4 (2 to 6) | 0.4 (0.2 to 0.5) | 479 (339 to 723) | 35.5 (25.7 to 54) |
|  | Male | 5 (3 to 8) | 1.4 (1 to 2) | 5 (3 to 8) | 1.5 (1.1 to 2.1) | 159 (97 to 233) | 34.6 (22.8 to 48.9) | 1 (1 to 2) | 0.3 (0.2 to 0.5) | 160 (98 to 234) | 34.9 (23 to 49.3) | 10 (7 to 15) | 1.2 (0.8 to 1.7) | 10 (7 to 15) | 1.3 (0.9 to 1.9) | 317 (201 to 463) | 29.6 (19.6 to 42.9) | 2 (1 to 4) | 0.3 (0.2 to 0.4) | 320 (203 to 467) | 29.8 (19.8 to 43.2) |
| Estonia | Both | 45 (41 to 49) | 2.1 (2 to 2.3) | 46 (43 to 49) | 2.2 (2 to 2.3) | 935 (870 to 1002) | 44.6 (41.5 to 47.8) | 10 (7 to 13) | 0.5 (0.3 to 0.6) | 945 (880 to 1012) | 45.1 (42 to 48.3) | 50 (41 to 62) | 1.8 (1.5 to 2.2) | 48 (40 to 55) | 1.7 (1.4 to 1.9) | 807 (681 to 935) | 31.9 (27 to 37.1) | 11 (7 to 16) | 0.4 (0.3 to 0.6) | 818 (690 to 950) | 32.3 (27.3 to 37.6) |
|  | Female | 31 (28 to 35) | 2.3 (2.1 to 2.5) | 32 (30 to 35) | 2.3 (2.1 to 2.5) | 617 (568 to 669) | 46.7 (43.1 to 50.6) | 7 (5 to 9) | 0.5 (0.3 to 0.7) | 624 (575 to 677) | 47.2 (43.6 to 51.1) | 34 (26 to 46) | 1.9 (1.5 to 2.5) | 32 (25 to 38) | 1.7 (1.4 to 2) | 496 (400 to 605) | 31.7 (25.6 to 39) | 8 (5 to 12) | 0.5 (0.3 to 0.7) | 504 (407 to 614) | 32.1 (25.9 to 39.5) |
|  | Male | 13 (11 to 15) | 1.8 (1.5 to 2) | 14 (11 to 15) | 1.9 (1.6 to 2.1) | 318 (266 to 352) | 40.3 (33.7 to 44.4) | 3 (2 to 4) | 0.4 (0.3 to 0.5) | 321 (268 to 355) | 40.7 (34.2 to 44.8) | 16 (12 to 20) | 1.6 (1.2 to 2) | 16 (12 to 20) | 1.7 (1.2 to 2.1) | 311 (238 to 381) | 32.2 (24.9 to 39.5) | 4 (2 to 5) | 0.4 (0.2 to 0.5) | 314 (241 to 386) | 32.5 (25.1 to 40) |
| Ethiopia | Both | 407 (274 to 533) | 2.2 (1.5 to 3) | 410 (275 to 539) | 2.4 (1.6 to 3.3) | 10850 (7291 to 14034) | 50.5 (33.9 to 66.9) | 91 (54 to 134) | 0.5 (0.3 to 0.7) | 10941 (7354 to 14132) | 51 (34.3 to 67.4) | 637 (449 to 854) | 1.7 (1.2 to 2.3) | 658 (466 to 893) | 1.8 (1.3 to 2.6) | 16137 (11331 to 20916) | 38.4 (27.1 to 51.1) | 142 (87 to 208) | 0.4 (0.2 to 0.5) | 16279 (11428 to 21115) | 38.8 (27.3 to 51.7) |
|  | Female | 293 (184 to 412) | 3.1 (2 to 4.4) | 294 (186 to 415) | 3.3 (2.2 to 4.8) | 7798 (4877 to 10970) | 73.2 (46.2 to 103.3) | 65 (37 to 103) | 0.7 (0.4 to 1.1) | 7863 (4919 to 11078) | 73.9 (46.8 to 104.6) | 434 (263 to 606) | 2.3 (1.4 to 3.4) | 445 (271 to 628) | 2.5 (1.5 to 3.7) | 11155 (6771 to 15149) | 53.9 (32.9 to 74.5) | 97 (50 to 148) | 0.5 (0.3 to 0.8) | 11252 (6817 to 15270) | 54.5 (33.2 to 75.3) |
|  | Male | 115 (61 to 217) | 1.3 (0.7 to 2.4) | 115 (61 to 222) | 1.4 (0.8 to 2.6) | 3052 (1586 to 5664) | 28.4 (15.3 to 54.3) | 26 (12 to 49) | 0.3 (0.1 to 0.5) | 3078 (1600 to 5720) | 28.7 (15.5 to 54.9) | 203 (159 to 284) | 1.1 (0.8 to 1.5) | 213 (168 to 300) | 1.2 (0.9 to 1.7) | 4982 (3887 to 6723) | 23.7 (18.5 to 32.7) | 45 (28 to 68) | 0.2 (0.2 to 0.4) | 5027 (3919 to 6793) | 24 (18.7 to 33.1) |
| Federated States of Micronesia | Both | 1 (1 to 1) | 1.5 (1.2 to 1.8) | 1 (1 to 1) | 1.6 (1.3 to 2) | 17 (13 to 21) | 33 (25.2 to 40.6) | 0 (0 to 0) | 0.3 (0.2 to 0.5) | 17 (13 to 21) | 33.3 (25.4 to 40.9) | 1 (1 to 1) | 1.2 (0.9 to 1.4) | 1 (1 to 1) | 1.3 (1 to 1.5) | 19 (14 to 24) | 25.4 (19.5 to 31.3) | 0 (0 to 0) | 0.3 (0.2 to 0.4) | 19 (14 to 24) | 25.6 (19.7 to 31.6) |
|  | Female | 0 (0 to 1) | 1.7 (1.2 to 2.3) | 0 (0 to 1) | 1.9 (1.3 to 2.4) | 10 (7 to 13) | 39.1 (27.4 to 51.2) | 0 (0 to 0) | 0.4 (0.2 to 0.6) | 10 (7 to 13) | 39.5 (27.7 to 51.7) | 0 (0 to 1) | 1.3 (0.9 to 1.7) | 0 (0 to 1) | 1.4 (1 to 1.9) | 11 (7 to 15) | 28.3 (19.4 to 39.1) | 0 (0 to 0) | 0.3 (0.2 to 0.4) | 11 (7 to 15) | 28.6 (19.6 to 39.6) |
|  | Male | 0 (0 to 0) | 1.2 (0.9 to 1.7) | 0 (0 to 0) | 1.3 (1 to 1.8) | 7 (5 to 9) | 26.7 (19.5 to 36.8) | 0 (0 to 0) | 0.3 (0.2 to 0.4) | 7 (5 to 9) | 27 (19.7 to 37.1) | 0 (0 to 0) | 1 (0.7 to 1.4) | 0 (0 to 0) | 1.1 (0.8 to 1.5) | 8 (5 to 11) | 22.2 (15.1 to 30.3) | 0 (0 to 0) | 0.2 (0.1 to 0.4) | 8 (5 to 11) | 22.4 (15.2 to 30.7) |
| Fiji | Both | 4 (3 to 5) | 1.2 (0.9 to 1.4) | 4 (3 to 5) | 1.3 (1 to 1.5) | 109 (81 to 130) | 27 (20.1 to 32.1) | 1 (1 to 1) | 0.3 (0.2 to 0.4) | 110 (81 to 130) | 27.3 (20.3 to 32.4) | 8 (5 to 10) | 1.2 (0.8 to 1.4) | 8 (5 to 10) | 1.2 (0.9 to 1.4) | 197 (132 to 238) | 25.5 (17.3 to 30.6) | 2 (1 to 3) | 0.3 (0.2 to 0.4) | 199 (133 to 240) | 25.7 (17.5 to 30.8) |
|  | Female | 3 (2 to 3) | 1.4 (1 to 1.7) | 2 (2 to 3) | 1.4 (1.1 to 1.8) | 67 (47 to 86) | 31.7 (22.8 to 40.4) | 1 (0 to 1) | 0.3 (0.2 to 0.4) | 67 (47 to 87) | 32.1 (22.9 to 40.9) | 5 (3 to 6) | 1.2 (0.8 to 1.5) | 5 (3 to 6) | 1.3 (0.9 to 1.6) | 116 (71 to 150) | 28.5 (17.8 to 36.4) | 1 (1 to 2) | 0.3 (0.2 to 0.4) | 117 (71 to 151) | 28.7 (18.1 to 36.8) |
|  | Male | 2 (1 to 2) | 1 (0.7 to 1.3) | 2 (1 to 2) | 1.1 (0.8 to 1.4) | 42 (29 to 53) | 22.3 (15.4 to 28.4) | 0 (0 to 1) | 0.2 (0.1 to 0.3) | 42 (29 to 53) | 22.5 (15.5 to 28.7) | 3 (2 to 4) | 1.1 (0.7 to 1.4) | 3 (2 to 4) | 1.2 (0.7 to 1.5) | 81 (50 to 103) | 22.8 (14 to 28.6) | 1 (0 to 1) | 0.2 (0.1 to 0.4) | 82 (50 to 104) | 23 (14.2 to 28.9) |
| Finland | Both | 326 (280 to 367) | 4.4 (3.8 to 5) | 263 (232 to 275) | 3.6 (3.1 to 3.7) | 4671 (4121 to 4889) | 64.4 (56.8 to 67.4) | 76 (52 to 103) | 1 (0.7 to 1.4) | 4747 (4179 to 4966) | 65.5 (57.7 to 68.5) | 363 (297 to 434) | 2.9 (2.4 to 3.5) | 263 (234 to 287) | 2 (1.8 to 2.2) | 4143 (3632 to 4538) | 35.9 (31.7 to 39.5) | 90 (59 to 125) | 0.7 (0.5 to 1) | 4233 (3697 to 4648) | 36.6 (32.4 to 40.4) |
|  | Female | 222 (201 to 242) | 4.8 (4.3 to 5.3) | 187 (176 to 198) | 4 (3.7 to 4.2) | 3172 (2968 to 3355) | 73.2 (67.9 to 77.5) | 50 (33 to 68) | 1.1 (0.7 to 1.5) | 3222 (3017 to 3419) | 74.3 (68.9 to 78.8) | 206 (169 to 257) | 2.9 (2.4 to 3.6) | 158 (140 to 177) | 2.1 (1.9 to 2.3) | 2347 (2060 to 2634) | 37 (32.5 to 41.6) | 49 (32 to 70) | 0.7 (0.5 to 1) | 2396 (2099 to 2696) | 37.7 (33.1 to 42.5) |
|  | Male | 104 (70 to 138) | 3.8 (2.6 to 4.9) | 76 (53 to 82) | 2.8 (2 to 3.1) | 1499 (1060 to 1617) | 52 (36.9 to 56) | 26 (16 to 39) | 0.9 (0.6 to 1.4) | 1525 (1077 to 1646) | 52.9 (37.5 to 57.1) | 156 (106 to 209) | 3 (2.1 to 3.9) | 105 (72 to 120) | 1.9 (1.4 to 2.2) | 1796 (1272 to 2058) | 34.6 (25.3 to 39.5) | 40 (24 to 60) | 0.8 (0.5 to 1.1) | 1837 (1304 to 2099) | 35.4 (25.9 to 40.4) |
| France | Both | 3249 (2614 to 3989) | 3.7 (3 to 4.6) | 2703 (2307 to 2809) | 3 (2.6 to 3.1) | 44811 (37730 to 46558) | 54 (45.4 to 56.2) | 752 (492 to 1048) | 0.9 (0.6 to 1.2) | 45563 (38361 to 47440) | 54.9 (46 to 57.2) | 2525 (2153 to 3130) | 1.8 (1.5 to 2.2) | 2183 (1995 to 2390) | 1.4 (1.3 to 1.6) | 31513 (28950 to 34925) | 24.9 (22.8 to 27.6) | 597 (404 to 833) | 0.4 (0.3 to 0.6) | 32110 (29513 to 35558) | 25.3 (23.2 to 28.1) |
|  | Female | 1816 (1525 to 2303) | 3.4 (2.8 to 4.1) | 1640 (1519 to 1720) | 2.9 (2.7 to 3) | 24606 (23187 to 25783) | 50.4 (47.8 to 52.8) | 394 (256 to 566) | 0.8 (0.5 to 1) | 25000 (23544 to 26206) | 51.2 (48.5 to 53.7) | 1253 (1052 to 1693) | 1.4 (1.2 to 1.8) | 1186 (1072 to 1329) | 1.2 (1.1 to 1.4) | 15247 (13725 to 17206) | 21.3 (19 to 24) | 280 (185 to 409) | 0.3 (0.2 to 0.5) | 15526 (13944 to 17497) | 21.6 (19.3 to 24.4) |
|  | Male | 1433 (1007 to 1909) | 4.2 (2.9 to 5.5) | 1063 (738 to 1126) | 3.1 (2.2 to 3.3) | 20204 (14027 to 21419) | 58 (40.2 to 61.5) | 358 (211 to 551) | 1 (0.6 to 1.6) | 20562 (14264 to 21768) | 59 (41 to 62.5) | 1272 (979 to 1681) | 2.2 (1.7 to 2.9) | 997 (827 to 1148) | 1.6 (1.4 to 1.9) | 16266 (13931 to 18711) | 29.1 (25.1 to 33.4) | 318 (199 to 483) | 0.6 (0.3 to 0.8) | 16584 (14296 to 19048) | 29.7 (25.6 to 34) |
| Gabon | Both | 9 (7 to 12) | 1.7 (1.3 to 2.3) | 10 (7 to 13) | 1.9 (1.4 to 2.5) | 219 (159 to 289) | 37.6 (27.6 to 49.3) | 2 (1 to 3) | 0.4 (0.2 to 0.6) | 221 (160 to 291) | 38 (27.8 to 49.8) | 12 (9 to 16) | 1.3 (1 to 1.6) | 13 (10 to 16) | 1.4 (1.1 to 1.8) | 290 (226 to 375) | 27.4 (21.2 to 35) | 3 (2 to 4) | 0.3 (0.2 to 0.4) | 293 (228 to 379) | 27.7 (21.5 to 35.4) |
|  | Female | 6 (4 to 9) | 2.1 (1.4 to 3) | 7 (5 to 10) | 2.3 (1.6 to 3.2) | 151 (98 to 210) | 47 (30.9 to 65.5) | 1 (1 to 2) | 0.5 (0.3 to 0.7) | 152 (99 to 212) | 47.4 (31.2 to 66.1) | 8 (5 to 11) | 1.5 (1 to 2.1) | 8 (6 to 12) | 1.6 (1.1 to 2.3) | 177 (122 to 256) | 32.3 (22 to 46.9) | 2 (1 to 3) | 0.3 (0.2 to 0.5) | 179 (123 to 258) | 32.6 (22.3 to 47.3) |
|  | Male | 3 (2 to 4) | 1.2 (0.8 to 1.5) | 3 (2 to 4) | 1.3 (0.9 to 1.7) | 68 (44 to 92) | 25.9 (16.8 to 34.2) | 1 (0 to 1) | 0.3 (0.2 to 0.4) | 69 (44 to 93) | 26.2 (17 to 34.6) | 4 (3 to 6) | 1 (0.7 to 1.3) | 5 (3 to 6) | 1.1 (0.8 to 1.4) | 114 (84 to 149) | 22 (16.4 to 28.6) | 1 (1 to 2) | 0.2 (0.1 to 0.3) | 115 (84 to 150) | 22.2 (16.5 to 28.9) |
| Georgia | Both | 75 (57 to 121) | 1.2 (0.9 to 2) | 77 (59 to 125) | 1.2 (1 to 2) | 1768 (1331 to 2715) | 27.6 (21 to 42.8) | 17 (11 to 26) | 0.3 (0.2 to 0.4) | 1785 (1341 to 2741) | 27.9 (21.2 to 43.3) | 125 (114 to 136) | 2.1 (1.9 to 2.3) | 133 (121 to 145) | 2.2 (2 to 2.4) | 2838 (2557 to 3096) | 49.8 (44.8 to 54) | 28 (19 to 37) | 0.5 (0.3 to 0.6) | 2866 (2580 to 3127) | 50.3 (45.2 to 54.5) |
|  | Female | 53 (39 to 87) | 1.4 (1 to 2.3) | 54 (40 to 90) | 1.4 (1.1 to 2.4) | 1238 (901 to 1907) | 33.2 (24.2 to 50.3) | 12 (8 to 18) | 0.3 (0.2 to 0.5) | 1250 (909 to 1928) | 33.5 (24.4 to 50.9) | 89 (79 to 99) | 2.5 (2.3 to 2.8) | 95 (85 to 106) | 2.6 (2.4 to 2.9) | 1980 (1746 to 2190) | 60.8 (53.5 to 67.1) | 20 (13 to 26) | 0.6 (0.4 to 0.8) | 1999 (1761 to 2213) | 61.3 (53.8 to 67.6) |
|  | Male | 22 (17 to 33) | 0.9 (0.7 to 1.4) | 22 (17 to 34) | 1 (0.8 to 1.5) | 530 (406 to 800) | 20.3 (15.8 to 31.2) | 5 (3 to 7) | 0.2 (0.1 to 0.3) | 535 (410 to 807) | 20.5 (16 to 31.5) | 36 (31 to 41) | 1.5 (1.3 to 1.7) | 38 (32 to 43) | 1.6 (1.4 to 1.8) | 858 (731 to 976) | 35.7 (30.6 to 40.4) | 8 (5 to 11) | 0.3 (0.2 to 0.5) | 866 (739 to 984) | 36 (30.9 to 40.8) |
| Germany | Both | 9220 (7576 to 10342) | 6.9 (5.7 to 7.7) | 7328 (6242 to 7648) | 5.4 (4.6 to 5.6) | 126316 (106777 to 131505) | 98.1 (82.9 to 102) | 2043 (1346 to 2715) | 1.5 (1 to 2) | 128359 (108509 to 133783) | 99.7 (84.2 to 103.8) | 8508 (6860 to 10642) | 4.3 (3.5 to 5.3) | 4986 (4421 to 5573) | 2.5 (2.2 to 2.7) | 78454 (69260 to 88516) | 44.5 (39.2 to 50.3) | 2175 (1427 to 3086) | 1.1 (0.8 to 1.6) | 80628 (71468 to 90639) | 45.7 (40.4 to 51.5) |
|  | Female | 6478 (5480 to 7480) | 7.5 (6.4 to 8.5) | 5159 (4253 to 5434) | 5.9 (4.8 to 6.2) | 84499 (69212 to 88791) | 108.3 (90.9 to 113.6) | 1416 (914 to 1942) | 1.7 (1.1 to 2.2) | 85915 (70610 to 90207) | 110 (92.2 to 115.5) | 4593 (3450 to 6456) | 4.1 (3.1 to 5.5) | 2844 (2421 to 3372) | 2.4 (2 to 2.8) | 41413 (34987 to 49569) | 42.4 (35.6 to 51) | 1141 (713 to 1774) | 1.1 (0.7 to 1.6) | 42554 (36031 to 51057) | 43.5 (36.5 to 52.3) |
|  | Male | 2742 (1763 to 3444) | 5.8 (3.7 to 7.3) | 2169 (1408 to 2297) | 4.6 (3 to 4.8) | 41817 (28191 to 44273) | 83.3 (55.9 to 88.1) | 627 (370 to 887) | 1.3 (0.8 to 1.8) | 42444 (28584 to 44943) | 84.6 (56.9 to 89.5) | 3916 (2912 to 5286) | 4.6 (3.5 to 6.1) | 2142 (1777 to 2477) | 2.5 (2.1 to 2.9) | 37040 (30617 to 43063) | 46.6 (38.6 to 54.5) | 1034 (638 to 1551) | 1.2 (0.8 to 1.8) | 38074 (31758 to 44216) | 47.9 (39.7 to 55.8) |
| Ghana | Both | 74 (56 to 100) | 1.3 (1 to 1.7) | 76 (58 to 102) | 1.4 (1.1 to 1.8) | 1782 (1325 to 2455) | 27.2 (20.6 to 36.6) | 16 (10 to 24) | 0.3 (0.2 to 0.4) | 1798 (1340 to 2484) | 27.4 (20.8 to 37.1) | 166 (119 to 242) | 1.2 (0.9 to 1.7) | 172 (124 to 246) | 1.3 (1 to 1.8) | 3941 (2769 to 5789) | 25.2 (18 to 36.6) | 37 (22 to 57) | 0.3 (0.2 to 0.4) | 3977 (2797 to 5850) | 25.5 (18.2 to 37.1) |
|  | Female | 41 (30 to 58) | 1.4 (1 to 1.9) | 43 (31 to 59) | 1.5 (1.1 to 2.1) | 989 (704 to 1428) | 29.5 (21.3 to 41.7) | 9 (6 to 14) | 0.3 (0.2 to 0.5) | 998 (709 to 1441) | 29.8 (21.6 to 42.1) | 97 (70 to 138) | 1.3 (0.9 to 1.8) | 102 (74 to 145) | 1.4 (1 to 2) | 2278 (1626 to 3260) | 26.5 (19.1 to 38) | 21 (13 to 33) | 0.3 (0.2 to 0.4) | 2299 (1643 to 3288) | 26.8 (19.3 to 38.3) |
|  | Male | 33 (21 to 48) | 1.2 (0.8 to 1.6) | 33 (21 to 49) | 1.2 (0.8 to 1.8) | 793 (491 to 1190) | 24.6 (15.8 to 36.2) | 7 (4 to 12) | 0.3 (0.1 to 0.4) | 800 (495 to 1200) | 24.9 (16 to 36.6) | 69 (39 to 115) | 1.1 (0.7 to 1.8) | 70 (40 to 117) | 1.2 (0.7 to 1.9) | 1663 (913 to 2803) | 23.6 (13.4 to 39.4) | 15 (7 to 26) | 0.2 (0.1 to 0.4) | 1678 (922 to 2824) | 23.8 (13.6 to 39.8) |
| Global | Both | 119943 (115271 to 133179) | 3.1 (3 to 3.5) | 105519 (101188 to 119200) | 2.8 (2.7 to 3.1) | 2258194 (2150541 to 2561979) | 54.5 (52 to 61.7) | 27318 (19111 to 35326) | 0.7 (0.5 to 0.9) | 2285512 (2172147 to 2595002) | 55.2 (52.5 to 62.5) | 210878 (186150 to 225429) | 2.7 (2.4 to 2.9) | 173974 (154232 to 184875) | 2.2 (2 to 2.4) | 3433985 (3009717 to 3659954) | 42.6 (37.3 to 45.4) | 49061 (34441 to 64307) | 0.6 (0.4 to 0.8) | 3483046 (3041856 to 3713905) | 43.2 (37.7 to 46.1) |
|  | Female | 74244 (71033 to 82834) | 3.5 (3.3 to 3.9) | 66018 (63091 to 74639) | 3.1 (3 to 3.5) | 1368412 (1294957 to 1565864) | 61.8 (58.6 to 70.8) | 16614 (11600 to 21653) | 0.8 (0.5 to 1) | 1385026 (1312703 to 1584446) | 62.6 (59.5 to 71.6) | 120498 (103966 to 131258) | 2.8 (2.4 to 3.1) | 102422 (88786 to 109618) | 2.4 (2.1 to 2.6) | 1993885 (1680853 to 2131303) | 46.9 (39.5 to 50.1) | 27394 (18963 to 36108) | 0.6 (0.4 to 0.8) | 2021279 (1708064 to 2156265) | 47.5 (40.2 to 50.7) |
|  | Male | 45699 (41745 to 53053) | 2.7 (2.5 to 3.1) | 39500 (35810 to 47186) | 2.4 (2.2 to 2.8) | 889782 (793833 to 1081856) | 46.2 (41.4 to 55.6) | 10705 (7463 to 14081) | 0.6 (0.4 to 0.8) | 900486 (804092 to 1092390) | 46.8 (41.9 to 56.3) | 90380 (76937 to 99871) | 2.6 (2.2 to 2.9) | 71552 (60221 to 78997) | 2.1 (1.8 to 2.3) | 1440100 (1194940 to 1600766) | 38.1 (31.7 to 42.3) | 21667 (15009 to 28850) | 0.6 (0.4 to 0.8) | 1461767 (1216186 to 1623957) | 38.7 (32.2 to 42.9) |
| Greece | Both | 332 (280 to 437) | 2.1 (1.8 to 2.8) | 251 (235 to 309) | 1.6 (1.5 to 2) | 4714 (4438 to 5742) | 30.5 (28.7 to 37.2) | 83 (55 to 121) | 0.5 (0.4 to 0.8) | 4797 (4519 to 5866) | 31 (29.2 to 37.9) | 588 (482 to 698) | 2.5 (2 to 3) | 512 (431 to 555) | 2 (1.7 to 2.2) | 8074 (6720 to 8830) | 37.5 (30.8 to 41.1) | 139 (91 to 192) | 0.6 (0.4 to 0.9) | 8213 (6819 to 8989) | 38.1 (31.3 to 41.8) |
|  | Female | 158 (132 to 213) | 1.8 (1.6 to 2.5) | 129 (119 to 155) | 1.5 (1.4 to 1.8) | 2392 (2225 to 2637) | 28.8 (26.8 to 31.3) | 38 (25 to 55) | 0.4 (0.3 to 0.6) | 2429 (2260 to 2671) | 29.3 (27.2 to 31.6) | 263 (221 to 330) | 2 (1.7 to 2.4) | 244 (216 to 275) | 1.7 (1.5 to 1.9) | 3747 (3288 to 4174) | 32.5 (28.3 to 36.3) | 60 (40 to 87) | 0.5 (0.3 to 0.7) | 3807 (3340 to 4235) | 33 (28.7 to 36.9) |
|  | Male | 174 (135 to 262) | 2.4 (1.9 to 3.6) | 122 (111 to 170) | 1.7 (1.6 to 2.4) | 2323 (2108 to 3178) | 32.6 (29.7 to 44.6) | 45 (29 to 71) | 0.6 (0.4 to 1) | 2368 (2152 to 3247) | 33.2 (30.3 to 45.5) | 325 (223 to 417) | 3.1 (2.1 to 4) | 267 (180 to 303) | 2.3 (1.6 to 2.6) | 4327 (2911 to 4914) | 43.3 (29 to 49.5) | 79 (47 to 115) | 0.8 (0.5 to 1.1) | 4406 (2960 to 5005) | 44.1 (29.5 to 50.4) |
| Greenland | Both | 1 (1 to 1) | 3.1 (1.8 to 3.7) | 1 (1 to 1) | 3.2 (1.9 to 3.9) | 21 (14 to 26) | 62 (38.5 to 75) | 0 (0 to 0) | 0.7 (0.4 to 1) | 22 (14 to 26) | 62.7 (38.8 to 75.8) | 2 (1 to 2) | 2.4 (1.5 to 2.8) | 2 (1 to 2) | 2.5 (1.5 to 2.9) | 32 (20 to 37) | 46.2 (29.4 to 53.5) | 0 (0 to 0) | 0.5 (0.3 to 0.8) | 32 (21 to 38) | 46.7 (29.7 to 54.2) |
|  | Female | 1 (0 to 1) | 3.2 (2 to 3.8) | 1 (0 to 1) | 3.4 (2.1 to 4) | 12 (8 to 14) | 67.9 (42.8 to 81.1) | 0 (0 to 0) | 0.7 (0.4 to 1) | 12 (8 to 15) | 68.6 (43.3 to 82.1) | 1 (1 to 1) | 2.5 (1.7 to 3) | 1 (1 to 1) | 2.7 (1.8 to 3.2) | 16 (11 to 20) | 50.6 (34.3 to 60.2) | 0 (0 to 0) | 0.6 (0.3 to 0.8) | 17 (11 to 20) | 51.2 (34.7 to 60.8) |
|  | Male | 0 (0 to 1) | 3 (1.2 to 4.1) | 0 (0 to 1) | 3.2 (1.3 to 4.4) | 9 (5 to 12) | 58.2 (25.2 to 78.2) | 0 (0 to 0) | 0.7 (0.3 to 1) | 10 (5 to 12) | 58.8 (25.5 to 79) | 1 (0 to 1) | 2.3 (1.1 to 2.8) | 1 (0 to 1) | 2.4 (1.2 to 2.9) | 16 (8 to 20) | 42.5 (21.9 to 52.6) | 0 (0 to 0) | 0.5 (0.2 to 0.7) | 16 (8 to 20) | 43 (22.2 to 53.3) |
| Grenada | Both | 4 (2 to 4) | 5.1 (3.3 to 5.5) | 4 (2 to 4) | 5.3 (3.5 to 5.8) | 78 (51 to 85) | 113.7 (73.8 to 123.3) | 1 (0 to 1) | 1.1 (0.6 to 1.5) | 79 (51 to 86) | 114.8 (74.5 to 124.6) | 2 (2 to 3) | 1.4 (1.3 to 1.9) | 2 (2 to 3) | 1.5 (1.3 to 2) | 45 (41 to 59) | 31.6 (28.6 to 41.3) | 0 (0 to 1) | 0.3 (0.2 to 0.4) | 46 (41 to 60) | 31.9 (28.9 to 41.7) |
|  | Female | 2 (2 to 3) | 5.7 (3.7 to 6.3) | 3 (2 to 3) | 5.9 (3.9 to 6.6) | 50 (33 to 56) | 129.8 (85.2 to 143.4) | 0 (0 to 1) | 1.2 (0.7 to 1.6) | 51 (33 to 56) | 131 (86 to 144.7) | 1 (1 to 2) | 1.4 (1.2 to 2.2) | 1 (1 to 2) | 1.5 (1.3 to 2.4) | 23 (20 to 35) | 31.1 (27.1 to 48.6) | 0 (0 to 0) | 0.3 (0.2 to 0.5) | 23 (20 to 36) | 31.4 (27.3 to 49) |
|  | Male | 1 (1 to 1) | 4.3 (2 to 4.8) | 1 (1 to 1) | 4.6 (2.2 to 5.1) | 28 (13 to 31) | 94 (45.1 to 106.2) | 0 (0 to 0) | 0.9 (0.4 to 1.3) | 28 (14 to 32) | 94.9 (45.5 to 107.3) | 1 (1 to 1) | 1.4 (1.2 to 1.6) | 1 (1 to 1) | 1.4 (1.3 to 1.7) | 23 (20 to 27) | 31.9 (28.2 to 37.6) | 0 (0 to 0) | 0.3 (0.2 to 0.4) | 23 (20 to 27) | 32.2 (28.5 to 38) |
| Guam | Both | 1 (1 to 1) | 1.2 (0.9 to 1.4) | 1 (1 to 1) | 1.2 (0.9 to 1.4) | 20 (15 to 23) | 23.8 (17.5 to 27.6) | 0 (0 to 0) | 0.3 (0.2 to 0.4) | 20 (15 to 23) | 24.1 (17.7 to 27.9) | 2 (1 to 2) | 0.9 (0.8 to 1.1) | 1 (1 to 2) | 0.8 (0.7 to 1) | 33 (28 to 40) | 17.9 (15.3 to 21.8) | 0 (0 to 1) | 0.2 (0.1 to 0.3) | 33 (28 to 41) | 18.1 (15.5 to 22.1) |
|  | Female | 0 (0 to 1) | 1.3 (0.9 to 1.6) | 0 (0 to 1) | 1.2 (0.8 to 1.5) | 11 (8 to 13) | 25.7 (17.9 to 31) | 0 (0 to 0) | 0.3 (0.2 to 0.4) | 11 (8 to 13) | 26 (18.2 to 31.3) | 1 (1 to 1) | 0.9 (0.7 to 1.2) | 1 (1 to 1) | 0.8 (0.7 to 1.1) | 17 (14 to 22) | 17.9 (14.7 to 23.7) | 0 (0 to 0) | 0.2 (0.1 to 0.3) | 17 (14 to 22) | 18.1 (15 to 23.9) |
|  | Male | 0 (0 to 1) | 1.1 (0.8 to 1.4) | 0 (0 to 0) | 1.2 (0.8 to 1.5) | 9 (6 to 12) | 22.4 (15.3 to 28.2) | 0 (0 to 0) | 0.3 (0.2 to 0.4) | 9 (6 to 12) | 22.7 (15.4 to 28.6) | 1 (1 to 1) | 0.9 (0.7 to 1.2) | 1 (1 to 1) | 0.9 (0.7 to 1.1) | 16 (13 to 21) | 17.9 (13.6 to 22.8) | 0 (0 to 0) | 0.2 (0.1 to 0.3) | 17 (13 to 21) | 18.1 (13.7 to 23) |
| Guatemala | Both | 170 (159 to 180) | 4.8 (4.5 to 5.1) | 175 (163 to 184) | 5.2 (4.8 to 5.5) | 4325 (3984 to 4573) | 107.5 (99.9 to 113.8) | 37 (25 to 50) | 1 (0.7 to 1.4) | 4362 (4016 to 4610) | 108.5 (100.6 to 114.7) | 184 (160 to 261) | 1.7 (1.5 to 2.5) | 193 (168 to 272) | 1.9 (1.6 to 2.6) | 4330 (3750 to 6222) | 38.7 (33.5 to 55.7) | 41 (27 to 61) | 0.4 (0.3 to 0.6) | 4371 (3782 to 6275) | 39 (33.8 to 56.3) |
|  | Female | 122 (114 to 131) | 6.7 (6.2 to 7.2) | 125 (116 to 134) | 7.2 (6.6 to 7.7) | 3133 (2903 to 3353) | 153.2 (141.8 to 163.5) | 26 (18 to 36) | 1.4 (0.9 to 1.9) | 3160 (2921 to 3387) | 154.6 (143 to 165.2) | 124 (103 to 179) | 2.1 (1.8 to 3.1) | 130 (109 to 187) | 2.3 (1.9 to 3.2) | 2963 (2465 to 4328) | 48.6 (40.5 to 70.9) | 28 (18 to 40) | 0.5 (0.3 to 0.7) | 2991 (2485 to 4375) | 49.1 (40.9 to 71.8) |
|  | Male | 48 (39 to 52) | 2.8 (2.3 to 3) | 50 (40 to 54) | 3.1 (2.5 to 3.3) | 1192 (955 to 1294) | 60.9 (49.5 to 66.1) | 11 (7 to 14) | 0.6 (0.4 to 0.8) | 1203 (963 to 1306) | 61.5 (49.9 to 66.7) | 60 (50 to 95) | 1.3 (1.1 to 2) | 63 (52 to 100) | 1.4 (1.1 to 2.2) | 1367 (1139 to 2130) | 26.6 (22.2 to 42.3) | 13 (9 to 21) | 0.3 (0.2 to 0.4) | 1380 (1150 to 2151) | 26.9 (22.4 to 42.8) |
| Guinea | Both | 54 (45 to 67) | 1.6 (1.4 to 2) | 57 (48 to 71) | 1.8 (1.5 to 2.2) | 1224 (1024 to 1542) | 35.3 (29.6 to 44.3) | 12 (8 to 17) | 0.4 (0.2 to 0.5) | 1236 (1033 to 1560) | 35.7 (29.9 to 44.7) | 71 (57 to 94) | 1.4 (1.2 to 1.9) | 76 (61 to 100) | 1.6 (1.3 to 2) | 1649 (1309 to 2223) | 30.4 (24.3 to 40.6) | 16 (10 to 23) | 0.3 (0.2 to 0.5) | 1665 (1322 to 2242) | 30.7 (24.6 to 41) |
|  | Female | 32 (25 to 43) | 2 (1.6 to 2.6) | 34 (26 to 45) | 2.2 (1.7 to 2.9) | 727 (569 to 1010) | 42.3 (33.3 to 58.1) | 7 (5 to 10) | 0.4 (0.3 to 0.6) | 734 (575 to 1019) | 42.7 (33.6 to 58.8) | 40 (30 to 57) | 1.6 (1.2 to 2.3) | 43 (31 to 61) | 1.8 (1.3 to 2.6) | 920 (681 to 1325) | 34.8 (25.8 to 50) | 9 (5 to 14) | 0.4 (0.2 to 0.6) | 929 (688 to 1339) | 35.1 (26.1 to 50.5) |
|  | Male | 22 (16 to 29) | 1.3 (1 to 1.7) | 23 (17 to 30) | 1.4 (1.1 to 1.9) | 496 (376 to 653) | 28.6 (21.6 to 37.4) | 5 (3 to 7) | 0.3 (0.2 to 0.4) | 501 (380 to 659) | 28.9 (21.8 to 37.8) | 31 (23 to 47) | 1.2 (0.9 to 1.8) | 33 (24 to 49) | 1.3 (1 to 1.9) | 729 (541 to 1099) | 26.2 (19.5 to 39.3) | 7 (4 to 11) | 0.3 (0.2 to 0.4) | 736 (545 to 1108) | 26.5 (19.7 to 39.7) |
| Guinea-Bissau | Both | 7 (5 to 10) | 1.9 (1.4 to 2.7) | 7 (5 to 10) | 2 (1.5 to 2.9) | 173 (122 to 253) | 41.4 (29.7 to 59.3) | 2 (1 to 2) | 0.4 (0.3 to 0.6) | 175 (123 to 256) | 41.8 (29.9 to 59.8) | 9 (7 to 13) | 1.5 (1.1 to 2.1) | 9 (7 to 13) | 1.6 (1.2 to 2.2) | 231 (170 to 331) | 32.4 (24.3 to 45.5) | 2 (1 to 3) | 0.3 (0.2 to 0.5) | 233 (172 to 334) | 32.8 (24.6 to 46) |
|  | Female | 4 (3 to 6) | 2.1 (1.5 to 3) | 4 (3 to 6) | 2.2 (1.6 to 3.2) | 92 (63 to 137) | 44.1 (30.9 to 64.8) | 1 (1 to 1) | 0.5 (0.3 to 0.7) | 93 (64 to 138) | 44.6 (31.3 to 65.4) | 6 (4 to 8) | 1.7 (1.2 to 2.3) | 6 (4 to 8) | 1.9 (1.3 to 2.6) | 136 (96 to 187) | 36.2 (25.7 to 49.9) | 1 (1 to 2) | 0.4 (0.2 to 0.6) | 138 (97 to 188) | 36.6 (26 to 50.4) |
|  | Male | 3 (2 to 5) | 1.7 (1 to 2.8) | 3 (2 to 5) | 1.9 (1.1 to 2.9) | 81 (45 to 136) | 38.7 (21.9 to 63.2) | 1 (0 to 1) | 0.4 (0.2 to 0.7) | 82 (46 to 137) | 39.1 (22.2 to 63.9) | 4 (2 to 6) | 1.2 (0.8 to 2) | 4 (2 to 6) | 1.3 (0.9 to 2.1) | 95 (60 to 162) | 27.8 (18.3 to 45.8) | 1 (0 to 1) | 0.3 (0.2 to 0.5) | 95 (61 to 163) | 28 (18.5 to 46.3) |
| Guyana | Both | 12 (9 to 13) | 3.2 (2.4 to 3.4) | 13 (9 to 14) | 3.4 (2.5 to 3.6) | 313 (229 to 337) | 74.8 (54.8 to 80.5) | 3 (2 to 4) | 0.7 (0.5 to 1) | 316 (231 to 340) | 75.5 (55.3 to 81.3) | 8 (7 to 10) | 1.3 (1.1 to 1.6) | 8 (7 to 10) | 1.3 (1.1 to 1.7) | 193 (166 to 248) | 29.5 (25.2 to 37.8) | 2 (1 to 2) | 0.3 (0.2 to 0.4) | 195 (168 to 250) | 29.7 (25.5 to 38.3) |
|  | Female | 7 (6 to 8) | 3.6 (2.9 to 4) | 7 (6 to 8) | 3.8 (3 to 4.2) | 184 (145 to 202) | 85.7 (67.6 to 94.3) | 2 (1 to 2) | 0.8 (0.5 to 1.1) | 186 (146 to 204) | 86.5 (68.2 to 95.2) | 5 (4 to 6) | 1.5 (1.3 to 1.9) | 5 (4 to 6) | 1.6 (1.3 to 2) | 124 (102 to 154) | 36.1 (29.8 to 44.9) | 1 (1 to 2) | 0.3 (0.2 to 0.5) | 125 (103 to 155) | 36.4 (30.1 to 45.3) |
|  | Male | 5 (3 to 6) | 2.7 (1.5 to 3.1) | 5 (3 to 6) | 2.9 (1.6 to 3.2) | 129 (69 to 143) | 63.3 (34.5 to 70.6) | 1 (1 to 2) | 0.6 (0.3 to 0.8) | 130 (70 to 145) | 63.9 (34.8 to 71.4) | 3 (2 to 4) | 1 (0.8 to 1.4) | 3 (2 to 4) | 1 (0.8 to 1.5) | 69 (57 to 96) | 22.2 (18.3 to 30.8) | 1 (0 to 1) | 0.2 (0.1 to 0.3) | 70 (57 to 97) | 22.4 (18.5 to 31.1) |
| Haiti | Both | 115 (75 to 152) | 3.7 (2.4 to 4.7) | 117 (76 to 152) | 3.9 (2.6 to 4.9) | 2987 (1905 to 4012) | 85 (54.9 to 111.8) | 25 (15 to 37) | 0.8 (0.5 to 1.2) | 3012 (1918 to 4042) | 85.8 (55.4 to 112.9) | 139 (99 to 203) | 2.2 (1.6 to 3.2) | 144 (103 to 209) | 2.4 (1.7 to 3.4) | 3505 (2459 to 5125) | 49.5 (35.2 to 72.2) | 31 (19 to 46) | 0.5 (0.3 to 0.7) | 3536 (2483 to 5167) | 50 (35.6 to 72.8) |
|  | Female | 69 (40 to 97) | 4.3 (2.6 to 5.8) | 70 (41 to 97) | 4.6 (2.7 to 6.1) | 1796 (1034 to 2610) | 100.7 (59 to 142.4) | 15 (8 to 23) | 0.9 (0.5 to 1.4) | 1811 (1041 to 2630) | 101.6 (59.4 to 143.7) | 80 (50 to 120) | 2.4 (1.5 to 3.5) | 83 (53 to 124) | 2.6 (1.7 to 3.8) | 2054 (1268 to 3038) | 54.1 (33.7 to 80.5) | 18 (10 to 28) | 0.5 (0.3 to 0.8) | 2072 (1279 to 3063) | 54.6 (34.1 to 81.5) |
|  | Male | 46 (26 to 64) | 3 (1.8 to 4.1) | 47 (27 to 65) | 3.3 (1.9 to 4.4) | 1191 (666 to 1681) | 68.9 (39.3 to 95.3) | 10 (5 to 17) | 0.7 (0.3 to 1.1) | 1201 (672 to 1697) | 69.6 (39.7 to 96.3) | 59 (37 to 99) | 2 (1.3 to 3.3) | 61 (40 to 101) | 2.2 (1.5 to 3.6) | 1452 (922 to 2492) | 44.4 (28.8 to 75.1) | 13 (7 to 22) | 0.4 (0.3 to 0.8) | 1465 (932 to 2512) | 44.9 (29.1 to 75.9) |
| High-income Asia Pacific | Both | 18851 (17452 to 19494) | 9.5 (8.9 to 9.8) | 15518 (14350 to 15861) | 7.8 (7.3 to 8) | 298557 (271963 to 306934) | 143.3 (130.9 to 147.2) | 4153 (2943 to 5333) | 2.1 (1.5 to 2.6) | 302710 (275539 to 311497) | 145.4 (132.5 to 149.5) | 43936 (35907 to 53057) | 8.7 (7.3 to 10.3) | 27063 (25357 to 28309) | 5.2 (5 to 5.5) | 364371 (346797 to 386524) | 84 (80 to 91) | 10023 (6978 to 13606) | 2.1 (1.5 to 2.8) | 374394 (355972 to 396418) | 86 (81.8 to 93) |
|  | Female | 10555 (10137 to 10982) | 9.1 (8.7 to 9.4) | 8583 (8329 to 8769) | 7.4 (7.1 to 7.5) | 156284 (150365 to 159969) | 133.3 (128.1 to 136.6) | 2299 (1632 to 2997) | 2 (1.4 to 2.6) | 158583 (152507 to 162421) | 135.3 (129.9 to 138.6) | 21762 (16296 to 29259) | 6.8 (5.4 to 8.7) | 13601 (12996 to 14322) | 4.2 (4 to 4.4) | 160576 (152644 to 170773) | 64.9 (61.4 to 69.5) | 4770 (3093 to 6828) | 1.6 (1.1 to 2.2) | 165346 (156870 to 175944) | 66.5 (62.9 to 71.3) |
|  | Male | 8296 (7005 to 8693) | 10.1 (8.6 to 10.5) | 6935 (5911 to 7216) | 8.5 (7.4 to 8.9) | 142273 (118649 to 149354) | 156.4 (131.5 to 163.4) | 1854 (1290 to 2441) | 2.2 (1.5 to 2.9) | 144127 (119883 to 151205) | 158.6 (132.9 to 165.7) | 22173 (17576 to 28250) | 11 (8.8 to 14) | 13462 (11779 to 14422) | 6.6 (5.8 to 7) | 203794 (187560 to 223442) | 106.2 (98.1 to 116.9) | 5253 (3528 to 7354) | 2.7 (1.8 to 3.7) | 209047 (191799 to 228821) | 108.9 (100.4 to 119.6) |
| High-income North America | Both | 8899 (8217 to 9452) | 2.4 (2.2 to 2.6) | 5310 (4921 to 5422) | 1.4 (1.3 to 1.5) | 96624 (89258 to 98536) | 27.4 (25.3 to 28) | 2350 (1646 to 3066) | 0.6 (0.4 to 0.8) | 98974 (91318 to 101233) | 28 (25.9 to 28.7) | 13031 (12202 to 14482) | 2.1 (2 to 2.3) | 6016 (5805 to 6571) | 1 (0.9 to 1) | 106725 (102830 to 117278) | 18.1 (17.5 to 19.9) | 3724 (2640 to 4905) | 0.6 (0.4 to 0.8) | 110450 (106330 to 121863) | 18.7 (18 to 20.6) |
|  | Female | 5210 (4852 to 5640) | 2.4 (2.2 to 2.6) | 3329 (3257 to 3419) | 1.5 (1.5 to 1.5) | 58176 (57083 to 59769) | 29.4 (28.8 to 30.2) | 1322 (931 to 1741) | 0.6 (0.4 to 0.8) | 59498 (58231 to 61179) | 30 (29.3 to 30.8) | 7024 (6449 to 7795) | 2 (1.9 to 2.2) | 3547 (3392 to 3735) | 1 (1 to 1.1) | 61175 (58527 to 64271) | 19.4 (18.6 to 20.4) | 1926 (1355 to 2594) | 0.6 (0.4 to 0.8) | 63101 (60274 to 66298) | 20 (19.1 to 21.1) |
|  | Male | 3689 (3039 to 4063) | 2.4 (2 to 2.7) | 1981 (1641 to 2035) | 1.3 (1.1 to 1.4) | 38448 (32511 to 39551) | 25.1 (21.2 to 25.8) | 1028 (702 to 1361) | 0.7 (0.5 to 0.9) | 39476 (33286 to 40653) | 25.7 (21.7 to 26.5) | 6007 (5466 to 7134) | 2.1 (2 to 2.5) | 2468 (2346 to 2910) | 0.9 (0.8 to 1) | 45551 (43280 to 53666) | 16.6 (15.8 to 19.6) | 1798 (1256 to 2408) | 0.6 (0.5 to 0.9) | 47349 (44957 to 55411) | 17.3 (16.4 to 20.2) |
| High-middle SDI | Both | 20568 (19570 to 22925) | 2.3 (2.2 to 2.5) | 20833 (19847 to 23441) | 2.4 (2.3 to 2.7) | 470303 (445230 to 520713) | 48.7 (46.2 to 54.1) | 4497 (3110 to 5904) | 0.5 (0.3 to 0.6) | 474800 (449610 to 525716) | 49.2 (46.7 to 54.6) | 36134 (29533 to 39014) | 2.1 (1.7 to 2.2) | 32822 (27483 to 34721) | 1.9 (1.6 to 2) | 673030 (562480 to 712261) | 37 (31 to 39.2) | 8342 (5720 to 11167) | 0.5 (0.3 to 0.6) | 681373 (567998 to 722364) | 37.5 (31.3 to 39.7) |
|  | Female | 13291 (12699 to 14637) | 2.6 (2.5 to 2.8) | 13554 (13027 to 15006) | 2.7 (2.6 to 2.9) | 295525 (276938 to 322982) | 55.5 (52.1 to 60.7) | 2870 (1975 to 3757) | 0.6 (0.4 to 0.7) | 298395 (280095 to 325848) | 56.1 (52.7 to 61.3) | 21131 (17209 to 23196) | 2.2 (1.8 to 2.4) | 19352 (16022 to 20690) | 2 (1.6 to 2.1) | 384258 (317782 to 411495) | 39.5 (32.7 to 42.3) | 4797 (3262 to 6432) | 0.5 (0.3 to 0.7) | 389054 (321230 to 417179) | 40 (33.1 to 42.9) |
|  | Male | 7277 (6459 to 8835) | 1.9 (1.7 to 2.3) | 7279 (6528 to 8874) | 2 (1.8 to 2.4) | 174778 (152862 to 209776) | 40.4 (35.8 to 48.8) | 1626 (1110 to 2215) | 0.4 (0.3 to 0.6) | 176405 (154196 to 211717) | 40.8 (36.1 to 49.2) | 15003 (11351 to 16821) | 1.9 (1.4 to 2.1) | 13471 (10442 to 14800) | 1.8 (1.4 to 1.9) | 288772 (222437 to 318852) | 34.3 (26.5 to 37.8) | 3546 (2320 to 4870) | 0.4 (0.3 to 0.6) | 292318 (225280 to 322954) | 34.8 (26.9 to 38.3) |
| High SDI | Both | 63137 (57700 to 65299) | 4.8 (4.4 to 5) | 47707 (43691 to 48436) | 3.6 (3.3 to 3.7) | 881269 (798238 to 896781) | 68.7 (62.1 to 70) | 14813 (10491 to 19290) | 1.1 (0.8 to 1.5) | 896082 (813112 to 913151) | 69.9 (63.3 to 71.2) | 94448 (84596 to 104546) | 4 (3.6 to 4.4) | 60103 (55811 to 62705) | 2.5 (2.3 to 2.6) | 905308 (848878 to 948436) | 42 (39.6 to 43.9) | 22814 (16346 to 29922) | 1 (0.7 to 1.3) | 928122 (871799 to 970044) | 43 (40.6 to 44.9) |
|  | Female | 38766 (37224 to 40411) | 5 (4.8 to 5.2) | 29787 (29208 to 30280) | 3.8 (3.7 to 3.8) | 522424 (511393 to 531236) | 71.7 (70.2 to 72.9) | 8878 (6287 to 11468) | 1.2 (0.8 to 1.5) | 531302 (519696 to 540601) | 72.9 (71.2 to 74.2) | 49685 (43067 to 57742) | 3.6 (3.2 to 4) | 32719 (31498 to 34279) | 2.3 (2.2 to 2.4) | 458483 (439710 to 482376) | 38.8 (37.1 to 40.7) | 11606 (8101 to 15388) | 0.9 (0.6 to 1.1) | 470088 (451126 to 494424) | 39.6 (37.9 to 41.7) |
|  | Male | 24371 (19846 to 25629) | 4.6 (3.7 to 4.8) | 17919 (14654 to 18368) | 3.4 (2.8 to 3.5) | 358844 (292019 to 369759) | 64.9 (52.8 to 66.9) | 5935 (4119 to 7789) | 1.1 (0.8 to 1.4) | 364780 (296378 to 375854) | 66 (53.6 to 68) | 44763 (38189 to 51834) | 4.4 (3.8 to 5.1) | 27384 (23340 to 29093) | 2.7 (2.3 to 2.9) | 446825 (391939 to 477385) | 45.8 (40.3 to 49) | 11209 (7828 to 15121) | 1.1 (0.8 to 1.5) | 458034 (400916 to 488738) | 46.9 (41.2 to 50.1) |
| Honduras | Both | 64 (54 to 88) | 3 (2.5 to 4.3) | 65 (54 to 90) | 3.2 (2.7 to 4.5) | 1647 (1388 to 2121) | 71.1 (59.9 to 95.7) | 14 (10 to 20) | 0.7 (0.4 to 1) | 1662 (1400 to 2143) | 71.8 (60.5 to 96.6) | 210 (154 to 294) | 3.5 (2.6 to 4.9) | 209 (155 to 294) | 3.6 (2.7 to 5.1) | 5100 (3731 to 7158) | 80.5 (59.1 to 113.9) | 46 (28 to 70) | 0.8 (0.5 to 1.2) | 5146 (3756 to 7225) | 81.2 (59.7 to 114.8) |
|  | Female | 48 (39 to 69) | 4.4 (3.6 to 6.6) | 48 (39 to 71) | 4.6 (3.7 to 7.1) | 1249 (1017 to 1710) | 105.6 (85.5 to 151.5) | 11 (7 to 16) | 1 (0.6 to 1.5) | 1260 (1024 to 1725) | 106.5 (86.2 to 152.9) | 164 (113 to 239) | 5.2 (3.6 to 7.7) | 161 (111 to 242) | 5.3 (3.7 to 8.1) | 4038 (2782 to 5915) | 121.1 (83.7 to 177.1) | 36 (21 to 57) | 1.1 (0.7 to 1.8) | 4074 (2809 to 5952) | 122.2 (84.4 to 178.6) |
|  | Male | 16 (13 to 22) | 1.6 (1.3 to 2.2) | 17 (13 to 23) | 1.7 (1.3 to 2.4) | 398 (304 to 533) | 35.6 (27.6 to 48.7) | 4 (2 to 5) | 0.4 (0.2 to 0.5) | 402 (307 to 537) | 35.9 (28 to 49.1) | 46 (32 to 68) | 1.6 (1.1 to 2.4) | 48 (34 to 71) | 1.7 (1.2 to 2.5) | 1061 (707 to 1593) | 35.9 (24.1 to 53.2) | 10 (6 to 16) | 0.4 (0.2 to 0.6) | 1072 (713 to 1606) | 36.3 (24.3 to 53.7) |
| Hungary | Both | 1047 (992 to 1127) | 6.9 (6.5 to 7.4) | 1111 (1061 to 1159) | 7.3 (7 to 7.6) | 21187 (20333 to 22180) | 138.2 (132.7 to 144.3) | 209 (140 to 283) | 1.4 (0.9 to 1.9) | 21396 (20539 to 22386) | 139.6 (134 to 145.8) | 653 (600 to 711) | 3.3 (3 to 3.6) | 720 (665 to 778) | 3.6 (3.3 to 3.9) | 12692 (11723 to 13721) | 67.8 (62.5 to 73.5) | 138 (94 to 184) | 0.7 (0.5 to 0.9) | 12830 (11871 to 13851) | 68.5 (63.2 to 74.2) |
|  | Female | 787 (716 to 863) | 8.5 (7.9 to 9.4) | 831 (763 to 872) | 8.9 (8.3 to 9.3) | 15361 (14422 to 16119) | 170.6 (160.5 to 178.8) | 154 (101 to 216) | 1.7 (1.1 to 2.3) | 15515 (14588 to 16278) | 172.3 (162.3 to 180.8) | 423 (379 to 477) | 3.5 (3.1 to 3.9) | 469 (421 to 523) | 3.7 (3.3 to 4.2) | 7815 (6946 to 8868) | 70.7 (62.6 to 80.8) | 88 (60 to 121) | 0.7 (0.5 to 1) | 7904 (7036 to 8970) | 71.4 (63.4 to 81.5) |
|  | Male | 260 (243 to 293) | 4.3 (4 to 4.8) | 280 (261 to 313) | 4.7 (4.4 to 5.2) | 5825 (5433 to 6650) | 92.1 (85.9 to 104.4) | 55 (37 to 75) | 0.9 (0.6 to 1.2) | 5880 (5487 to 6712) | 93 (86.7 to 105.5) | 229 (203 to 256) | 3 (2.7 to 3.3) | 251 (222 to 278) | 3.3 (2.9 to 3.7) | 4877 (4342 to 5466) | 63.6 (56.5 to 71.1) | 50 (34 to 67) | 0.7 (0.4 to 0.9) | 4927 (4382 to 5520) | 64.3 (57.2 to 71.9) |
| Iceland | Both | 10 (8 to 13) | 3.5 (2.8 to 4.5) | 7 (6 to 7) | 2.2 (2 to 2.3) | 117 (108 to 126) | 40.8 (38 to 43.9) | 3 (2 to 4) | 0.9 (0.6 to 1.3) | 120 (111 to 128) | 41.7 (38.6 to 44.8) | 10 (8 to 13) | 1.8 (1.5 to 2.3) | 7 (7 to 8) | 1.3 (1.2 to 1.4) | 116 (106 to 128) | 22.5 (20.5 to 24.8) | 2 (2 to 3) | 0.5 (0.3 to 0.7) | 119 (108 to 131) | 22.9 (21 to 25.3) |
|  | Female | 5 (4 to 8) | 3.4 (2.6 to 4.6) | 4 (3 to 4) | 2.3 (2.1 to 2.5) | 66 (60 to 72) | 43.4 (39 to 47.8) | 1 (1 to 2) | 0.8 (0.5 to 1.3) | 67 (61 to 74) | 44.2 (39.9 to 48.6) | 4 (3 to 6) | 1.4 (1.2 to 1.9) | 3 (3 to 4) | 1.1 (1 to 1.3) | 53 (48 to 60) | 19.8 (17.6 to 22.2) | 1 (1 to 2) | 0.4 (0.2 to 0.5) | 55 (49 to 61) | 20.2 (17.9 to 22.6) |
|  | Male | 5 (4 to 7) | 3.6 (2.7 to 5.4) | 3 (2 to 3) | 2.1 (1.8 to 2.3) | 51 (44 to 57) | 38.2 (33.3 to 42.5) | 1 (1 to 2) | 1 (0.6 to 1.6) | 52 (45 to 58) | 39.1 (34.2 to 43.7) | 6 (4 to 8) | 2.2 (1.7 to 3.2) | 4 (3 to 4) | 1.4 (1.3 to 1.6) | 63 (56 to 72) | 25.3 (22.6 to 29.1) | 1 (1 to 2) | 0.6 (0.4 to 0.9) | 64 (57 to 74) | 25.9 (23.1 to 29.7) |
| India | Both | 9390 (7940 to 13581) | 2.1 (1.8 to 3) | 9472 (8030 to 13658) | 2.2 (1.9 to 3.2) | 246749 (207741 to 354211) | 47.6 (40.2 to 68.6) | 2098 (1394 to 3201) | 0.5 (0.3 to 0.7) | 248847 (209482 to 357174) | 48 (40.7 to 69.4) | 29178 (21906 to 32264) | 2.8 (2.1 to 3.1) | 30239 (22684 to 33444) | 3 (2.2 to 3.3) | 719943 (535648 to 798041) | 63.2 (47.1 to 69.9) | 6450 (4219 to 8685) | 0.6 (0.4 to 0.8) | 726393 (540049 to 806372) | 63.8 (47.6 to 70.6) |
|  | Female | 5401 (4547 to 8015) | 2.4 (2.1 to 3.6) | 5449 (4604 to 8086) | 2.6 (2.2 to 3.8) | 141887 (118975 to 206177) | 56.4 (47.5 to 83.3) | 1202 (784 to 1857) | 0.5 (0.3 to 0.8) | 143089 (120102 to 207659) | 56.9 (48.2 to 84.2) | 18827 (13062 to 21382) | 3.4 (2.4 to 3.9) | 19536 (13569 to 22127) | 3.6 (2.5 to 4.1) | 465845 (316441 to 529171) | 79.9 (54.7 to 90.8) | 4133 (2566 to 5659) | 0.7 (0.5 to 1) | 469978 (319257 to 534464) | 80.6 (55.3 to 91.6) |
|  | Male | 3989 (3157 to 6314) | 1.7 (1.4 to 2.7) | 4023 (3191 to 6319) | 1.9 (1.5 to 2.9) | 104862 (82721 to 165431) | 39.4 (31.3 to 61.8) | 896 (576 to 1420) | 0.4 (0.2 to 0.6) | 105758 (83389 to 166679) | 39.8 (31.6 to 62.4) | 10351 (7305 to 12703) | 2.1 (1.5 to 2.5) | 10702 (7571 to 13077) | 2.2 (1.6 to 2.7) | 254098 (178489 to 310727) | 45.9 (32.4 to 56.1) | 2316 (1461 to 3206) | 0.5 (0.3 to 0.6) | 256415 (179990 to 313136) | 46.4 (32.8 to 56.7) |
| Indonesia | Both | 1479 (1201 to 1748) | 1.6 (1.3 to 1.9) | 1494 (1219 to 1761) | 1.7 (1.4 to 2) | 37943 (30812 to 44765) | 35.7 (29 to 42.2) | 332 (218 to 456) | 0.4 (0.2 to 0.5) | 38274 (31092 to 45173) | 36.1 (29.3 to 42.6) | 2898 (2450 to 3519) | 1.5 (1.3 to 1.8) | 2975 (2519 to 3597) | 1.6 (1.4 to 1.9) | 67576 (56693 to 82699) | 31.2 (26.3 to 37.9) | 650 (436 to 893) | 0.3 (0.2 to 0.5) | 68225 (57297 to 83486) | 31.5 (26.5 to 38.3) |
|  | Female | 858 (607 to 1056) | 1.8 (1.3 to 2.1) | 865 (621 to 1055) | 1.9 (1.4 to 2.2) | 21891 (15144 to 27463) | 39.7 (28.2 to 48.9) | 192 (118 to 278) | 0.4 (0.2 to 0.6) | 22083 (15264 to 27730) | 40.1 (28.5 to 49.3) | 1569 (1193 to 1885) | 1.5 (1.2 to 1.8) | 1617 (1238 to 1943) | 1.6 (1.2 to 2) | 35852 (27003 to 43191) | 31.6 (24 to 37.9) | 350 (219 to 486) | 0.3 (0.2 to 0.5) | 36203 (27294 to 43605) | 31.9 (24.2 to 38.3) |
|  | Male | 622 (464 to 832) | 1.4 (1.1 to 1.9) | 629 (469 to 842) | 1.5 (1.2 to 2) | 16051 (11977 to 21429) | 31.4 (23.5 to 42.1) | 140 (89 to 196) | 0.3 (0.2 to 0.4) | 16191 (12096 to 21655) | 31.7 (23.8 to 42.6) | 1329 (1053 to 1809) | 1.5 (1.2 to 2) | 1358 (1079 to 1855) | 1.6 (1.3 to 2.2) | 31723 (24967 to 42954) | 30.8 (24.4 to 42) | 299 (187 to 440) | 0.3 (0.2 to 0.5) | 32023 (25184 to 43387) | 31.2 (24.7 to 42.4) |
| Iran | Both | 256 (212 to 329) | 1 (0.9 to 1.3) | 256 (212 to 327) | 1.1 (0.9 to 1.4) | 6447 (5280 to 8206) | 22.6 (18.7 to 28.8) | 58 (39 to 79) | 0.2 (0.2 to 0.3) | 6504 (5321 to 8279) | 22.8 (18.8 to 29.1) | 721 (582 to 823) | 1.1 (0.9 to 1.2) | 729 (593 to 836) | 1.1 (0.9 to 1.3) | 15776 (12823 to 17867) | 21.9 (17.9 to 25) | 163 (107 to 221) | 0.2 (0.2 to 0.3) | 15939 (12924 to 18058) | 22.1 (18 to 25.2) |
|  | Female | 131 (103 to 170) | 1.1 (0.9 to 1.5) | 132 (104 to 171) | 1.2 (1 to 1.6) | 3234 (2548 to 4228) | 23.9 (18.9 to 31.3) | 29 (19 to 40) | 0.2 (0.2 to 0.3) | 3263 (2577 to 4266) | 24.2 (19.1 to 31.5) | 382 (313 to 458) | 1.2 (1 to 1.4) | 393 (319 to 482) | 1.2 (1 to 1.5) | 8285 (6746 to 9874) | 23.2 (18.9 to 27.9) | 85 (57 to 118) | 0.3 (0.2 to 0.4) | 8371 (6829 to 9973) | 23.4 (19.1 to 28.3) |
|  | Male | 125 (100 to 173) | 1 (0.8 to 1.3) | 124 (100 to 171) | 1 (0.8 to 1.4) | 3213 (2537 to 4434) | 21.1 (16.9 to 29.1) | 28 (18 to 39) | 0.2 (0.1 to 0.3) | 3241 (2551 to 4474) | 21.4 (17.1 to 29.4) | 339 (225 to 414) | 1 (0.7 to 1.2) | 336 (230 to 413) | 1 (0.7 to 1.3) | 7491 (5070 to 9056) | 20.6 (14.1 to 25) | 78 (47 to 109) | 0.2 (0.1 to 0.3) | 7569 (5130 to 9165) | 20.8 (14.2 to 25.2) |
| Iraq | Both | 82 (67 to 107) | 1.1 (0.9 to 1.4) | 84 (69 to 110) | 1.1 (0.9 to 1.5) | 2078 (1666 to 2717) | 24.9 (20.1 to 32) | 18 (12 to 26) | 0.2 (0.2 to 0.3) | 2096 (1679 to 2741) | 25.2 (20.3 to 32.3) | 120 (101 to 143) | 0.5 (0.4 to 0.6) | 121 (102 to 147) | 0.6 (0.5 to 0.7) | 3089 (2551 to 3620) | 12 (10.1 to 14.4) | 27 (18 to 37) | 0.1 (0.1 to 0.2) | 3116 (2576 to 3651) | 12.2 (10.2 to 14.5) |
|  | Female | 47 (36 to 61) | 1.2 (0.9 to 1.6) | 48 (37 to 63) | 1.3 (1 to 1.7) | 1169 (849 to 1586) | 28.1 (21.1 to 37.3) | 10 (7 to 15) | 0.3 (0.2 to 0.4) | 1179 (856 to 1599) | 28.4 (21.3 to 37.7) | 70 (56 to 84) | 0.6 (0.5 to 0.8) | 71 (57 to 87) | 0.7 (0.6 to 0.8) | 1793 (1418 to 2159) | 14.4 (11.5 to 17.3) | 16 (10 to 22) | 0.1 (0.1 to 0.2) | 1809 (1428 to 2176) | 14.6 (11.6 to 17.5) |
|  | Male | 35 (24 to 52) | 0.9 (0.7 to 1.4) | 36 (25 to 53) | 1 (0.7 to 1.5) | 909 (602 to 1329) | 21.7 (14.7 to 31.9) | 8 (5 to 13) | 0.2 (0.1 to 0.3) | 917 (608 to 1340) | 21.9 (14.8 to 32.2) | 50 (40 to 70) | 0.4 (0.3 to 0.6) | 50 (41 to 71) | 0.4 (0.4 to 0.6) | 1295 (1025 to 1762) | 9.8 (7.9 to 13.6) | 11 (7 to 16) | 0.1 (0.1 to 0.1) | 1307 (1035 to 1776) | 9.9 (8 to 13.7) |
| Ireland | Both | 123 (108 to 147) | 2.9 (2.6 to 3.5) | 102 (96 to 108) | 2.4 (2.3 to 2.5) | 1881 (1769 to 1990) | 44.8 (42 to 47.3) | 29 (20 to 40) | 0.7 (0.5 to 0.9) | 1911 (1793 to 2023) | 45.5 (42.7 to 48.1) | 150 (125 to 188) | 2 (1.7 to 2.6) | 118 (105 to 133) | 1.6 (1.4 to 1.8) | 1944 (1722 to 2193) | 27.2 (24 to 30.6) | 37 (24 to 51) | 0.5 (0.3 to 0.7) | 1981 (1750 to 2238) | 27.7 (24.5 to 31.2) |
|  | Female | 68 (59 to 83) | 2.9 (2.5 to 3.6) | 60 (56 to 64) | 2.5 (2.3 to 2.7) | 1073 (998 to 1148) | 47.3 (43.8 to 50.6) | 16 (11 to 22) | 0.7 (0.5 to 0.9) | 1089 (1010 to 1162) | 48 (44.4 to 51.3) | 77 (63 to 102) | 1.9 (1.6 to 2.5) | 66 (57 to 75) | 1.6 (1.4 to 1.8) | 1042 (903 to 1205) | 27.7 (24.1 to 32.2) | 18 (12 to 26) | 0.5 (0.3 to 0.7) | 1060 (920 to 1226) | 28.2 (24.5 to 32.7) |
|  | Male | 56 (45 to 73) | 3 (2.4 to 3.9) | 42 (37 to 46) | 2.3 (2.1 to 2.5) | 808 (716 to 887) | 42.2 (37.8 to 46.1) | 14 (9 to 20) | 0.7 (0.5 to 1.1) | 822 (728 to 900) | 43 (38.5 to 47.1) | 73 (55 to 103) | 2.2 (1.6 to 3.1) | 53 (43 to 63) | 1.6 (1.3 to 1.9) | 902 (734 to 1088) | 26.6 (21.8 to 31.9) | 19 (11 to 29) | 0.6 (0.3 to 0.8) | 920 (753 to 1114) | 27.2 (22.3 to 32.7) |
| Israel | Both | 163 (145 to 188) | 3.3 (3 to 3.8) | 148 (137 to 159) | 3 (2.8 to 3.2) | 2709 (2498 to 2893) | 54.5 (50.2 to 58.2) | 37 (25 to 51) | 0.8 (0.5 to 1) | 2747 (2535 to 2935) | 55.2 (51 to 59) | 151 (131 to 219) | 1.3 (1.1 to 1.9) | 142 (127 to 202) | 1.2 (1.1 to 1.7) | 2289 (2028 to 3357) | 20.9 (18.5 to 30.7) | 35 (24 to 53) | 0.3 (0.2 to 0.5) | 2324 (2060 to 3404) | 21.2 (18.8 to 31.2) |
|  | Female | 93 (82 to 110) | 3.5 (3.1 to 4.1) | 88 (81 to 96) | 3.3 (3 to 3.5) | 1604 (1467 to 1739) | 59.4 (54.4 to 64.4) | 21 (14 to 28) | 0.8 (0.5 to 1.1) | 1625 (1486 to 1761) | 60.2 (55 to 65.2) | 78 (66 to 105) | 1.2 (1 to 1.6) | 78 (68 to 101) | 1.2 (1 to 1.5) | 1180 (1021 to 1522) | 19.7 (17 to 25.5) | 18 (12 to 27) | 0.3 (0.2 to 0.4) | 1198 (1036 to 1552) | 19.9 (17.2 to 26) |
|  | Male | 69 (57 to 89) | 3.1 (2.5 to 4) | 60 (51 to 66) | 2.7 (2.3 to 3) | 1105 (955 to 1215) | 48.5 (41.9 to 53.2) | 17 (11 to 23) | 0.7 (0.5 to 1) | 1122 (969 to 1233) | 49.3 (42.5 to 54.1) | 73 (59 to 126) | 1.4 (1.2 to 2.5) | 64 (54 to 111) | 1.2 (1.1 to 2.1) | 1109 (928 to 1948) | 22.2 (18.6 to 38.8) | 18 (11 to 30) | 0.4 (0.2 to 0.6) | 1127 (943 to 1976) | 22.5 (18.9 to 39.3) |
| Italy | Both | 4031 (3589 to 4776) | 4.4 (3.9 to 5.2) | 3504 (3344 to 3652) | 3.8 (3.6 to 3.9) | 64396 (60812 to 66678) | 71.6 (67.5 to 74.1) | 904 (614 to 1222) | 1 (0.7 to 1.3) | 65301 (61801 to 67706) | 72.6 (68.5 to 75.2) | 5728 (4698 to 7326) | 3.7 (3.1 to 4.6) | 4307 (3778 to 4718) | 2.7 (2.3 to 2.9) | 63843 (55156 to 70040) | 47.4 (40.8 to 52) | 1316 (862 to 1860) | 0.9 (0.6 to 1.2) | 65159 (56375 to 71411) | 48.3 (41.7 to 53) |
|  | Female | 2592 (2229 to 3360) | 4.8 (4.2 to 6.1) | 2248 (2143 to 2346) | 4.1 (3.9 to 4.2) | 39537 (37574 to 41230) | 77.8 (74 to 81) | 570 (369 to 816) | 1.1 (0.7 to 1.5) | 40108 (38062 to 41825) | 78.9 (75 to 82.1) | 3101 (2423 to 4534) | 3.5 (2.8 to 4.6) | 2336 (2077 to 2614) | 2.5 (2.2 to 2.8) | 32779 (29059 to 36985) | 44.1 (39.1 to 50) | 703 (443 to 1054) | 0.8 (0.6 to 1.2) | 33482 (29715 to 37847) | 44.9 (39.8 to 50.9) |
|  | Male | 1439 (1255 to 1744) | 3.8 (3.3 to 4.7) | 1256 (1136 to 1331) | 3.3 (3.1 to 3.5) | 24859 (21896 to 26129) | 63.7 (56.3 to 66.9) | 334 (230 to 468) | 0.9 (0.6 to 1.2) | 25193 (22198 to 26426) | 64.6 (57.3 to 68) | 2626 (1832 to 3478) | 4.1 (2.9 to 5.4) | 1971 (1375 to 2248) | 3 (2.1 to 3.4) | 31064 (21304 to 35591) | 51.5 (35.5 to 59.1) | 613 (359 to 902) | 1 (0.6 to 1.4) | 31677 (21784 to 36351) | 52.5 (36.3 to 60.2) |
| Jamaica | Both | 42 (38 to 45) | 2.3 (2.1 to 2.5) | 44 (40 to 47) | 2.4 (2.2 to 2.6) | 885 (796 to 962) | 49.8 (44.9 to 54.3) | 9 (6 to 13) | 0.5 (0.4 to 0.7) | 894 (802 to 971) | 50.4 (45.2 to 54.8) | 42 (34 to 60) | 1.5 (1.2 to 2.1) | 43 (35 to 61) | 1.5 (1.2 to 2.1) | 946 (757 to 1317) | 32.9 (26.3 to 45.8) | 9 (6 to 14) | 0.3 (0.2 to 0.5) | 956 (766 to 1331) | 33.2 (26.6 to 46.2) |
|  | Female | 25 (23 to 28) | 2.6 (2.3 to 2.9) | 27 (24 to 29) | 2.7 (2.4 to 3) | 539 (477 to 599) | 57.9 (50.9 to 64.4) | 6 (4 to 8) | 0.6 (0.4 to 0.8) | 545 (482 to 605) | 58.5 (51.3 to 65) | 27 (21 to 35) | 1.8 (1.4 to 2.3) | 28 (22 to 36) | 1.9 (1.5 to 2.4) | 614 (481 to 784) | 41.4 (32.4 to 52.8) | 6 (4 to 9) | 0.4 (0.3 to 0.6) | 620 (485 to 792) | 41.8 (32.7 to 53.4) |
|  | Male | 17 (14 to 18) | 2 (1.7 to 2.2) | 17 (15 to 19) | 2.1 (1.8 to 2.3) | 345 (284 to 388) | 41.2 (33.9 to 46.1) | 4 (2 to 5) | 0.4 (0.3 to 0.6) | 349 (288 to 392) | 41.7 (34.4 to 46.7) | 15 (10 to 27) | 1.1 (0.7 to 2) | 15 (10 to 27) | 1.1 (0.7 to 2) | 333 (225 to 565) | 23.9 (16.2 to 40.9) | 3 (2 to 6) | 0.2 (0.1 to 0.4) | 336 (227 to 571) | 24.2 (16.3 to 41.3) |
| Japan | Both | 16205 (14930 to 16746) | 9.5 (8.9 to 9.9) | 12912 (11958 to 13170) | 7.6 (7.1 to 7.7) | 237441 (217406 to 241991) | 135.2 (124 to 137.8) | 3594 (2547 to 4663) | 2.1 (1.5 to 2.7) | 241034 (220564 to 245953) | 137.3 (125.9 to 140.1) | 36705 (29140 to 45681) | 8.9 (7.3 to 10.8) | 22033 (20435 to 22974) | 5.1 (4.8 to 5.3) | 277176 (264843 to 292053) | 80.3 (76.8 to 85.9) | 8366 (5742 to 11536) | 2.1 (1.5 to 2.9) | 285542 (271915 to 302391) | 82.5 (78.7 to 88.3) |
|  | Female | 9229 (8846 to 9623) | 9.3 (8.9 to 9.7) | 7252 (7123 to 7396) | 7.2 (7.1 to 7.4) | 127073 (124484 to 129935) | 128.6 (126 to 131.5) | 2022 (1441 to 2614) | 2 (1.5 to 2.6) | 129096 (126348 to 132013) | 130.6 (127.7 to 133.5) | 18477 (13272 to 25765) | 7 (5.4 to 9.1) | 11234 (10751 to 11837) | 4.1 (3.9 to 4.3) | 123372 (117297 to 131411) | 62 (58.7 to 66.5) | 4045 (2598 to 5928) | 1.6 (1.1 to 2.3) | 127417 (120991 to 135448) | 63.6 (60.2 to 68.4) |
|  | Male | 6975 (5911 to 7226) | 9.9 (8.5 to 10.2) | 5660 (4777 to 5792) | 8.1 (6.9 to 8.3) | 110367 (93110 to 113110) | 144.2 (121.8 to 147.7) | 1571 (1085 to 2063) | 2.2 (1.5 to 2.8) | 111938 (94301 to 114794) | 146.4 (123.5 to 150.1) | 18228 (14099 to 23924) | 11.2 (8.8 to 14.7) | 10799 (9155 to 11515) | 6.4 (5.5 to 6.8) | 153804 (140511 to 165345) | 101.6 (94.7 to 110.1) | 4321 (2861 to 6286) | 2.7 (1.8 to 4) | 158124 (143814 to 170014) | 104.4 (97.1 to 113.4) |
| Jordan | Both | 23 (18 to 30) | 1.7 (1.3 to 2.2) | 23 (17 to 30) | 1.8 (1.4 to 2.3) | 597 (449 to 769) | 38.8 (29 to 49.6) | 5 (3 to 8) | 0.4 (0.2 to 0.5) | 602 (453 to 777) | 39.1 (29.2 to 50.1) | 70 (54 to 85) | 1.3 (1 to 1.6) | 70 (54 to 86) | 1.4 (1.1 to 1.7) | 1627 (1270 to 1986) | 26.5 (20.5 to 32.6) | 16 (10 to 22) | 0.3 (0.2 to 0.4) | 1643 (1278 to 2002) | 26.8 (20.8 to 32.8) |
|  | Female | 15 (10 to 19) | 2.2 (1.5 to 2.7) | 15 (10 to 18) | 2.3 (1.6 to 2.9) | 372 (244 to 481) | 49.7 (33 to 63.4) | 3 (2 to 5) | 0.5 (0.3 to 0.7) | 376 (246 to 486) | 50.2 (33.3 to 64) | 41 (29 to 52) | 1.6 (1.2 to 2.1) | 41 (30 to 52) | 1.7 (1.3 to 2.2) | 945 (686 to 1206) | 32.6 (23.5 to 41.2) | 9 (6 to 13) | 0.4 (0.2 to 0.5) | 954 (692 to 1219) | 32.9 (23.8 to 41.8) |
|  | Male | 9 (5 to 14) | 1.3 (0.7 to 2) | 9 (5 to 14) | 1.3 (0.8 to 2.1) | 224 (130 to 367) | 28.2 (15.8 to 45.7) | 2 (1 to 3) | 0.3 (0.1 to 0.5) | 226 (131 to 370) | 28.5 (16 to 46.2) | 29 (20 to 38) | 1.1 (0.7 to 1.4) | 29 (20 to 37) | 1.1 (0.8 to 1.5) | 682 (490 to 882) | 20.9 (14.6 to 27.1) | 7 (4 to 10) | 0.2 (0.1 to 0.4) | 688 (495 to 890) | 21.2 (14.7 to 27.4) |
| Kazakhstan | Both | 183 (146 to 276) | 1.4 (1.1 to 2.2) | 189 (151 to 286) | 1.5 (1.2 to 2.3) | 4411 (3482 to 6597) | 32.7 (25.9 to 49) | 41 (25 to 67) | 0.3 (0.2 to 0.5) | 4452 (3516 to 6651) | 33 (26.2 to 49.4) | 178 (156 to 197) | 1.1 (0.9 to 1.2) | 186 (163 to 206) | 1.1 (1 to 1.3) | 4291 (3795 to 4750) | 24.4 (21.4 to 26.9) | 40 (26 to 54) | 0.2 (0.2 to 0.3) | 4331 (3830 to 4800) | 24.6 (21.7 to 27.2) |
|  | Female | 117 (96 to 206) | 1.5 (1.2 to 2.6) | 122 (100 to 214) | 1.5 (1.3 to 2.7) | 2707 (2222 to 4759) | 33.3 (27.3 to 58.6) | 26 (16 to 48) | 0.3 (0.2 to 0.6) | 2733 (2246 to 4805) | 33.6 (27.6 to 59.2) | 115 (100 to 130) | 1.2 (1 to 1.3) | 121 (105 to 137) | 1.2 (1.1 to 1.4) | 2681 (2327 to 3081) | 26.2 (22.7 to 29.9) | 26 (17 to 35) | 0.3 (0.2 to 0.3) | 2706 (2346 to 3108) | 26.4 (23 to 30.2) |
|  | Male | 66 (43 to 106) | 1.4 (0.9 to 2.2) | 67 (44 to 108) | 1.5 (1 to 2.3) | 1704 (1112 to 2766) | 32 (21 to 51.6) | 15 (8 to 25) | 0.3 (0.2 to 0.5) | 1719 (1122 to 2791) | 32.3 (21.2 to 52.1) | 63 (49 to 72) | 0.9 (0.7 to 1.1) | 65 (50 to 74) | 1 (0.8 to 1.1) | 1610 (1258 to 1844) | 21.8 (17 to 24.8) | 14 (9 to 20) | 0.2 (0.1 to 0.3) | 1624 (1268 to 1865) | 22 (17.1 to 25.1) |
| Kenya | Both | 133 (105 to 184) | 1.7 (1.3 to 2.4) | 137 (107 to 191) | 1.8 (1.4 to 2.6) | 3361 (2633 to 4545) | 37.7 (29.6 to 51.7) | 30 (18 to 46) | 0.4 (0.2 to 0.6) | 3391 (2654 to 4590) | 38 (29.8 to 52.2) | 332 (258 to 431) | 1.7 (1.3 to 2.2) | 341 (267 to 441) | 1.8 (1.5 to 2.4) | 8487 (6375 to 11044) | 38 (29.4 to 49.3) | 74 (46 to 109) | 0.4 (0.2 to 0.5) | 8561 (6420 to 11151) | 38.4 (29.7 to 49.7) |
|  | Female | 94 (72 to 140) | 2.3 (1.7 to 3.6) | 96 (73 to 145) | 2.4 (1.9 to 3.9) | 2406 (1810 to 3526) | 52.9 (40.3 to 77.9) | 21 (13 to 35) | 0.5 (0.3 to 0.9) | 2427 (1824 to 3557) | 53.4 (40.7 to 78.7) | 222 (162 to 303) | 2.1 (1.6 to 2.9) | 228 (169 to 310) | 2.2 (1.7 to 3.1) | 5634 (3878 to 7746) | 47.8 (34.6 to 65.2) | 50 (29 to 76) | 0.5 (0.3 to 0.7) | 5684 (3917 to 7833) | 48.2 (34.8 to 65.9) |
|  | Male | 39 (29 to 69) | 1 (0.8 to 1.8) | 41 (31 to 72) | 1.1 (0.8 to 1.9) | 955 (701 to 1657) | 22.1 (16.3 to 38.8) | 9 (5 to 16) | 0.2 (0.1 to 0.4) | 963 (707 to 1674) | 22.3 (16.4 to 39.2) | 111 (80 to 144) | 1.2 (0.9 to 1.7) | 113 (82 to 149) | 1.4 (1 to 1.8) | 2853 (2041 to 3676) | 27.3 (19.7 to 35.9) | 25 (15 to 37) | 0.3 (0.2 to 0.4) | 2877 (2057 to 3715) | 27.6 (19.9 to 36.3) |
| Kiribati | Both | 0 (0 to 1) | 1.4 (1 to 1.6) | 0 (0 to 1) | 1.5 (1.1 to 1.8) | 12 (9 to 14) | 31.5 (23.4 to 36) | 0 (0 to 0) | 0.3 (0.2 to 0.4) | 12 (9 to 14) | 31.8 (23.6 to 36.4) | 1 (1 to 1) | 1.3 (0.9 to 1.6) | 1 (1 to 1) | 1.5 (1 to 1.8) | 20 (14 to 24) | 29 (20.5 to 34.7) | 0 (0 to 0) | 0.3 (0.2 to 0.4) | 20 (15 to 24) | 29.3 (20.7 to 35) |
|  | Female | 0 (0 to 0) | 1.6 (1 to 1.9) | 0 (0 to 0) | 1.7 (1.1 to 2) | 8 (5 to 9) | 35 (22.8 to 42.2) | 0 (0 to 0) | 0.3 (0.2 to 0.5) | 8 (5 to 9) | 35.4 (23 to 42.7) | 1 (0 to 1) | 1.5 (0.9 to 1.8) | 1 (0 to 1) | 1.6 (1 to 2) | 13 (8 to 16) | 32.3 (20.3 to 40.8) | 0 (0 to 0) | 0.3 (0.2 to 0.5) | 13 (8 to 17) | 32.6 (20.5 to 41.2) |
|  | Male | 0 (0 to 0) | 1.2 (0.9 to 1.5) | 0 (0 to 0) | 1.3 (1 to 1.7) | 5 (4 to 6) | 27.1 (19.7 to 34.3) | 0 (0 to 0) | 0.3 (0.2 to 0.4) | 5 (4 to 6) | 27.3 (19.9 to 34.6) | 0 (0 to 0) | 1.1 (0.8 to 1.5) | 0 (0 to 0) | 1.2 (0.9 to 1.6) | 7 (5 to 9) | 24.3 (17.9 to 31.4) | 0 (0 to 0) | 0.3 (0.2 to 0.4) | 7 (6 to 10) | 24.6 (18.1 to 31.7) |
| Kuwait | Both | 11 (9 to 12) | 1.8 (1.4 to 1.9) | 10 (8 to 11) | 1.8 (1.4 to 1.9) | 267 (221 to 290) | 35.6 (29.2 to 38.5) | 2 (2 to 3) | 0.4 (0.3 to 0.5) | 269 (223 to 292) | 36 (29.5 to 38.9) | 18 (16 to 24) | 0.8 (0.7 to 1) | 17 (15 to 22) | 0.8 (0.7 to 1) | 427 (367 to 562) | 15.3 (13.2 to 20) | 4 (3 to 6) | 0.2 (0.1 to 0.2) | 432 (371 to 567) | 15.4 (13.4 to 20.2) |
|  | Female | 6 (5 to 6) | 2.3 (2 to 2.7) | 5 (4 to 6) | 2.3 (2 to 2.5) | 135 (118 to 150) | 48.8 (42 to 54.2) | 1 (1 to 2) | 0.5 (0.4 to 0.7) | 136 (119 to 151) | 49.3 (42.5 to 54.6) | 8 (6 to 10) | 0.8 (0.7 to 1) | 7 (6 to 9) | 0.8 (0.7 to 1) | 181 (154 to 233) | 15.6 (13.5 to 19.7) | 2 (1 to 2) | 0.2 (0.1 to 0.2) | 183 (156 to 235) | 15.8 (13.6 to 19.9) |
|  | Male | 5 (3 to 6) | 1.4 (0.9 to 1.5) | 5 (3 to 5) | 1.4 (0.9 to 1.5) | 132 (90 to 148) | 27.9 (18.4 to 31) | 1 (1 to 2) | 0.3 (0.2 to 0.4) | 133 (90 to 150) | 28.2 (18.6 to 31.4) | 11 (9 to 16) | 0.8 (0.6 to 1.1) | 10 (8 to 15) | 0.7 (0.6 to 1.1) | 246 (196 to 378) | 15 (12.1 to 23) | 3 (2 to 4) | 0.2 (0.1 to 0.3) | 249 (198 to 382) | 15.2 (12.3 to 23.2) |
| Kyrgyzstan | Both | 38 (34 to 46) | 1.3 (1.1 to 1.6) | 40 (36 to 49) | 1.4 (1.2 to 1.7) | 899 (797 to 1069) | 28.8 (25.6 to 34.4) | 9 (6 to 12) | 0.3 (0.2 to 0.4) | 907 (805 to 1081) | 29.1 (25.9 to 34.8) | 38 (34 to 43) | 0.9 (0.8 to 1) | 39 (36 to 44) | 1 (0.9 to 1.1) | 950 (850 to 1075) | 21.1 (18.8 to 23.6) | 9 (6 to 12) | 0.2 (0.1 to 0.3) | 959 (857 to 1086) | 21.3 (19 to 23.8) |
|  | Female | 26 (23 to 34) | 1.4 (1.2 to 1.9) | 27 (24 to 36) | 1.5 (1.3 to 2) | 597 (521 to 770) | 32.5 (28.5 to 41.8) | 6 (4 to 8) | 0.3 (0.2 to 0.4) | 603 (525 to 777) | 32.9 (28.7 to 42.2) | 24 (22 to 28) | 1 (0.9 to 1.2) | 25 (23 to 29) | 1.1 (1 to 1.2) | 601 (534 to 698) | 23.7 (21.2 to 27.4) | 5 (4 to 7) | 0.2 (0.2 to 0.3) | 607 (538 to 705) | 24 (21.4 to 27.7) |
|  | Male | 12 (10 to 15) | 1.1 (0.8 to 1.4) | 13 (10 to 16) | 1.2 (0.9 to 1.5) | 302 (235 to 372) | 24.1 (18.9 to 29.7) | 3 (2 to 4) | 0.2 (0.2 to 0.3) | 304 (238 to 375) | 24.3 (19.1 to 30) | 14 (10 to 16) | 0.8 (0.6 to 0.9) | 14 (11 to 16) | 0.8 (0.6 to 1) | 349 (275 to 412) | 17.8 (13.4 to 20.9) | 3 (2 to 4) | 0.2 (0.1 to 0.2) | 352 (277 to 416) | 17.9 (13.5 to 21.1) |
| Laos | Both | 44 (31 to 59) | 2.2 (1.6 to 2.9) | 45 (32 to 60) | 2.4 (1.7 to 3.1) | 1125 (774 to 1530) | 50.6 (35.2 to 67.6) | 10 (6 to 14) | 0.5 (0.3 to 0.7) | 1135 (779 to 1544) | 51.1 (35.7 to 68.2) | 65 (52 to 82) | 1.7 (1.3 to 2.1) | 67 (53 to 84) | 1.8 (1.4 to 2.2) | 1605 (1264 to 2036) | 36.6 (28.9 to 46.2) | 15 (9 to 21) | 0.4 (0.2 to 0.5) | 1620 (1277 to 2054) | 37 (29.2 to 46.5) |
|  | Female | 25 (16 to 36) | 2.4 (1.5 to 3.3) | 26 (16 to 36) | 2.6 (1.7 to 3.5) | 633 (396 to 920) | 54.8 (34.8 to 77.9) | 6 (3 to 9) | 0.5 (0.3 to 0.8) | 638 (400 to 927) | 55.3 (35.2 to 78.6) | 36 (26 to 48) | 1.8 (1.3 to 2.3) | 37 (27 to 50) | 1.9 (1.4 to 2.5) | 889 (627 to 1187) | 39.1 (27.8 to 51.7) | 8 (5 to 12) | 0.4 (0.2 to 0.6) | 897 (632 to 1198) | 39.4 (28.1 to 52.3) |
|  | Male | 19 (11 to 29) | 2 (1.2 to 3) | 19 (11 to 30) | 2.2 (1.3 to 3.2) | 492 (283 to 777) | 45.9 (27.1 to 70.7) | 4 (2 to 7) | 0.4 (0.2 to 0.8) | 496 (285 to 783) | 46.4 (27.4 to 71.3) | 29 (21 to 40) | 1.6 (1.2 to 2.2) | 30 (22 to 41) | 1.7 (1.3 to 2.4) | 716 (518 to 999) | 34.1 (25.3 to 47.2) | 6 (4 to 10) | 0.3 (0.2 to 0.5) | 723 (523 to 1006) | 34.4 (25.6 to 47.6) |
| Latvia | Both | 58 (54 to 64) | 1.6 (1.5 to 1.7) | 60 (56 to 65) | 1.6 (1.5 to 1.7) | 1243 (1158 to 1343) | 33.7 (31.3 to 36.3) | 13 (9 to 17) | 0.4 (0.2 to 0.5) | 1256 (1171 to 1357) | 34 (31.7 to 36.8) | 54 (46 to 63) | 1.3 (1.1 to 1.5) | 54 (47 to 61) | 1.3 (1.1 to 1.4) | 955 (827 to 1098) | 25.2 (21.9 to 29.1) | 12 (8 to 17) | 0.3 (0.2 to 0.4) | 967 (837 to 1114) | 25.5 (22.1 to 29.6) |
|  | Female | 40 (36 to 44) | 1.6 (1.5 to 1.8) | 41 (37 to 44) | 1.6 (1.5 to 1.8) | 791 (713 to 863) | 34 (30.7 to 37.1) | 9 (6 to 12) | 0.4 (0.2 to 0.5) | 800 (721 to 871) | 34.4 (31.1 to 37.5) | 34 (27 to 44) | 1.3 (1 to 1.6) | 34 (28 to 41) | 1.2 (1 to 1.4) | 550 (456 to 667) | 22.6 (18.6 to 27.7) | 8 (5 to 11) | 0.3 (0.2 to 0.4) | 558 (462 to 675) | 22.9 (18.8 to 28) |
|  | Male | 19 (17 to 22) | 1.4 (1.3 to 1.7) | 19 (18 to 22) | 1.5 (1.4 to 1.7) | 452 (406 to 517) | 32.5 (29.4 to 36.9) | 4 (3 to 6) | 0.3 (0.2 to 0.4) | 456 (410 to 521) | 32.8 (29.7 to 37.3) | 19 (16 to 23) | 1.4 (1.1 to 1.6) | 20 (17 to 23) | 1.4 (1.2 to 1.6) | 405 (336 to 483) | 28.6 (23.7 to 34.1) | 4 (3 to 6) | 0.3 (0.2 to 0.4) | 409 (340 to 488) | 28.9 (24 to 34.4) |
| Lebanon | Both | 65 (49 to 78) | 3.1 (2.3 to 3.7) | 66 (50 to 79) | 3.2 (2.5 to 3.9) | 1520 (1138 to 1840) | 65.7 (50 to 79.3) | 14 (9 to 20) | 0.7 (0.4 to 0.9) | 1535 (1149 to 1857) | 66.3 (50.5 to 80.1) | 138 (109 to 172) | 2.4 (1.9 to 3) | 130 (102 to 161) | 2.3 (1.8 to 2.9) | 2695 (2155 to 3354) | 44.5 (35.5 to 54.9) | 32 (20 to 45) | 0.5 (0.3 to 0.8) | 2727 (2181 to 3394) | 45 (35.8 to 55.6) |
|  | Female | 43 (30 to 54) | 3.7 (2.6 to 4.6) | 44 (31 to 55) | 3.8 (2.8 to 4.8) | 1021 (677 to 1293) | 80.8 (54.7 to 102.2) | 10 (6 to 14) | 0.8 (0.5 to 1.2) | 1031 (685 to 1305) | 81.6 (55.3 to 103.4) | 91 (70 to 115) | 2.8 (2.2 to 3.6) | 88 (68 to 111) | 2.7 (2.1 to 3.5) | 1772 (1372 to 2237) | 53.5 (41.2 to 67.9) | 20 (13 to 30) | 0.6 (0.4 to 0.9) | 1792 (1387 to 2265) | 54.2 (41.7 to 68.8) |
|  | Male | 22 (14 to 27) | 2.3 (1.5 to 2.9) | 22 (14 to 28) | 2.5 (1.6 to 3.1) | 499 (322 to 657) | 48.2 (30.8 to 61.6) | 5 (3 to 7) | 0.5 (0.3 to 0.7) | 504 (325 to 664) | 48.7 (31.2 to 62.1) | 47 (33 to 63) | 1.8 (1.3 to 2.4) | 42 (28 to 55) | 1.7 (1.1 to 2.3) | 923 (652 to 1212) | 33.4 (22.9 to 43.9) | 11 (7 to 17) | 0.4 (0.3 to 0.6) | 934 (661 to 1228) | 33.8 (23.2 to 44.5) |
| Lesotho | Both | 11 (9 to 15) | 1.2 (0.9 to 1.6) | 12 (9 to 15) | 1.3 (1 to 1.7) | 263 (210 to 352) | 25.8 (20.7 to 34.4) | 2 (2 to 3) | 0.3 (0.2 to 0.4) | 265 (213 to 355) | 26 (20.9 to 34.7) | 14 (11 to 19) | 1.3 (1 to 1.7) | 15 (11 to 20) | 1.5 (1.1 to 1.9) | 342 (255 to 464) | 28.2 (21.2 to 38.2) | 3 (2 to 5) | 0.3 (0.2 to 0.4) | 345 (257 to 467) | 28.5 (21.4 to 38.6) |
|  | Female | 7 (5 to 10) | 1.2 (0.9 to 1.8) | 7 (5 to 11) | 1.4 (1 to 2) | 151 (111 to 230) | 26.7 (19.8 to 40.6) | 1 (1 to 2) | 0.3 (0.2 to 0.4) | 153 (112 to 232) | 27 (20 to 40.9) | 10 (6 to 14) | 1.5 (1 to 2.1) | 10 (7 to 15) | 1.6 (1.1 to 2.3) | 219 (142 to 318) | 30.5 (20 to 44) | 2 (1 to 3) | 0.3 (0.2 to 0.5) | 221 (144 to 321) | 30.8 (20.2 to 44.5) |
|  | Male | 4 (3 to 6) | 1.1 (0.7 to 1.4) | 4 (3 to 6) | 1.1 (0.8 to 1.5) | 112 (79 to 148) | 24 (16.8 to 31.1) | 1 (1 to 1) | 0.2 (0.1 to 0.3) | 113 (80 to 149) | 24.3 (16.9 to 31.3) | 5 (3 to 6) | 1 (0.7 to 1.4) | 5 (3 to 7) | 1.1 (0.7 to 1.5) | 123 (79 to 178) | 23.7 (15.6 to 33.5) | 1 (1 to 2) | 0.2 (0.1 to 0.4) | 124 (80 to 179) | 23.9 (15.7 to 33.8) |
| Liberia | Both | 16 (13 to 20) | 1.4 (1.2 to 1.8) | 17 (14 to 21) | 1.6 (1.3 to 1.9) | 362 (292 to 455) | 30.7 (24.9 to 38.3) | 3 (2 to 5) | 0.3 (0.2 to 0.4) | 365 (295 to 460) | 31 (25.1 to 38.7) | 19 (14 to 26) | 1.1 (0.8 to 1.5) | 20 (15 to 27) | 1.2 (0.9 to 1.7) | 455 (347 to 635) | 23.4 (18 to 32.3) | 4 (3 to 6) | 0.2 (0.2 to 0.4) | 459 (351 to 641) | 23.7 (18.2 to 32.7) |
|  | Female | 8 (6 to 11) | 1.6 (1.3 to 2.3) | 9 (7 to 12) | 1.8 (1.4 to 2.4) | 186 (142 to 256) | 35.2 (27 to 48.4) | 2 (1 to 3) | 0.4 (0.2 to 0.5) | 188 (144 to 258) | 35.6 (27.2 to 48.8) | 10 (7 to 15) | 1.2 (0.9 to 1.8) | 11 (8 to 16) | 1.4 (1 to 1.9) | 248 (175 to 356) | 26.3 (18.6 to 37.7) | 2 (1 to 4) | 0.3 (0.2 to 0.4) | 250 (176 to 360) | 26.5 (18.8 to 38.1) |
|  | Male | 8 (5 to 10) | 1.3 (0.9 to 1.6) | 8 (6 to 10) | 1.4 (1 to 1.8) | 175 (125 to 234) | 27 (19.2 to 35.7) | 2 (1 to 2) | 0.3 (0.2 to 0.4) | 177 (126 to 236) | 27.3 (19.5 to 36.1) | 8 (6 to 13) | 1 (0.7 to 1.5) | 9 (6 to 14) | 1 (0.8 to 1.6) | 207 (146 to 317) | 20.7 (14.8 to 31.8) | 2 (1 to 3) | 0.2 (0.1 to 0.3) | 209 (147 to 321) | 20.9 (15 to 32.1) |
| Libya | Both | 58 (46 to 72) | 3.1 (2.5 to 3.9) | 59 (47 to 73) | 3.3 (2.6 to 4.1) | 1395 (1088 to 1717) | 69.2 (54.1 to 85.4) | 13 (8 to 18) | 0.7 (0.4 to 1) | 1408 (1099 to 1732) | 69.9 (54.7 to 86.2) | 117 (90 to 150) | 2.7 (2.1 to 3.5) | 116 (90 to 149) | 2.8 (2.2 to 3.6) | 2816 (2120 to 3595) | 58 (44 to 73.8) | 26 (16 to 37) | 0.6 (0.4 to 0.8) | 2842 (2135 to 3633) | 58.5 (44.5 to 74.5) |
|  | Female | 42 (30 to 54) | 4.8 (3.5 to 6.2) | 43 (31 to 55) | 5 (3.7 to 6.5) | 998 (701 to 1270) | 106.4 (75.7 to 136.2) | 9 (6 to 14) | 1 (0.6 to 1.5) | 1007 (708 to 1281) | 107.4 (76.3 to 137.6) | 82 (56 to 113) | 3.9 (2.7 to 5.3) | 82 (58 to 113) | 4 (2.9 to 5.5) | 1972 (1293 to 2739) | 83.1 (56.1 to 114.5) | 18 (11 to 27) | 0.8 (0.5 to 1.3) | 1990 (1305 to 2771) | 84 (56.8 to 115.4) |
|  | Male | 16 (12 to 21) | 1.6 (1.2 to 2.1) | 16 (11 to 21) | 1.7 (1.2 to 2.2) | 398 (280 to 523) | 36.3 (25.6 to 47.4) | 4 (2 to 5) | 0.4 (0.2 to 0.5) | 401 (283 to 529) | 36.6 (25.8 to 47.9) | 35 (24 to 47) | 1.6 (1.1 to 2.1) | 34 (23 to 45) | 1.6 (1.1 to 2.1) | 844 (573 to 1153) | 33.4 (22.5 to 44.7) | 8 (5 to 12) | 0.4 (0.2 to 0.5) | 852 (578 to 1165) | 33.7 (22.8 to 45.2) |
| Lithuania | Both | 104 (95 to 116) | 2.2 (2.1 to 2.5) | 104 (97 to 111) | 2.2 (2.1 to 2.4) | 2136 (1991 to 2271) | 45.9 (42.7 to 48.8) | 23 (16 to 31) | 0.5 (0.3 to 0.7) | 2159 (2015 to 2299) | 46.4 (43.2 to 49.4) | 116 (100 to 139) | 1.9 (1.7 to 2.3) | 108 (94 to 118) | 1.7 (1.5 to 1.9) | 1891 (1671 to 2076) | 34.5 (30.6 to 37.8) | 26 (17 to 37) | 0.4 (0.3 to 0.6) | 1917 (1694 to 2101) | 34.9 (31.1 to 38.4) |
|  | Female | 76 (69 to 87) | 2.6 (2.4 to 3) | 75 (69 to 81) | 2.6 (2.4 to 2.7) | 1486 (1359 to 1588) | 52.6 (48.2 to 56.1) | 17 (12 to 24) | 0.6 (0.4 to 0.8) | 1503 (1376 to 1605) | 53.2 (48.8 to 56.8) | 81 (66 to 105) | 2 (1.7 to 2.6) | 71 (63 to 80) | 1.8 (1.5 to 2) | 1168 (1023 to 1314) | 34.2 (29.8 to 38.7) | 18 (12 to 27) | 0.5 (0.3 to 0.7) | 1186 (1039 to 1335) | 34.7 (30.2 to 39.2) |
|  | Male | 28 (25 to 31) | 1.6 (1.4 to 1.7) | 29 (26 to 32) | 1.7 (1.5 to 1.8) | 650 (581 to 730) | 35.3 (31.4 to 39.4) | 6 (4 to 9) | 0.4 (0.2 to 0.5) | 657 (586 to 737) | 35.6 (31.7 to 39.7) | 35 (25 to 40) | 1.7 (1.2 to 1.9) | 36 (26 to 41) | 1.7 (1.2 to 1.9) | 723 (552 to 829) | 34.6 (26.5 to 39.6) | 8 (5 to 11) | 0.4 (0.2 to 0.5) | 731 (557 to 837) | 35 (26.9 to 40) |
| Low-middle SDI | Both | 11118 (9892 to 15256) | 2.1 (1.8 to 2.8) | 11340 (10107 to 15565) | 2.2 (2 to 3.1) | 281758 (250324 to 382748) | 46.9 (41.8 to 64.2) | 2449 (1634 to 3687) | 0.5 (0.3 to 0.7) | 284207 (252324 to 385526) | 47.4 (42.1 to 64.8) | 24819 (21947 to 30464) | 2.2 (1.9 to 2.7) | 25574 (22570 to 31591) | 2.3 (2.1 to 2.9) | 608991 (534477 to 731794) | 49.3 (43.4 to 59.9) | 5472 (3713 to 7387) | 0.5 (0.3 to 0.6) | 614463 (538819 to 737866) | 49.8 (43.8 to 60.4) |
|  | Female | 7171 (6357 to 9719) | 2.7 (2.4 to 3.6) | 7318 (6480 to 10022) | 2.8 (2.5 to 3.9) | 180739 (159999 to 240851) | 60.7 (53.9 to 81.7) | 1564 (1049 to 2237) | 0.6 (0.4 to 0.8) | 182304 (161387 to 242898) | 61.2 (54.4 to 82.4) | 16643 (14070 to 20361) | 2.8 (2.4 to 3.4) | 17145 (14616 to 21007) | 2.9 (2.5 to 3.7) | 409275 (335827 to 490283) | 63.9 (53 to 77.2) | 3639 (2394 to 4953) | 0.6 (0.4 to 0.8) | 412915 (338607 to 494805) | 64.5 (53.5 to 78) |
|  | Male | 3947 (3288 to 6097) | 1.5 (1.2 to 2.2) | 4022 (3374 to 6166) | 1.6 (1.3 to 2.4) | 101019 (83413 to 157263) | 33.4 (27.8 to 51.4) | 885 (576 to 1353) | 0.3 (0.2 to 0.5) | 101903 (84088 to 158555) | 33.7 (28.1 to 51.8) | 8176 (6743 to 10982) | 1.5 (1.3 to 2.1) | 8429 (6962 to 11358) | 1.6 (1.4 to 2.2) | 199716 (164779 to 268576) | 33.8 (27.9 to 45.4) | 1832 (1197 to 2596) | 0.3 (0.2 to 0.5) | 201548 (166287 to 271287) | 34.1 (28.2 to 45.9) |
| Low SDI | Both | 6067 (4765 to 9060) | 2 (1.6 to 2.9) | 6189 (4879 to 9146) | 2.1 (1.7 to 3.1) | 156847 (122097 to 232711) | 44.7 (35.1 to 66.5) | 1354 (869 to 2116) | 0.4 (0.3 to 0.7) | 158201 (123277 to 234996) | 45.1 (35.4 to 67.3) | 14664 (11631 to 16907) | 2.2 (1.8 to 2.5) | 15216 (12082 to 17524) | 2.4 (1.9 to 2.7) | 364702 (287683 to 422162) | 49.9 (39.5 to 57.6) | 3258 (2135 to 4463) | 0.5 (0.3 to 0.7) | 367959 (289952 to 426083) | 50.4 (39.8 to 58.3) |
|  | Female | 3546 (2773 to 5539) | 2.4 (1.9 to 3.7) | 3611 (2834 to 5623) | 2.5 (2 to 3.9) | 92048 (71496 to 144520) | 54.4 (42.6 to 85) | 790 (501 to 1317) | 0.5 (0.3 to 0.9) | 92838 (72185 to 145406) | 55 (43 to 85.6) | 9322 (6992 to 10961) | 2.7 (2.1 to 3.2) | 9668 (7205 to 11359) | 3 (2.2 to 3.5) | 233254 (173388 to 276135) | 62.9 (46.8 to 74.1) | 2063 (1312 to 2882) | 0.6 (0.4 to 0.8) | 235317 (175175 to 278244) | 63.5 (47.4 to 74.8) |
|  | Male | 2521 (1872 to 3932) | 1.6 (1.2 to 2.4) | 2578 (1925 to 3998) | 1.7 (1.3 to 2.6) | 64799 (47242 to 100788) | 35.7 (26.5 to 55.6) | 564 (355 to 878) | 0.3 (0.2 to 0.5) | 65363 (47691 to 101477) | 36.1 (26.8 to 56) | 5341 (3805 to 6550) | 1.7 (1.2 to 2) | 5548 (3955 to 6792) | 1.8 (1.3 to 2.2) | 131447 (93900 to 160067) | 36.6 (26.1 to 44.7) | 1195 (746 to 1669) | 0.4 (0.2 to 0.5) | 132642 (94687 to 161613) | 37 (26.3 to 45.3) |
| Luxembourg | Both | 21 (18 to 27) | 3.8 (3.2 to 4.8) | 16 (14 to 17) | 2.8 (2.5 to 3) | 289 (257 to 308) | 51.7 (45.9 to 55.2) | 5 (3 to 7) | 0.9 (0.6 to 1.3) | 294 (262 to 314) | 52.6 (46.6 to 56.2) | 21 (17 to 27) | 2.1 (1.8 to 2.8) | 16 (14 to 19) | 1.5 (1.4 to 1.9) | 256 (223 to 307) | 27.3 (23.6 to 33) | 5 (3 to 8) | 0.5 (0.4 to 0.8) | 261 (227 to 314) | 27.9 (24.2 to 33.7) |
|  | Female | 13 (10 to 17) | 3.7 (3 to 4.9) | 10 (9 to 11) | 2.9 (2.6 to 3.2) | 174 (156 to 190) | 53.9 (48.3 to 58.9) | 3 (2 to 4) | 0.9 (0.6 to 1.3) | 177 (158 to 194) | 54.7 (48.9 to 60) | 10 (8 to 15) | 1.9 (1.5 to 2.6) | 9 (7 to 11) | 1.5 (1.2 to 1.9) | 132 (108 to 169) | 26.6 (21.6 to 33.8) | 2 (2 to 4) | 0.5 (0.3 to 0.7) | 135 (110 to 173) | 27 (21.9 to 34.5) |
|  | Male | 9 (6 to 13) | 3.9 (2.9 to 5.5) | 6 (4 to 6) | 2.6 (2 to 2.9) | 115 (88 to 126) | 48.9 (37.6 to 53.5) | 2 (1 to 4) | 1 (0.6 to 1.6) | 117 (90 to 129) | 49.9 (38.3 to 54.6) | 10 (8 to 15) | 2.4 (1.7 to 3.5) | 7 (6 to 9) | 1.6 (1.3 to 2) | 124 (100 to 159) | 28.2 (22.7 to 36.4) | 3 (2 to 4) | 0.6 (0.4 to 1) | 126 (102 to 162) | 28.8 (23.2 to 37.1) |
| Macedonia | Both | 41 (35 to 48) | 2.2 (1.9 to 2.6) | 42 (36 to 50) | 2.3 (2 to 2.8) | 973 (802 to 1121) | 49.3 (41 to 57.2) | 9 (6 to 12) | 0.5 (0.3 to 0.7) | 982 (809 to 1131) | 49.8 (41.4 to 57.8) | 57 (48 to 72) | 1.7 (1.4 to 2.1) | 61 (51 to 77) | 1.8 (1.5 to 2.3) | 1275 (1070 to 1586) | 37.8 (31.8 to 46.9) | 13 (8 to 18) | 0.4 (0.2 to 0.5) | 1288 (1082 to 1598) | 38.1 (32.1 to 47.3) |
|  | Female | 28 (22 to 33) | 2.8 (2.3 to 3.3) | 29 (23 to 33) | 3 (2.4 to 3.5) | 659 (499 to 763) | 63.9 (49.1 to 74.1) | 6 (4 to 9) | 0.6 (0.4 to 0.9) | 665 (504 to 772) | 64.5 (49.5 to 74.7) | 35 (29 to 44) | 1.9 (1.6 to 2.4) | 38 (30 to 48) | 2 (1.7 to 2.6) | 795 (644 to 991) | 45.3 (36.6 to 56.3) | 8 (5 to 11) | 0.4 (0.3 to 0.6) | 802 (650 to 999) | 45.7 (37 to 56.9) |
|  | Male | 13 (10 to 18) | 1.5 (1.2 to 2.1) | 14 (11 to 19) | 1.6 (1.3 to 2.3) | 314 (243 to 412) | 33.5 (26.3 to 44.9) | 3 (2 to 4) | 0.3 (0.2 to 0.5) | 317 (245 to 417) | 33.8 (26.6 to 45.5) | 21 (16 to 31) | 1.4 (1.1 to 2) | 23 (18 to 34) | 1.5 (1.2 to 2.2) | 480 (366 to 699) | 30.3 (23.1 to 43.9) | 5 (3 to 7) | 0.3 (0.2 to 0.5) | 485 (370 to 706) | 30.6 (23.4 to 44.4) |
| Madagascar | Both | 48 (39 to 68) | 1 (0.8 to 1.4) | 50 (40 to 70) | 1.1 (0.9 to 1.5) | 1235 (982 to 1754) | 22.4 (18 to 31.8) | 11 (7 to 16) | 0.2 (0.1 to 0.3) | 1246 (990 to 1773) | 22.7 (18.1 to 32.1) | 85 (67 to 116) | 0.9 (0.7 to 1.2) | 87 (69 to 119) | 1 (0.8 to 1.4) | 2278 (1788 to 3110) | 20.6 (16.3 to 28.1) | 19 (12 to 28) | 0.2 (0.1 to 0.3) | 2297 (1802 to 3134) | 20.8 (16.5 to 28.3) |
|  | Female | 29 (22 to 45) | 1.3 (1 to 1.9) | 30 (23 to 46) | 1.4 (1.1 to 2.1) | 754 (566 to 1146) | 28.5 (21.6 to 43.1) | 6 (4 to 11) | 0.3 (0.2 to 0.4) | 761 (571 to 1157) | 28.7 (21.7 to 43.6) | 53 (37 to 78) | 1.1 (0.8 to 1.6) | 54 (38 to 81) | 1.2 (0.9 to 1.8) | 1387 (951 to 2031) | 24.5 (17.1 to 36.5) | 12 (7 to 18) | 0.2 (0.1 to 0.4) | 1398 (958 to 2048) | 24.7 (17.3 to 36.8) |
|  | Male | 19 (14 to 25) | 0.7 (0.5 to 1) | 20 (14 to 26) | 0.8 (0.6 to 1) | 481 (354 to 644) | 16.9 (12.3 to 22.6) | 4 (3 to 6) | 0.2 (0.1 to 0.2) | 485 (356 to 647) | 17.1 (12.4 to 22.7) | 32 (22 to 45) | 0.7 (0.5 to 1) | 33 (23 to 46) | 0.8 (0.6 to 1.1) | 892 (609 to 1239) | 16.4 (11.5 to 22.5) | 7 (4 to 11) | 0.2 (0.1 to 0.2) | 899 (616 to 1247) | 16.5 (11.6 to 22.7) |
| Malawi | Both | 37 (26 to 48) | 1 (0.7 to 1.2) | 39 (28 to 51) | 1.1 (0.8 to 1.3) | 915 (587 to 1196) | 21.3 (14.4 to 27.5) | 8 (5 to 12) | 0.2 (0.1 to 0.3) | 923 (593 to 1206) | 21.5 (14.6 to 27.8) | 58 (47 to 73) | 0.8 (0.7 to 1) | 62 (51 to 78) | 0.9 (0.7 to 1.1) | 1405 (1117 to 1761) | 18.3 (14.6 to 23) | 13 (9 to 18) | 0.2 (0.1 to 0.3) | 1418 (1126 to 1779) | 18.4 (14.8 to 23.1) |
|  | Female | 26 (17 to 35) | 1.3 (0.9 to 1.7) | 27 (18 to 37) | 1.4 (1 to 1.8) | 636 (402 to 897) | 28.1 (18.4 to 39.2) | 6 (3 to 9) | 0.3 (0.2 to 0.4) | 642 (405 to 907) | 28.4 (18.6 to 39.5) | 39 (30 to 53) | 1 (0.7 to 1.3) | 42 (32 to 57) | 1 (0.8 to 1.4) | 929 (691 to 1248) | 21.6 (16.1 to 29.2) | 9 (5 to 13) | 0.2 (0.1 to 0.3) | 938 (698 to 1260) | 21.8 (16.3 to 29.6) |
|  | Male | 12 (7 to 16) | 0.6 (0.4 to 0.9) | 12 (7 to 17) | 0.7 (0.4 to 1) | 278 (146 to 392) | 13.8 (7.5 to 19.1) | 3 (1 to 4) | 0.1 (0.1 to 0.2) | 281 (148 to 395) | 14 (7.5 to 19.3) | 19 (13 to 25) | 0.6 (0.4 to 0.8) | 20 (14 to 26) | 0.7 (0.5 to 0.9) | 476 (325 to 604) | 14.1 (9.6 to 17.8) | 4 (3 to 6) | 0.1 (0.1 to 0.2) | 480 (328 to 609) | 14.3 (9.7 to 18) |
| Malaysia | Both | 128 (88 to 148) | 1.5 (1 to 1.7) | 131 (89 to 152) | 1.6 (1.1 to 1.8) | 3069 (2134 to 3541) | 31.8 (21.9 to 36.8) | 29 (18 to 40) | 0.3 (0.2 to 0.5) | 3098 (2158 to 3569) | 32.1 (22.1 to 37.2) | 308 (216 to 368) | 1.3 (0.9 to 1.5) | 284 (205 to 334) | 1.2 (0.9 to 1.4) | 6236 (4540 to 7354) | 23.8 (17.3 to 28.1) | 72 (46 to 101) | 0.3 (0.2 to 0.4) | 6308 (4593 to 7430) | 24.1 (17.5 to 28.4) |
|  | Female | 65 (40 to 78) | 1.5 (0.9 to 1.7) | 67 (41 to 80) | 1.5 (0.9 to 1.9) | 1524 (956 to 1795) | 31 (19.1 to 36.6) | 15 (8 to 21) | 0.3 (0.2 to 0.5) | 1538 (965 to 1814) | 31.3 (19.4 to 37) | 144 (88 to 188) | 1.2 (0.7 to 1.5) | 132 (81 to 166) | 1.1 (0.7 to 1.4) | 2820 (1774 to 3598) | 21.6 (13.5 to 27.4) | 34 (20 to 49) | 0.3 (0.2 to 0.4) | 2854 (1794 to 3638) | 21.9 (13.6 to 27.8) |
|  | Male | 63 (37 to 77) | 1.5 (0.9 to 1.8) | 64 (38 to 79) | 1.6 (1 to 2) | 1545 (927 to 1893) | 32.6 (19.4 to 40) | 14 (8 to 20) | 0.3 (0.2 to 0.5) | 1559 (936 to 1906) | 32.9 (19.5 to 40.5) | 163 (102 to 208) | 1.4 (0.9 to 1.7) | 151 (95 to 187) | 1.3 (0.8 to 1.6) | 3416 (2127 to 4213) | 26 (16.3 to 32.1) | 38 (23 to 56) | 0.3 (0.2 to 0.5) | 3454 (2152 to 4262) | 26.3 (16.5 to 32.5) |
| Maldives | Both | 1 (1 to 2) | 1.3 (0.8 to 1.9) | 1 (1 to 2) | 1.4 (0.9 to 2.1) | 26 (17 to 41) | 27.8 (17.8 to 41.7) | 0 (0 to 0) | 0.3 (0.2 to 0.5) | 27 (17 to 41) | 28.1 (18 to 42.2) | 3 (2 to 3) | 0.9 (0.6 to 1.2) | 2 (2 to 3) | 0.9 (0.6 to 1.1) | 49 (36 to 58) | 16.9 (12.1 to 20.3) | 1 (0 to 1) | 0.2 (0.1 to 0.3) | 49 (37 to 59) | 17.1 (12.3 to 20.5) |
|  | Female | 0 (0 to 1) | 0.9 (0.5 to 1.5) | 0 (0 to 1) | 1 (0.6 to 1.6) | 8 (5 to 16) | 20.5 (11.9 to 35.6) | 0 (0 to 0) | 0.2 (0.1 to 0.4) | 8 (5 to 16) | 20.8 (12 to 36) | 1 (1 to 1) | 0.7 (0.5 to 0.9) | 1 (1 to 1) | 0.6 (0.4 to 0.8) | 16 (12 to 20) | 12.3 (8.9 to 15) | 0 (0 to 0) | 0.2 (0.1 to 0.2) | 17 (12 to 20) | 12.5 (9 to 15.2) |
|  | Male | 1 (0 to 1) | 1.5 (0.7 to 2.4) | 1 (0 to 1) | 1.6 (0.8 to 2.6) | 18 (9 to 28) | 32.7 (15.4 to 51.4) | 0 (0 to 0) | 0.3 (0.1 to 0.6) | 18 (9 to 28) | 33 (15.6 to 51.9) | 2 (1 to 2) | 1.2 (0.7 to 1.5) | 2 (1 to 2) | 1.1 (0.6 to 1.4) | 32 (22 to 40) | 20.9 (13.1 to 26.3) | 0 (0 to 1) | 0.3 (0.2 to 0.4) | 33 (22 to 41) | 21.2 (13.3 to 26.7) |
| Mali | Both | 45 (36 to 53) | 1.3 (1 to 1.5) | 47 (37 to 55) | 1.4 (1 to 1.6) | 1097 (874 to 1268) | 26.8 (21 to 30.9) | 10 (7 to 14) | 0.3 (0.2 to 0.4) | 1107 (881 to 1280) | 27.1 (21.3 to 31.2) | 75 (54 to 98) | 1 (0.7 to 1.3) | 79 (57 to 103) | 1.1 (0.8 to 1.4) | 1762 (1287 to 2315) | 21.2 (15.3 to 27.8) | 17 (10 to 25) | 0.2 (0.1 to 0.3) | 1779 (1300 to 2338) | 21.5 (15.5 to 28.1) |
|  | Female | 24 (17 to 29) | 1.4 (1 to 1.6) | 25 (18 to 31) | 1.5 (1 to 1.8) | 581 (432 to 699) | 28.9 (20.8 to 35) | 5 (3 to 8) | 0.3 (0.2 to 0.4) | 587 (436 to 706) | 29.2 (21.1 to 35.4) | 35 (26 to 49) | 1 (0.7 to 1.4) | 37 (27 to 52) | 1.1 (0.8 to 1.5) | 821 (604 to 1154) | 20.8 (15.4 to 28.8) | 8 (5 to 12) | 0.2 (0.1 to 0.3) | 829 (608 to 1163) | 21 (15.5 to 29.1) |
|  | Male | 21 (15 to 26) | 1.2 (0.8 to 1.4) | 22 (15 to 27) | 1.3 (0.9 to 1.6) | 516 (365 to 643) | 24.7 (17.2 to 30.5) | 5 (3 to 7) | 0.3 (0.2 to 0.4) | 521 (368 to 649) | 25 (17.4 to 30.7) | 40 (24 to 57) | 1 (0.6 to 1.5) | 42 (25 to 60) | 1.1 (0.7 to 1.6) | 941 (572 to 1370) | 21.7 (13.2 to 31.4) | 9 (5 to 14) | 0.2 (0.1 to 0.4) | 950 (577 to 1382) | 22 (13.3 to 31.7) |
| Malta | Both | 10 (9 to 12) | 2.3 (2 to 2.7) | 8 (7 to 9) | 1.9 (1.7 to 2.1) | 155 (142 to 166) | 35.3 (32.4 to 37.6) | 2 (2 to 3) | 0.5 (0.4 to 0.8) | 157 (145 to 168) | 35.8 (32.9 to 38.2) | 13 (11 to 16) | 1.5 (1.2 to 1.8) | 11 (10 to 12) | 1.2 (1.1 to 1.3) | 187 (169 to 204) | 21.6 (19.6 to 23.4) | 3 (2 to 4) | 0.4 (0.3 to 0.5) | 190 (172 to 208) | 21.9 (20 to 23.8) |
|  | Female | 5 (5 to 6) | 2.2 (1.9 to 2.7) | 5 (4 to 5) | 2 (1.8 to 2.1) | 88 (79 to 96) | 36 (32.5 to 39.5) | 1 (1 to 2) | 0.5 (0.3 to 0.7) | 89 (80 to 98) | 36.5 (33 to 40.1) | 6 (5 to 8) | 1.2 (1 to 1.5) | 6 (5 to 6) | 1.1 (1 to 1.2) | 89 (79 to 102) | 19.2 (17 to 21.6) | 1 (1 to 2) | 0.3 (0.2 to 0.4) | 91 (80 to 103) | 19.5 (17.2 to 22) |
|  | Male | 4 (4 to 6) | 2.4 (1.9 to 3.1) | 3 (3 to 4) | 1.9 (1.6 to 2.1) | 67 (58 to 74) | 34.4 (29.8 to 37.8) | 1 (1 to 2) | 0.6 (0.4 to 0.9) | 68 (59 to 75) | 35 (30.2 to 38.6) | 7 (5 to 9) | 1.8 (1.4 to 2.3) | 6 (5 to 6) | 1.4 (1.1 to 1.6) | 97 (82 to 109) | 24.3 (20.6 to 27.2) | 2 (1 to 3) | 0.4 (0.3 to 0.6) | 99 (84 to 112) | 24.8 (21 to 27.7) |
| Marshall Islands | Both | 0 (0 to 0) | 1.4 (1.1 to 1.8) | 0 (0 to 0) | 1.5 (1.2 to 1.9) | 6 (4 to 7) | 32.4 (25 to 40.6) | 0 (0 to 0) | 0.3 (0.2 to 0.4) | 6 (4 to 7) | 32.7 (25.2 to 40.9) | 0 (0 to 1) | 1.3 (1 to 1.6) | 0 (0 to 0) | 1.4 (1.1 to 1.7) | 11 (8 to 14) | 29.7 (22.5 to 37.9) | 0 (0 to 0) | 0.3 (0.2 to 0.4) | 11 (8 to 14) | 30 (22.7 to 38.3) |
|  | Female | 0 (0 to 0) | 1.5 (1 to 1.9) | 0 (0 to 0) | 1.5 (1.1 to 2) | 3 (2 to 4) | 33.6 (24 to 42.8) | 0 (0 to 0) | 0.3 (0.2 to 0.5) | 3 (2 to 4) | 33.9 (24.2 to 43.3) | 0 (0 to 0) | 1.3 (0.8 to 1.8) | 0 (0 to 0) | 1.4 (0.9 to 1.9) | 6 (3 to 8) | 30.2 (18.9 to 42.5) | 0 (0 to 0) | 0.3 (0.2 to 0.4) | 6 (3 to 8) | 30.5 (19.1 to 43) |
|  | Male | 0 (0 to 0) | 1.4 (1 to 2.1) | 0 (0 to 0) | 1.5 (1 to 2.2) | 3 (2 to 4) | 31.5 (20.6 to 47.2) | 0 (0 to 0) | 0.3 (0.2 to 0.5) | 3 (2 to 4) | 31.8 (20.9 to 47.6) | 0 (0 to 0) | 1.3 (0.9 to 1.9) | 0 (0 to 0) | 1.4 (1 to 2) | 5 (3 to 7) | 29.3 (19.6 to 41.6) | 0 (0 to 0) | 0.3 (0.2 to 0.5) | 5 (3 to 7) | 29.6 (19.8 to 41.9) |
| Mauritania | Both | 17 (13 to 22) | 1.7 (1.4 to 2.2) | 17 (14 to 23) | 1.8 (1.5 to 2.3) | 374 (304 to 500) | 35.6 (29.1 to 47.4) | 4 (2 to 5) | 0.4 (0.2 to 0.5) | 377 (307 to 503) | 36 (29.4 to 47.8) | 23 (17 to 33) | 1.2 (0.9 to 1.8) | 24 (18 to 34) | 1.3 (1 to 1.9) | 523 (383 to 742) | 26 (19.2 to 37) | 5 (3 to 8) | 0.3 (0.2 to 0.4) | 528 (387 to 751) | 26.2 (19.4 to 37.5) |
|  | Female | 10 (8 to 15) | 1.9 (1.5 to 2.9) | 11 (8 to 16) | 2.1 (1.6 to 3.1) | 223 (166 to 334) | 41.1 (30.4 to 61.3) | 2 (1 to 3) | 0.4 (0.3 to 0.7) | 226 (167 to 337) | 41.5 (30.8 to 62) | 13 (9 to 19) | 1.4 (1 to 2) | 14 (10 to 20) | 1.5 (1.1 to 2.2) | 293 (212 to 423) | 29.2 (21.2 to 42.2) | 3 (2 to 4) | 0.3 (0.2 to 0.5) | 296 (214 to 427) | 29.5 (21.4 to 42.7) |
|  | Male | 6 (5 to 8) | 1.4 (1 to 1.8) | 7 (5 to 9) | 1.5 (1.1 to 1.9) | 150 (107 to 199) | 29.5 (21.1 to 38.9) | 1 (1 to 2) | 0.3 (0.2 to 0.4) | 152 (108 to 201) | 29.8 (21.3 to 39.3) | 10 (6 to 18) | 1.1 (0.7 to 1.9) | 11 (6 to 18) | 1.2 (0.7 to 2) | 229 (134 to 402) | 22.7 (13.4 to 39.9) | 2 (1 to 4) | 0.2 (0.1 to 0.4) | 232 (135 to 406) | 23 (13.5 to 40.3) |
| Mauritius | Both | 11 (10 to 12) | 1.6 (1.4 to 1.7) | 11 (10 to 12) | 1.6 (1.4 to 1.7) | 255 (223 to 273) | 33 (28.7 to 35.2) | 3 (2 to 3) | 0.4 (0.2 to 0.5) | 258 (226 to 276) | 33.3 (29.1 to 35.6) | 19 (17 to 22) | 1.2 (1.1 to 1.4) | 18 (16 to 20) | 1.1 (1 to 1.2) | 386 (349 to 428) | 22.4 (20.3 to 24.7) | 5 (3 to 6) | 0.3 (0.2 to 0.4) | 391 (352 to 434) | 22.7 (20.5 to 25) |
|  | Female | 6 (6 to 7) | 1.6 (1.4 to 1.8) | 6 (6 to 7) | 1.6 (1.5 to 1.7) | 137 (125 to 150) | 32.7 (29.9 to 35.8) | 1 (1 to 2) | 0.4 (0.2 to 0.5) | 138 (127 to 151) | 33.1 (30.3 to 36.1) | 13 (11 to 15) | 1.4 (1.2 to 1.6) | 11 (10 to 13) | 1.3 (1.1 to 1.4) | 245 (212 to 277) | 26.2 (22.7 to 29.6) | 3 (2 to 4) | 0.3 (0.2 to 0.4) | 248 (215 to 281) | 26.5 (23 to 30) |
|  | Male | 5 (4 to 5) | 1.6 (1.2 to 1.7) | 5 (4 to 5) | 1.7 (1.2 to 1.8) | 118 (90 to 131) | 33.6 (25.2 to 36.8) | 1 (1 to 1) | 0.4 (0.2 to 0.5) | 119 (91 to 132) | 33.9 (25.5 to 37.3) | 7 (6 to 8) | 1 (0.8 to 1.2) | 6 (6 to 7) | 0.9 (0.8 to 1.1) | 141 (124 to 164) | 18.2 (16.1 to 21.1) | 2 (1 to 2) | 0.2 (0.2 to 0.3) | 143 (125 to 166) | 18.4 (16.3 to 21.3) |
| Mexico | Both | 1760 (1617 to 1831) | 4.1 (3.7 to 4.3) | 1822 (1671 to 1895) | 4.3 (4 to 4.5) | 41669 (38390 to 43312) | 90.6 (83.2 to 94.2) | 381 (263 to 502) | 0.9 (0.6 to 1.2) | 42050 (38692 to 43710) | 91.5 (84 to 95.1) | 2440 (2324 to 2924) | 2.2 (2.1 to 2.6) | 2534 (2418 to 3026) | 2.3 (2.2 to 2.7) | 54821 (52337 to 65494) | 46.9 (44.7 to 56.2) | 543 (373 to 744) | 0.5 (0.3 to 0.7) | 55365 (52885 to 66253) | 47.3 (45.2 to 56.9) |
|  | Female | 1282 (1240 to 1340) | 5.7 (5.5 to 5.9) | 1323 (1281 to 1384) | 5.9 (5.8 to 6.2) | 30620 (29566 to 31991) | 127.7 (123.3 to 133.4) | 275 (188 to 363) | 1.2 (0.8 to 1.6) | 30895 (29869 to 32275) | 128.9 (124.7 to 134.7) | 1691 (1603 to 1985) | 2.8 (2.7 to 3.3) | 1759 (1669 to 2053) | 2.9 (2.8 to 3.4) | 38367 (36393 to 44938) | 61.5 (58.3 to 72) | 375 (257 to 509) | 0.6 (0.4 to 0.8) | 38742 (36748 to 45276) | 62.1 (58.9 to 72.5) |
|  | Male | 478 (389 to 497) | 2.4 (1.9 to 2.5) | 499 (400 to 520) | 2.6 (2 to 2.7) | 11049 (8906 to 11507) | 50.4 (40.7 to 52.5) | 106 (71 to 141) | 0.5 (0.3 to 0.7) | 11155 (8999 to 11626) | 51 (41.1 to 53.1) | 749 (692 to 911) | 1.4 (1.3 to 1.8) | 775 (717 to 946) | 1.5 (1.4 to 1.9) | 16454 (15229 to 19819) | 30.3 (28 to 36.5) | 169 (114 to 230) | 0.3 (0.2 to 0.4) | 16623 (15393 to 20011) | 30.6 (28.3 to 36.8) |
| Middle SDI | Both | 18781 (17386 to 22623) | 2.1 (1.9 to 2.5) | 19186 (17775 to 23250) | 2.2 (2.1 to 2.7) | 461726 (424615 to 549336) | 45.9 (42.5 to 55.2) | 4143 (2842 to 5591) | 0.5 (0.3 to 0.6) | 465870 (428061 to 555465) | 46.3 (42.8 to 55.7) | 40177 (33270 to 43681) | 1.9 (1.6 to 2.1) | 39712 (32966 to 43332) | 1.9 (1.6 to 2.1) | 870710 (713300 to 938142) | 38.5 (31.6 to 41.6) | 9025 (6118 to 12062) | 0.4 (0.3 to 0.6) | 879735 (721174 to 947949) | 38.9 (32 to 42) |
|  | Female | 11319 (10431 to 13727) | 2.4 (2.2 to 2.9) | 11600 (10735 to 14104) | 2.5 (2.4 to 3.1) | 274279 (249469 to 329354) | 53.2 (48.9 to 64.1) | 2477 (1690 to 3328) | 0.5 (0.4 to 0.7) | 276756 (251942 to 332360) | 53.7 (49.3 to 64.7) | 23374 (18425 to 25101) | 2.1 (1.6 to 2.2) | 23246 (18492 to 24913) | 2.1 (1.7 to 2.2) | 502759 (392883 to 538109) | 42.7 (33.5 to 45.8) | 5210 (3500 to 7007) | 0.5 (0.3 to 0.6) | 507969 (397779 to 544474) | 43.1 (33.9 to 46.3) |
|  | Male | 7462 (6309 to 9792) | 1.8 (1.5 to 2.3) | 7585 (6433 to 9959) | 1.9 (1.6 to 2.5) | 187447 (157465 to 248067) | 38.2 (32.3 to 50.1) | 1666 (1117 to 2343) | 0.4 (0.3 to 0.5) | 189114 (158894 to 250299) | 38.6 (32.6 to 50.6) | 16803 (12812 to 20043) | 1.7 (1.3 to 2) | 16467 (12527 to 19774) | 1.7 (1.3 to 2.1) | 367951 (275504 to 434613) | 34 (25.6 to 40.4) | 3815 (2508 to 5262) | 0.4 (0.2 to 0.5) | 371766 (277966 to 440134) | 34.4 (25.8 to 40.9) |
| Moldova | Both | 74 (63 to 82) | 1.6 (1.4 to 1.8) | 75 (65 to 82) | 1.7 (1.5 to 1.8) | 1812 (1526 to 2009) | 38.3 (32.4 to 42.2) | 17 (11 to 22) | 0.4 (0.2 to 0.5) | 1829 (1541 to 2027) | 38.6 (32.7 to 42.7) | 43 (39 to 52) | 0.7 (0.7 to 0.9) | 43 (39 to 53) | 0.7 (0.7 to 0.9) | 966 (884 to 1160) | 17 (15.6 to 20.3) | 10 (6 to 13) | 0.2 (0.1 to 0.2) | 976 (893 to 1171) | 17.2 (15.7 to 20.5) |
|  | Female | 48 (42 to 54) | 1.8 (1.6 to 2) | 49 (43 to 54) | 1.8 (1.6 to 2) | 1149 (980 to 1288) | 41.6 (35.7 to 46.6) | 11 (7 to 14) | 0.4 (0.3 to 0.5) | 1160 (989 to 1298) | 42 (36 to 47) | 26 (23 to 31) | 0.8 (0.7 to 0.9) | 26 (23 to 31) | 0.7 (0.7 to 0.9) | 560 (497 to 651) | 16.9 (15 to 19.6) | 6 (4 to 8) | 0.2 (0.1 to 0.2) | 566 (502 to 659) | 17.1 (15.1 to 19.8) |
|  | Male | 26 (19 to 30) | 1.4 (1.1 to 1.6) | 26 (20 to 30) | 1.4 (1.2 to 1.7) | 663 (468 to 769) | 33.5 (24.6 to 38.7) | 6 (4 to 8) | 0.3 (0.2 to 0.4) | 669 (473 to 777) | 33.8 (24.8 to 39.1) | 17 (15 to 23) | 0.7 (0.6 to 1) | 17 (15 to 24) | 0.7 (0.6 to 1.1) | 406 (360 to 534) | 17 (15.1 to 22.7) | 4 (3 to 5) | 0.2 (0.1 to 0.2) | 410 (364 to 539) | 17.1 (15.2 to 22.9) |
| Mongolia | Both | 31 (11 to 45) | 2.9 (1 to 4.3) | 32 (11 to 47) | 3.1 (1.1 to 4.6) | 759 (265 to 1090) | 68.4 (23.7 to 98.9) | 7 (2 to 11) | 0.6 (0.2 to 1) | 766 (266 to 1100) | 69.1 (23.8 to 100) | 45 (17 to 64) | 2.2 (0.8 to 3.1) | 45 (17 to 64) | 2.3 (0.8 to 3.3) | 1195 (462 to 1687) | 49.8 (18.8 to 70.6) | 10 (4 to 16) | 0.5 (0.2 to 0.8) | 1205 (466 to 1702) | 50.3 (19 to 71.4) |
|  | Female | 27 (4 to 43) | 4.8 (0.8 to 7.4) | 28 (5 to 45) | 5 (0.8 to 7.8) | 676 (107 to 1037) | 113.7 (17.9 to 174.4) | 6 (1 to 10) | 1 (0.1 to 1.8) | 682 (108 to 1044) | 114.7 (18.1 to 175.9) | 38 (7 to 58) | 3.2 (0.6 to 4.9) | 39 (7 to 59) | 3.4 (0.6 to 5.2) | 1009 (174 to 1538) | 75.6 (13.1 to 115.7) | 9 (1 to 15) | 0.7 (0.1 to 1.2) | 1018 (176 to 1554) | 76.3 (13.3 to 116.9) |
|  | Male | 3 (2 to 9) | 0.7 (0.4 to 2) | 3 (2 to 9) | 0.8 (0.4 to 2.1) | 83 (44 to 229) | 16.1 (8.5 to 45.2) | 1 (0 to 2) | 0.2 (0.1 to 0.4) | 84 (44 to 231) | 16.3 (8.6 to 45.6) | 7 (4 to 15) | 0.7 (0.4 to 1.6) | 7 (4 to 15) | 0.8 (0.5 to 1.8) | 186 (112 to 418) | 16.9 (10.4 to 38.1) | 2 (1 to 4) | 0.2 (0.1 to 0.4) | 188 (113 to 421) | 17 (10.5 to 38.4) |
| Montenegro | Both | 14 (12 to 17) | 2.3 (1.9 to 2.8) | 15 (12 to 18) | 2.5 (2 to 2.9) | 309 (254 to 362) | 47.9 (39.4 to 56.1) | 3 (2 to 4) | 0.5 (0.3 to 0.7) | 312 (257 to 366) | 48.4 (39.8 to 56.7) | 20 (17 to 24) | 2 (1.6 to 2.4) | 22 (18 to 26) | 2.2 (1.8 to 2.5) | 420 (350 to 497) | 41.8 (34.8 to 49.5) | 4 (3 to 6) | 0.4 (0.3 to 0.6) | 425 (354 to 501) | 42.3 (35.1 to 50.1) |
|  | Female | 11 (8 to 13) | 3 (2.3 to 3.7) | 11 (9 to 13) | 3.1 (2.4 to 3.7) | 218 (172 to 265) | 60.4 (47.5 to 73.5) | 2 (1 to 3) | 0.6 (0.4 to 0.9) | 220 (173 to 268) | 61.1 (48 to 74.2) | 14 (11 to 17) | 2.6 (2 to 3.1) | 16 (12 to 19) | 2.7 (2.1 to 3.3) | 290 (229 to 358) | 53.1 (42.2 to 65.4) | 3 (2 to 4) | 0.6 (0.4 to 0.8) | 293 (231 to 361) | 53.6 (42.6 to 66) |
|  | Male | 4 (3 to 5) | 1.5 (1.1 to 1.9) | 4 (3 to 6) | 1.6 (1.2 to 2.1) | 91 (70 to 120) | 31.8 (24.3 to 41.8) | 1 (1 to 1) | 0.3 (0.2 to 0.5) | 92 (70 to 121) | 32.1 (24.5 to 42.2) | 6 (5 to 8) | 1.3 (1 to 1.7) | 7 (5 to 8) | 1.5 (1.1 to 1.9) | 130 (99 to 170) | 28.5 (21.8 to 37.3) | 1 (1 to 2) | 0.3 (0.2 to 0.4) | 132 (100 to 172) | 28.8 (22 to 37.7) |
| Morocco | Both | 167 (132 to 195) | 1.2 (0.9 to 1.4) | 173 (138 to 202) | 1.2 (1 to 1.5) | 4095 (3069 to 4792) | 26.9 (20.4 to 31.4) | 37 (24 to 51) | 0.3 (0.2 to 0.4) | 4132 (3100 to 4831) | 27.2 (20.7 to 31.7) | 314 (238 to 388) | 1 (0.8 to 1.3) | 326 (250 to 407) | 1.1 (0.8 to 1.4) | 7418 (5395 to 9277) | 22.6 (16.7 to 28.2) | 70 (44 to 100) | 0.2 (0.1 to 0.3) | 7488 (5447 to 9381) | 22.8 (16.9 to 28.4) |
|  | Female | 124 (91 to 150) | 1.7 (1.3 to 2.1) | 127 (96 to 154) | 1.8 (1.4 to 2.2) | 3086 (2096 to 3765) | 40.3 (28.6 to 49) | 28 (17 to 39) | 0.4 (0.2 to 0.5) | 3113 (2111 to 3795) | 40.6 (28.9 to 49.6) | 223 (159 to 294) | 1.4 (1 to 1.8) | 231 (168 to 302) | 1.5 (1.1 to 2) | 5352 (3550 to 7019) | 32.3 (22 to 42.1) | 50 (30 to 72) | 0.3 (0.2 to 0.5) | 5402 (3579 to 7084) | 32.6 (22.3 to 42.5) |
|  | Male | 43 (31 to 55) | 0.6 (0.4 to 0.8) | 45 (32 to 57) | 0.7 (0.5 to 0.8) | 1009 (721 to 1281) | 13.5 (9.5 to 17.2) | 10 (6 to 14) | 0.1 (0.1 to 0.2) | 1018 (728 to 1292) | 13.6 (9.6 to 17.3) | 91 (62 to 121) | 0.6 (0.4 to 0.8) | 96 (64 to 127) | 0.7 (0.4 to 0.9) | 2066 (1419 to 2756) | 12.9 (8.8 to 17.2) | 20 (12 to 32) | 0.1 (0.1 to 0.2) | 2087 (1430 to 2781) | 13 (8.9 to 17.4) |
| Mozambique | Both | 71 (55 to 109) | 1.3 (1 to 1.9) | 74 (58 to 113) | 1.4 (1.1 to 2.1) | 1810 (1399 to 2749) | 28 (21.9 to 42.7) | 16 (10 to 25) | 0.3 (0.2 to 0.4) | 1825 (1411 to 2777) | 28.3 (22.1 to 43.1) | 107 (82 to 153) | 1.1 (0.9 to 1.6) | 111 (86 to 159) | 1.2 (1 to 1.8) | 2746 (2084 to 3913) | 24.2 (18.6 to 34.6) | 24 (15 to 36) | 0.2 (0.2 to 0.4) | 2770 (2102 to 3952) | 24.5 (18.9 to 35) |
|  | Female | 41 (30 to 72) | 1.4 (1 to 2.4) | 42 (32 to 74) | 1.5 (1.1 to 2.6) | 1038 (764 to 1829) | 30.7 (23 to 54) | 9 (5 to 16) | 0.3 (0.2 to 0.5) | 1047 (772 to 1847) | 31 (23.2 to 54.5) | 62 (43 to 93) | 1.2 (0.8 to 1.8) | 65 (45 to 97) | 1.3 (0.9 to 1.9) | 1532 (1042 to 2334) | 25 (17.3 to 37.8) | 14 (8 to 21) | 0.3 (0.2 to 0.4) | 1546 (1052 to 2353) | 25.2 (17.4 to 38.2) |
|  | Male | 30 (22 to 43) | 1.1 (0.8 to 1.6) | 32 (23 to 44) | 1.3 (0.9 to 1.8) | 772 (553 to 1098) | 25.1 (18.3 to 35.4) | 7 (4 to 10) | 0.3 (0.2 to 0.4) | 778 (558 to 1105) | 25.4 (18.5 to 35.7) | 45 (32 to 65) | 1 (0.8 to 1.4) | 46 (33 to 66) | 1.1 (0.8 to 1.6) | 1214 (846 to 1781) | 23.2 (16.5 to 33) | 10 (6 to 16) | 0.2 (0.1 to 0.3) | 1224 (853 to 1800) | 23.4 (16.6 to 33.4) |
| Myanmar | Both | 578 (437 to 748) | 2.6 (2 to 3.3) | 592 (450 to 767) | 2.8 (2.1 to 3.6) | 14708 (10958 to 19083) | 59.6 (44.9 to 77) | 129 (82 to 189) | 0.6 (0.4 to 0.8) | 14837 (11054 to 19225) | 60.2 (45.4 to 77.7) | 855 (694 to 1070) | 2 (1.6 to 2.5) | 885 (724 to 1107) | 2.1 (1.8 to 2.7) | 20181 (16395 to 25302) | 43.7 (35.6 to 54.7) | 191 (125 to 269) | 0.4 (0.3 to 0.6) | 20372 (16556 to 25585) | 44.1 (35.9 to 55.4) |
|  | Female | 364 (238 to 553) | 3.1 (2 to 4.6) | 374 (246 to 572) | 3.3 (2.2 to 4.9) | 9130 (5855 to 13896) | 70.3 (45.6 to 106.5) | 81 (46 to 131) | 0.7 (0.4 to 1.1) | 9211 (5911 to 14023) | 71 (46.1 to 107.6) | 532 (396 to 704) | 2.2 (1.6 to 2.9) | 553 (412 to 732) | 2.3 (1.7 to 3.1) | 12337 (9151 to 16339) | 47.6 (35.3 to 63) | 118 (73 to 174) | 0.5 (0.3 to 0.7) | 12455 (9229 to 16482) | 48.1 (35.6 to 63.6) |
|  | Male | 215 (127 to 324) | 2.1 (1.3 to 3) | 218 (130 to 327) | 2.2 (1.4 to 3.2) | 5578 (3259 to 8647) | 47.4 (28.1 to 71.8) | 48 (25 to 79) | 0.5 (0.2 to 0.7) | 5626 (3289 to 8719) | 47.9 (28.4 to 72.7) | 323 (256 to 452) | 1.8 (1.4 to 2.4) | 332 (264 to 465) | 1.9 (1.5 to 2.6) | 7844 (6097 to 11295) | 38.6 (30.5 to 54.2) | 73 (48 to 104) | 0.4 (0.3 to 0.6) | 7917 (6148 to 11396) | 38.9 (30.8 to 54.7) |
| Namibia | Both | 8 (6 to 9) | 1.1 (0.8 to 1.3) | 8 (6 to 10) | 1.2 (0.9 to 1.4) | 186 (133 to 233) | 24.3 (17.7 to 30.3) | 2 (1 to 2) | 0.2 (0.2 to 0.3) | 187 (135 to 236) | 24.5 (17.9 to 30.6) | 10 (8 to 14) | 0.8 (0.6 to 1) | 11 (9 to 15) | 0.8 (0.7 to 1.1) | 243 (191 to 310) | 16.9 (13.4 to 21.6) | 2 (2 to 3) | 0.2 (0.1 to 0.2) | 245 (193 to 313) | 17.1 (13.6 to 21.8) |
|  | Female | 5 (3 to 7) | 1.3 (0.9 to 1.8) | 5 (4 to 7) | 1.4 (1 to 1.9) | 126 (80 to 175) | 30.6 (19.6 to 42.3) | 1 (1 to 2) | 0.3 (0.2 to 0.5) | 127 (80 to 177) | 30.9 (19.9 to 42.7) | 7 (5 to 10) | 0.9 (0.6 to 1.2) | 8 (5 to 11) | 1 (0.7 to 1.4) | 161 (112 to 227) | 19.5 (13.7 to 27.5) | 2 (1 to 2) | 0.2 (0.1 to 0.3) | 162 (113 to 230) | 19.7 (13.9 to 27.8) |
|  | Male | 2 (2 to 3) | 0.7 (0.5 to 0.9) | 2 (2 to 3) | 0.8 (0.5 to 1) | 60 (41 to 76) | 16.9 (11.5 to 21.4) | 1 (0 to 1) | 0.2 (0.1 to 0.2) | 60 (41 to 76) | 17.1 (11.6 to 21.6) | 3 (2 to 4) | 0.6 (0.4 to 0.7) | 4 (3 to 4) | 0.7 (0.5 to 0.8) | 82 (60 to 105) | 13.3 (9.7 to 16.7) | 1 (0 to 1) | 0.1 (0.1 to 0.2) | 83 (60 to 106) | 13.4 (9.8 to 16.9) |
| Nepal | Both | 237 (176 to 408) | 2.6 (1.9 to 4.5) | 241 (180 to 413) | 2.8 (2.1 to 4.9) | 6177 (4569 to 10628) | 60 (44.6 to 103.5) | 53 (32 to 95) | 0.6 (0.4 to 1) | 6229 (4597 to 10689) | 60.5 (45.1 to 104.5) | 599 (451 to 809) | 2.9 (2.2 to 3.9) | 631 (473 to 850) | 3.1 (2.3 to 4.2) | 14473 (10710 to 19563) | 64.7 (48.3 to 86.7) | 133 (80 to 196) | 0.6 (0.4 to 0.9) | 14606 (10797 to 19727) | 65.3 (48.7 to 87.5) |
|  | Female | 155 (107 to 314) | 3.5 (2.4 to 7.1) | 158 (108 to 321) | 3.7 (2.5 to 7.8) | 4027 (2751 to 7983) | 80.4 (55.5 to 162.2) | 34 (20 to 69) | 0.8 (0.4 to 1.6) | 4061 (2772 to 8058) | 81.1 (56.1 to 163.3) | 394 (276 to 617) | 3.6 (2.5 to 5.6) | 413 (288 to 647) | 3.8 (2.7 to 6) | 9642 (6760 to 14817) | 81.7 (57.4 to 125.9) | 87 (51 to 144) | 0.8 (0.5 to 1.3) | 9729 (6830 to 14962) | 82.5 (58 to 127.5) |
|  | Male | 82 (58 to 125) | 1.8 (1.3 to 2.6) | 84 (59 to 127) | 1.9 (1.4 to 2.8) | 2150 (1489 to 3253) | 40.6 (28.7 to 62) | 18 (11 to 29) | 0.4 (0.2 to 0.6) | 2168 (1504 to 3278) | 41 (29 to 62.4) | 205 (126 to 290) | 2.1 (1.3 to 2.9) | 219 (134 to 307) | 2.3 (1.4 to 3.2) | 4831 (2953 to 6887) | 45.9 (28.4 to 65.1) | 46 (26 to 71) | 0.5 (0.3 to 0.7) | 4877 (2979 to 6941) | 46.3 (28.6 to 65.7) |
| Netherlands | Both | 990 (823 to 1279) | 4.8 (4 to 6.2) | 695 (638 to 722) | 3.3 (3.1 to 3.4) | 12143 (11094 to 12616) | 60.1 (54.9 to 62.6) | 240 (158 to 342) | 1.2 (0.8 to 1.7) | 12384 (11339 to 12870) | 61.3 (56 to 63.8) | 974 (821 to 1218) | 2.9 (2.4 to 3.6) | 747 (693 to 805) | 2.1 (2 to 2.3) | 11963 (11049 to 12945) | 37 (34.1 to 40) | 236 (161 to 329) | 0.7 (0.5 to 1) | 12199 (11284 to 13237) | 37.7 (34.8 to 41) |
|  | Female | 572 (458 to 797) | 4.7 (3.8 to 6.3) | 440 (418 to 462) | 3.5 (3.3 to 3.6) | 7262 (6892 to 7605) | 63.2 (60 to 66.1) | 130 (84 to 195) | 1.1 (0.7 to 1.6) | 7392 (7000 to 7747) | 64.2 (60.9 to 67.3) | 511 (418 to 682) | 2.7 (2.2 to 3.5) | 430 (391 to 475) | 2.2 (1.9 to 2.4) | 6506 (5861 to 7236) | 37.7 (33.9 to 42.1) | 117 (75 to 173) | 0.6 (0.4 to 0.9) | 6623 (5956 to 7386) | 38.4 (34.4 to 42.9) |
|  | Male | 419 (310 to 617) | 4.9 (3.7 to 7.2) | 255 (207 to 270) | 3.1 (2.5 to 3.3) | 4881 (3979 to 5162) | 56.3 (46 to 59.6) | 110 (66 to 176) | 1.3 (0.8 to 2) | 4991 (4056 to 5285) | 57.6 (46.8 to 61) | 463 (353 to 645) | 3.1 (2.4 to 4.2) | 317 (271 to 352) | 2.1 (1.8 to 2.3) | 5456 (4619 to 6087) | 36.2 (30.4 to 40.4) | 119 (75 to 182) | 0.8 (0.5 to 1.2) | 5576 (4733 to 6241) | 37 (31.1 to 41.3) |
| New Zealand | Both | 123 (97 to 165) | 3 (2.4 to 4) | 86 (75 to 92) | 2.1 (1.8 to 2.3) | 1624 (1419 to 1728) | 40.6 (35.6 to 43.1) | 30 (20 to 44) | 0.7 (0.5 to 1.1) | 1654 (1445 to 1762) | 41.3 (36.2 to 44) | 195 (144 to 261) | 2.5 (1.9 to 3.3) | 119 (103 to 130) | 1.5 (1.3 to 1.6) | 2019 (1761 to 2200) | 27.6 (24.2 to 30) | 49 (31 to 71) | 0.6 (0.4 to 0.9) | 2068 (1815 to 2264) | 28.2 (24.9 to 30.7) |
|  | Female | 75 (57 to 109) | 3.2 (2.5 to 4.7) | 53 (49 to 57) | 2.3 (2.1 to 2.4) | 962 (885 to 1033) | 44.3 (40.8 to 47.7) | 18 (11 to 27) | 0.8 (0.5 to 1.2) | 980 (903 to 1054) | 45 (41.5 to 48.5) | 123 (81 to 184) | 2.8 (1.9 to 4.2) | 67 (59 to 75) | 1.5 (1.4 to 1.7) | 1111 (983 to 1247) | 28.7 (25.4 to 32.1) | 31 (18 to 48) | 0.7 (0.4 to 1.1) | 1142 (1009 to 1281) | 29.4 (26 to 33) |
|  | Male | 48 (31 to 74) | 2.7 (1.7 to 4) | 33 (22 to 36) | 1.9 (1.3 to 2.1) | 662 (446 to 725) | 36.5 (24.7 to 39.9) | 12 (7 to 20) | 0.7 (0.4 to 1.1) | 674 (452 to 741) | 37.1 (25 to 40.6) | 72 (54 to 101) | 2 (1.5 to 2.9) | 52 (41 to 59) | 1.5 (1.1 to 1.7) | 908 (737 to 1016) | 26.4 (21.5 to 29.4) | 18 (11 to 28) | 0.5 (0.3 to 0.8) | 926 (750 to 1040) | 26.9 (21.9 to 30.1) |
| Nicaragua | Both | 57 (40 to 65) | 3.6 (2.6 to 4.1) | 59 (42 to 67) | 3.9 (2.8 to 4.4) | 1374 (954 to 1576) | 81.7 (57.5 to 93.3) | 12 (8 to 17) | 0.8 (0.5 to 1.1) | 1387 (964 to 1589) | 82.5 (58 to 94.2) | 86 (71 to 122) | 1.9 (1.6 to 2.7) | 90 (74 to 128) | 2 (1.7 to 2.9) | 1946 (1607 to 2740) | 42 (34.8 to 58.9) | 19 (12 to 29) | 0.4 (0.3 to 0.6) | 1965 (1623 to 2768) | 42.5 (35.1 to 59.5) |
|  | Female | 44 (30 to 51) | 5.3 (3.7 to 6.2) | 45 (31 to 52) | 5.7 (3.9 to 6.5) | 1055 (702 to 1229) | 121 (80.9 to 140.8) | 9 (6 to 13) | 1.1 (0.7 to 1.6) | 1065 (708 to 1240) | 122.1 (81.9 to 142.2) | 64 (51 to 96) | 2.7 (2.1 to 4) | 68 (54 to 102) | 2.9 (2.3 to 4.3) | 1439 (1138 to 2125) | 58.1 (46.1 to 85.7) | 14 (9 to 23) | 0.6 (0.4 to 0.9) | 1453 (1149 to 2150) | 58.7 (46.6 to 86.5) |
|  | Male | 13 (8 to 16) | 1.8 (1.2 to 2.1) | 14 (9 to 16) | 1.9 (1.3 to 2.2) | 319 (203 to 384) | 39.2 (25.3 to 47.2) | 3 (2 to 4) | 0.4 (0.2 to 0.6) | 322 (205 to 387) | 39.6 (25.5 to 47.6) | 22 (17 to 33) | 1 (0.8 to 1.6) | 22 (17 to 34) | 1.1 (0.9 to 1.7) | 507 (396 to 738) | 23.3 (18.3 to 34.5) | 5 (3 to 8) | 0.2 (0.1 to 0.4) | 512 (399 to 745) | 23.6 (18.5 to 34.8) |
| Niger | Both | 40 (31 to 53) | 1.6 (1.2 to 2) | 41 (32 to 55) | 1.7 (1.4 to 2.2) | 978 (761 to 1312) | 33.3 (26.3 to 44.6) | 9 (6 to 13) | 0.3 (0.2 to 0.5) | 987 (768 to 1325) | 33.7 (26.6 to 45) | 81 (61 to 115) | 1.2 (0.9 to 1.7) | 84 (64 to 118) | 1.3 (1 to 1.9) | 1987 (1505 to 2818) | 26.4 (20.1 to 37.3) | 18 (11 to 27) | 0.3 (0.2 to 0.4) | 2005 (1517 to 2847) | 26.6 (20.3 to 37.7) |
|  | Female | 21 (17 to 30) | 1.8 (1.4 to 2.4) | 22 (17 to 31) | 1.9 (1.5 to 2.6) | 520 (403 to 726) | 38.3 (30 to 53.2) | 5 (3 to 7) | 0.4 (0.2 to 0.6) | 525 (406 to 732) | 38.7 (30.2 to 53.7) | 47 (33 to 72) | 1.4 (1 to 2.1) | 49 (35 to 74) | 1.5 (1.1 to 2.3) | 1142 (806 to 1743) | 30 (21.2 to 46) | 10 (6 to 17) | 0.3 (0.2 to 0.5) | 1152 (813 to 1755) | 30.3 (21.4 to 46.5) |
|  | Male | 18 (13 to 26) | 1.3 (1 to 1.9) | 19 (13 to 26) | 1.5 (1 to 2) | 458 (313 to 661) | 29 (20 to 40.9) | 4 (2 to 6) | 0.3 (0.2 to 0.4) | 462 (315 to 666) | 29.3 (20.2 to 41.2) | 34 (25 to 56) | 1 (0.8 to 1.7) | 35 (25 to 57) | 1.1 (0.8 to 1.8) | 845 (593 to 1399) | 22.6 (16.3 to 37.1) | 8 (5 to 12) | 0.2 (0.1 to 0.4) | 852 (599 to 1413) | 22.8 (16.5 to 37.4) |
| Nigeria | Both | 532 (397 to 730) | 1.3 (1 to 1.7) | 572 (429 to 777) | 1.4 (1.1 to 1.9) | 11958 (8729 to 16704) | 26.3 (19.4 to 36.4) | 118 (73 to 174) | 0.3 (0.2 to 0.4) | 12076 (8811 to 16878) | 26.6 (19.6 to 36.8) | 872 (588 to 1310) | 1.2 (0.8 to 1.7) | 922 (628 to 1378) | 1.3 (0.9 to 1.9) | 20383 (13558 to 30805) | 24.4 (16.4 to 36.6) | 193 (112 to 307) | 0.3 (0.2 to 0.4) | 20577 (13703 to 31092) | 24.7 (16.6 to 36.9) |
|  | Female | 309 (224 to 435) | 1.5 (1.1 to 2.1) | 337 (246 to 472) | 1.7 (1.2 to 2.3) | 6696 (4787 to 9590) | 31.5 (22.6 to 45.1) | 68 (42 to 102) | 0.3 (0.2 to 0.5) | 6764 (4829 to 9683) | 31.8 (22.8 to 45.5) | 566 (357 to 912) | 1.5 (0.9 to 2.3) | 598 (378 to 955) | 1.6 (1.1 to 2.5) | 13287 (8121 to 21730) | 30.8 (19.2 to 49.7) | 125 (69 to 210) | 0.3 (0.2 to 0.5) | 13412 (8213 to 21917) | 31.1 (19.4 to 50.1) |
|  | Male | 223 (151 to 323) | 1 (0.7 to 1.5) | 235 (160 to 339) | 1.2 (0.8 to 1.6) | 5262 (3492 to 7775) | 21.7 (14.7 to 31.7) | 50 (30 to 78) | 0.2 (0.1 to 0.4) | 5312 (3523 to 7855) | 22 (14.8 to 32) | 306 (185 to 510) | 0.8 (0.5 to 1.4) | 324 (198 to 540) | 0.9 (0.6 to 1.5) | 7097 (4207 to 12021) | 17.7 (10.6 to 29.5) | 68 (37 to 116) | 0.2 (0.1 to 0.3) | 7165 (4251 to 12110) | 17.9 (10.7 to 29.7) |
| North Africa and Middle East | Both | 2879 (2339 to 3616) | 1.7 (1.4 to 2.2) | 2924 (2378 to 3722) | 1.8 (1.5 to 2.4) | 71173 (57661 to 87760) | 38.7 (31.4 to 48.4) | 637 (420 to 880) | 0.4 (0.2 to 0.5) | 71810 (58152 to 88352) | 39.1 (31.7 to 48.8) | 5461 (4724 to 6549) | 1.4 (1.2 to 1.7) | 5486 (4758 to 6700) | 1.4 (1.2 to 1.8) | 127677 (110243 to 150882) | 29 (25 to 34.8) | 1226 (831 to 1656) | 0.3 (0.2 to 0.4) | 128903 (111197 to 152265) | 29.3 (25.2 to 35.2) |
|  | Female | 1784 (1418 to 2203) | 2.2 (1.7 to 2.7) | 1817 (1440 to 2273) | 2.3 (1.8 to 3) | 43972 (34843 to 52768) | 48.8 (38.6 to 59.6) | 391 (252 to 553) | 0.5 (0.3 to 0.7) | 44363 (35118 to 53213) | 49.2 (39 to 60.1) | 3176 (2677 to 3900) | 1.6 (1.4 to 2) | 3224 (2739 to 3999) | 1.7 (1.4 to 2.1) | 74509 (61404 to 88540) | 34.3 (28.4 to 41.6) | 704 (472 to 966) | 0.3 (0.2 to 0.5) | 75213 (61937 to 89255) | 34.7 (28.7 to 42) |
|  | Male | 1095 (789 to 1562) | 1.3 (1 to 1.8) | 1107 (791 to 1580) | 1.4 (1 to 2) | 27201 (18908 to 38854) | 29 (20.5 to 41.4) | 246 (152 to 361) | 0.3 (0.2 to 0.4) | 27447 (19087 to 39126) | 29.3 (20.7 to 41.7) | 2285 (1683 to 2879) | 1.1 (0.8 to 1.5) | 2262 (1662 to 2866) | 1.2 (0.9 to 1.5) | 53168 (38660 to 66469) | 23.9 (17.4 to 30.1) | 523 (341 to 728) | 0.3 (0.2 to 0.4) | 53690 (39007 to 66964) | 24.1 (17.6 to 30.4) |
| North Korea | Both | 298 (223 to 411) | 1.9 (1.4 to 2.7) | 286 (216 to 400) | 1.9 (1.4 to 2.7) | 7106 (5408 to 9622) | 40.8 (30.8 to 56.3) | 68 (43 to 101) | 0.4 (0.3 to 0.6) | 7175 (5449 to 9722) | 41.2 (31.2 to 56.9) | 540 (420 to 709) | 1.8 (1.4 to 2.3) | 528 (410 to 696) | 1.7 (1.4 to 2.3) | 11889 (9136 to 15851) | 37.1 (28.6 to 49.4) | 124 (79 to 178) | 0.4 (0.3 to 0.6) | 12012 (9247 to 15997) | 37.5 (28.9 to 49.9) |
|  | Female | 184 (120 to 266) | 1.9 (1.2 to 2.8) | 175 (116 to 259) | 1.9 (1.2 to 2.8) | 4160 (2834 to 5930) | 40.2 (27.2 to 57.8) | 42 (24 to 65) | 0.4 (0.2 to 0.7) | 4203 (2860 to 6002) | 40.6 (27.5 to 58.3) | 305 (212 to 447) | 1.6 (1.1 to 2.4) | 301 (209 to 434) | 1.6 (1.1 to 2.3) | 6248 (4325 to 9047) | 34.1 (23.5 to 49.3) | 70 (42 to 108) | 0.4 (0.2 to 0.6) | 6318 (4366 to 9147) | 34.4 (23.8 to 49.8) |
|  | Male | 114 (81 to 175) | 2 (1.4 to 3) | 111 (80 to 169) | 2.1 (1.5 to 3.1) | 2946 (2034 to 4563) | 42.6 (30.8 to 65.1) | 26 (16 to 41) | 0.4 (0.3 to 0.7) | 2972 (2054 to 4600) | 43.1 (31.1 to 65.8) | 235 (154 to 367) | 2 (1.3 to 3) | 226 (151 to 347) | 2 (1.4 to 2.9) | 5641 (3608 to 8892) | 41.7 (27.3 to 65.5) | 54 (31 to 89) | 0.4 (0.3 to 0.7) | 5695 (3645 to 8978) | 42.1 (27.6 to 66.1) |
| Northern Mariana Islands | Both | 0 (0 to 0) | 1 (0.8 to 1.3) | 0 (0 to 0) | 1 (0.8 to 1.3) | 4 (3 to 5) | 20.3 (15.9 to 24.8) | 0 (0 to 0) | 0.2 (0.2 to 0.3) | 4 (3 to 5) | 20.5 (16.1 to 25.1) | 0 (0 to 1) | 0.9 (0.7 to 1.1) | 0 (0 to 0) | 0.8 (0.6 to 0.9) | 8 (7 to 10) | 15 (12.4 to 18.1) | 0 (0 to 0) | 0.2 (0.1 to 0.3) | 8 (7 to 10) | 15.2 (12.5 to 18.4) |
|  | Female | 0 (0 to 0) | 1.2 (0.8 to 1.6) | 0 (0 to 0) | 1.2 (0.8 to 1.6) | 2 (1 to 3) | 23.5 (16.3 to 30.7) | 0 (0 to 0) | 0.3 (0.2 to 0.4) | 2 (1 to 3) | 23.8 (16.5 to 31.1) | 0 (0 to 0) | 0.8 (0.6 to 1) | 0 (0 to 0) | 0.7 (0.5 to 0.8) | 4 (3 to 5) | 13.3 (11 to 17.8) | 0 (0 to 0) | 0.2 (0.1 to 0.3) | 4 (3 to 5) | 13.5 (11.2 to 18) |
|  | Male | 0 (0 to 0) | 0.9 (0.6 to 1.2) | 0 (0 to 0) | 0.9 (0.7 to 1.2) | 2 (1 to 3) | 17.7 (12.9 to 24.3) | 0 (0 to 0) | 0.2 (0.1 to 0.3) | 2 (1 to 3) | 17.9 (13.1 to 24.5) | 0 (0 to 0) | 1 (0.7 to 1.3) | 0 (0 to 0) | 0.9 (0.7 to 1.2) | 5 (3 to 6) | 16.8 (12.3 to 21.6) | 0 (0 to 0) | 0.2 (0.1 to 0.3) | 5 (3 to 6) | 17 (12.6 to 21.8) |
| Norway | Both | 216 (178 to 275) | 3.1 (2.6 to 3.9) | 141 (135 to 146) | 1.9 (1.9 to 2) | 2411 (2287 to 2492) | 36.5 (34.5 to 37.6) | 54 (35 to 78) | 0.8 (0.5 to 1.1) | 2465 (2328 to 2561) | 37.3 (35.2 to 38.6) | 247 (195 to 310) | 2.6 (2 to 3.2) | 131 (124 to 144) | 1.3 (1.2 to 1.4) | 2085 (1970 to 2300) | 23.1 (21.8 to 25.5) | 66 (42 to 93) | 0.7 (0.4 to 1) | 2151 (2028 to 2371) | 23.8 (22.5 to 26.2) |
|  | Female | 111 (95 to 142) | 2.7 (2.3 to 3.5) | 86 (83 to 88) | 2 (1.9 to 2.1) | 1399 (1349 to 1443) | 38.2 (36.7 to 39.5) | 26 (18 to 37) | 0.7 (0.4 to 0.9) | 1425 (1371 to 1468) | 38.9 (37.3 to 40.1) | 109 (87 to 141) | 2 (1.6 to 2.6) | 76 (72 to 80) | 1.4 (1.3 to 1.4) | 1149 (1086 to 1230) | 24.2 (22.8 to 26) | 26 (17 to 36) | 0.5 (0.3 to 0.7) | 1175 (1107 to 1258) | 24.7 (23.3 to 26.6) |
|  | Male | 105 (74 to 162) | 3.5 (2.5 to 5.2) | 55 (51 to 59) | 1.9 (1.7 to 2) | 1012 (931 to 1074) | 34.6 (31.9 to 36.6) | 29 (16 to 46) | 0.9 (0.6 to 1.5) | 1040 (951 to 1103) | 35.5 (32.7 to 37.6) | 138 (92 to 193) | 3.1 (2.1 to 4.4) | 55 (50 to 63) | 1.3 (1.2 to 1.4) | 936 (852 to 1087) | 22 (20.1 to 25.6) | 40 (23 to 63) | 0.9 (0.5 to 1.4) | 976 (880 to 1145) | 23 (20.7 to 26.9) |
| Oceania | Both | 40 (31 to 48) | 1.4 (1.1 to 1.7) | 39 (30 to 47) | 1.5 (1.2 to 1.8) | 1105 (831 to 1346) | 33.5 (25.8 to 40.4) | 9 (6 to 13) | 0.3 (0.2 to 0.4) | 1114 (838 to 1356) | 33.9 (26 to 40.7) | 82 (63 to 100) | 1.3 (1 to 1.6) | 80 (61 to 96) | 1.4 (1.1 to 1.6) | 2246 (1707 to 2748) | 30.5 (23.4 to 36.9) | 19 (12 to 26) | 0.3 (0.2 to 0.4) | 2265 (1720 to 2771) | 30.8 (23.7 to 37.3) |
|  | Female | 23 (17 to 29) | 1.7 (1.3 to 2.1) | 22 (16 to 28) | 1.7 (1.3 to 2.1) | 644 (462 to 821) | 39.8 (29.3 to 49.5) | 5 (3 to 8) | 0.4 (0.2 to 0.5) | 649 (466 to 827) | 40.2 (29.5 to 50) | 47 (33 to 60) | 1.5 (1.1 to 1.9) | 45 (32 to 57) | 1.5 (1.1 to 1.9) | 1299 (899 to 1694) | 35.3 (25.4 to 44.9) | 11 (6 to 16) | 0.3 (0.2 to 0.5) | 1310 (907 to 1707) | 35.6 (25.7 to 45.2) |
|  | Male | 17 (12 to 23) | 1.2 (0.9 to 1.6) | 17 (12 to 23) | 1.3 (0.9 to 1.7) | 461 (315 to 640) | 27.7 (19.7 to 37.6) | 4 (2 to 6) | 0.3 (0.2 to 0.4) | 465 (317 to 645) | 27.9 (19.9 to 37.9) | 35 (25 to 47) | 1.1 (0.8 to 1.5) | 35 (25 to 46) | 1.2 (0.9 to 1.5) | 947 (655 to 1288) | 25.9 (18.5 to 34) | 8 (5 to 12) | 0.3 (0.2 to 0.4) | 955 (660 to 1299) | 26.2 (18.7 to 34.3) |
| Oman | Both | 8 (6 to 11) | 1.3 (1 to 1.6) | 8 (6 to 11) | 1.4 (1.1 to 1.7) | 207 (155 to 263) | 27.9 (21.2 to 35.1) | 2 (1 to 3) | 0.3 (0.2 to 0.4) | 209 (156 to 265) | 28.2 (21.4 to 35.5) | 17 (13 to 21) | 0.9 (0.7 to 1.1) | 16 (12 to 19) | 1 (0.8 to 1.2) | 403 (303 to 501) | 18.7 (13.9 to 23) | 4 (2 to 6) | 0.2 (0.1 to 0.3) | 407 (306 to 506) | 18.9 (14.1 to 23.2) |
|  | Female | 4 (3 to 6) | 1.4 (1 to 1.9) | 4 (3 to 6) | 1.5 (1.1 to 2) | 102 (67 to 136) | 31.5 (20.6 to 41.8) | 1 (1 to 1) | 0.3 (0.2 to 0.5) | 103 (68 to 137) | 31.8 (20.8 to 42.2) | 7 (5 to 9) | 1 (0.7 to 1.3) | 7 (5 to 9) | 1.1 (0.7 to 1.3) | 175 (118 to 226) | 21 (13.9 to 26.7) | 2 (1 to 2) | 0.2 (0.1 to 0.3) | 177 (119 to 228) | 21.2 (14 to 27) |
|  | Male | 4 (3 to 6) | 1.2 (0.8 to 1.6) | 4 (3 to 6) | 1.3 (0.8 to 1.7) | 105 (68 to 153) | 25.3 (16.1 to 36.5) | 1 (1 to 1) | 0.3 (0.1 to 0.4) | 105 (69 to 154) | 25.6 (16.3 to 36.9) | 9 (6 to 13) | 0.9 (0.6 to 1.2) | 8 (6 to 12) | 0.9 (0.6 to 1.2) | 228 (158 to 315) | 17.7 (11.6 to 24.2) | 2 (1 to 3) | 0.2 (0.1 to 0.3) | 230 (160 to 318) | 17.9 (11.8 to 24.5) |
| Pakistan | Both | 2288 (1827 to 2825) | 4 (3.2 to 5) | 2345 (1874 to 2917) | 4.2 (3.4 to 5.3) | 57128 (45807 to 69036) | 94.9 (75.9 to 116.1) | 482 (312 to 696) | 0.8 (0.5 to 1.2) | 57609 (46160 to 69628) | 95.7 (76.8 to 117.1) | 4849 (3584 to 6382) | 4.6 (3.4 to 6.1) | 4897 (3654 to 6463) | 4.9 (3.7 to 6.5) | 124808 (91293 to 163575) | 103.4 (76.6 to 136.3) | 1038 (643 to 1506) | 0.9 (0.6 to 1.4) | 125845 (92215 to 164949) | 104.4 (77.5 to 137.8) |
|  | Female | 1861 (1452 to 2352) | 7.3 (5.7 to 9.4) | 1897 (1486 to 2442) | 7.6 (5.9 to 10.1) | 46705 (36007 to 57250) | 170.7 (133.6 to 212.5) | 386 (243 to 574) | 1.5 (0.9 to 2.2) | 47091 (36305 to 57685) | 172.2 (134.9 to 214.3) | 4042 (2818 to 5567) | 7.9 (5.5 to 11) | 4065 (2846 to 5637) | 8.4 (5.9 to 11.8) | 104271 (72976 to 143350) | 179.2 (125.9 to 246.6) | 857 (505 to 1303) | 1.6 (1 to 2.5) | 105127 (73709 to 144709) | 180.8 (127 to 248.6) |
|  | Male | 428 (322 to 591) | 1.4 (1 to 1.9) | 449 (338 to 611) | 1.5 (1.1 to 2) | 10423 (7881 to 14537) | 32 (24.2 to 44.4) | 96 (59 to 143) | 0.3 (0.2 to 0.5) | 10518 (7947 to 14665) | 32.4 (24.4 to 44.8) | 807 (569 to 1075) | 1.5 (1.1 to 2) | 832 (586 to 1102) | 1.6 (1.2 to 2.1) | 20537 (14525 to 27593) | 33.5 (23.6 to 44.7) | 181 (111 to 268) | 0.3 (0.2 to 0.5) | 20718 (14645 to 27805) | 33.8 (23.9 to 45.1) |
| Palestine | Both | 8 (6 to 10) | 0.9 (0.7 to 1.2) | 9 (6 to 11) | 1 (0.8 to 1.3) | 195 (145 to 244) | 21 (15.7 to 26.5) | 2 (1 to 3) | 0.2 (0.1 to 0.3) | 196 (147 to 247) | 21.2 (15.8 to 26.8) | 19 (14 to 22) | 0.8 (0.6 to 0.9) | 19 (15 to 22) | 0.9 (0.7 to 1) | 464 (344 to 534) | 17.9 (13.4 to 20.5) | 4 (3 to 6) | 0.2 (0.1 to 0.2) | 468 (346 to 539) | 18 (13.5 to 20.8) |
|  | Female | 5 (4 to 6) | 1 (0.8 to 1.4) | 5 (4 to 7) | 1.1 (0.9 to 1.4) | 112 (81 to 147) | 22.7 (16.6 to 29.9) | 1 (1 to 2) | 0.2 (0.1 to 0.3) | 113 (82 to 149) | 22.9 (16.8 to 30.2) | 11 (9 to 13) | 0.9 (0.7 to 1.1) | 11 (9 to 14) | 1 (0.8 to 1.2) | 262 (199 to 311) | 20.3 (15.4 to 24.4) | 2 (2 to 3) | 0.2 (0.1 to 0.3) | 264 (201 to 313) | 20.5 (15.6 to 24.6) |
|  | Male | 3 (2 to 5) | 0.8 (0.5 to 1.2) | 4 (2 to 5) | 0.9 (0.5 to 1.2) | 82 (48 to 117) | 19.1 (10.9 to 27.4) | 1 (0 to 1) | 0.2 (0.1 to 0.3) | 83 (49 to 118) | 19.3 (11 to 27.6) | 8 (5 to 10) | 0.7 (0.4 to 0.8) | 8 (5 to 9) | 0.7 (0.4 to 0.8) | 203 (119 to 249) | 15.2 (8.9 to 18.8) | 2 (1 to 3) | 0.1 (0.1 to 0.2) | 204 (120 to 252) | 15.4 (9 to 19) |
| Panama | Both | 41 (36 to 44) | 2.8 (2.5 to 3) | 43 (38 to 46) | 3 (2.6 to 3.2) | 913 (806 to 980) | 58.4 (51.6 to 62.6) | 9 (6 to 12) | 0.6 (0.4 to 0.8) | 922 (814 to 990) | 59 (52.1 to 63.3) | 56 (51 to 68) | 1.4 (1.3 to 1.7) | 59 (53 to 72) | 1.5 (1.3 to 1.8) | 1190 (1081 to 1426) | 30 (27.3 to 36.1) | 12 (8 to 17) | 0.3 (0.2 to 0.4) | 1202 (1092 to 1443) | 30.3 (27.5 to 36.5) |
|  | Female | 27 (25 to 30) | 3.7 (3.4 to 4) | 29 (26 to 31) | 3.9 (3.5 to 4.3) | 609 (552 to 660) | 78.4 (71.1 to 85.1) | 6 (4 to 8) | 0.8 (0.5 to 1.1) | 614 (557 to 666) | 79.2 (71.8 to 85.9) | 37 (33 to 43) | 1.8 (1.6 to 2.1) | 39 (35 to 45) | 1.9 (1.7 to 2.2) | 796 (695 to 919) | 39.4 (34.4 to 45.7) | 8 (6 to 11) | 0.4 (0.3 to 0.6) | 804 (703 to 928) | 39.8 (34.8 to 46.2) |
|  | Male | 14 (10 to 15) | 1.9 (1.4 to 2.1) | 15 (10 to 16) | 2.1 (1.5 to 2.3) | 304 (220 to 337) | 38.9 (28.2 to 43.1) | 3 (2 to 4) | 0.4 (0.3 to 0.6) | 307 (222 to 340) | 39.3 (28.5 to 43.5) | 18 (16 to 25) | 1 (0.8 to 1.3) | 19 (17 to 27) | 1 (0.9 to 1.4) | 394 (342 to 530) | 20.3 (17.7 to 27.5) | 4 (3 to 6) | 0.2 (0.1 to 0.3) | 398 (346 to 536) | 20.5 (17.8 to 27.8) |
| Papua New Guinea | Both | 26 (19 to 33) | 1.5 (1.1 to 1.8) | 25 (18 to 31) | 1.5 (1.1 to 1.9) | 745 (527 to 943) | 35.9 (25.8 to 44.7) | 6 (4 to 9) | 0.3 (0.2 to 0.5) | 751 (530 to 951) | 36.2 (26 to 45.1) | 57 (42 to 72) | 1.4 (1 to 1.7) | 54 (40 to 69) | 1.4 (1.1 to 1.8) | 1609 (1165 to 2066) | 32.3 (23.9 to 40.7) | 13 (8 to 19) | 0.3 (0.2 to 0.4) | 1622 (1175 to 2080) | 32.6 (24.1 to 41.1) |
|  | Female | 15 (10 to 20) | 1.8 (1.3 to 2.3) | 14 (10 to 19) | 1.8 (1.3 to 2.4) | 435 (299 to 572) | 43.4 (30.2 to 56.2) | 3 (2 to 5) | 0.4 (0.2 to 0.6) | 439 (301 to 578) | 43.8 (30.4 to 56.8) | 32 (21 to 44) | 1.6 (1.1 to 2.1) | 30 (20 to 41) | 1.7 (1.2 to 2.2) | 932 (606 to 1309) | 38.2 (25.8 to 51.7) | 7 (4 to 11) | 0.4 (0.2 to 0.5) | 939 (611 to 1319) | 38.5 (26 to 52.2) |
|  | Male | 11 (7 to 16) | 1.2 (0.8 to 1.7) | 11 (7 to 16) | 1.3 (0.8 to 1.8) | 310 (191 to 454) | 29 (18.7 to 41.5) | 2 (1 to 4) | 0.3 (0.2 to 0.4) | 312 (192 to 457) | 29.3 (18.9 to 41.8) | 24 (16 to 34) | 1.1 (0.8 to 1.6) | 24 (15 to 33) | 1.2 (0.8 to 1.6) | 677 (425 to 967) | 26.8 (17.3 to 36.8) | 6 (3 to 9) | 0.3 (0.1 to 0.4) | 682 (429 to 974) | 27 (17.5 to 37.1) |
| Paraguay | Both | 55 (42 to 63) | 2.6 (2 to 2.9) | 58 (45 to 66) | 2.8 (2.1 to 3.1) | 1271 (971 to 1447) | 54.9 (42.2 to 62.4) | 12 (8 to 17) | 0.6 (0.4 to 0.8) | 1283 (981 to 1461) | 55.5 (42.7 to 63.1) | 99 (81 to 130) | 1.9 (1.6 to 2.5) | 105 (85 to 137) | 2.1 (1.7 to 2.7) | 2266 (1819 to 2936) | 41.8 (33.6 to 54.3) | 22 (15 to 32) | 0.4 (0.3 to 0.6) | 2288 (1836 to 2966) | 42.2 (34 to 54.7) |
|  | Female | 40 (30 to 47) | 3.5 (2.6 to 4.1) | 42 (31 to 50) | 3.7 (2.8 to 4.4) | 921 (682 to 1087) | 76.5 (56.8 to 90.1) | 9 (6 to 12) | 0.8 (0.5 to 1.1) | 930 (688 to 1099) | 77.2 (57.5 to 91) | 70 (52 to 93) | 2.6 (1.9 to 3.4) | 74 (56 to 98) | 2.7 (2.1 to 3.6) | 1586 (1176 to 2118) | 56.8 (42.1 to 75.7) | 16 (10 to 23) | 0.6 (0.4 to 0.8) | 1601 (1187 to 2140) | 57.3 (42.5 to 76.4) |
|  | Male | 15 (11 to 18) | 1.5 (1 to 1.8) | 16 (11 to 19) | 1.6 (1.1 to 1.9) | 350 (247 to 425) | 31.6 (22.3 to 38) | 3 (2 to 5) | 0.3 (0.2 to 0.5) | 353 (249 to 430) | 31.9 (22.6 to 38.4) | 29 (22 to 44) | 1.2 (0.9 to 1.8) | 31 (23 to 46) | 1.3 (1 to 1.9) | 680 (502 to 1023) | 25.9 (19.3 to 38.5) | 7 (4 to 10) | 0.3 (0.2 to 0.4) | 687 (508 to 1033) | 26.1 (19.5 to 38.8) |
| Peru | Both | 517 (433 to 610) | 4.3 (3.6 to 5.1) | 537 (451 to 640) | 4.6 (3.9 to 5.5) | 12443 (10372 to 14461) | 97.5 (81.4 to 114.5) | 112 (73 to 155) | 0.9 (0.6 to 1.3) | 12555 (10468 to 14619) | 98.4 (82.2 to 115.4) | 954 (775 to 1210) | 3.1 (2.5 to 4) | 1005 (821 to 1282) | 3.3 (2.7 to 4.2) | 20927 (17009 to 26322) | 68.6 (55.8 to 86.1) | 209 (138 to 297) | 0.7 (0.5 to 1) | 21137 (17174 to 26594) | 69.2 (56.4 to 87.1) |
|  | Female | 370 (293 to 441) | 6 (4.8 to 7.2) | 384 (306 to 461) | 6.3 (5 to 7.6) | 8909 (6987 to 10607) | 137.1 (107.9 to 163.5) | 79 (49 to 114) | 1.3 (0.8 to 1.8) | 8988 (7051 to 10724) | 138.4 (108.8 to 165) | 651 (508 to 848) | 4.2 (3.2 to 5.4) | 686 (537 to 897) | 4.4 (3.4 to 5.7) | 14425 (11076 to 18599) | 92.5 (71.2 to 119.1) | 141 (88 to 207) | 0.9 (0.6 to 1.3) | 14566 (11175 to 18782) | 93.4 (71.9 to 120.3) |
|  | Male | 146 (116 to 202) | 2.5 (2 to 3.5) | 153 (122 to 212) | 2.7 (2.2 to 3.8) | 3534 (2789 to 4880) | 56.3 (44.8 to 78.4) | 33 (22 to 48) | 0.6 (0.4 to 0.8) | 3566 (2818 to 4924) | 56.9 (45.2 to 79) | 303 (225 to 421) | 2 (1.5 to 2.8) | 320 (239 to 444) | 2.2 (1.6 to 3) | 6502 (4749 to 9056) | 43.5 (31.9 to 60.5) | 68 (43 to 104) | 0.5 (0.3 to 0.7) | 6570 (4802 to 9149) | 44 (32.3 to 61.1) |
| Philippines | Both | 317 (289 to 354) | 1.1 (1 to 1.2) | 319 (291 to 356) | 1.2 (1.1 to 1.3) | 8004 (7337 to 8879) | 24.3 (22.1 to 27.2) | 71 (48 to 95) | 0.2 (0.2 to 0.3) | 8075 (7396 to 8962) | 24.5 (22.4 to 27.4) | 452 (374 to 779) | 0.7 (0.6 to 1.2) | 448 (374 to 785) | 0.7 (0.6 to 1.2) | 10858 (8966 to 18541) | 14.4 (11.9 to 24.9) | 102 (66 to 177) | 0.2 (0.1 to 0.3) | 10960 (9067 to 18708) | 14.5 (12.1 to 25.1) |
|  | Female | 160 (143 to 181) | 1.1 (1 to 1.2) | 161 (145 to 182) | 1.2 (1.1 to 1.3) | 3904 (3491 to 4392) | 23.4 (21 to 26.3) | 36 (24 to 48) | 0.2 (0.2 to 0.3) | 3940 (3523 to 4434) | 23.7 (21.2 to 26.6) | 232 (182 to 449) | 0.6 (0.5 to 1.2) | 234 (185 to 451) | 0.7 (0.5 to 1.3) | 5282 (4130 to 10394) | 13.3 (10.4 to 26) | 52 (33 to 97) | 0.1 (0.1 to 0.3) | 5334 (4166 to 10501) | 13.5 (10.6 to 26.3) |
|  | Male | 157 (138 to 183) | 1.1 (1 to 1.3) | 157 (138 to 186) | 1.2 (1 to 1.4) | 4100 (3613 to 4716) | 25.1 (22 to 29.6) | 36 (23 to 48) | 0.2 (0.2 to 0.3) | 4136 (3645 to 4750) | 25.4 (22.3 to 29.9) | 220 (172 to 394) | 0.7 (0.6 to 1.3) | 214 (169 to 387) | 0.7 (0.6 to 1.4) | 5576 (4375 to 9553) | 15.4 (12.1 to 27.5) | 50 (31 to 90) | 0.2 (0.1 to 0.3) | 5626 (4424 to 9633) | 15.6 (12.3 to 27.7) |
| Poland | Both | 2079 (1995 to 2223) | 4.6 (4.4 to 4.9) | 2264 (2169 to 2404) | 5 (4.8 to 5.4) | 45709 (43902 to 49167) | 99.6 (95.8 to 107.4) | 435 (291 to 586) | 1 (0.6 to 1.3) | 46144 (44313 to 49570) | 100.6 (96.6 to 108.3) | 2274 (2091 to 2450) | 3.2 (2.9 to 3.5) | 2537 (2335 to 2731) | 3.5 (3.2 to 3.8) | 45971 (42307 to 49557) | 67.6 (62.2 to 73) | 485 (330 to 645) | 0.7 (0.5 to 0.9) | 46456 (42792 to 50164) | 68.3 (62.9 to 73.8) |
|  | Female | 1654 (1573 to 1731) | 6.1 (5.8 to 6.3) | 1814 (1728 to 1896) | 6.6 (6.3 to 6.9) | 35652 (33869 to 37288) | 133.6 (126.9 to 139.7) | 340 (229 to 462) | 1.3 (0.8 to 1.7) | 35992 (34184 to 37711) | 134.9 (128.2 to 141) | 1613 (1466 to 1783) | 3.8 (3.5 to 4.2) | 1822 (1655 to 2011) | 4.2 (3.8 to 4.6) | 31582 (28477 to 34961) | 81.5 (73.7 to 90.5) | 338 (229 to 458) | 0.8 (0.5 to 1.1) | 31920 (28835 to 35361) | 82.4 (74.5 to 91.3) |
|  | Male | 425 (400 to 542) | 2.4 (2.2 to 3) | 450 (423 to 580) | 2.6 (2.4 to 3.3) | 10057 (9454 to 12791) | 52.3 (49.2 to 66.7) | 95 (63 to 128) | 0.5 (0.3 to 0.7) | 10152 (9545 to 12931) | 52.8 (49.7 to 67.4) | 661 (526 to 729) | 2.3 (1.8 to 2.6) | 715 (569 to 788) | 2.5 (2 to 2.8) | 14389 (11446 to 15915) | 49.5 (39.4 to 54.7) | 147 (97 to 197) | 0.5 (0.3 to 0.7) | 14535 (11582 to 16077) | 50 (39.9 to 55.3) |
| Portugal | Both | 393 (361 to 447) | 2.8 (2.6 to 3.2) | 372 (354 to 399) | 2.7 (2.5 to 2.9) | 6993 (6571 to 7416) | 49.7 (46.7 to 52.3) | 89 (61 to 119) | 0.6 (0.4 to 0.8) | 7082 (6681 to 7517) | 50.3 (47.2 to 53.1) | 484 (415 to 580) | 2 (1.7 to 2.4) | 441 (395 to 489) | 1.7 (1.5 to 1.9) | 6964 (6228 to 7704) | 31.6 (28.3 to 35) | 112 (77 to 155) | 0.5 (0.3 to 0.7) | 7076 (6340 to 7833) | 32.1 (28.6 to 35.5) |
|  | Female | 222 (202 to 254) | 2.7 (2.5 to 3.1) | 222 (209 to 235) | 2.7 (2.5 to 2.8) | 3881 (3655 to 4103) | 48.4 (45.5 to 51.3) | 48 (32 to 66) | 0.6 (0.4 to 0.8) | 3930 (3700 to 4161) | 49 (46.1 to 51.9) | 238 (199 to 323) | 1.6 (1.4 to 2) | 225 (201 to 253) | 1.5 (1.3 to 1.6) | 3279 (2914 to 3700) | 26.1 (23.2 to 29.6) | 53 (35 to 77) | 0.4 (0.3 to 0.5) | 3332 (2955 to 3755) | 26.5 (23.5 to 30) |
|  | Male | 171 (149 to 215) | 2.9 (2.5 to 3.5) | 151 (140 to 172) | 2.6 (2.4 to 3) | 3112 (2874 to 3403) | 50.8 (46.9 to 55.5) | 41 (27 to 57) | 0.7 (0.5 to 1) | 3153 (2918 to 3447) | 51.4 (47.6 to 56.3) | 246 (193 to 309) | 2.4 (1.9 to 3.1) | 216 (173 to 253) | 2.1 (1.6 to 2.4) | 3684 (2901 to 4289) | 38.2 (30 to 44.2) | 60 (37 to 85) | 0.6 (0.4 to 0.9) | 3744 (2945 to 4365) | 38.8 (30.4 to 44.8) |
| Puerto Rico | Both | 50 (43 to 55) | 1.3 (1.2 to 1.5) | 50 (44 to 54) | 1.3 (1.2 to 1.5) | 988 (863 to 1067) | 26.4 (23.2 to 28.5) | 11 (8 to 15) | 0.3 (0.2 to 0.4) | 999 (873 to 1081) | 26.7 (23.4 to 28.9) | 51 (46 to 58) | 0.7 (0.7 to 0.8) | 52 (47 to 57) | 0.7 (0.6 to 0.8) | 929 (848 to 1040) | 14.4 (13.1 to 16.1) | 11 (8 to 15) | 0.2 (0.1 to 0.2) | 940 (859 to 1052) | 14.5 (13.2 to 16.3) |
|  | Female | 30 (27 to 34) | 1.5 (1.3 to 1.7) | 30 (27 to 33) | 1.5 (1.3 to 1.6) | 593 (537 to 655) | 29.6 (26.9 to 32.6) | 7 (5 to 9) | 0.3 (0.2 to 0.5) | 600 (544 to 663) | 29.9 (27.2 to 33) | 30 (27 to 36) | 0.8 (0.7 to 0.9) | 31 (27 to 36) | 0.8 (0.7 to 0.9) | 556 (489 to 639) | 15.7 (13.9 to 18.1) | 7 (5 to 9) | 0.2 (0.1 to 0.2) | 563 (495 to 645) | 15.9 (14 to 18.3) |
|  | Male | 20 (14 to 22) | 1.2 (0.8 to 1.3) | 20 (14 to 22) | 1.2 (0.8 to 1.3) | 395 (288 to 438) | 22.8 (16.7 to 25.2) | 4 (3 to 6) | 0.3 (0.2 to 0.4) | 399 (291 to 444) | 23.1 (16.9 to 25.5) | 20 (18 to 25) | 0.7 (0.6 to 0.8) | 21 (18 to 24) | 0.6 (0.6 to 0.8) | 372 (322 to 436) | 12.7 (11 to 15.1) | 5 (3 to 6) | 0.2 (0.1 to 0.2) | 377 (326 to 442) | 12.9 (11.2 to 15.3) |
| Qatar | Both | 3 (1 to 3) | 3.2 (1.6 to 3.9) | 2 (1 to 3) | 3.5 (1.8 to 4.3) | 66 (38 to 83) | 58.5 (31.4 to 72.2) | 1 (0 to 1) | 0.6 (0.3 to 1) | 67 (38 to 84) | 59.2 (31.9 to 73) | 8 (6 to 11) | 1.1 (0.8 to 1.3) | 7 (5 to 9) | 1.1 (0.8 to 1.3) | 209 (162 to 263) | 19.7 (15.2 to 24.2) | 2 (1 to 3) | 0.2 (0.2 to 0.3) | 211 (164 to 266) | 19.9 (15.4 to 24.5) |
|  | Female | 1 (1 to 2) | 4.9 (2 to 6.5) | 1 (1 to 2) | 5.3 (2.2 to 6.9) | 34 (14 to 46) | 95.3 (39.4 to 125.5) | 0 (0 to 0) | 1 (0.4 to 1.6) | 35 (14 to 46) | 96.3 (39.9 to 126.7) | 3 (2 to 3) | 1.5 (1.1 to 2) | 2 (2 to 3) | 1.5 (1.1 to 2) | 61 (46 to 84) | 26.9 (20.3 to 36) | 1 (0 to 1) | 0.3 (0.2 to 0.5) | 62 (47 to 85) | 27.3 (20.6 to 36.5) |
|  | Male | 1 (1 to 2) | 1.8 (1 to 2.4) | 1 (1 to 1) | 2 (1 to 2.6) | 32 (17 to 43) | 36 (18.3 to 46.8) | 0 (0 to 0) | 0.4 (0.2 to 0.6) | 32 (17 to 44) | 36.5 (18.6 to 47.4) | 6 (4 to 8) | 0.9 (0.6 to 1.1) | 5 (3 to 6) | 0.9 (0.6 to 1.1) | 148 (105 to 196) | 16.5 (11.2 to 21.7) | 1 (1 to 2) | 0.2 (0.1 to 0.3) | 149 (106 to 198) | 16.7 (11.3 to 21.9) |
| Romania | Both | 719 (680 to 758) | 2.5 (2.4 to 2.6) | 742 (709 to 781) | 2.6 (2.5 to 2.8) | 17127 (15802 to 17937) | 58.4 (54.1 to 61.2) | 160 (109 to 214) | 0.6 (0.4 to 0.7) | 17286 (15910 to 18111) | 59 (54.6 to 61.7) | 579 (536 to 634) | 1.6 (1.5 to 1.7) | 625 (579 to 688) | 1.7 (1.5 to 1.8) | 12495 (11533 to 13495) | 35.8 (33 to 38.6) | 128 (87 to 174) | 0.3 (0.2 to 0.5) | 12622 (11636 to 13621) | 36.2 (33.3 to 39) |
|  | Female | 476 (448 to 505) | 3 (2.8 to 3.1) | 491 (463 to 519) | 3.1 (2.9 to 3.2) | 11023 (10358 to 11654) | 68.6 (64.3 to 72.4) | 105 (72 to 141) | 0.7 (0.4 to 0.9) | 11129 (10457 to 11764) | 69.3 (64.9 to 73.2) | 330 (296 to 369) | 1.6 (1.4 to 1.7) | 357 (321 to 400) | 1.6 (1.5 to 1.8) | 6875 (6164 to 7638) | 35.5 (31.9 to 39.5) | 73 (49 to 99) | 0.3 (0.2 to 0.5) | 6947 (6224 to 7723) | 35.9 (32.2 to 39.9) |
|  | Male | 243 (224 to 267) | 1.9 (1.8 to 2.2) | 251 (231 to 284) | 2 (1.9 to 2.4) | 6103 (5161 to 6515) | 45.9 (39.7 to 48.9) | 54 (37 to 72) | 0.4 (0.3 to 0.6) | 6158 (5205 to 6580) | 46.3 (40.1 to 49.4) | 249 (223 to 278) | 1.6 (1.4 to 1.8) | 268 (242 to 303) | 1.7 (1.5 to 1.9) | 5620 (4991 to 6267) | 36.4 (32.2 to 40.3) | 55 (37 to 75) | 0.4 (0.2 to 0.5) | 5675 (5037 to 6316) | 36.7 (32.5 to 40.7) |
| Russian Federation | Both | 3004 (2806 to 3255) | 1.6 (1.5 to 1.8) | 3035 (2826 to 3246) | 1.7 (1.6 to 1.8) | 65193 (60550 to 70300) | 34.7 (32.2 to 37.4) | 678 (459 to 907) | 0.4 (0.3 to 0.5) | 65871 (61239 to 71066) | 35 (32.6 to 37.8) | 3429 (3147 to 3845) | 1.5 (1.3 to 1.6) | 3169 (2935 to 3281) | 1.3 (1.2 to 1.4) | 64104 (58979 to 66380) | 27.8 (25.6 to 28.8) | 796 (547 to 1072) | 0.3 (0.2 to 0.5) | 64900 (59841 to 67226) | 28.1 (25.9 to 29.1) |
|  | Female | 2087 (1965 to 2287) | 1.7 (1.6 to 1.9) | 2128 (2047 to 2292) | 1.7 (1.7 to 1.9) | 42870 (40975 to 46597) | 35.4 (33.7 to 38.7) | 468 (315 to 629) | 0.4 (0.3 to 0.5) | 43338 (41440 to 47098) | 35.8 (34.2 to 39.1) | 2299 (2070 to 2686) | 1.5 (1.4 to 1.8) | 2085 (2014 to 2174) | 1.4 (1.3 to 1.4) | 39412 (37938 to 41255) | 27.9 (26.8 to 29.2) | 530 (358 to 721) | 0.4 (0.2 to 0.5) | 39942 (38473 to 41833) | 28.3 (27.1 to 29.6) |
|  | Male | 917 (768 to 1022) | 1.5 (1.2 to 1.6) | 906 (747 to 992) | 1.6 (1.3 to 1.7) | 22323 (18609 to 24674) | 32.9 (27.2 to 36.1) | 210 (137 to 286) | 0.3 (0.2 to 0.5) | 22533 (18791 to 24919) | 33.2 (27.5 to 36.5) | 1130 (927 to 1214) | 1.3 (1.1 to 1.4) | 1084 (889 to 1132) | 1.3 (1 to 1.3) | 24692 (20235 to 25809) | 27.4 (22.4 to 28.6) | 266 (179 to 354) | 0.3 (0.2 to 0.4) | 24958 (20507 to 26091) | 27.7 (22.7 to 29) |
| Rwanda | Both | 36 (27 to 53) | 1.3 (1 to 1.9) | 37 (28 to 55) | 1.5 (1.1 to 2.1) | 901 (669 to 1348) | 29.6 (22.3 to 43.5) | 8 (5 to 12) | 0.3 (0.2 to 0.4) | 909 (676 to 1358) | 29.9 (22.6 to 43.9) | 54 (41 to 77) | 1 (0.8 to 1.5) | 56 (44 to 80) | 1.2 (0.9 to 1.6) | 1335 (1021 to 1908) | 22.7 (17.4 to 32.3) | 12 (7 to 18) | 0.2 (0.1 to 0.3) | 1347 (1031 to 1926) | 22.9 (17.6 to 32.6) |
|  | Female | 22 (16 to 35) | 1.5 (1.1 to 2.4) | 22 (17 to 36) | 1.6 (1.2 to 2.6) | 549 (401 to 889) | 33.1 (24.4 to 53.2) | 5 (3 to 8) | 0.3 (0.2 to 0.5) | 554 (406 to 897) | 33.4 (24.7 to 53.7) | 35 (24 to 56) | 1.1 (0.8 to 1.8) | 36 (26 to 59) | 1.2 (0.9 to 2) | 849 (587 to 1385) | 24.8 (17.3 to 40.3) | 8 (5 to 13) | 0.2 (0.1 to 0.4) | 857 (593 to 1395) | 25.1 (17.5 to 40.7) |
|  | Male | 14 (9 to 20) | 1.2 (0.8 to 1.6) | 15 (10 to 21) | 1.3 (0.8 to 1.8) | 352 (223 to 516) | 25.5 (16.3 to 37.2) | 3 (2 to 5) | 0.3 (0.1 to 0.4) | 355 (224 to 521) | 25.8 (16.5 to 37.6) | 19 (14 to 27) | 0.9 (0.7 to 1.3) | 20 (14 to 28) | 1 (0.7 to 1.4) | 486 (341 to 687) | 19.7 (14.1 to 27.8) | 4 (3 to 7) | 0.2 (0.1 to 0.3) | 490 (343 to 694) | 19.9 (14.3 to 28) |
| Saint Lucia | Both | 3 (2 to 3) | 3.5 (2.5 to 3.7) | 3 (2 to 4) | 3.7 (2.7 to 4) | 71 (52 to 77) | 78 (57 to 84.3) | 1 (0 to 1) | 0.8 (0.5 to 1) | 72 (52 to 78) | 78.8 (57.6 to 85.2) | 2 (2 to 3) | 1 (0.9 to 1.5) | 2 (2 to 3) | 1.1 (0.9 to 1.5) | 47 (42 to 68) | 22.4 (19.7 to 32.2) | 0 (0 to 1) | 0.2 (0.1 to 0.3) | 48 (42 to 69) | 22.6 (19.9 to 32.6) |
|  | Female | 2 (1 to 2) | 3.6 (2.8 to 4) | 2 (1 to 2) | 3.8 (2.9 to 4.1) | 41 (32 to 45) | 83.1 (64.4 to 91.3) | 0 (0 to 1) | 0.8 (0.5 to 1.1) | 42 (32 to 46) | 83.9 (64.9 to 92.2) | 1 (1 to 2) | 1.1 (1 to 1.5) | 1 (1 to 2) | 1.2 (1 to 1.6) | 27 (23 to 36) | 25.1 (21.3 to 33.5) | 0 (0 to 0) | 0.2 (0.2 to 0.4) | 28 (23 to 37) | 25.3 (21.5 to 33.8) |
|  | Male | 1 (1 to 1) | 3.3 (1.8 to 3.8) | 1 (1 to 2) | 3.6 (1.9 to 4) | 30 (16 to 34) | 72.5 (38.8 to 81.6) | 0 (0 to 0) | 0.7 (0.4 to 1) | 30 (16 to 34) | 73.2 (39.1 to 82.4) | 1 (1 to 2) | 0.9 (0.8 to 1.7) | 1 (1 to 2) | 0.9 (0.8 to 1.8) | 20 (17 to 37) | 19.6 (16.5 to 36.1) | 0 (0 to 0) | 0.2 (0.1 to 0.4) | 20 (17 to 37) | 19.8 (16.6 to 36.4) |
| Saint Vincent and the Grenadines | Both | 2 (2 to 3) | 3.2 (2.3 to 3.5) | 3 (2 to 3) | 3.4 (2.5 to 3.7) | 53 (38 to 57) | 71.3 (51.4 to 77.7) | 1 (0 to 1) | 0.7 (0.4 to 0.9) | 53 (38 to 58) | 72 (51.8 to 78.5) | 2 (1 to 2) | 1.1 (1 to 1.5) | 2 (1 to 2) | 1.2 (1.1 to 1.6) | 36 (32 to 48) | 26.3 (23.9 to 35.2) | 0 (0 to 0) | 0.3 (0.2 to 0.4) | 36 (33 to 48) | 26.6 (24.1 to 35.5) |
|  | Female | 1 (1 to 2) | 3.5 (2.6 to 3.9) | 2 (1 to 2) | 3.7 (2.7 to 4.1) | 32 (23 to 35) | 79.8 (58.1 to 87.7) | 0 (0 to 0) | 0.8 (0.5 to 1) | 32 (24 to 36) | 80.5 (58.6 to 88.6) | 1 (1 to 1) | 1.2 (1.1 to 1.6) | 1 (1 to 1) | 1.3 (1.1 to 1.7) | 19 (17 to 25) | 29.1 (25.4 to 37.5) | 0 (0 to 0) | 0.3 (0.2 to 0.4) | 19 (17 to 25) | 29.4 (25.7 to 37.9) |
|  | Male | 1 (0 to 1) | 2.8 (1.5 to 3.1) | 1 (1 to 1) | 3 (1.6 to 3.3) | 21 (11 to 23) | 61.5 (34.1 to 69.2) | 0 (0 to 0) | 0.6 (0.3 to 0.8) | 21 (12 to 23) | 62.2 (34.5 to 69.8) | 1 (1 to 1) | 1 (0.9 to 1.5) | 1 (1 to 1) | 1.1 (1 to 1.5) | 17 (14 to 23) | 23.7 (20.7 to 33.3) | 0 (0 to 0) | 0.2 (0.2 to 0.3) | 17 (15 to 23) | 24 (20.9 to 33.6) |
| Samoa | Both | 1 (1 to 1) | 1.1 (0.8 to 1.4) | 1 (1 to 1) | 1.2 (0.9 to 1.5) | 22 (15 to 27) | 24.9 (17.6 to 31.5) | 0 (0 to 0) | 0.3 (0.2 to 0.4) | 22 (16 to 28) | 25.2 (17.7 to 31.9) | 1 (1 to 2) | 1.1 (0.8 to 1.4) | 1 (1 to 2) | 1.1 (0.9 to 1.4) | 32 (23 to 40) | 22.8 (16.9 to 28.9) | 0 (0 to 0) | 0.2 (0.1 to 0.3) | 32 (24 to 41) | 23 (17.1 to 29.2) |
|  | Female | 1 (0 to 1) | 1.3 (0.7 to 1.7) | 1 (0 to 1) | 1.3 (0.8 to 1.8) | 12 (7 to 17) | 28.1 (16.4 to 38.7) | 0 (0 to 0) | 0.3 (0.1 to 0.4) | 12 (7 to 17) | 28.4 (16.5 to 39.1) | 1 (1 to 1) | 1.2 (0.8 to 1.6) | 1 (1 to 1) | 1.2 (0.8 to 1.7) | 18 (12 to 25) | 25.7 (16.7 to 35) | 0 (0 to 0) | 0.3 (0.2 to 0.4) | 19 (12 to 26) | 26 (16.9 to 35.4) |
|  | Male | 0 (0 to 1) | 1 (0.7 to 1.4) | 0 (0 to 1) | 1.1 (0.8 to 1.5) | 9 (7 to 13) | 21.7 (16 to 29.6) | 0 (0 to 0) | 0.2 (0.1 to 0.3) | 9 (7 to 13) | 21.9 (16.2 to 29.9) | 1 (0 to 1) | 1 (0.7 to 1.3) | 1 (0 to 1) | 1.1 (0.8 to 1.4) | 13 (9 to 18) | 20 (14.1 to 26.5) | 0 (0 to 0) | 0.2 (0.1 to 0.3) | 13 (9 to 18) | 20.2 (14.2 to 26.7) |
| Sao Tome and Principe | Both | 1 (1 to 2) | 1.9 (1.6 to 2.6) | 1 (1 to 2) | 2 (1.7 to 2.8) | 30 (25 to 40) | 43.1 (36.3 to 57.9) | 0 (0 to 0) | 0.4 (0.3 to 0.6) | 30 (26 to 40) | 43.5 (36.8 to 58.3) | 2 (1 to 3) | 2 (1.5 to 2.7) | 2 (2 to 3) | 2.2 (1.6 to 2.9) | 49 (36 to 64) | 45.4 (33.8 to 59.8) | 0 (0 to 1) | 0.5 (0.3 to 0.7) | 49 (36 to 64) | 45.8 (34.2 to 60.3) |
|  | Female | 1 (1 to 1) | 2.3 (1.9 to 3.3) | 1 (1 to 1) | 2.5 (2 to 3.6) | 20 (16 to 27) | 53.7 (43 to 73.4) | 0 (0 to 0) | 0.5 (0.3 to 0.8) | 20 (16 to 27) | 54.2 (43.4 to 74) | 1 (1 to 2) | 2.4 (1.7 to 3.5) | 1 (1 to 2) | 2.6 (1.8 to 3.8) | 30 (21 to 44) | 54.2 (37.9 to 79.1) | 0 (0 to 0) | 0.5 (0.3 to 0.9) | 31 (21 to 44) | 54.8 (38.3 to 79.9) |
|  | Male | 0 (0 to 1) | 1.4 (1.1 to 2.1) | 0 (0 to 1) | 1.5 (1.2 to 2.2) | 10 (8 to 15) | 31.6 (25.4 to 45.5) | 0 (0 to 0) | 0.3 (0.2 to 0.5) | 10 (8 to 15) | 31.9 (25.6 to 46) | 1 (0 to 1) | 1.6 (1.1 to 2.2) | 1 (1 to 1) | 1.7 (1.2 to 2.4) | 18 (13 to 25) | 35.6 (24.2 to 48.5) | 0 (0 to 0) | 0.4 (0.2 to 0.5) | 18 (13 to 25) | 35.9 (24.4 to 48.9) |
| Saudi Arabia | Both | 92 (75 to 121) | 1.5 (1.3 to 2.1) | 94 (77 to 126) | 1.7 (1.4 to 2.2) | 2153 (1719 to 2825) | 32.6 (26.3 to 42.6) | 21 (13 to 29) | 0.3 (0.2 to 0.5) | 2173 (1732 to 2856) | 33 (26.6 to 43.1) | 233 (172 to 294) | 1.6 (1.1 to 1.9) | 213 (156 to 263) | 1.6 (1.2 to 1.9) | 5817 (4274 to 7365) | 30.8 (22.6 to 38) | 55 (35 to 78) | 0.3 (0.2 to 0.5) | 5872 (4320 to 7424) | 31.1 (22.8 to 38.5) |
|  | Female | 45 (34 to 63) | 1.7 (1.3 to 2.5) | 46 (36 to 65) | 1.9 (1.4 to 2.6) | 1051 (804 to 1419) | 38.2 (29.2 to 51.2) | 10 (6 to 15) | 0.4 (0.2 to 0.6) | 1061 (812 to 1432) | 38.6 (29.6 to 51.7) | 98 (67 to 129) | 1.5 (1.1 to 1.9) | 91 (63 to 119) | 1.6 (1.1 to 2) | 2559 (1731 to 3402) | 32.1 (22 to 41.3) | 23 (14 to 32) | 0.3 (0.2 to 0.5) | 2581 (1748 to 3432) | 32.4 (22.3 to 41.7) |
|  | Male | 47 (34 to 65) | 1.4 (1 to 2) | 48 (35 to 67) | 1.5 (1.1 to 2.1) | 1102 (790 to 1542) | 29 (20.9 to 40.7) | 11 (6 to 16) | 0.3 (0.2 to 0.5) | 1112 (797 to 1555) | 29.3 (21.1 to 41.2) | 135 (88 to 179) | 1.6 (1 to 2) | 122 (80 to 158) | 1.7 (1.1 to 2.1) | 3259 (2206 to 4338) | 30.2 (19.6 to 38.6) | 32 (19 to 48) | 0.4 (0.2 to 0.5) | 3291 (2229 to 4378) | 30.6 (19.9 to 39.1) |
| Senegal | Both | 47 (40 to 60) | 1.6 (1.3 to 2) | 50 (42 to 63) | 1.7 (1.4 to 2.2) | 1100 (917 to 1412) | 33 (27.6 to 42.4) | 10 (7 to 15) | 0.3 (0.2 to 0.5) | 1110 (926 to 1425) | 33.3 (27.8 to 42.7) | 93 (76 to 127) | 1.4 (1.1 to 1.9) | 98 (80 to 133) | 1.5 (1.2 to 2.1) | 2161 (1749 to 2961) | 29.6 (24.1 to 40.2) | 21 (14 to 30) | 0.3 (0.2 to 0.4) | 2181 (1767 to 2994) | 29.9 (24.3 to 40.7) |
|  | Female | 25 (20 to 34) | 1.7 (1.3 to 2.3) | 26 (21 to 36) | 1.8 (1.5 to 2.5) | 567 (451 to 797) | 34.9 (27.7 to 49) | 5 (3 to 8) | 0.4 (0.2 to 0.5) | 572 (454 to 804) | 35.3 (27.9 to 49.5) | 50 (38 to 68) | 1.5 (1.1 to 2) | 53 (41 to 73) | 1.6 (1.2 to 2.2) | 1161 (874 to 1611) | 30.8 (23.3 to 42.2) | 11 (7 to 17) | 0.3 (0.2 to 0.5) | 1172 (883 to 1624) | 31.1 (23.5 to 42.6) |
|  | Male | 23 (17 to 29) | 1.5 (1.1 to 1.9) | 24 (17 to 30) | 1.6 (1.1 to 2) | 533 (391 to 694) | 31.2 (22.9 to 40.4) | 5 (3 to 7) | 0.3 (0.2 to 0.5) | 538 (395 to 700) | 31.5 (23.2 to 40.8) | 43 (31 to 64) | 1.3 (1 to 2) | 45 (33 to 66) | 1.4 (1.1 to 2.1) | 1000 (729 to 1477) | 28.3 (20.8 to 42) | 9 (6 to 15) | 0.3 (0.2 to 0.5) | 1010 (737 to 1495) | 28.6 (21 to 42.5) |
| Serbia | Both | 409 (328 to 482) | 3.6 (2.9 to 4.3) | 430 (346 to 508) | 3.9 (3.2 to 4.6) | 9617 (7600 to 11303) | 80.8 (64.4 to 94.9) | 90 (58 to 123) | 0.8 (0.5 to 1.1) | 9706 (7666 to 11421) | 81.6 (65 to 95.8) | 473 (395 to 564) | 2.9 (2.4 to 3.5) | 524 (440 to 632) | 3.2 (2.7 to 3.9) | 9656 (7997 to 11176) | 61.1 (50.6 to 70.4) | 101 (67 to 137) | 0.6 (0.4 to 0.8) | 9757 (8063 to 11299) | 61.7 (51.2 to 71.1) |
|  | Female | 285 (227 to 348) | 4.6 (3.7 to 5.6) | 299 (240 to 367) | 4.9 (3.9 to 6) | 6620 (5192 to 8129) | 103.8 (81.8 to 126.8) | 62 (39 to 89) | 1 (0.6 to 1.4) | 6681 (5238 to 8204) | 104.7 (82.4 to 128) | 325 (278 to 422) | 3.6 (3.1 to 4.7) | 363 (311 to 475) | 4 (3.4 to 5.2) | 6466 (5494 to 8213) | 75.8 (64.3 to 95.6) | 68 (45 to 97) | 0.8 (0.5 to 1.1) | 6534 (5538 to 8298) | 76.6 (65 to 96.5) |
|  | Male | 124 (88 to 157) | 2.5 (1.8 to 3.2) | 131 (94 to 165) | 2.7 (2 to 3.5) | 2997 (2070 to 3800) | 54.6 (38.4 to 68.8) | 28 (17 to 40) | 0.5 (0.3 to 0.8) | 3025 (2092 to 3833) | 55.2 (38.8 to 69.4) | 148 (98 to 177) | 2 (1.3 to 2.4) | 161 (107 to 192) | 2.2 (1.5 to 2.6) | 3190 (2067 to 3838) | 43.6 (28.7 to 52.5) | 33 (19 to 47) | 0.4 (0.3 to 0.6) | 3223 (2089 to 3880) | 44.1 (29 to 53.1) |
| Seychelles | Both | 1 (1 to 1) | 1.6 (1.3 to 1.8) | 1 (1 to 1) | 1.7 (1.3 to 1.9) | 20 (16 to 23) | 35.4 (27.1 to 40.4) | 0 (0 to 0) | 0.4 (0.2 to 0.5) | 21 (16 to 24) | 35.7 (27.4 to 40.9) | 1 (1 to 2) | 1.4 (1.1 to 1.6) | 1 (1 to 2) | 1.3 (1.1 to 1.5) | 29 (23 to 33) | 26.4 (20.9 to 30) | 0 (0 to 0) | 0.3 (0.2 to 0.4) | 29 (23 to 33) | 26.7 (21.2 to 30.4) |
|  | Female | 0 (0 to 1) | 1.5 (1.1 to 1.8) | 1 (0 to 1) | 1.6 (1.1 to 1.8) | 10 (8 to 12) | 32.4 (23.7 to 38.2) | 0 (0 to 0) | 0.3 (0.2 to 0.5) | 10 (8 to 12) | 32.8 (24 to 38.6) | 1 (1 to 1) | 1.3 (1 to 1.5) | 1 (1 to 1) | 1.2 (1 to 1.5) | 13 (11 to 16) | 23.9 (19 to 28.5) | 0 (0 to 0) | 0.3 (0.2 to 0.4) | 14 (11 to 16) | 24.2 (19.2 to 28.8) |
|  | Male | 0 (0 to 1) | 1.8 (1.2 to 2.1) | 0 (0 to 1) | 1.9 (1.3 to 2.3) | 10 (7 to 12) | 39.2 (25.3 to 47.7) | 0 (0 to 0) | 0.4 (0.2 to 0.6) | 10 (7 to 12) | 39.6 (25.6 to 48.1) | 1 (0 to 1) | 1.5 (1 to 1.8) | 1 (0 to 1) | 1.4 (1 to 1.7) | 15 (11 to 19) | 28.9 (20.1 to 35) | 0 (0 to 0) | 0.3 (0.2 to 0.5) | 15 (11 to 19) | 29.2 (20.3 to 35.4) |
| Sierra Leone | Both | 27 (22 to 36) | 1.4 (1.2 to 1.9) | 29 (24 to 38) | 1.6 (1.3 to 2) | 621 (494 to 819) | 31.2 (24.9 to 40.9) | 6 (4 to 9) | 0.3 (0.2 to 0.5) | 627 (499 to 827) | 31.5 (25.2 to 41.3) | 39 (31 to 53) | 1.2 (1 to 1.7) | 41 (32 to 55) | 1.4 (1.1 to 1.8) | 920 (718 to 1249) | 26.5 (20.8 to 35.8) | 9 (5 to 13) | 0.3 (0.2 to 0.4) | 928 (723 to 1259) | 26.8 (20.9 to 36.1) |
|  | Female | 15 (11 to 21) | 1.7 (1.3 to 2.3) | 16 (12 to 23) | 1.8 (1.4 to 2.6) | 347 (263 to 487) | 36.3 (27.5 to 51.1) | 3 (2 to 5) | 0.4 (0.2 to 0.6) | 350 (265 to 492) | 36.7 (27.8 to 51.6) | 23 (17 to 32) | 1.5 (1.1 to 2.1) | 24 (18 to 34) | 1.6 (1.2 to 2.2) | 535 (387 to 753) | 31.3 (22.8 to 44.1) | 5 (3 to 8) | 0.3 (0.2 to 0.5) | 540 (391 to 761) | 31.6 (23 to 44.6) |
|  | Male | 12 (8 to 17) | 1.2 (0.8 to 1.7) | 13 (8 to 18) | 1.3 (0.9 to 1.9) | 275 (175 to 401) | 26.5 (17 to 38.5) | 3 (2 to 4) | 0.3 (0.2 to 0.4) | 277 (177 to 405) | 26.7 (17.2 to 38.8) | 16 (12 to 23) | 1 (0.8 to 1.5) | 17 (13 to 24) | 1.1 (0.8 to 1.6) | 384 (283 to 566) | 21.8 (16.3 to 31.9) | 4 (2 to 6) | 0.2 (0.1 to 0.4) | 388 (286 to 571) | 22 (16.5 to 32.2) |
| Singapore | Both | 38 (34 to 47) | 1.7 (1.6 to 2.2) | 35 (33 to 44) | 1.7 (1.5 to 2.1) | 787 (733 to 935) | 32.4 (30.2 to 39.6) | 9 (6 to 12) | 0.4 (0.3 to 0.5) | 796 (740 to 947) | 32.8 (30.6 to 40) | 95 (76 to 127) | 1.4 (1.1 to 1.9) | 63 (57 to 80) | 0.9 (0.9 to 1.2) | 1244 (1112 to 1503) | 17.9 (16 to 21.7) | 24 (16 to 35) | 0.4 (0.2 to 0.5) | 1268 (1135 to 1526) | 18.2 (16.3 to 22.1) |
|  | Female | 18 (16 to 21) | 1.5 (1.4 to 1.9) | 17 (15 to 20) | 1.5 (1.3 to 1.7) | 358 (330 to 397) | 28.5 (26.2 to 31.9) | 4 (3 to 6) | 0.3 (0.2 to 0.5) | 362 (333 to 400) | 28.8 (26.6 to 32.3) | 42 (32 to 60) | 1.1 (0.9 to 1.6) | 29 (26 to 33) | 0.8 (0.7 to 0.9) | 542 (473 to 639) | 14.7 (12.9 to 17.3) | 10 (7 to 16) | 0.3 (0.2 to 0.4) | 553 (483 to 650) | 15 (13.1 to 17.6) |
|  | Male | 20 (18 to 25) | 2 (1.7 to 2.7) | 18 (17 to 24) | 1.9 (1.7 to 2.6) | 429 (386 to 528) | 37 (33.6 to 47.8) | 5 (3 to 6) | 0.5 (0.3 to 0.6) | 434 (391 to 533) | 37.5 (34.1 to 48.4) | 53 (39 to 82) | 1.8 (1.3 to 2.9) | 34 (29 to 50) | 1.1 (1 to 1.7) | 702 (597 to 958) | 21.6 (18.4 to 30.2) | 14 (9 to 22) | 0.5 (0.3 to 0.7) | 716 (608 to 979) | 22 (18.7 to 30.9) |
| Slovakia | Both | 415 (347 to 470) | 6.8 (5.7 to 7.7) | 293 (243 to 330) | 4.8 (4 to 5.4) | 6037 (4917 to 6710) | 98.4 (80.3 to 109.5) | 98 (68 to 135) | 1.6 (1.1 to 2.2) | 6135 (5011 to 6821) | 100 (81.9 to 111.1) | 650 (453 to 867) | 7.1 (5 to 9.5) | 366 (274 to 413) | 4 (3 to 4.5) | 6841 (5267 to 7720) | 75.1 (58.1 to 84.7) | 157 (98 to 231) | 1.7 (1.1 to 2.5) | 6998 (5396 to 7896) | 76.9 (59.5 to 86.5) |
|  | Female | 299 (237 to 342) | 8.4 (6.6 to 9.6) | 202 (156 to 227) | 5.6 (4.4 to 6.3) | 4030 (3060 to 4506) | 115.4 (87.6 to 128.4) | 71 (48 to 101) | 2 (1.3 to 2.8) | 4101 (3110 to 4593) | 117.4 (89.1 to 130.9) | 490 (321 to 696) | 8.8 (5.9 to 12.3) | 257 (185 to 299) | 4.7 (3.4 to 5.4) | 4605 (3383 to 5400) | 89 (65.9 to 104.2) | 119 (70 to 184) | 2.1 (1.3 to 3.3) | 4725 (3463 to 5542) | 91.1 (67.5 to 107.1) |
|  | Male | 116 (81 to 148) | 4.6 (3.2 to 6) | 91 (64 to 114) | 3.7 (2.6 to 4.6) | 2007 (1390 to 2524) | 75.8 (52.7 to 95.6) | 27 (17 to 39) | 1.1 (0.7 to 1.6) | 2034 (1411 to 2560) | 76.9 (53.4 to 97) | 160 (102 to 227) | 4.5 (2.8 to 6.5) | 109 (71 to 132) | 3 (1.9 to 3.6) | 2235 (1495 to 2734) | 57.2 (38.4 to 69.1) | 38 (21 to 59) | 1.1 (0.6 to 1.7) | 2273 (1522 to 2773) | 58.2 (39.1 to 70.4) |
| Slovenia | Both | 131 (118 to 152) | 5.3 (4.8 to 6.3) | 128 (121 to 137) | 5.2 (4.9 to 5.6) | 2496 (2340 to 2692) | 98.9 (92.9 to 106.6) | 27 (18 to 38) | 1.1 (0.7 to 1.5) | 2523 (2365 to 2726) | 100 (93.9 to 108) | 184 (147 to 246) | 4 (3.2 to 5.2) | 151 (130 to 167) | 3.3 (2.9 to 3.7) | 2423 (2144 to 2695) | 58.6 (52 to 65.1) | 40 (26 to 58) | 0.9 (0.6 to 1.3) | 2463 (2181 to 2738) | 59.4 (52.8 to 66.1) |
|  | Female | 99 (86 to 118) | 6.4 (5.6 to 7.6) | 94 (87 to 100) | 6 (5.6 to 6.4) | 1757 (1628 to 1885) | 116.2 (107.9 to 124.9) | 20 (13 to 29) | 1.3 (0.9 to 1.9) | 1777 (1647 to 1907) | 117.5 (109.1 to 126.3) | 130 (94 to 190) | 4.7 (3.5 to 6.6) | 91 (79 to 106) | 3.3 (2.9 to 3.9) | 1372 (1189 to 1605) | 60.1 (52.2 to 69.9) | 29 (18 to 45) | 1.1 (0.7 to 1.6) | 1401 (1215 to 1640) | 61.2 (53.1 to 71.4) |
|  | Male | 32 (30 to 37) | 3.5 (3.2 to 3.9) | 34 (32 to 39) | 3.8 (3.5 to 4.3) | 739 (676 to 857) | 73 (66.9 to 83.7) | 7 (5 to 9) | 0.7 (0.5 to 1) | 746 (682 to 864) | 73.8 (67.5 to 84.5) | 54 (38 to 61) | 3 (2.1 to 3.4) | 60 (42 to 69) | 3.3 (2.3 to 3.8) | 1051 (780 to 1199) | 57.3 (42.7 to 65.2) | 11 (7 to 16) | 0.6 (0.4 to 0.9) | 1063 (788 to 1213) | 58 (43.1 to 65.9) |
| Solomon Islands | Both | 2 (1 to 2) | 1.3 (1 to 1.7) | 2 (1 to 2) | 1.4 (1.1 to 1.8) | 47 (36 to 60) | 31.1 (23.6 to 38.9) | 0 (0 to 1) | 0.3 (0.2 to 0.4) | 48 (36 to 60) | 31.4 (23.9 to 39.3) | 3 (3 to 4) | 1.2 (0.9 to 1.4) | 4 (3 to 4) | 1.2 (0.9 to 1.6) | 91 (68 to 112) | 26.4 (20 to 32.9) | 1 (0 to 1) | 0.3 (0.2 to 0.4) | 91 (68 to 113) | 26.7 (20.1 to 33.2) |
|  | Female | 1 (1 to 1) | 1.6 (1.1 to 2.1) | 1 (1 to 1) | 1.7 (1.2 to 2.2) | 25 (17 to 34) | 36.7 (25.7 to 48.9) | 0 (0 to 0) | 0.3 (0.2 to 0.5) | 25 (17 to 34) | 37.1 (25.9 to 49.4) | 2 (1 to 3) | 1.3 (0.9 to 1.8) | 2 (1 to 3) | 1.4 (1 to 1.9) | 53 (36 to 71) | 30.6 (21.2 to 41.2) | 0 (0 to 1) | 0.3 (0.2 to 0.4) | 53 (36 to 72) | 30.9 (21.3 to 41.6) |
|  | Male | 1 (1 to 1) | 1.1 (0.8 to 1.6) | 1 (1 to 1) | 1.2 (0.9 to 1.7) | 22 (15 to 32) | 26.6 (17.8 to 37.6) | 0 (0 to 0) | 0.3 (0.2 to 0.4) | 22 (15 to 32) | 26.8 (18 to 37.9) | 1 (1 to 2) | 1 (0.7 to 1.3) | 2 (1 to 2) | 1.1 (0.8 to 1.4) | 38 (26 to 50) | 22.4 (16 to 29.7) | 0 (0 to 0) | 0.2 (0.1 to 0.3) | 38 (27 to 51) | 22.6 (16.2 to 30.1) |
| Somalia | Both | 27 (17 to 41) | 1.2 (0.8 to 1.8) | 28 (17 to 41) | 1.3 (0.9 to 1.9) | 719 (426 to 1114) | 26.7 (16.9 to 40) | 6 (3 to 10) | 0.3 (0.2 to 0.4) | 725 (429 to 1125) | 27 (17.1 to 40.3) | 70 (51 to 102) | 1.2 (0.9 to 1.8) | 72 (52 to 105) | 1.4 (1 to 2) | 1841 (1317 to 2692) | 27.9 (20.3 to 40.5) | 16 (10 to 24) | 0.3 (0.2 to 0.4) | 1856 (1329 to 2716) | 28.2 (20.5 to 40.9) |
|  | Female | 16 (10 to 27) | 1.4 (0.9 to 2.3) | 16 (10 to 27) | 1.5 (1 to 2.5) | 420 (252 to 707) | 31.3 (19.5 to 51.8) | 4 (2 to 6) | 0.3 (0.2 to 0.5) | 424 (254 to 713) | 31.6 (19.7 to 52.2) | 44 (30 to 70) | 1.5 (1 to 2.4) | 45 (31 to 72) | 1.6 (1.1 to 2.6) | 1123 (757 to 1802) | 33.1 (22.6 to 53.2) | 10 (6 to 16) | 0.3 (0.2 to 0.5) | 1132 (763 to 1816) | 33.4 (22.8 to 53.7) |
|  | Male | 11 (6 to 17) | 1 (0.6 to 1.4) | 11 (7 to 17) | 1.1 (0.7 to 1.6) | 299 (158 to 476) | 22.1 (13.1 to 33.3) | 2 (1 to 4) | 0.2 (0.1 to 0.3) | 301 (159 to 480) | 22.4 (13.2 to 33.6) | 27 (19 to 38) | 1 (0.7 to 1.4) | 27 (20 to 39) | 1.1 (0.8 to 1.5) | 718 (504 to 1022) | 22.2 (15.9 to 31.6) | 6 (4 to 9) | 0.2 (0.1 to 0.3) | 724 (510 to 1029) | 22.4 (16.1 to 31.9) |
| South Africa | Both | 251 (192 to 290) | 1.2 (0.9 to 1.4) | 262 (202 to 304) | 1.3 (1 to 1.5) | 5874 (4647 to 6735) | 26.6 (20.7 to 30.8) | 56 (35 to 76) | 0.3 (0.2 to 0.4) | 5930 (4688 to 6795) | 26.9 (20.9 to 31) | 454 (352 to 504) | 1.1 (0.8 to 1.2) | 483 (372 to 535) | 1.2 (0.9 to 1.3) | 10313 (7999 to 11489) | 23.1 (17.9 to 25.6) | 101 (65 to 138) | 0.2 (0.2 to 0.3) | 10414 (8066 to 11607) | 23.3 (18.1 to 25.9) |
|  | Female | 182 (133 to 220) | 1.5 (1.1 to 1.9) | 192 (141 to 233) | 1.7 (1.2 to 2) | 4142 (3182 to 4954) | 33.3 (25.2 to 40.1) | 40 (25 to 58) | 0.3 (0.2 to 0.5) | 4182 (3217 to 5012) | 33.6 (25.4 to 40.5) | 318 (241 to 358) | 1.3 (1 to 1.4) | 343 (259 to 387) | 1.4 (1.1 to 1.6) | 6966 (5315 to 7867) | 27.1 (20.6 to 30.5) | 70 (45 to 97) | 0.3 (0.2 to 0.4) | 7036 (5367 to 7945) | 27.4 (20.8 to 30.8) |
|  | Male | 69 (47 to 92) | 0.8 (0.5 to 1) | 70 (48 to 94) | 0.8 (0.6 to 1.1) | 1732 (1238 to 2272) | 17.6 (12.2 to 23.4) | 16 (9 to 23) | 0.2 (0.1 to 0.3) | 1747 (1249 to 2294) | 17.8 (12.2 to 23.6) | 136 (93 to 171) | 0.8 (0.6 to 1) | 140 (96 to 174) | 0.9 (0.6 to 1.1) | 3348 (2264 to 4272) | 17.3 (11.8 to 21.8) | 31 (19 to 43) | 0.2 (0.1 to 0.3) | 3378 (2288 to 4301) | 17.5 (11.9 to 22) |
| South Asia | Both | 13074 (11230 to 18780) | 2.3 (2 to 3.4) | 13255 (11401 to 19086) | 2.5 (2.1 to 3.6) | 339843 (291735 to 479931) | 53.2 (45.7 to 76.3) | 2892 (1918 to 4364) | 0.5 (0.3 to 0.8) | 342734 (294232 to 483706) | 53.7 (46.1 to 76.9) | 36859 (28578 to 41294) | 2.8 (2.2 to 3.2) | 38087 (29570 to 42667) | 3 (2.4 to 3.4) | 914949 (702467 to 1028946) | 64.7 (49.7 to 72.6) | 8121 (5268 to 10917) | 0.6 (0.4 to 0.8) | 923070 (707826 to 1038260) | 65.4 (50.2 to 73.4) |
|  | Female | 8081 (6949 to 11750) | 3 (2.5 to 4.4) | 8186 (7040 to 11957) | 3.2 (2.7 to 4.7) | 210040 (180485 to 301708) | 68.6 (59 to 99.6) | 1771 (1166 to 2694) | 0.6 (0.4 to 1) | 211811 (181858 to 303916) | 69.2 (59.6 to 100.4) | 24588 (17854 to 28479) | 3.7 (2.7 to 4.3) | 25385 (18483 to 29382) | 3.9 (2.9 to 4.5) | 613567 (437657 to 711774) | 85.5 (61.2 to 99.2) | 5374 (3381 to 7371) | 0.8 (0.5 to 1.1) | 618941 (442199 to 717757) | 86.3 (61.9 to 100.1) |
|  | Male | 4993 (4027 to 7749) | 1.7 (1.4 to 2.6) | 5070 (4106 to 7863) | 1.8 (1.5 to 2.8) | 129803 (103695 to 201229) | 39.1 (31.5 to 60.5) | 1120 (713 to 1762) | 0.4 (0.2 to 0.6) | 130923 (104589 to 202829) | 39.4 (31.8 to 61.1) | 12271 (8696 to 15003) | 2 (1.4 to 2.4) | 12702 (8998 to 15511) | 2.1 (1.5 to 2.6) | 301382 (213026 to 366720) | 43.6 (30.9 to 53.2) | 2747 (1725 to 3795) | 0.4 (0.3 to 0.6) | 304129 (215123 to 370453) | 44 (31.2 to 53.8) |
| South Korea | Both | 2605 (2202 to 2965) | 9.3 (7.9 to 10.8) | 2567 (2183 to 2885) | 9.6 (8.2 to 11.2) | 60245 (50872 to 67522) | 189.4 (160.7 to 212.2) | 550 (372 to 755) | 1.9 (1.3 to 2.6) | 60795 (51295 to 68152) | 191.3 (162.3 to 214.6) | 7126 (5720 to 9280) | 8.6 (6.8 to 11.3) | 4959 (4506 to 5519) | 6 (5.4 to 6.6) | 85735 (77511 to 95568) | 100.1 (90.5 to 111.6) | 1630 (1110 to 2342) | 1.9 (1.3 to 2.8) | 87366 (78896 to 97235) | 102.1 (92.1 to 113.7) |
|  | Female | 1306 (1027 to 1525) | 7.9 (6.3 to 9.3) | 1313 (1043 to 1499) | 8.2 (6.5 to 9.7) | 28811 (22742 to 31870) | 160.4 (127.1 to 176.6) | 272 (179 to 387) | 1.6 (1.1 to 2.3) | 29083 (22950 to 32156) | 162 (128.3 to 178.2) | 3239 (2435 to 4821) | 6.6 (5 to 9.6) | 2334 (2062 to 2629) | 4.7 (4.2 to 5.3) | 36553 (32402 to 41594) | 77.3 (68.5 to 88.3) | 713 (437 to 1199) | 1.5 (0.9 to 2.4) | 37266 (33086 to 42427) | 78.8 (69.8 to 89.6) |
|  | Male | 1299 (1072 to 1557) | 11.7 (9.9 to 13.9) | 1255 (1048 to 1464) | 12.1 (10.4 to 14.1) | 31434 (25481 to 37314) | 233 (195.9 to 271.1) | 278 (181 to 395) | 2.3 (1.5 to 3.3) | 31712 (25687 to 37629) | 235.3 (197.8 to 274.3) | 3887 (2863 to 5496) | 11.3 (8.2 to 16.3) | 2625 (2245 to 3090) | 7.7 (6.6 to 9.1) | 49183 (41798 to 58276) | 128.3 (109.3 to 151.8) | 917 (577 to 1405) | 2.6 (1.6 to 4) | 50100 (42501 to 59285) | 130.9 (111.3 to 155.2) |
| South Sudan | Both | 27 (17 to 42) | 1.3 (0.8 to 1.9) | 29 (18 to 43) | 1.4 (0.9 to 2) | 677 (399 to 1056) | 27.5 (17 to 41.8) | 6 (3 to 10) | 0.3 (0.2 to 0.4) | 683 (403 to 1063) | 27.8 (17.3 to 42.2) | 38 (26 to 54) | 1.1 (0.8 to 1.6) | 39 (27 to 55) | 1.2 (0.9 to 1.7) | 990 (657 to 1410) | 25.1 (17.1 to 35.8) | 8 (5 to 13) | 0.2 (0.1 to 0.4) | 998 (663 to 1420) | 25.4 (17.2 to 36.1) |
|  | Female | 15 (9 to 25) | 1.6 (0.9 to 2.6) | 16 (9 to 27) | 1.7 (1 to 2.9) | 365 (203 to 633) | 34.2 (19.6 to 58.1) | 3 (2 to 6) | 0.3 (0.2 to 0.6) | 368 (205 to 638) | 34.5 (19.9 to 58.7) | 21 (13 to 33) | 1.4 (0.9 to 2.1) | 21 (13 to 34) | 1.5 (1 to 2.3) | 549 (337 to 876) | 30.1 (19 to 47.5) | 5 (3 to 8) | 0.3 (0.2 to 0.5) | 553 (340 to 882) | 30.4 (19.2 to 48) |
|  | Male | 12 (7 to 19) | 1 (0.6 to 1.5) | 13 (8 to 20) | 1.1 (0.7 to 1.6) | 312 (178 to 493) | 22.3 (13.4 to 34.1) | 3 (1 to 5) | 0.2 (0.1 to 0.4) | 315 (180 to 498) | 22.5 (13.6 to 34.4) | 17 (11 to 25) | 0.9 (0.6 to 1.3) | 17 (12 to 26) | 1 (0.7 to 1.4) | 441 (289 to 662) | 20.9 (14.1 to 30.7) | 4 (2 to 6) | 0.2 (0.1 to 0.3) | 445 (292 to 668) | 21.1 (14.2 to 31) |
| Southeast Asia | Both | 5663 (4401 to 6312) | 2.3 (1.8 to 2.6) | 5759 (4501 to 6415) | 2.5 (1.9 to 2.7) | 139977 (108773 to 156155) | 51.3 (40 to 57.2) | 1254 (829 to 1667) | 0.5 (0.3 to 0.7) | 141230 (109602 to 157642) | 51.8 (40.4 to 57.8) | 11569 (8848 to 12831) | 2.1 (1.6 to 2.3) | 11109 (8552 to 12134) | 2.1 (1.6 to 2.3) | 248073 (190817 to 271919) | 41.1 (31.7 to 45) | 2624 (1726 to 3562) | 0.5 (0.3 to 0.6) | 250697 (192488 to 274930) | 41.6 (32 to 45.5) |
|  | Female | 3029 (2240 to 3423) | 2.3 (1.7 to 2.6) | 3084 (2292 to 3489) | 2.5 (1.8 to 2.8) | 73522 (53819 to 83903) | 51.1 (37.6 to 58.1) | 671 (431 to 923) | 0.5 (0.3 to 0.7) | 74193 (54331 to 84824) | 51.6 (38 to 58.7) | 5664 (4432 to 6601) | 1.9 (1.5 to 2.2) | 5486 (4322 to 6265) | 1.8 (1.5 to 2.1) | 117956 (91493 to 134791) | 36.5 (28.4 to 41.5) | 1284 (842 to 1770) | 0.4 (0.3 to 0.6) | 119241 (92527 to 136280) | 36.9 (28.7 to 42) |
|  | Male | 2634 (1758 to 3108) | 2.3 (1.6 to 2.8) | 2675 (1791 to 3155) | 2.5 (1.7 to 3) | 66455 (44324 to 77588) | 51.7 (34.5 to 60.7) | 583 (357 to 815) | 0.5 (0.3 to 0.7) | 67038 (44727 to 78226) | 52.2 (34.9 to 61.3) | 5905 (3768 to 6914) | 2.4 (1.5 to 2.7) | 5623 (3633 to 6431) | 2.4 (1.6 to 2.7) | 130116 (83612 to 149537) | 46.7 (30.1 to 53.6) | 1340 (758 to 1876) | 0.5 (0.3 to 0.7) | 131456 (84497 to 151227) | 47.2 (30.5 to 54.2) |
| Southern Latin America | Both | 4948 (4689 to 5154) | 10.6 (10 to 11.1) | 5072 (4805 to 5255) | 11 (10.4 to 11.4) | 108736 (103414 to 112588) | 228.1 (217 to 236.2) | 1022 (708 to 1336) | 2.2 (1.5 to 2.8) | 109758 (104516 to 113786) | 230.3 (219.3 to 238.5) | 5762 (5303 to 6400) | 7 (6.4 to 7.8) | 5779 (5350 to 6370) | 7 (6.4 to 7.7) | 113578 (104748 to 124935) | 141.4 (130.1 to 155.5) | 1231 (843 to 1646) | 1.5 (1 to 2) | 114809 (105825 to 126332) | 142.9 (131.6 to 157.1) |
|  | Female | 3503 (3294 to 3679) | 13.4 (12.6 to 14.1) | 3611 (3417 to 3767) | 13.8 (13.1 to 14.4) | 76891 (71835 to 80359) | 295.9 (276.1 to 309) | 715 (491 to 942) | 2.7 (1.9 to 3.6) | 77606 (72528 to 81160) | 298.7 (278.8 to 312.2) | 3755 (3352 to 4271) | 8.1 (7.2 to 9.3) | 3831 (3453 to 4315) | 8.1 (7.3 to 9.1) | 74354 (66685 to 83879) | 170.1 (152.3 to 192.1) | 789 (538 to 1073) | 1.7 (1.2 to 2.4) | 75143 (67431 to 84675) | 171.8 (154 to 193.9) |
|  | Male | 1445 (1257 to 1540) | 7.1 (6.2 to 7.5) | 1461 (1273 to 1537) | 7.3 (6.4 to 7.7) | 31845 (27715 to 33611) | 148 (128.8 to 155.9) | 308 (210 to 420) | 1.5 (1 to 2) | 32152 (27898 to 33924) | 149.5 (129.6 to 157.5) | 2008 (1772 to 2381) | 5.6 (5 to 6.7) | 1948 (1753 to 2261) | 5.5 (5 to 6.4) | 39224 (35053 to 45870) | 108.2 (96.9 to 126.8) | 442 (294 to 597) | 1.2 (0.8 to 1.7) | 39666 (35422 to 46375) | 109.5 (97.9 to 128.1) |
| Southern Sub-Saharan Africa | Both | 334 (267 to 383) | 1.3 (1 to 1.4) | 349 (277 to 402) | 1.4 (1.1 to 1.6) | 7898 (6361 to 9004) | 27.1 (21.8 to 31.1) | 74 (48 to 102) | 0.3 (0.2 to 0.4) | 7972 (6413 to 9073) | 27.4 (21.9 to 31.4) | 578 (466 to 646) | 1.1 (0.9 to 1.3) | 612 (497 to 689) | 1.2 (1 to 1.4) | 13411 (10788 to 14976) | 23.8 (19.2 to 26.5) | 128 (85 to 175) | 0.2 (0.2 to 0.3) | 13540 (10880 to 15148) | 24 (19.3 to 26.8) |
|  | Female | 229 (180 to 273) | 1.5 (1.2 to 1.8) | 241 (189 to 290) | 1.6 (1.3 to 2) | 5255 (4153 to 6273) | 32.5 (25.5 to 38.8) | 51 (32 to 71) | 0.3 (0.2 to 0.5) | 5305 (4189 to 6330) | 32.9 (25.8 to 39.2) | 397 (310 to 469) | 1.3 (1 to 1.5) | 426 (334 to 504) | 1.4 (1.1 to 1.7) | 8873 (6950 to 10356) | 27.4 (21.4 to 32) | 88 (58 to 121) | 0.3 (0.2 to 0.4) | 8961 (7012 to 10451) | 27.7 (21.6 to 32.3) |
|  | Male | 105 (74 to 135) | 0.9 (0.6 to 1.1) | 108 (75 to 138) | 1 (0.7 to 1.2) | 2643 (1881 to 3367) | 20.1 (14.1 to 25.7) | 24 (14 to 34) | 0.2 (0.1 to 0.3) | 2667 (1898 to 3397) | 20.3 (14.2 to 26) | 181 (132 to 226) | 0.8 (0.6 to 1) | 185 (137 to 231) | 0.9 (0.7 to 1.1) | 4538 (3350 to 5653) | 18.4 (13.6 to 22.9) | 41 (26 to 57) | 0.2 (0.1 to 0.3) | 4579 (3375 to 5705) | 18.6 (13.8 to 23.1) |
| Spain | Both | 2658 (2289 to 3057) | 4.6 (4 to 5.3) | 1742 (1563 to 1808) | 3 (2.7 to 3.1) | 31442 (28274 to 32681) | 55.8 (50 to 58) | 661 (455 to 885) | 1.1 (0.8 to 1.5) | 32103 (28752 to 33388) | 57 (51.2 to 59.2) | 3379 (2515 to 4571) | 3.2 (2.4 to 4.2) | 1813 (1668 to 1964) | 1.7 (1.6 to 1.8) | 26708 (24515 to 28961) | 29.4 (27 to 31.9) | 869 (562 to 1263) | 0.8 (0.6 to 1.2) | 27577 (25348 to 29993) | 30.2 (27.6 to 32.9) |
|  | Female | 1751 (1505 to 2053) | 5.1 (4.4 to 6) | 1093 (1035 to 1147) | 3.2 (3 to 3.3) | 18637 (17510 to 19595) | 58.2 (54.6 to 61.1) | 435 (294 to 589) | 1.3 (0.9 to 1.7) | 19072 (17872 to 20005) | 59.5 (55.7 to 62.4) | 1865 (1239 to 2815) | 2.9 (2.1 to 4.1) | 982 (886 to 1101) | 1.5 (1.4 to 1.7) | 13162 (11882 to 14730) | 25.8 (23.2 to 28.9) | 468 (266 to 747) | 0.8 (0.5 to 1.2) | 13630 (12263 to 15230) | 26.5 (23.9 to 29.7) |
|  | Male | 907 (710 to 1200) | 3.8 (3 to 5) | 649 (525 to 686) | 2.8 (2.2 to 2.9) | 12806 (10165 to 13517) | 52.4 (41.6 to 55.2) | 226 (146 to 333) | 0.9 (0.6 to 1.4) | 13031 (10354 to 13770) | 53.3 (42.5 to 56.3) | 1514 (1050 to 2226) | 3.5 (2.5 to 5.1) | 831 (694 to 931) | 1.9 (1.6 to 2.1) | 13546 (11470 to 15244) | 33.6 (28.8 to 37.9) | 401 (237 to 632) | 0.9 (0.6 to 1.5) | 13947 (11937 to 15789) | 34.5 (29.4 to 39.1) |
| Sri Lanka | Both | 427 (231 to 500) | 4.1 (2.2 to 4.8) | 435 (233 to 508) | 4.4 (2.4 to 5.1) | 10051 (5381 to 11756) | 88.6 (47.4 to 103.7) | 93 (46 to 134) | 0.9 (0.4 to 1.3) | 10144 (5432 to 11844) | 89.5 (47.9 to 104.8) | 526 (402 to 663) | 2.2 (1.7 to 2.7) | 471 (366 to 582) | 2 (1.6 to 2.5) | 10134 (7799 to 12558) | 39.7 (30.7 to 49.3) | 124 (80 to 180) | 0.5 (0.3 to 0.7) | 10257 (7876 to 12713) | 40.3 (31.1 to 50) |
|  | Female | 230 (110 to 279) | 4.4 (2.2 to 5.3) | 229 (111 to 274) | 4.5 (2.3 to 5.4) | 5495 (2542 to 6617) | 96.3 (45.4 to 115.6) | 51 (23 to 74) | 0.9 (0.4 to 1.4) | 5546 (2565 to 6674) | 97.2 (45.8 to 116.7) | 287 (193 to 392) | 2.1 (1.4 to 2.8) | 253 (177 to 327) | 1.9 (1.3 to 2.4) | 5434 (3708 to 7160) | 38.8 (26.7 to 50.8) | 68 (39 to 105) | 0.5 (0.3 to 0.7) | 5501 (3753 to 7246) | 39.3 (27.1 to 51.5) |
|  | Male | 197 (102 to 240) | 3.9 (2 to 4.7) | 206 (106 to 251) | 4.2 (2.2 to 5.2) | 4556 (2459 to 5519) | 81.2 (42.8 to 98.5) | 43 (21 to 62) | 0.8 (0.4 to 1.2) | 4598 (2483 to 5570) | 82 (43.2 to 99.6) | 239 (155 to 332) | 2.3 (1.5 to 3.1) | 219 (146 to 293) | 2.1 (1.5 to 2.9) | 4700 (3097 to 6448) | 41.3 (27.7 to 56.4) | 56 (32 to 88) | 0.5 (0.3 to 0.8) | 4756 (3135 to 6508) | 41.8 (28 to 56.9) |
| Sudan | Both | 135 (97 to 192) | 1.5 (1.1 to 2.1) | 141 (102 to 199) | 1.6 (1.2 to 2.3) | 3270 (2292 to 4697) | 33.1 (23.6 to 47.4) | 30 (19 to 46) | 0.3 (0.2 to 0.5) | 3299 (2313 to 4737) | 33.4 (23.8 to 47.8) | 204 (157 to 268) | 1.2 (0.9 to 1.6) | 213 (165 to 282) | 1.3 (1 to 1.7) | 4908 (3690 to 6510) | 25.9 (19.8 to 33.9) | 46 (30 to 66) | 0.3 (0.2 to 0.4) | 4953 (3730 to 6558) | 26.2 (20 to 34.3) |
|  | Female | 76 (51 to 109) | 1.8 (1.2 to 2.6) | 80 (54 to 115) | 1.9 (1.3 to 2.8) | 1823 (1204 to 2604) | 38.9 (25.9 to 56.1) | 17 (10 to 26) | 0.4 (0.2 to 0.6) | 1840 (1216 to 2634) | 39.3 (26.2 to 56.6) | 103 (70 to 153) | 1.3 (0.9 to 2) | 107 (73 to 160) | 1.4 (1 to 2.2) | 2502 (1652 to 3738) | 28.4 (19.1 to 42.6) | 23 (13 to 37) | 0.3 (0.2 to 0.5) | 2525 (1669 to 3780) | 28.7 (19.3 to 42.9) |
|  | Male | 59 (36 to 95) | 1.2 (0.8 to 2) | 61 (38 to 98) | 1.3 (0.9 to 2.1) | 1446 (813 to 2370) | 27.8 (16.4 to 45.1) | 13 (7 to 22) | 0.3 (0.2 to 0.5) | 1460 (821 to 2390) | 28.1 (16.6 to 45.4) | 101 (68 to 143) | 1.1 (0.8 to 1.5) | 106 (72 to 149) | 1.2 (0.8 to 1.7) | 2406 (1516 to 3544) | 23.9 (15.7 to 34.1) | 23 (14 to 35) | 0.2 (0.1 to 0.4) | 2429 (1531 to 3572) | 24.1 (15.9 to 34.5) |
| Suriname | Both | 8 (5 to 9) | 3.1 (2.2 to 3.4) | 8 (6 to 9) | 3.2 (2.3 to 3.6) | 187 (132 to 208) | 69.1 (48.5 to 76.4) | 2 (1 to 2) | 0.7 (0.4 to 0.9) | 189 (133 to 210) | 69.8 (49 to 77.3) | 6 (5 to 9) | 1.1 (1 to 1.6) | 7 (6 to 10) | 1.2 (1 to 1.7) | 149 (129 to 217) | 24.9 (21.6 to 36.2) | 1 (1 to 2) | 0.2 (0.2 to 0.4) | 150 (131 to 219) | 25.1 (21.9 to 36.6) |
|  | Female | 4 (3 to 5) | 3.3 (2.5 to 3.7) | 5 (3 to 5) | 3.5 (2.7 to 3.9) | 106 (82 to 119) | 76 (58.7 to 85.3) | 1 (1 to 1) | 0.7 (0.5 to 1) | 107 (83 to 120) | 76.8 (59.4 to 86.1) | 4 (3 to 5) | 1.1 (1 to 1.6) | 4 (3 to 5) | 1.2 (1 to 1.6) | 82 (70 to 113) | 25.9 (22.1 to 35.4) | 1 (1 to 1) | 0.3 (0.2 to 0.4) | 83 (71 to 114) | 26.2 (22.3 to 35.8) |
|  | Male | 3 (2 to 4) | 2.8 (1.5 to 3.3) | 3 (2 to 4) | 3 (1.6 to 3.5) | 82 (44 to 95) | 62.3 (33.2 to 71.8) | 1 (0 to 1) | 0.6 (0.3 to 0.9) | 82 (44 to 96) | 62.9 (33.6 to 72.6) | 3 (2 to 5) | 1.1 (0.9 to 1.9) | 3 (2 to 5) | 1.1 (0.9 to 2) | 67 (53 to 116) | 23.8 (19.2 to 41.3) | 1 (0 to 1) | 0.2 (0.2 to 0.4) | 67 (54 to 116) | 24 (19.4 to 41.8) |
| Swaziland | Both | 4 (3 to 5) | 1.4 (1.1 to 1.7) | 4 (3 to 5) | 1.5 (1.2 to 1.9) | 93 (74 to 117) | 30.1 (24.1 to 37.5) | 1 (1 to 1) | 0.3 (0.2 to 0.4) | 94 (74 to 118) | 30.4 (24.3 to 37.8) | 6 (4 to 8) | 1.2 (0.9 to 1.6) | 6 (5 to 9) | 1.3 (1 to 1.8) | 152 (112 to 207) | 26.1 (19.3 to 35.3) | 1 (1 to 2) | 0.3 (0.2 to 0.4) | 153 (113 to 208) | 26.4 (19.5 to 35.7) |
|  | Female | 2 (2 to 3) | 1.6 (1.2 to 2) | 3 (2 to 3) | 1.7 (1.3 to 2.2) | 58 (44 to 77) | 33.9 (25.9 to 45) | 1 (0 to 1) | 0.3 (0.2 to 0.5) | 58 (44 to 78) | 34.2 (26.2 to 45.6) | 4 (3 to 6) | 1.3 (0.9 to 1.9) | 4 (3 to 6) | 1.5 (1 to 2.1) | 93 (58 to 140) | 27.7 (17.7 to 41.3) | 1 (1 to 1) | 0.3 (0.2 to 0.5) | 94 (59 to 141) | 28 (17.9 to 41.8) |
|  | Male | 1 (1 to 2) | 1.1 (0.8 to 1.5) | 1 (1 to 2) | 1.2 (0.8 to 1.6) | 35 (24 to 46) | 25 (17.3 to 32.7) | 0 (0 to 0) | 0.2 (0.2 to 0.4) | 36 (25 to 47) | 25.2 (17.4 to 33) | 2 (1 to 3) | 1 (0.7 to 1.3) | 2 (1 to 3) | 1.1 (0.7 to 1.4) | 59 (40 to 84) | 22.7 (15.5 to 31.7) | 0 (0 to 1) | 0.2 (0.1 to 0.3) | 60 (40 to 84) | 23 (15.6 to 32) |
| Sweden | Both | 3186 (2816 to 3457) | 19.8 (17.4 to 21.6) | 538 (498 to 559) | 3.4 (3.1 to 3.5) | 9322 (8514 to 9685) | 64.9 (58.1 to 67.5) | 957 (683 to 1275) | 6 (4.2 to 8) | 10279 (9321 to 10803) | 70.9 (63.4 to 74.4) | 531 (473 to 582) | 2.5 (2.2 to 2.8) | 511 (463 to 547) | 2.3 (2.1 to 2.5) | 8021 (7179 to 8638) | 41 (36.3 to 44.3) | 125 (85 to 166) | 0.6 (0.4 to 0.8) | 8146 (7287 to 8777) | 41.7 (36.9 to 45.1) |
|  | Female | 2122 (1842 to 2356) | 23.8 (20.2 to 26.6) | 361 (342 to 376) | 4 (3.7 to 4.1) | 6085 (5707 to 6374) | 78.4 (72.4 to 82.5) | 629 (447 to 837) | 7.2 (5 to 9.7) | 6714 (6265 to 7092) | 85.6 (79 to 90.7) | 309 (273 to 346) | 2.7 (2.4 to 3.1) | 322 (290 to 353) | 2.6 (2.4 to 2.9) | 4886 (4337 to 5396) | 47.6 (42.3 to 52.9) | 69 (47 to 94) | 0.6 (0.4 to 0.8) | 4956 (4404 to 5484) | 48.2 (42.8 to 53.7) |
|  | Male | 1064 (871 to 1212) | 15 (12.3 to 17.1) | 178 (151 to 189) | 2.6 (2.2 to 2.8) | 3237 (2649 to 3446) | 49.6 (40.3 to 52.5) | 328 (225 to 452) | 4.6 (3.2 to 6.4) | 3565 (2906 to 3820) | 54.2 (44 to 58.2) | 222 (173 to 261) | 2.3 (1.8 to 2.8) | 189 (149 to 206) | 1.9 (1.5 to 2.1) | 3134 (2382 to 3442) | 34.1 (25.7 to 37.5) | 56 (37 to 77) | 0.6 (0.4 to 0.8) | 3190 (2427 to 3502) | 34.7 (26.2 to 38.1) |
| Switzerland | Both | 429 (339 to 561) | 3.9 (3.1 to 5.1) | 284 (250 to 300) | 2.5 (2.2 to 2.6) | 4794 (4218 to 5049) | 45.7 (40.4 to 48.2) | 105 (69 to 150) | 1 (0.6 to 1.4) | 4899 (4310 to 5165) | 46.7 (41.3 to 49.2) | 342 (288 to 431) | 2 (1.6 to 2.5) | 273 (246 to 300) | 1.5 (1.3 to 1.6) | 4205 (3731 to 4654) | 25.7 (22.7 to 28.6) | 83 (56 to 118) | 0.5 (0.3 to 0.7) | 4288 (3812 to 4749) | 26.2 (23.2 to 29.2) |
|  | Female | 266 (203 to 390) | 4.1 (3.2 to 5.8) | 190 (174 to 204) | 2.8 (2.6 to 3) | 3079 (2836 to 3293) | 51.5 (47.4 to 55.1) | 62 (39 to 95) | 1 (0.6 to 1.5) | 3141 (2900 to 3359) | 52.5 (48.3 to 56.2) | 183 (149 to 259) | 1.9 (1.5 to 2.5) | 158 (141 to 178) | 1.5 (1.3 to 1.7) | 2330 (2068 to 2635) | 26.7 (23.7 to 30.3) | 42 (28 to 64) | 0.4 (0.3 to 0.6) | 2372 (2103 to 2686) | 27.2 (24.1 to 30.8) |
|  | Male | 163 (114 to 242) | 3.7 (2.6 to 5.5) | 93 (68 to 102) | 2.1 (1.6 to 2.3) | 1715 (1289 to 1883) | 38.4 (28.9 to 42) | 44 (26 to 73) | 1 (0.6 to 1.6) | 1758 (1322 to 1929) | 39.3 (29.6 to 43.1) | 158 (113 to 226) | 2.1 (1.5 to 2.9) | 115 (88 to 134) | 1.5 (1.1 to 1.7) | 1875 (1459 to 2188) | 24.8 (19.5 to 29.1) | 41 (24 to 63) | 0.5 (0.3 to 0.8) | 1915 (1493 to 2236) | 25.4 (19.9 to 29.7) |
| Syria | Both | 41 (30 to 48) | 0.8 (0.6 to 0.9) | 42 (31 to 49) | 0.9 (0.6 to 1) | 985 (740 to 1182) | 17.6 (13.2 to 21) | 9 (6 to 12) | 0.2 (0.1 to 0.2) | 995 (747 to 1195) | 17.8 (13.3 to 21.2) | 77 (55 to 95) | 0.6 (0.5 to 0.8) | 79 (56 to 96) | 0.7 (0.5 to 0.8) | 1818 (1314 to 2241) | 13.5 (9.7 to 16.6) | 17 (11 to 25) | 0.1 (0.1 to 0.2) | 1836 (1329 to 2261) | 13.6 (9.8 to 16.8) |
|  | Female | 19 (12 to 24) | 0.8 (0.5 to 1) | 20 (12 to 24) | 0.9 (0.5 to 1) | 458 (293 to 582) | 17.3 (11 to 21.7) | 4 (2 to 6) | 0.2 (0.1 to 0.2) | 462 (296 to 587) | 17.4 (11.1 to 21.9) | 33 (20 to 41) | 0.5 (0.3 to 0.7) | 33 (21 to 41) | 0.6 (0.4 to 0.7) | 775 (486 to 987) | 11.9 (7.5 to 15) | 7 (4 to 10) | 0.1 (0.1 to 0.2) | 782 (491 to 996) | 12.1 (7.6 to 15.2) |
|  | Male | 22 (13 to 28) | 0.8 (0.5 to 1) | 22 (14 to 29) | 0.9 (0.5 to 1.1) | 527 (328 to 702) | 18 (11.1 to 23.7) | 5 (3 to 7) | 0.2 (0.1 to 0.3) | 532 (331 to 708) | 18.2 (11.2 to 23.9) | 45 (30 to 60) | 0.7 (0.5 to 1) | 46 (30 to 60) | 0.8 (0.5 to 1) | 1043 (689 to 1398) | 15 (10 to 20) | 10 (6 to 15) | 0.2 (0.1 to 0.2) | 1054 (697 to 1411) | 15.2 (10.1 to 20.2) |
| Taiwan | Both | 423 (395 to 467) | 2.6 (2.5 to 2.9) | 336 (322 to 370) | 2.2 (2.1 to 2.4) | 8030 (7691 to 8788) | 46.9 (45 to 51.3) | 105 (74 to 138) | 0.6 (0.5 to 0.8) | 8135 (7790 to 8896) | 47.6 (45.6 to 52) | 1281 (1017 to 1593) | 3.3 (2.7 to 4.1) | 768 (661 to 828) | 2 (1.7 to 2.2) | 14911 (13122 to 16090) | 39.3 (34.6 to 42.3) | 338 (226 to 470) | 0.9 (0.6 to 1.2) | 15250 (13454 to 16450) | 40.1 (35.4 to 43.3) |
|  | Female | 213 (192 to 241) | 2.8 (2.6 to 3.2) | 165 (157 to 175) | 2.3 (2.2 to 2.4) | 3878 (3659 to 4105) | 48.7 (46 to 51.4) | 53 (37 to 71) | 0.7 (0.5 to 0.9) | 3932 (3706 to 4164) | 49.4 (46.7 to 52.3) | 651 (493 to 900) | 3.2 (2.4 to 4.4) | 380 (337 to 420) | 1.8 (1.6 to 2) | 7014 (6172 to 7783) | 35.1 (30.8 to 38.8) | 172 (113 to 257) | 0.8 (0.6 to 1.3) | 7186 (6325 to 7973) | 35.9 (31.6 to 39.7) |
|  | Male | 210 (193 to 240) | 2.5 (2.3 to 2.8) | 171 (161 to 193) | 2.1 (2 to 2.4) | 4151 (3910 to 4644) | 45.3 (42.8 to 51) | 52 (36 to 70) | 0.6 (0.4 to 0.8) | 4204 (3960 to 4687) | 45.9 (43.3 to 51.6) | 631 (457 to 832) | 3.5 (2.6 to 4.6) | 388 (298 to 429) | 2.2 (1.7 to 2.4) | 7897 (6266 to 8750) | 43.8 (34.7 to 48.5) | 167 (106 to 242) | 0.9 (0.6 to 1.3) | 8064 (6400 to 8932) | 44.7 (35.3 to 49.5) |
| Tajikistan | Both | 18 (14 to 23) | 0.6 (0.5 to 0.8) | 19 (15 to 24) | 0.7 (0.6 to 0.9) | 429 (335 to 556) | 14.5 (11.4 to 18.9) | 4 (3 to 6) | 0.1 (0.1 to 0.2) | 433 (338 to 561) | 14.7 (11.5 to 19.1) | 27 (23 to 34) | 0.5 (0.5 to 0.7) | 28 (24 to 35) | 0.6 (0.5 to 0.7) | 705 (609 to 890) | 12.4 (10.7 to 16) | 6 (4 to 8) | 0.1 (0.1 to 0.2) | 711 (613 to 899) | 12.5 (10.8 to 16.1) |
|  | Female | 11 (8 to 14) | 0.7 (0.5 to 0.9) | 12 (9 to 15) | 0.7 (0.6 to 1) | 250 (185 to 334) | 15.6 (11.6 to 20.9) | 2 (1 to 4) | 0.2 (0.1 to 0.2) | 252 (187 to 338) | 15.8 (11.7 to 21) | 14 (12 to 18) | 0.5 (0.4 to 0.7) | 15 (12 to 19) | 0.6 (0.5 to 0.7) | 379 (307 to 487) | 12.6 (10.3 to 16.3) | 3 (2 to 5) | 0.1 (0.1 to 0.2) | 382 (310 to 491) | 12.7 (10.4 to 16.4) |
|  | Male | 7 (6 to 11) | 0.6 (0.5 to 0.9) | 8 (6 to 12) | 0.6 (0.5 to 1) | 179 (138 to 274) | 13.3 (10.2 to 20.7) | 2 (1 to 3) | 0.1 (0.1 to 0.2) | 181 (139 to 276) | 13.4 (10.3 to 20.8) | 13 (10 to 18) | 0.5 (0.5 to 0.8) | 13 (11 to 19) | 0.6 (0.5 to 0.9) | 326 (266 to 470) | 12.2 (10 to 17.8) | 3 (2 to 4) | 0.1 (0.1 to 0.2) | 329 (268 to 475) | 12.3 (10.1 to 18) |
| Tanzania | Both | 121 (92 to 175) | 1.2 (0.9 to 1.7) | 127 (97 to 182) | 1.3 (1 to 1.8) | 2958 (2192 to 4267) | 25.6 (19.5 to 36.9) | 27 (17 to 40) | 0.3 (0.2 to 0.4) | 2985 (2214 to 4307) | 25.9 (19.7 to 37.2) | 233 (182 to 326) | 1 (0.8 to 1.5) | 246 (194 to 346) | 1.1 (0.9 to 1.6) | 5623 (4359 to 7808) | 22.8 (17.7 to 31.8) | 52 (33 to 74) | 0.2 (0.1 to 0.3) | 5675 (4406 to 7880) | 23 (17.9 to 32.2) |
|  | Female | 77 (56 to 123) | 1.5 (1.1 to 2.4) | 80 (59 to 130) | 1.6 (1.2 to 2.6) | 1877 (1359 to 3028) | 32 (23.5 to 51.6) | 17 (10 to 28) | 0.3 (0.2 to 0.5) | 1894 (1371 to 3055) | 32.4 (23.7 to 52.1) | 148 (111 to 229) | 1.2 (0.9 to 1.9) | 156 (118 to 241) | 1.4 (1 to 2.1) | 3538 (2602 to 5474) | 27.4 (20.3 to 42.2) | 33 (20 to 51) | 0.3 (0.2 to 0.4) | 3571 (2624 to 5512) | 27.7 (20.5 to 42.5) |
|  | Male | 45 (32 to 62) | 0.9 (0.6 to 1.2) | 47 (34 to 65) | 1 (0.7 to 1.3) | 1081 (747 to 1520) | 19.1 (13.5 to 26.4) | 10 (6 to 15) | 0.2 (0.1 to 0.3) | 1091 (755 to 1530) | 19.3 (13.7 to 26.7) | 85 (59 to 121) | 0.8 (0.6 to 1.1) | 90 (62 to 127) | 0.9 (0.6 to 1.3) | 2085 (1437 to 2979) | 17.7 (12.2 to 25.1) | 19 (11 to 30) | 0.2 (0.1 to 0.3) | 2104 (1451 to 3008) | 17.8 (12.3 to 25.3) |
| Thailand | Both | 2071 (1413 to 2448) | 6 (4 to 7.1) | 2096 (1441 to 2487) | 6.3 (4.3 to 7.5) | 50587 (34858 to 59624) | 131.1 (90.6 to 155.1) | 452 (277 to 630) | 1.3 (0.8 to 1.8) | 51039 (35206 to 60091) | 132.4 (91.6 to 156.4) | 5139 (3091 to 6163) | 5.2 (3.2 to 6.3) | 4672 (2867 to 5455) | 4.8 (3 to 5.6) | 102089 (61501 to 120375) | 101.9 (61.8 to 120.1) | 1168 (656 to 1673) | 1.2 (0.7 to 1.7) | 103257 (62000 to 121699) | 103.1 (62.3 to 121.6) |
|  | Female | 974 (737 to 1125) | 5.2 (4 to 6.1) | 989 (748 to 1131) | 5.5 (4.1 to 6.4) | 22909 (17492 to 26492) | 112.9 (86.6 to 129.6) | 213 (138 to 296) | 1.1 (0.7 to 1.6) | 23122 (17659 to 26658) | 114.1 (87.4 to 130.7) | 2203 (1608 to 2770) | 4.1 (3 to 5.1) | 2001 (1481 to 2388) | 3.7 (2.8 to 4.5) | 41474 (31173 to 49827) | 77.1 (57.8 to 92.3) | 504 (312 to 732) | 0.9 (0.6 to 1.4) | 41978 (31460 to 50428) | 78.1 (58.4 to 93.5) |
|  | Male | 1097 (599 to 1426) | 6.8 (3.7 to 9) | 1107 (604 to 1447) | 7.3 (4 to 9.7) | 27678 (15152 to 35613) | 151.9 (82.8 to 197.6) | 239 (120 to 359) | 1.4 (0.7 to 2.2) | 27917 (15278 to 35896) | 153.3 (83.5 to 199.4) | 2936 (1276 to 3755) | 6.6 (2.9 to 8.4) | 2671 (1196 to 3344) | 6.1 (2.8 to 7.6) | 60615 (27127 to 76579) | 130.4 (59.3 to 164.1) | 664 (271 to 1003) | 1.5 (0.6 to 2.2) | 61279 (27345 to 77431) | 131.9 (59.8 to 166) |
| The Bahamas | Both | 7 (5 to 8) | 4.4 (3.3 to 4.8) | 7 (5 to 7) | 4.5 (3.4 to 4.9) | 169 (126 to 184) | 100.8 (75.6 to 109.7) | 2 (1 to 2) | 1 (0.6 to 1.3) | 171 (127 to 186) | 101.8 (76.2 to 110.9) | 6 (6 to 9) | 1.8 (1.5 to 2.4) | 7 (6 to 9) | 1.8 (1.6 to 2.5) | 156 (136 to 209) | 39.4 (34.4 to 53.1) | 1 (1 to 2) | 0.4 (0.3 to 0.6) | 157 (137 to 211) | 39.8 (34.8 to 53.6) |
|  | Female | 4 (3 to 4) | 4.3 (3.6 to 4.7) | 4 (3 to 4) | 4.3 (3.7 to 4.8) | 90 (72 to 100) | 98.3 (78.1 to 108.8) | 1 (1 to 1) | 0.9 (0.6 to 1.3) | 91 (72 to 101) | 99.2 (79.1 to 109.8) | 4 (3 to 5) | 1.8 (1.5 to 2.2) | 4 (3 to 5) | 1.9 (1.6 to 2.3) | 88 (74 to 108) | 41.1 (34.8 to 50.8) | 1 (1 to 1) | 0.4 (0.3 to 0.6) | 88 (75 to 109) | 41.5 (35.2 to 51.3) |
|  | Male | 3 (2 to 4) | 4.7 (2.7 to 5.3) | 3 (2 to 4) | 4.9 (2.9 to 5.5) | 79 (44 to 90) | 104.8 (59.5 to 118.5) | 1 (0 to 1) | 1 (0.6 to 1.4) | 80 (44 to 90) | 105.8 (60 to 119.7) | 3 (2 to 5) | 1.7 (1.4 to 2.8) | 3 (2 to 5) | 1.8 (1.5 to 2.9) | 68 (57 to 106) | 37.5 (31.2 to 59.6) | 1 (0 to 1) | 0.4 (0.2 to 0.6) | 69 (57 to 108) | 37.9 (31.6 to 60.3) |
| The Gambia | Both | 5 (4 to 6) | 1.5 (1.2 to 1.9) | 5 (4 to 6) | 1.6 (1.3 to 2) | 116 (92 to 152) | 31.6 (25.3 to 41.4) | 1 (1 to 2) | 0.3 (0.2 to 0.5) | 117 (93 to 153) | 31.9 (25.5 to 41.8) | 12 (10 to 16) | 1.4 (1.1 to 1.9) | 13 (10 to 17) | 1.5 (1.2 to 2) | 279 (224 to 379) | 29.9 (24 to 40.3) | 3 (2 to 4) | 0.3 (0.2 to 0.4) | 281 (227 to 382) | 30.2 (24.3 to 40.6) |
|  | Female | 2 (2 to 3) | 1.6 (1.2 to 2.2) | 3 (2 to 4) | 1.7 (1.3 to 2.4) | 56 (42 to 80) | 33.6 (25.3 to 47.8) | 1 (0 to 1) | 0.3 (0.2 to 0.5) | 56 (42 to 81) | 33.9 (25.6 to 48.2) | 7 (5 to 9) | 1.5 (1.1 to 2.1) | 7 (5 to 10) | 1.7 (1.3 to 2.3) | 154 (113 to 214) | 32.6 (23.8 to 45.2) | 1 (1 to 2) | 0.3 (0.2 to 0.5) | 156 (114 to 216) | 32.9 (24 to 45.5) |
|  | Male | 2 (2 to 3) | 1.4 (1 to 1.9) | 2 (2 to 3) | 1.5 (1.1 to 2) | 60 (43 to 86) | 29.8 (21.8 to 42.1) | 1 (0 to 1) | 0.3 (0.2 to 0.4) | 60 (43 to 86) | 30.1 (22.1 to 42.5) | 5 (4 to 8) | 1.2 (1 to 1.8) | 5 (4 to 8) | 1.3 (1 to 2) | 124 (92 to 184) | 27 (20.3 to 40.1) | 1 (1 to 2) | 0.3 (0.2 to 0.4) | 126 (93 to 186) | 27.2 (20.5 to 40.5) |
| Timor-Leste | Both | 5 (4 to 6) | 1.9 (1.4 to 2.3) | 5 (4 to 6) | 2 (1.6 to 2.5) | 129 (97 to 161) | 41.6 (31.6 to 51.3) | 1 (1 to 2) | 0.4 (0.3 to 0.6) | 130 (97 to 162) | 42 (31.9 to 51.8) | 13 (10 to 17) | 1.7 (1.3 to 2.2) | 14 (11 to 18) | 1.9 (1.5 to 2.4) | 308 (228 to 400) | 37.4 (27.9 to 48.3) | 3 (2 to 4) | 0.4 (0.2 to 0.6) | 311 (230 to 403) | 37.8 (28.1 to 48.8) |
|  | Female | 3 (2 to 4) | 2.1 (1.4 to 2.8) | 3 (2 to 4) | 2.3 (1.5 to 3) | 72 (46 to 97) | 48 (31 to 62.4) | 1 (0 to 1) | 0.5 (0.3 to 0.7) | 72 (46 to 98) | 48.4 (31.3 to 63.1) | 7 (5 to 9) | 1.7 (1.3 to 2.2) | 7 (5 to 9) | 1.8 (1.4 to 2.3) | 155 (116 to 198) | 37.4 (27.9 to 47.6) | 2 (1 to 2) | 0.4 (0.2 to 0.5) | 157 (118 to 200) | 37.7 (28.2 to 48.1) |
|  | Male | 2 (2 to 3) | 1.6 (1.2 to 2.2) | 2 (2 to 3) | 1.7 (1.3 to 2.4) | 57 (41 to 82) | 35.5 (26.3 to 50.2) | 0 (0 to 1) | 0.4 (0.2 to 0.5) | 58 (42 to 83) | 35.9 (26.6 to 50.6) | 7 (4 to 10) | 1.8 (1.2 to 2.6) | 7 (5 to 10) | 1.9 (1.3 to 2.8) | 152 (93 to 234) | 37.5 (23.3 to 57) | 1 (1 to 2) | 0.4 (0.2 to 0.6) | 154 (94 to 236) | 37.9 (23.5 to 57.6) |
| Togo | Both | 17 (14 to 22) | 1.5 (1.2 to 1.9) | 18 (15 to 23) | 1.6 (1.4 to 2.1) | 418 (345 to 539) | 32.4 (26.8 to 41.4) | 4 (3 to 5) | 0.3 (0.2 to 0.5) | 421 (349 to 544) | 32.7 (27 to 41.8) | 36 (28 to 49) | 1.2 (0.9 to 1.6) | 37 (29 to 50) | 1.3 (1 to 1.7) | 895 (690 to 1228) | 25.3 (19.9 to 34.2) | 8 (5 to 12) | 0.3 (0.2 to 0.4) | 903 (697 to 1239) | 25.6 (20.1 to 34.5) |
|  | Female | 10 (8 to 14) | 1.7 (1.4 to 2.3) | 10 (8 to 14) | 1.8 (1.5 to 2.5) | 241 (190 to 335) | 36.4 (29 to 50) | 2 (1 to 3) | 0.4 (0.2 to 0.6) | 243 (192 to 338) | 36.7 (29.3 to 50.6) | 21 (16 to 31) | 1.2 (0.9 to 1.8) | 22 (16 to 32) | 1.4 (1 to 1.9) | 517 (373 to 743) | 26.1 (19 to 37.5) | 5 (3 to 7) | 0.3 (0.2 to 0.4) | 522 (377 to 751) | 26.4 (19.2 to 37.9) |
|  | Male | 7 (5 to 9) | 1.3 (0.9 to 1.7) | 7 (5 to 10) | 1.4 (1 to 1.8) | 176 (126 to 237) | 28.2 (20.1 to 37.1) | 2 (1 to 2) | 0.3 (0.2 to 0.4) | 178 (127 to 239) | 28.5 (20.3 to 37.5) | 15 (10 to 23) | 1.1 (0.8 to 1.7) | 15 (10 to 23) | 1.2 (0.8 to 1.8) | 377 (245 to 592) | 24 (16.2 to 37.2) | 3 (2 to 5) | 0.2 (0.1 to 0.4) | 380 (248 to 596) | 24.3 (16.4 to 37.6) |
| Tonga | Both | 1 (0 to 1) | 1 (0.8 to 1.2) | 1 (0 to 1) | 1.1 (0.9 to 1.3) | 12 (10 to 14) | 21 (17.2 to 24.8) | 0 (0 to 0) | 0.2 (0.1 to 0.3) | 12 (10 to 14) | 21.2 (17.4 to 25.1) | 1 (1 to 1) | 0.9 (0.7 to 1.1) | 1 (1 to 1) | 1 (0.8 to 1.2) | 16 (12 to 19) | 19.5 (15.1 to 23.7) | 0 (0 to 0) | 0.2 (0.1 to 0.3) | 16 (12 to 19) | 19.7 (15.3 to 23.9) |
|  | Female | 0 (0 to 0) | 1.1 (0.8 to 1.3) | 0 (0 to 0) | 1.2 (0.9 to 1.4) | 7 (5 to 8) | 23 (17.9 to 28.6) | 0 (0 to 0) | 0.2 (0.2 to 0.3) | 7 (5 to 8) | 23.3 (18.1 to 28.9) | 0 (0 to 1) | 1 (0.7 to 1.3) | 0 (0 to 1) | 1 (0.7 to 1.3) | 9 (6 to 12) | 20.3 (14.2 to 26.5) | 0 (0 to 0) | 0.2 (0.1 to 0.3) | 9 (6 to 12) | 20.5 (14.4 to 26.7) |
|  | Male | 0 (0 to 0) | 0.9 (0.7 to 1.2) | 0 (0 to 0) | 1 (0.8 to 1.3) | 5 (4 to 7) | 18.6 (14.7 to 24.8) | 0 (0 to 0) | 0.2 (0.1 to 0.3) | 5 (4 to 7) | 18.8 (14.8 to 25.1) | 0 (0 to 0) | 0.9 (0.7 to 1.1) | 0 (0 to 0) | 0.9 (0.7 to 1.2) | 7 (5 to 9) | 18.6 (13.4 to 23.6) | 0 (0 to 0) | 0.2 (0.1 to 0.3) | 7 (5 to 9) | 18.8 (13.6 to 23.9) |
| Trinidad and Tobago | Both | 31 (25 to 33) | 3.7 (2.9 to 3.9) | 32 (26 to 34) | 3.8 (3 to 4.1) | 728 (579 to 784) | 82.2 (65.7 to 88.5) | 7 (5 to 9) | 0.8 (0.5 to 1.1) | 735 (584 to 792) | 83 (66.3 to 89.3) | 20 (16 to 30) | 1.1 (0.9 to 1.7) | 21 (17 to 31) | 1.2 (0.9 to 1.8) | 461 (365 to 694) | 25.3 (20 to 37.9) | 4 (3 to 7) | 0.3 (0.2 to 0.4) | 466 (368 to 700) | 25.5 (20.2 to 38.3) |
|  | Female | 20 (17 to 21) | 4.3 (3.8 to 4.7) | 20 (18 to 22) | 4.5 (4 to 4.9) | 460 (406 to 502) | 100.4 (88.2 to 109.6) | 4 (3 to 6) | 1 (0.6 to 1.3) | 465 (410 to 506) | 101.3 (89 to 110.7) | 12 (9 to 18) | 1.3 (1 to 1.9) | 13 (10 to 18) | 1.4 (1 to 2) | 284 (207 to 409) | 30.2 (22 to 43.7) | 3 (2 to 4) | 0.3 (0.2 to 0.4) | 286 (209 to 413) | 30.5 (22.2 to 44.1) |
|  | Male | 12 (7 to 13) | 2.9 (1.7 to 3.2) | 12 (7 to 13) | 3.1 (1.8 to 3.4) | 268 (158 to 298) | 63.2 (37 to 70.2) | 3 (1 to 3) | 0.6 (0.4 to 0.9) | 270 (159 to 301) | 63.9 (37.4 to 71) | 8 (6 to 14) | 0.9 (0.7 to 1.6) | 8 (6 to 14) | 1 (0.7 to 1.7) | 178 (130 to 306) | 20.1 (14.8 to 34.7) | 2 (1 to 3) | 0.2 (0.1 to 0.4) | 179 (131 to 309) | 20.4 (15 to 35.1) |
| Tropical Latin America | Both | 2964 (2861 to 3057) | 3.3 (3.2 to 3.5) | 3042 (2938 to 3131) | 3.6 (3.4 to 3.7) | 70987 (68732 to 73402) | 72.7 (70.3 to 75.1) | 649 (445 to 858) | 0.7 (0.5 to 1) | 71637 (69384 to 74121) | 73.4 (71.1 to 75.8) | 5084 (4908 to 5230) | 2.2 (2.1 to 2.3) | 5308 (5131 to 5466) | 2.3 (2.3 to 2.4) | 113646 (109594 to 116904) | 48 (46.3 to 49.3) | 1128 (771 to 1493) | 0.5 (0.3 to 0.6) | 114774 (110696 to 118156) | 48.5 (46.8 to 49.9) |
|  | Female | 2110 (2049 to 2179) | 4.4 (4.3 to 4.6) | 2173 (2110 to 2238) | 4.7 (4.6 to 4.8) | 50067 (48547 to 51742) | 97 (94.1 to 100.1) | 457 (317 to 608) | 0.9 (0.7 to 1.3) | 50524 (48979 to 52217) | 97.9 (95 to 101.1) | 3467 (3332 to 3581) | 2.7 (2.6 to 2.8) | 3652 (3522 to 3765) | 2.9 (2.8 to 3) | 76642 (73655 to 79107) | 59.6 (57.3 to 61.5) | 763 (520 to 1015) | 0.6 (0.4 to 0.8) | 77405 (74326 to 79884) | 60.2 (57.9 to 62.2) |
|  | Male | 854 (794 to 899) | 2.1 (1.9 to 2.2) | 870 (809 to 918) | 2.2 (2 to 2.3) | 20920 (19634 to 22225) | 45.2 (42.1 to 47.8) | 192 (130 to 256) | 0.5 (0.3 to 0.6) | 21113 (19827 to 22381) | 45.6 (42.6 to 48.4) | 1617 (1443 to 1691) | 1.6 (1.4 to 1.6) | 1656 (1459 to 1735) | 1.7 (1.5 to 1.7) | 37004 (32644 to 38851) | 34.1 (30.3 to 35.8) | 365 (249 to 489) | 0.4 (0.2 to 0.5) | 37369 (32984 to 39207) | 34.5 (30.5 to 36.2) |
| Tunisia | Both | 131 (104 to 151) | 2.7 (2.2 to 3.2) | 131 (104 to 152) | 2.9 (2.3 to 3.3) | 2802 (2254 to 3244) | 53.4 (42.8 to 61.6) | 29 (19 to 39) | 0.6 (0.4 to 0.8) | 2830 (2275 to 3280) | 54 (43.2 to 62.2) | 265 (198 to 337) | 2.3 (1.7 to 2.8) | 270 (201 to 340) | 2.4 (1.8 to 2.9) | 5267 (3892 to 6740) | 43 (31.8 to 54.8) | 59 (38 to 85) | 0.5 (0.3 to 0.7) | 5326 (3932 to 6809) | 43.5 (32.2 to 55.5) |
|  | Female | 90 (68 to 108) | 3.9 (3 to 4.7) | 90 (67 to 108) | 4.1 (3.1 to 4.9) | 1950 (1470 to 2334) | 77.2 (58.3 to 92.1) | 20 (12 to 27) | 0.8 (0.5 to 1.2) | 1969 (1483 to 2356) | 78.1 (58.8 to 93.2) | 182 (126 to 244) | 3 (2.1 to 4) | 186 (130 to 248) | 3.1 (2.2 to 4.1) | 3624 (2513 to 4943) | 57.8 (40.1 to 78.3) | 40 (24 to 62) | 0.7 (0.4 to 1) | 3664 (2538 to 4994) | 58.5 (40.5 to 79.4) |
|  | Male | 41 (29 to 50) | 1.6 (1.1 to 2) | 41 (29 to 51) | 1.7 (1.2 to 2.1) | 852 (607 to 1077) | 31.4 (22.4 to 39) | 9 (6 to 13) | 0.4 (0.2 to 0.5) | 861 (612 to 1090) | 31.8 (22.6 to 39.4) | 83 (54 to 113) | 1.5 (1 to 2) | 84 (54 to 115) | 1.5 (1 to 2.1) | 1643 (1068 to 2303) | 27.6 (18 to 38.3) | 19 (11 to 29) | 0.3 (0.2 to 0.5) | 1662 (1080 to 2328) | 27.9 (18.2 to 38.7) |
| Turkey | Both | 686 (481 to 894) | 1.9 (1.4 to 2.6) | 700 (494 to 914) | 2.1 (1.5 to 2.7) | 16706 (11650 to 21501) | 44.2 (30.8 to 57.4) | 153 (92 to 224) | 0.4 (0.3 to 0.6) | 16859 (11751 to 21697) | 44.6 (31.1 to 57.9) | 1012 (860 to 1418) | 1.2 (1 to 1.6) | 1014 (868 to 1432) | 1.2 (1 to 1.7) | 22126 (18954 to 29991) | 25 (21.4 to 34.1) | 229 (156 to 332) | 0.3 (0.2 to 0.4) | 22355 (19148 to 30260) | 25.3 (21.7 to 34.4) |
|  | Female | 408 (275 to 509) | 2.2 (1.5 to 2.8) | 421 (286 to 529) | 2.3 (1.6 to 2.9) | 9794 (6648 to 12074) | 50 (33.9 to 61.6) | 91 (54 to 131) | 0.5 (0.3 to 0.7) | 9885 (6710 to 12168) | 50.5 (34.2 to 62.3) | 530 (441 to 705) | 1.1 (0.9 to 1.5) | 535 (448 to 727) | 1.1 (0.9 to 1.5) | 11653 (9664 to 14955) | 24.9 (20.7 to 32) | 119 (80 to 167) | 0.2 (0.2 to 0.4) | 11772 (9762 to 15106) | 25.2 (20.9 to 32.3) |
|  | Male | 278 (166 to 446) | 1.7 (1 to 2.7) | 279 (170 to 450) | 1.7 (1.1 to 2.8) | 6912 (4056 to 11050) | 37.9 (22.2 to 61.4) | 62 (33 to 105) | 0.4 (0.2 to 0.6) | 6974 (4095 to 11155) | 38.3 (22.4 to 62.1) | 482 (345 to 771) | 1.2 (0.9 to 2) | 479 (342 to 768) | 1.3 (0.9 to 2.1) | 10473 (7557 to 16039) | 25.6 (18.3 to 39.8) | 110 (69 to 169) | 0.3 (0.2 to 0.4) | 10583 (7640 to 16182) | 25.9 (18.5 to 40.2) |
| Turkmenistan | Both | 48 (28 to 53) | 2.4 (1.5 to 2.7) | 48 (29 to 54) | 2.6 (1.6 to 2.9) | 1223 (696 to 1366) | 58.8 (33.9 to 65.8) | 11 (6 to 15) | 0.5 (0.3 to 0.7) | 1234 (702 to 1380) | 59.3 (34.2 to 66.3) | 39 (35 to 43) | 1 (0.9 to 1.1) | 39 (36 to 44) | 1.1 (1 to 1.2) | 1020 (921 to 1128) | 24.6 (22.3 to 27.2) | 9 (6 to 12) | 0.2 (0.2 to 0.3) | 1029 (929 to 1138) | 24.8 (22.5 to 27.4) |
|  | Female | 31 (20 to 36) | 2.8 (1.8 to 3.2) | 32 (20 to 37) | 2.9 (1.9 to 3.3) | 779 (463 to 883) | 66.6 (40.2 to 75.6) | 7 (4 to 10) | 0.6 (0.4 to 0.8) | 786 (468 to 892) | 67.2 (40.6 to 76.3) | 24 (20 to 27) | 1.1 (1 to 1.3) | 24 (21 to 28) | 1.2 (1 to 1.4) | 603 (520 to 694) | 26.9 (23.2 to 30.6) | 5 (4 to 7) | 0.3 (0.2 to 0.3) | 609 (525 to 700) | 27.1 (23.5 to 30.9) |
|  | Male | 16 (8 to 19) | 2 (1.1 to 2.3) | 16 (8 to 19) | 2.1 (1.1 to 2.4) | 444 (214 to 510) | 48.6 (24.5 to 55.8) | 4 (2 to 5) | 0.4 (0.2 to 0.6) | 448 (216 to 514) | 49 (24.7 to 56.4) | 15 (13 to 18) | 0.9 (0.8 to 1.1) | 15 (13 to 17) | 0.9 (0.8 to 1.1) | 417 (360 to 480) | 21.7 (19 to 24.9) | 3 (2 to 5) | 0.2 (0.1 to 0.3) | 420 (362 to 484) | 21.9 (19.2 to 25.1) |
| Uganda | Both | 48 (34 to 58) | 0.8 (0.6 to 1) | 51 (36 to 62) | 0.9 (0.6 to 1.1) | 1162 (819 to 1404) | 17.3 (12 to 20.8) | 11 (6 to 15) | 0.2 (0.1 to 0.2) | 1172 (825 to 1415) | 17.5 (12.2 to 21) | 72 (59 to 90) | 0.6 (0.5 to 0.7) | 76 (62 to 95) | 0.7 (0.5 to 0.8) | 1790 (1446 to 2241) | 12.8 (10.4 to 16) | 16 (10 to 23) | 0.1 (0.1 to 0.2) | 1806 (1458 to 2260) | 13 (10.5 to 16.2) |
|  | Female | 27 (18 to 34) | 0.9 (0.6 to 1.1) | 29 (19 to 35) | 1 (0.7 to 1.3) | 660 (452 to 817) | 19.9 (13.4 to 24.6) | 6 (4 to 9) | 0.2 (0.1 to 0.3) | 666 (456 to 823) | 20.1 (13.6 to 24.8) | 39 (30 to 52) | 0.6 (0.4 to 0.8) | 41 (31 to 55) | 0.6 (0.5 to 0.8) | 927 (694 to 1268) | 12.1 (9.2 to 16.4) | 9 (5 to 12) | 0.1 (0.1 to 0.2) | 936 (701 to 1280) | 12.2 (9.3 to 16.5) |
|  | Male | 21 (13 to 28) | 0.7 (0.4 to 0.9) | 23 (13 to 30) | 0.8 (0.5 to 1) | 501 (297 to 681) | 14.8 (8.7 to 19.9) | 5 (3 to 7) | 0.2 (0.1 to 0.2) | 506 (300 to 687) | 15 (8.8 to 20.1) | 33 (24 to 46) | 0.6 (0.4 to 0.9) | 35 (25 to 49) | 0.7 (0.5 to 1) | 863 (610 to 1191) | 13.7 (9.8 to 19.3) | 7 (5 to 11) | 0.1 (0.1 to 0.2) | 870 (617 to 1202) | 13.9 (9.9 to 19.4) |
| Ukraine | Both | 1047 (869 to 1391) | 1.4 (1.2 to 1.9) | 995 (843 to 1317) | 1.3 (1.1 to 1.8) | 21997 (18490 to 28562) | 29.6 (25 to 38.6) | 240 (155 to 352) | 0.3 (0.2 to 0.5) | 22237 (18699 to 28876) | 29.9 (25.2 to 38.9) | 1214 (1073 to 1418) | 1.6 (1.4 to 1.8) | 1019 (939 to 1170) | 1.3 (1.2 to 1.5) | 22373 (20646 to 25585) | 30.4 (27.9 to 34.6) | 294 (197 to 400) | 0.4 (0.3 to 0.5) | 22668 (20920 to 25933) | 30.8 (28.4 to 35) |
|  | Female | 674 (570 to 999) | 1.4 (1.2 to 2.1) | 639 (554 to 935) | 1.3 (1.1 to 1.9) | 13351 (11607 to 19521) | 28.7 (25.1 to 41.7) | 154 (101 to 242) | 0.3 (0.2 to 0.5) | 13505 (11757 to 19718) | 29.1 (25.4 to 42.2) | 751 (636 to 909) | 1.6 (1.4 to 1.9) | 617 (559 to 707) | 1.2 (1.1 to 1.4) | 12736 (11529 to 14365) | 28.4 (25.4 to 31.7) | 182 (121 to 255) | 0.4 (0.3 to 0.6) | 12918 (11690 to 14591) | 28.8 (25.8 to 32.1) |
|  | Male | 373 (265 to 534) | 1.4 (1 to 2) | 356 (256 to 512) | 1.4 (1 to 2) | 8645 (6118 to 12563) | 30.8 (22.1 to 44.4) | 86 (48 to 137) | 0.3 (0.2 to 0.5) | 8731 (6171 to 12689) | 31.2 (22.3 to 44.8) | 463 (399 to 584) | 1.6 (1.4 to 2) | 402 (360 to 503) | 1.4 (1.3 to 1.8) | 9637 (8581 to 12023) | 33 (29.3 to 41.6) | 112 (73 to 157) | 0.4 (0.3 to 0.5) | 9749 (8670 to 12141) | 33.4 (29.6 to 42) |
| United Arab Emirates | Both | 7 (5 to 11) | 1.7 (1 to 2.6) | 7 (4 to 10) | 1.8 (1.1 to 2.8) | 215 (143 to 314) | 36.3 (22.6 to 57.1) | 2 (1 to 3) | 0.4 (0.2 to 0.6) | 217 (144 to 317) | 36.7 (22.9 to 57.7) | 51 (35 to 76) | 1.3 (0.8 to 2.1) | 43 (29 to 64) | 1.4 (0.8 to 2.1) | 1510 (1052 to 2158) | 28 (17.7 to 44.4) | 12 (7 to 19) | 0.3 (0.2 to 0.5) | 1523 (1061 to 2182) | 28.3 (17.9 to 44.9) |
|  | Female | 3 (2 to 5) | 2.3 (1.1 to 3.7) | 3 (2 to 5) | 2.4 (1.2 to 3.9) | 91 (45 to 139) | 51.4 (25.1 to 82.8) | 1 (0 to 1) | 0.5 (0.2 to 0.9) | 92 (45 to 140) | 51.9 (25.4 to 83.7) | 13 (7 to 22) | 1.5 (0.9 to 2.7) | 12 (6 to 20) | 1.6 (0.9 to 2.8) | 365 (193 to 625) | 32.7 (17.5 to 59.4) | 3 (1 to 5) | 0.3 (0.2 to 0.7) | 368 (194 to 631) | 33 (17.7 to 60.1) |
|  | Male | 4 (3 to 6) | 1.2 (0.7 to 2.2) | 4 (2 to 6) | 1.3 (0.8 to 2.2) | 124 (78 to 191) | 27.5 (16.1 to 46.6) | 1 (1 to 2) | 0.3 (0.1 to 0.5) | 125 (79 to 192) | 27.8 (16.3 to 47.2) | 39 (24 to 61) | 1.2 (0.7 to 2.1) | 32 (20 to 49) | 1.2 (0.7 to 2.1) | 1145 (736 to 1720) | 25.7 (14.8 to 44.6) | 9 (5 to 15) | 0.3 (0.1 to 0.5) | 1155 (742 to 1734) | 25.9 (14.9 to 45.1) |
| United Kingdom | Both | 2715 (2508 to 2938) | 2.9 (2.7 to 3.1) | 2086 (1933 to 2125) | 2.2 (2 to 2.2) | 36150 (33403 to 36901) | 39.9 (36.7 to 40.7) | 651 (448 to 862) | 0.7 (0.5 to 0.9) | 36802 (33914 to 37601) | 40.6 (37.3 to 41.5) | 2833 (2610 to 3117) | 2.2 (2 to 2.4) | 2048 (1981 to 2165) | 1.5 (1.5 to 1.6) | 31921 (31044 to 34201) | 26.9 (26.2 to 28.9) | 670 (470 to 877) | 0.5 (0.4 to 0.7) | 32591 (31701 to 34953) | 27.5 (26.7 to 29.5) |
|  | Female | 1415 (1308 to 1548) | 2.5 (2.3 to 2.7) | 1201 (1176 to 1224) | 2 (2 to 2.1) | 19479 (19028 to 19830) | 37.8 (36.9 to 38.5) | 322 (221 to 424) | 0.6 (0.4 to 0.8) | 19801 (19321 to 20196) | 38.3 (37.4 to 39.1) | 1572 (1405 to 1776) | 2.1 (1.9 to 2.4) | 1167 (1133 to 1206) | 1.5 (1.5 to 1.6) | 17337 (16823 to 17983) | 27.4 (26.5 to 28.4) | 360 (251 to 477) | 0.5 (0.4 to 0.7) | 17697 (17147 to 18368) | 27.9 (27 to 28.9) |
|  | Male | 1299 (1123 to 1471) | 3.4 (2.9 to 3.8) | 885 (755 to 913) | 2.3 (2 to 2.4) | 16672 (14210 to 17218) | 42.8 (36.6 to 44.2) | 329 (226 to 450) | 0.8 (0.6 to 1.1) | 17001 (14455 to 17592) | 43.6 (37.2 to 45.1) | 1261 (1130 to 1451) | 2.2 (2 to 2.5) | 881 (836 to 980) | 1.5 (1.4 to 1.7) | 14584 (13913 to 16613) | 26.5 (25.2 to 30.1) | 310 (211 to 416) | 0.6 (0.4 to 0.7) | 14894 (14222 to 16924) | 27 (25.8 to 30.6) |
| United States | Both | 7503 (6817 to 7957) | 2.2 (2 to 2.4) | 4253 (3902 to 4361) | 1.3 (1.2 to 1.3) | 76868 (70472 to 78863) | 24 (22 to 24.6) | 2019 (1427 to 2645) | 0.6 (0.4 to 0.8) | 78887 (72392 to 80786) | 24.6 (22.7 to 25.2) | 11123 (10381 to 12255) | 2 (1.9 to 2.2) | 4531 (4372 to 4945) | 0.8 (0.8 to 0.9) | 82256 (79322 to 89602) | 15.7 (15.2 to 17.1) | 3268 (2304 to 4289) | 0.6 (0.4 to 0.8) | 85524 (82357 to 93278) | 16.3 (15.7 to 17.7) |
|  | Female | 4407 (4099 to 4764) | 2.2 (2.1 to 2.4) | 2695 (2632 to 2780) | 1.3 (1.3 to 1.4) | 46924 (45933 to 48398) | 26.1 (25.4 to 26.9) | 1140 (796 to 1501) | 0.6 (0.4 to 0.8) | 48064 (46995 to 49578) | 26.6 (26 to 27.5) | 5991 (5436 to 6596) | 2 (1.8 to 2.1) | 2682 (2566 to 2824) | 0.9 (0.8 to 0.9) | 47688 (45513 to 50170) | 17.1 (16.3 to 18) | 1691 (1193 to 2260) | 0.6 (0.4 to 0.7) | 49379 (47047 to 51881) | 17.6 (16.8 to 18.5) |
|  | Male | 3096 (2502 to 3393) | 2.3 (1.8 to 2.5) | 1559 (1268 to 1607) | 1.1 (0.9 to 1.2) | 29944 (24874 to 30817) | 21.6 (17.9 to 22.2) | 880 (586 to 1161) | 0.6 (0.4 to 0.8) | 30824 (25469 to 31767) | 22.2 (18.4 to 22.9) | 5132 (4645 to 5958) | 2.1 (1.9 to 2.4) | 1849 (1753 to 2159) | 0.7 (0.7 to 0.9) | 34568 (32730 to 40110) | 14.2 (13.5 to 16.5) | 1577 (1109 to 2108) | 0.6 (0.4 to 0.8) | 36145 (34191 to 41871) | 14.8 (14 to 17.2) |
| Uruguay | Both | 387 (364 to 415) | 9.7 (9.1 to 10.4) | 401 (380 to 426) | 10 (9.4 to 10.6) | 7846 (7441 to 8333) | 200.2 (189.8 to 212.7) | 79 (56 to 105) | 2 (1.4 to 2.6) | 7925 (7514 to 8420) | 202.2 (191.5 to 214.7) | 419 (366 to 476) | 7.6 (6.6 to 8.6) | 441 (387 to 495) | 7.7 (6.8 to 8.7) | 7626 (6698 to 8591) | 149.8 (131.2 to 169.1) | 86 (58 to 116) | 1.6 (1.1 to 2.2) | 7712 (6767 to 8680) | 151.4 (132.6 to 170.8) |
|  | Female | 253 (234 to 275) | 11.1 (10.3 to 12) | 266 (248 to 284) | 11.4 (10.7 to 12.2) | 5026 (4667 to 5384) | 232.6 (216.4 to 249) | 51 (36 to 70) | 2.3 (1.6 to 3.1) | 5077 (4721 to 5441) | 234.9 (218.6 to 251.4) | 267 (221 to 317) | 8.2 (6.7 to 9.7) | 288 (239 to 336) | 8.4 (7 to 9.9) | 4760 (3939 to 5636) | 165.5 (137 to 197.3) | 54 (34 to 77) | 1.7 (1.1 to 2.4) | 4814 (3979 to 5701) | 167.2 (138.4 to 199.4) |
|  | Male | 134 (122 to 150) | 7.8 (7.1 to 8.7) | 135 (125 to 150) | 7.9 (7.3 to 8.7) | 2820 (2613 to 3115) | 160.5 (148.9 to 176.5) | 28 (19 to 39) | 1.6 (1.1 to 2.2) | 2848 (2636 to 3146) | 162.1 (150.2 to 178.3) | 152 (125 to 179) | 6.8 (5.6 to 7.9) | 153 (128 to 178) | 6.8 (5.6 to 7.9) | 2866 (2408 to 3367) | 130.5 (109.5 to 153.5) | 32 (21 to 45) | 1.5 (0.9 to 2) | 2899 (2437 to 3403) | 131.9 (110.7 to 154.9) |
| Uzbekistan | Both | 63 (50 to 99) | 0.5 (0.4 to 0.9) | 65 (51 to 105) | 0.6 (0.5 to 1) | 1506 (1232 to 2245) | 12.5 (10.1 to 19) | 14 (9 to 25) | 0.1 (0.1 to 0.2) | 1520 (1243 to 2271) | 12.6 (10.2 to 19.2) | 117 (103 to 135) | 0.6 (0.5 to 0.7) | 121 (107 to 140) | 0.7 (0.6 to 0.7) | 2946 (2581 to 3408) | 12.9 (11.3 to 14.8) | 26 (18 to 36) | 0.1 (0.1 to 0.2) | 2973 (2606 to 3446) | 13 (11.5 to 14.9) |
|  | Female | 35 (28 to 69) | 0.5 (0.4 to 1) | 36 (28 to 74) | 0.5 (0.4 to 1.1) | 831 (671 to 1533) | 12.1 (9.7 to 22.5) | 8 (5 to 17) | 0.1 (0.1 to 0.3) | 838 (676 to 1547) | 12.2 (9.8 to 22.8) | 66 (55 to 79) | 0.6 (0.5 to 0.7) | 67 (56 to 82) | 0.6 (0.5 to 0.7) | 1660 (1381 to 2010) | 12.8 (10.7 to 15.6) | 15 (10 to 21) | 0.1 (0.1 to 0.2) | 1675 (1394 to 2025) | 13 (10.8 to 15.7) |
|  | Male | 28 (20 to 40) | 0.6 (0.4 to 0.9) | 29 (21 to 42) | 0.7 (0.5 to 1) | 675 (512 to 973) | 13.4 (9.9 to 19.4) | 6 (4 to 10) | 0.1 (0.1 to 0.2) | 681 (516 to 980) | 13.5 (10 to 19.6) | 52 (44 to 62) | 0.7 (0.6 to 0.8) | 54 (46 to 65) | 0.8 (0.7 to 0.9) | 1287 (1073 to 1555) | 13.5 (11.4 to 16.1) | 12 (8 to 16) | 0.1 (0.1 to 0.2) | 1298 (1083 to 1572) | 13.6 (11.5 to 16.3) |
| Vanuatu | Both | 1 (1 to 2) | 1.8 (1.2 to 2.5) | 1 (1 to 2) | 1.9 (1.3 to 2.6) | 32 (21 to 45) | 43.1 (28.4 to 61.2) | 0 (0 to 0) | 0.4 (0.2 to 0.6) | 32 (21 to 46) | 43.5 (28.7 to 61.7) | 3 (2 to 4) | 1.9 (1.1 to 2.7) | 3 (2 to 4) | 2 (1.2 to 2.8) | 78 (45 to 114) | 43.6 (25.3 to 63.5) | 1 (0 to 1) | 0.4 (0.2 to 0.7) | 78 (45 to 115) | 44 (25.5 to 64) |
|  | Female | 1 (0 to 1) | 2.1 (1.2 to 3) | 1 (0 to 1) | 2.2 (1.3 to 3.2) | 17 (9 to 26) | 50.7 (27.8 to 74) | 0 (0 to 0) | 0.5 (0.2 to 0.8) | 17 (9 to 26) | 51.1 (28.1 to 74.7) | 2 (1 to 2) | 2.1 (1.1 to 3) | 2 (1 to 2) | 2.2 (1.1 to 3.2) | 43 (22 to 66) | 49.8 (24.8 to 73.8) | 0 (0 to 1) | 0.5 (0.2 to 0.7) | 44 (22 to 66) | 50.3 (25.1 to 74.4) |
|  | Male | 1 (0 to 1) | 1.6 (1 to 2.4) | 1 (0 to 1) | 1.7 (1.1 to 2.6) | 14 (9 to 23) | 36.7 (22.4 to 58) | 0 (0 to 0) | 0.3 (0.2 to 0.6) | 14 (9 to 23) | 37 (22.6 to 58.5) | 1 (1 to 2) | 1.6 (1 to 2.7) | 1 (1 to 2) | 1.8 (1.1 to 2.9) | 34 (19 to 57) | 37.6 (21.8 to 62.4) | 0 (0 to 1) | 0.4 (0.2 to 0.6) | 34 (20 to 58) | 38 (22.1 to 62.8) |
| Venezuela | Both | 371 (328 to 392) | 3.9 (3.4 to 4.1) | 380 (339 to 400) | 4.1 (3.7 to 4.3) | 8785 (7761 to 9276) | 85.4 (75.4 to 90) | 81 (55 to 109) | 0.8 (0.6 to 1.1) | 8866 (7835 to 9354) | 86.2 (76.1 to 90.9) | 443 (375 to 549) | 1.6 (1.4 to 2) | 454 (385 to 560) | 1.7 (1.4 to 2.1) | 10049 (8485 to 12330) | 34.9 (29.5 to 42.8) | 99 (66 to 141) | 0.4 (0.2 to 0.5) | 10149 (8567 to 12464) | 35.2 (29.7 to 43.3) |
|  | Female | 262 (239 to 280) | 5.1 (4.7 to 5.5) | 268 (247 to 286) | 5.4 (4.9 to 5.7) | 6196 (5667 to 6625) | 114.9 (104.9 to 122.7) | 57 (38 to 76) | 1.1 (0.7 to 1.5) | 6253 (5729 to 6690) | 116 (106 to 124) | 291 (231 to 371) | 2 (1.6 to 2.5) | 302 (241 to 380) | 2.1 (1.7 to 2.6) | 6533 (5139 to 8309) | 42.9 (33.8 to 54.4) | 65 (42 to 94) | 0.4 (0.3 to 0.6) | 6597 (5196 to 8391) | 43.3 (34.2 to 55.1) |
|  | Male | 109 (76 to 119) | 2.5 (1.7 to 2.7) | 112 (79 to 121) | 2.6 (1.9 to 2.9) | 2589 (1787 to 2821) | 53.1 (36.7 to 57.8) | 24 (15 to 33) | 0.5 (0.3 to 0.7) | 2613 (1803 to 2848) | 53.6 (37.2 to 58.4) | 152 (120 to 199) | 1.2 (0.9 to 1.6) | 153 (122 to 200) | 1.3 (1 to 1.7) | 3517 (2789 to 4486) | 25.8 (20.5 to 33) | 35 (22 to 50) | 0.3 (0.2 to 0.4) | 3551 (2813 to 4537) | 26.1 (20.8 to 33.3) |
| Vietnam | Both | 494 (419 to 611) | 1.2 (1.1 to 1.5) | 520 (441 to 646) | 1.3 (1.1 to 1.7) | 11345 (9546 to 14063) | 27.1 (22.9 to 33.7) | 110 (73 to 152) | 0.3 (0.2 to 0.4) | 11455 (9617 to 14200) | 27.4 (23.1 to 34) | 1082 (807 to 1286) | 1.2 (0.9 to 1.5) | 1058 (794 to 1250) | 1.2 (0.9 to 1.5) | 23661 (17408 to 28464) | 25.4 (18.7 to 30.3) | 248 (160 to 344) | 0.3 (0.2 to 0.4) | 23909 (17636 to 28694) | 25.7 (19 to 30.6) |
|  | Female | 282 (224 to 347) | 1.2 (1 to 1.5) | 300 (238 to 368) | 1.3 (1 to 1.6) | 6247 (4984 to 7738) | 26.1 (20.7 to 32.2) | 63 (41 to 90) | 0.3 (0.2 to 0.4) | 6310 (5032 to 7827) | 26.3 (21 to 32.6) | 519 (378 to 675) | 1 (0.8 to 1.3) | 514 (383 to 657) | 1 (0.8 to 1.3) | 10593 (7697 to 13782) | 20.5 (14.9 to 26.5) | 118 (75 to 173) | 0.2 (0.1 to 0.3) | 10711 (7781 to 13952) | 20.7 (15 to 26.8) |
|  | Male | 212 (168 to 298) | 1.3 (1 to 1.8) | 220 (176 to 307) | 1.4 (1.1 to 2) | 5098 (4014 to 7091) | 28.6 (22.6 to 40) | 47 (30 to 70) | 0.3 (0.2 to 0.4) | 5145 (4064 to 7156) | 28.9 (22.8 to 40.4) | 563 (348 to 722) | 1.5 (1 to 2) | 544 (343 to 692) | 1.5 (1 to 2) | 13068 (7951 to 16980) | 31.7 (19.8 to 40.7) | 130 (77 to 188) | 0.3 (0.2 to 0.5) | 13198 (8031 to 17111) | 32.1 (20 to 41.3) |
| Virgin Islands, U.S. | Both | 2 (2 to 3) | 3 (2.4 to 3.3) | 3 (2 to 3) | 3.1 (2.5 to 3.4) | 58 (47 to 65) | 63.8 (51.7 to 70.6) | 1 (0 to 1) | 0.7 (0.4 to 0.9) | 59 (47 to 65) | 64.5 (52.1 to 71.4) | 3 (2 to 4) | 1.6 (1.3 to 2.1) | 3 (3 to 4) | 1.6 (1.4 to 2.2) | 64 (52 to 87) | 34 (28.1 to 46.2) | 1 (0 to 1) | 0.4 (0.2 to 0.5) | 64 (53 to 88) | 34.4 (28.4 to 46.6) |
|  | Female | 1 (1 to 1) | 2.5 (2.1 to 2.8) | 1 (1 to 1) | 2.6 (2.2 to 2.9) | 26 (22 to 29) | 52.5 (44.3 to 60.3) | 0 (0 to 0) | 0.5 (0.4 to 0.8) | 26 (22 to 30) | 53.1 (44.8 to 60.7) | 1 (1 to 1) | 1.1 (1 to 1.4) | 1 (1 to 1) | 1.2 (1 to 1.4) | 24 (21 to 29) | 24.3 (20.6 to 28.7) | 0 (0 to 0) | 0.3 (0.2 to 0.4) | 25 (21 to 29) | 24.5 (20.9 to 29) |
|  | Male | 1 (1 to 2) | 3.6 (2.6 to 4.2) | 1 (1 to 2) | 3.8 (2.8 to 4.3) | 32 (24 to 38) | 77.3 (56.5 to 88.9) | 0 (0 to 0) | 0.8 (0.5 to 1.1) | 33 (24 to 38) | 78.1 (57.1 to 89.8) | 2 (1 to 3) | 2.2 (1.6 to 3.3) | 2 (1 to 3) | 2.3 (1.7 to 3.4) | 39 (29 to 60) | 45.9 (34.4 to 69) | 0 (0 to 1) | 0.5 (0.3 to 0.7) | 40 (29 to 61) | 46.4 (34.8 to 69.6) |
| Western Europe | Both | 29679 (26937 to 31454) | 4.9 (4.4 to 5.2) | 21474 (19381 to 21945) | 3.5 (3.2 to 3.6) | 375313 (336982 to 383452) | 64.1 (57.4 to 65.5) | 7067 (4976 to 9157) | 1.2 (0.8 to 1.5) | 382380 (344097 to 391444) | 65.3 (58.6 to 66.8) | 28274 (25549 to 31515) | 3 (2.7 to 3.3) | 19550 (18513 to 20707) | 2 (1.9 to 2.1) | 299057 (283592 to 316873) | 35 (33.2 to 37.2) | 6915 (4828 to 9146) | 0.8 (0.5 to 1) | 305973 (289758 to 324775) | 35.8 (34 to 38.1) |
|  | Female | 18927 (17640 to 20433) | 5.1 (4.8 to 5.5) | 13980 (13622 to 14332) | 3.7 (3.6 to 3.8) | 230976 (224088 to 236800) | 68.3 (66.3 to 70) | 4389 (3068 to 5668) | 1.2 (0.9 to 1.6) | 235365 (228246 to 241258) | 69.5 (67.5 to 71.2) | 15118 (13202 to 17625) | 2.7 (2.4 to 3.1) | 10855 (10206 to 11560) | 1.9 (1.8 to 2) | 154990 (145267 to 166109) | 33.1 (31 to 35.5) | 3585 (2476 to 4859) | 0.7 (0.5 to 0.9) | 158575 (148490 to 169913) | 33.7 (31.6 to 36.3) |
|  | Male | 10752 (8346 to 11773) | 4.5 (3.4 to 4.9) | 7494 (5811 to 7723) | 3.2 (2.4 to 3.2) | 144337 (112819 to 148607) | 58.6 (45.7 to 60.3) | 2678 (1827 to 3540) | 1.1 (0.7 to 1.4) | 147016 (114472 to 151576) | 59.7 (46.4 to 61.5) | 13157 (10942 to 15262) | 3.2 (2.7 to 3.8) | 8694 (7096 to 9387) | 2.1 (1.7 to 2.3) | 144067 (121049 to 155873) | 37.3 (31.7 to 40.4) | 3331 (2196 to 4536) | 0.8 (0.6 to 1.1) | 147397 (123503 to 159437) | 38.1 (32.3 to 41.4) |
| Western Sub-Saharan Africa | Both | 1159 (974 to 1499) | 1.4 (1.2 to 1.8) | 1225 (1031 to 1576) | 1.5 (1.3 to 1.9) | 26850 (22305 to 35146) | 29.6 (24.8 to 38.5) | 257 (168 to 363) | 0.3 (0.2 to 0.4) | 27106 (22534 to 35466) | 29.9 (25 to 38.9) | 1992 (1634 to 2688) | 1.2 (1 to 1.7) | 2090 (1715 to 2810) | 1.3 (1.1 to 1.8) | 47203 (38587 to 64229) | 25.9 (21.2 to 35) | 442 (293 to 626) | 0.3 (0.2 to 0.4) | 47645 (38937 to 64830) | 26.2 (21.5 to 35.3) |
|  | Female | 663 (555 to 884) | 1.6 (1.4 to 2.2) | 708 (590 to 939) | 1.8 (1.5 to 2.4) | 15065 (12600 to 20590) | 34.5 (28.8 to 46.3) | 146 (94 to 208) | 0.4 (0.2 to 0.5) | 15211 (12726 to 20769) | 34.8 (29.1 to 46.8) | 1198 (956 to 1672) | 1.4 (1.2 to 2) | 1263 (1009 to 1754) | 1.6 (1.3 to 2.2) | 28250 (22402 to 39880) | 30.3 (24.1 to 42.2) | 265 (170 to 390) | 0.3 (0.2 to 0.5) | 28516 (22625 to 40146) | 30.6 (24.3 to 42.5) |
|  | Male | 496 (377 to 677) | 1.2 (0.9 to 1.6) | 517 (394 to 705) | 1.3 (1 to 1.7) | 11785 (8962 to 16331) | 25 (19 to 34.1) | 110 (70 to 164) | 0.3 (0.2 to 0.4) | 11895 (9039 to 16497) | 25.2 (19.2 to 34.4) | 794 (641 to 1200) | 1 (0.8 to 1.5) | 827 (667 to 1255) | 1.1 (0.9 to 1.6) | 18952 (15218 to 28768) | 21.3 (17.2 to 32.2) | 177 (112 to 271) | 0.2 (0.1 to 0.3) | 19129 (15355 to 29008) | 21.6 (17.4 to 32.5) |
| Yemen | Both | 78 (48 to 118) | 1.7 (1.1 to 2.4) | 80 (50 to 118) | 1.8 (1.2 to 2.6) | 1977 (1156 to 3058) | 36.9 (22.6 to 55.7) | 17 (10 to 28) | 0.4 (0.2 to 0.6) | 1994 (1167 to 3085) | 37.3 (22.8 to 56.1) | 168 (128 to 230) | 1.4 (1.1 to 1.9) | 173 (132 to 237) | 1.5 (1.2 to 2.1) | 4176 (3088 to 5838) | 30.8 (23.1 to 42.2) | 38 (24 to 56) | 0.3 (0.2 to 0.5) | 4213 (3120 to 5889) | 31.1 (23.4 to 42.7) |
|  | Female | 47 (29 to 69) | 2 (1.2 to 2.9) | 49 (31 to 71) | 2.1 (1.3 to 3.1) | 1152 (689 to 1701) | 43.3 (26.4 to 63.8) | 10 (6 to 17) | 0.4 (0.2 to 0.7) | 1162 (696 to 1718) | 43.7 (26.7 to 64.6) | 92 (66 to 136) | 1.5 (1.1 to 2.2) | 95 (68 to 140) | 1.6 (1.2 to 2.4) | 2282 (1612 to 3392) | 33.3 (23.6 to 49.5) | 20 (12 to 32) | 0.3 (0.2 to 0.5) | 2302 (1627 to 3419) | 33.6 (23.9 to 50) |
|  | Male | 31 (16 to 53) | 1.3 (0.7 to 2.2) | 31 (16 to 53) | 1.4 (0.8 to 2.3) | 825 (391 to 1451) | 30 (15.6 to 51) | 7 (3 to 13) | 0.3 (0.1 to 0.5) | 832 (394 to 1461) | 30.3 (15.8 to 51.4) | 76 (51 to 111) | 1.3 (0.9 to 1.8) | 78 (53 to 113) | 1.4 (1 to 2) | 1894 (1212 to 2878) | 28.3 (18.6 to 41.3) | 17 (10 to 27) | 0.3 (0.2 to 0.4) | 1911 (1224 to 2905) | 28.5 (18.8 to 41.7) |
| Zambia | Both | 38 (31 to 53) | 1.4 (1.2 to 1.9) | 40 (32 to 54) | 1.6 (1.3 to 2.1) | 972 (762 to 1338) | 31.8 (25.7 to 43.7) | 8 (5 to 12) | 0.3 (0.2 to 0.5) | 980 (767 to 1349) | 32.1 (25.9 to 44.1) | 65 (51 to 88) | 1.1 (0.9 to 1.6) | 67 (54 to 92) | 1.2 (1 to 1.7) | 1622 (1275 to 2194) | 24.5 (19.3 to 33.4) | 14 (9 to 22) | 0.2 (0.2 to 0.4) | 1636 (1286 to 2214) | 24.8 (19.5 to 33.7) |
|  | Female | 23 (17 to 35) | 1.8 (1.4 to 2.8) | 23 (17 to 36) | 2 (1.5 to 3) | 587 (424 to 904) | 40.2 (29.8 to 61.6) | 5 (3 to 8) | 0.4 (0.2 to 0.6) | 592 (428 to 913) | 40.6 (30.1 to 62.1) | 41 (29 to 64) | 1.4 (1 to 2.1) | 43 (31 to 68) | 1.5 (1.1 to 2.3) | 1021 (721 to 1576) | 29.9 (21 to 46.3) | 9 (6 to 15) | 0.3 (0.2 to 0.5) | 1031 (728 to 1590) | 30.2 (21.3 to 46.8) |
|  | Male | 15 (11 to 21) | 1.1 (0.8 to 1.5) | 16 (12 to 22) | 1.2 (0.9 to 1.6) | 385 (282 to 530) | 24.3 (17.9 to 32.9) | 3 (2 to 5) | 0.2 (0.1 to 0.4) | 389 (284 to 535) | 24.5 (18 to 33.3) | 23 (17 to 31) | 0.8 (0.6 to 1.1) | 24 (18 to 32) | 0.9 (0.7 to 1.2) | 601 (452 to 803) | 18.7 (14.1 to 25) | 5 (3 to 8) | 0.2 (0.1 to 0.3) | 606 (458 to 811) | 18.9 (14.2 to 25.3) |
| Zimbabwe | Both | 54 (45 to 66) | 1.4 (1.2 to 1.7) | 56 (47 to 69) | 1.5 (1.3 to 1.9) | 1322 (1090 to 1611) | 30.3 (25.1 to 36.8) | 12 (8 to 17) | 0.3 (0.2 to 0.4) | 1334 (1098 to 1623) | 30.6 (25.3 to 37.3) | 81 (65 to 110) | 1.3 (1 to 1.8) | 84 (68 to 115) | 1.4 (1.1 to 2) | 2089 (1664 to 2746) | 29.2 (23.4 to 39.4) | 18 (12 to 27) | 0.3 (0.2 to 0.4) | 2107 (1677 to 2772) | 29.5 (23.6 to 39.7) |
|  | Female | 28 (23 to 38) | 1.4 (1.2 to 1.9) | 30 (24 to 41) | 1.5 (1.3 to 2.1) | 679 (550 to 907) | 30.6 (24.8 to 41.2) | 6 (4 to 9) | 0.3 (0.2 to 0.5) | 685 (555 to 917) | 30.9 (25.1 to 41.5) | 50 (36 to 69) | 1.4 (1 to 2) | 52 (38 to 74) | 1.6 (1.1 to 2.3) | 1250 (897 to 1668) | 31.1 (22.4 to 42.7) | 11 (7 to 17) | 0.3 (0.2 to 0.5) | 1262 (905 to 1684) | 31.4 (22.6 to 43.2) |
|  | Male | 26 (18 to 33) | 1.4 (1 to 1.7) | 27 (18 to 34) | 1.5 (1.1 to 1.9) | 643 (435 to 815) | 29.9 (20.6 to 38) | 6 (4 to 8) | 0.3 (0.2 to 0.4) | 648 (438 to 821) | 30.2 (20.8 to 38.3) | 31 (23 to 47) | 1.1 (0.8 to 1.8) | 32 (23 to 48) | 1.2 (0.9 to 1.9) | 838 (593 to 1220) | 26.3 (18.9 to 39.7) | 7 (4 to 11) | 0.3 (0.2 to 0.4) | 845 (599 to 1232) | 26.6 (19.1 to 40.1) |
