# Supplementary material for: Global, regional, and national quality of care of gallbladder and biliary tract cancer: a systematic analysis for the global burden of disease study 1990–2017
Source: Int J Equity Health. 2021 Dec 18;20:259. doi: 10.1186/s12939-021-01596-y (PMC8684179; doi:10.1186/s12939-021-01596-y)
Supplement: Supplementary file 3 — Additional file 3. Age-standardized Quality of Care Index in 21 GBD regions, 195 countries, and SDI quintiles in 1990 and 2017. [file 12939_2021_1596_MOESM3_ESM.docx]

| **Supplementary Table 2: Age-standardized Quality of Care Index in 21 GBD regions, 195 countries, and SDI quintiles in 1990 and 2017** | | | | | |
| --- | --- | --- | --- | --- | --- |
| **location_name** | **SDI in 2017** | **Regions** | **QCI 1990** | **QCI 2017** | **Percent Change** |
| Global |  |  | 25.97710541 | 33.51002 | 28.99827 |
| East Asia |  |  | 7.075874952 | 29.31496 | 314.2945 |
| China | High-middle SDI | East Asia | 5.88665361 | 28.29328 | 380.6343 |
| North Korea | Low-middle SDI | East Asia | 11.32195205 | 13.50224 | 19.25717 |
| Taiwan | High SDI | East Asia | 32.10426778 | 57.78879 | 80.00347 |
| Southeast Asia |  |  | 4.714750132 | 14.50943 | 207.7454 |
| Cambodia | Low-middle SDI | Southeast Asia | 3.060278102 | 5.108226 | 66.92032 |
| Indonesia | Middle SDI | Southeast Asia | 4.053679028 | 6.859087 | 69.20647 |
| Laos | Low-middle SDI | Southeast Asia | 2.874240954 | 4.670476 | 62.49423 |
| Malaysia | High-middle SDI | Southeast Asia | 5.961303197 | 19.15432 | 221.311 |
| Maldives | Middle SDI | Southeast Asia | 4.708594134 | 21.97555 | 366.7115 |
| Myanmar | Low-middle SDI | Southeast Asia | 2.933634212 | 5.412933 | 84.51288 |
| Philippines | Middle SDI | Southeast Asia | 5.44568354 | 8.217944 | 50.90749 |
| Sri Lanka | Middle SDI | Southeast Asia | 6.138066077 | 22.76832 | 270.9363 |
| Thailand | Middle SDI | Southeast Asia | 5.146595299 | 20.88563 | 305.8145 |
| Timor-Leste | Low-middle SDI | Southeast Asia | 3.490892218 | 5.774569 | 65.41815 |
| Vietnam | High-middle SDI | Southeast Asia | 4.835800327 | 12.7999 | 164.6905 |
| Oceania |  |  | 3.398956461 | 4.267528 | 25.55405 |
| Fiji | Middle SDI | Oceania | 5.167334387 | 6.657174 | 28.83188 |
| Kiribati | Low SDI | Oceania | 3.194840742 | 3.845463 | 20.36478 |
| Marshall Islands | Low-middle SDI | Oceania | 3.761876483 | 4.938315 | 31.27265 |
| Federated States of Micronesia | Low-middle SDI | Oceania | 3.661144239 | 5.209429 | 42.28964 |
| Papua New Guinea | Low SDI | Oceania | 2.266615404 | 3.028862 | 33.6293 |
| Samoa | Low-middle SDI | Oceania | 5.031701434 | 7.039534 | 39.90365 |
| Solomon Islands | Low SDI | Oceania | 2.829147477 | 3.620449 | 27.9696 |
| Tonga | Middle SDI | Oceania | 5.614748059 | 7.572849 | 34.87425 |
| Vanuatu | Low-middle SDI | Oceania | 2.595623728 | 2.870248 | 10.58026 |
| Central Asia |  |  | 3.686199746 | 3.684522 | -0.04553 |
| Armenia | High-middle SDI | Central Asia | 4.455082578 | 4.912112 | 10.25861 |
| Azerbaijan | High-middle SDI | Central Asia | 3.638657561 | 3.186753 | -12.4196 |
| Georgia | High-middle SDI | Central Asia | 4.616116845 | 3.238641 | -29.8406 |
| Kazakhstan | High-middle SDI | Central Asia | 3.728337298 | 3.592354 | -3.6473 |
| Kyrgyzstan | Low-middle SDI | Central Asia | 3.965166738 | 2.987951 | -24.645 |
| Mongolia | Middle SDI | Central Asia | 2.335627262 | 3.008989 | 28.83 |
| Tajikistan | Low-middle SDI | Central Asia | 3.853617797 | 3.312135 | -14.0513 |
| Turkmenistan | Middle SDI | Central Asia | 2.65131842 | 2.922846 | 10.24122 |
| Uzbekistan | Middle SDI | Central Asia | 3.750535721 | 4.049921 | 7.982463 |
| Central Europe |  |  | 9.431361949 | 18.03257 | 91.19793 |
| Albania | Middle SDI | Central Europe | 4.067721193 | 4.72422 | 16.13923 |
| Bosnia and Herzegovina | High-middle SDI | Central Europe | 2.541441073 | 4.164241 | 63.85352 |
| Bulgaria | High-middle SDI | Central Europe | 9.146267106 | 7.738925 | -15.3871 |
| Croatia | High SDI | Central Europe | 21.80258899 | 34.89416 | 60.04593 |
| Czech Republic | High SDI | Central Europe | 12.87896601 | 33.35895 | 159.0188 |
| Hungary | High-middle SDI | Central Europe | 5.108570237 | 5.108307 | -0.00515 |
| Macedonia | High-middle SDI | Central Europe | 4.412935678 | 3.709987 | -15.9293 |
| Montenegro | High-middle SDI | Central Europe | 7.388866267 | 5.524692 | -25.2295 |
| Poland | High SDI | Central Europe | 2.448698371 | 4.221175 | 72.38445 |
| Romania | Middle SDI | Central Europe | 3.708491446 | 3.874828 | 4.485293 |
| Serbia | High-middle SDI | Central Europe | 3.177550505 | 4.039154 | 27.11533 |
| Slovakia | High SDI | Central Europe | 44.88488334 | 58.87959 | 31.17911 |
| Slovenia | High SDI | Central Europe | 13.01203408 | 34.44865 | 164.7445 |
| Eastern Europe |  |  | 11.75859886 | 24.41073 | 107.599 |
| Belarus | High-middle SDI | Eastern Europe | 8.07064015 | 16.98269 | 110.4255 |
| Estonia | High SDI | Eastern Europe | 9.569886057 | 24.41935 | 155.1687 |
| Latvia | High SDI | Eastern Europe | 8.915785968 | 18.62469 | 108.8957 |
| Lithuania | High SDI | Eastern Europe | 11.6356992 | 22.56004 | 93.88643 |
| Moldova | Middle SDI | Eastern Europe | 4.674170117 | 9.323875 | 99.47659 |
| Russian Federation | High-middle SDI | Eastern Europe | 10.7008941 | 22.68769 | 112.0168 |
| Ukraine | High-middle SDI | Eastern Europe | 15.94209052 | 31.14725 | 95.37747 |
| High-income Asia Pacific |  |  | 32.4639883 | 56.4678 | 73.9398 |
| Brunei | High SDI | High‐income Asia Pacific | 10.32911504 | 21.49607 | 108.1114 |
| Japan | High SDI | High‐income Asia Pacific | 36.00816945 | 59.22915 | 64.4881 |
| South Korea | High SDI | High‐income Asia Pacific | 7.78602972 | 46.32755 | 495.0087 |
| Singapore | High SDI | High‐income Asia Pacific | 16.87402485 | 48.75269 | 188.9215 |
| Australasia |  |  | 41.00905234 | 48.81709 | 19.0398 |
| Australia | High SDI | Australasia | 40.47496677 | 47.4415 | 17.21194 |
| New Zealand | High SDI | Australasia | 43.86173192 | 55.9217 | 27.49543 |
| Western Europe |  |  | 45.8840026 | 52.28491 | 13.9502 |
| Andorra | High SDI | Western Europe | 50.54579562 | 44.28227 | -12.3918 |
| Austria | High SDI | Western Europe | 35.60685004 | 53.70585 | 50.83011 |
| Belgium | High SDI | Western Europe | 41.69375716 | 41.42075 | -0.65479 |
| Cyprus | High SDI | Western Europe | 22.52385756 | 37.1632 | 64.99481 |
| Denmark | High SDI | Western Europe | 40.78119941 | 52.90142 | 29.72012 |
| Finland | High SDI | Western Europe | 37.54299686 | 49.55488 | 31.995 |
| France | High SDI | Western Europe | 36.68353811 | 40.37652 | 10.06713 |
| Germany | High SDI | Western Europe | 37.14418395 | 62.16546 | 67.36257 |
| Greece | High SDI | Western Europe | 41.85200895 | 39.29755 | -6.10355 |
| Iceland | High SDI | Western Europe | 54.70757119 | 48.68795 | -11.0033 |
| Ireland | High SDI | Western Europe | 34.48293293 | 41.91057 | 21.54004 |
| Israel | High-middle SDI | Western Europe | 26.06834592 | 28.98706 | 11.1964 |
| Italy | High SDI | Western Europe | 29.15851005 | 45.82775 | 57.16767 |
| Luxembourg | High SDI | Western Europe | 42.84443453 | 46.55283 | 8.655482 |
| Malta | High SDI | Western Europe | 32.62236605 | 36.73131 | 12.59547 |
| Netherlands | High SDI | Western Europe | 47.73388353 | 45.53922 | -4.5977 |
| Norway | High SDI | Western Europe | 52.07323724 | 65.36266 | 25.52063 |
| Portugal | High-middle SDI | Western Europe | 21.68858624 | 32.56935 | 50.16814 |
| Spain | High SDI | Western Europe | 51.6073372 | 64.69957 | 25.36894 |
| Sweden | High SDI | Western Europe | 100 | 32.06829 | -67.9317 |
| Switzerland | High SDI | Western Europe | 53.00804304 | 43.94824 | -17.0914 |
| United Kingdom | High SDI | Western Europe | 40.3841436 | 45.24021 | 12.02468 |
| Southern Latin America |  |  | 6.493706002 | 13.68913 | 110.8062 |
| Argentina | High-middle SDI | Southern Latin America | 6.531429364 | 10.82966 | 65.80839 |
| Chile | High-middle SDI | Southern Latin America | 6.078197723 | 16.91653 | 178.3149 |
| Uruguay | High-middle SDI | Southern Latin America | 8.135071254 | 12.03269 | 47.91136 |
| High-income North America |  |  | 57.54257422 | 72.46501 | 25.93286 |
| Canada | High SDI | High‐income North America | 38.83833819 | 41.79466 | 7.611852 |
| United States | High SDI | High‐income North America | 60.77193657 | 77.30538 | 27.20572 |
| Caribbean |  |  | 7.50082345 | 7.288181 | -2.83493 |
| Antigua and Barbuda | High-middle SDI | Caribbean | 7.184394558 | 8.073481 | 12.37525 |
| The Bahamas | High-middle SDI | Caribbean | 6.412381372 | 6.81025 | 6.20469 |
| Barbados | High-middle SDI | Caribbean | 7.797777695 | 8.740527 | 12.08997 |
| Belize | Low-middle SDI | Caribbean | 4.819788112 | 4.581149 | -4.95123 |
| Cuba | Middle SDI | Caribbean | 9.765133469 | 10.55964 | 8.136187 |
| Dominica | Middle SDI | Caribbean | 5.364189916 | 6.171618 | 15.0522 |
| Dominican Republic | Low-middle SDI | Caribbean | 4.555288026 | 5.734065 | 25.87711 |
| Grenada | Middle SDI | Caribbean | 3.728876515 | 5.253846 | 40.89621 |
| Guyana | Low-middle SDI | Caribbean | 3.479376542 | 3.570286 | 2.612821 |
| Haiti | Low SDI | Caribbean | 2.207206085 | 3.17775 | 43.97162 |
| Jamaica | Middle SDI | Caribbean | 6.835145543 | 6.52747 | -4.50138 |
| Saint Lucia | Middle SDI | Caribbean | 4.838882151 | 6.400362 | 32.26943 |
| Saint Vincent and the Grenadines | Middle SDI | Caribbean | 5.12898114 | 4.63366 | -9.6573 |
| Suriname | Middle SDI | Caribbean | 4.159516516 | 4.621784 | 11.11349 |
| Trinidad and Tobago | Middle SDI | Caribbean | 5.133896232 | 6.025554 | 17.36806 |
| Andean Latin America |  |  | 2.918625179 | 5.374587 | 84.14789 |
| Bolivia | Low-middle SDI | Andean Latin America | 1.275539463 | 3.171782 | 148.662 |
| Ecuador | Middle SDI | Andean Latin America | 3.44211244 | 6.142769 | 78.45926 |
| Peru | Middle SDI | Andean Latin America | 3.386150948 | 6.074092 | 79.38042 |
| Central Latin America |  |  | 4.449675906 | 6.924855 | 55.62605 |
| Colombia | Middle SDI | Central Latin America | 4.333813304 | 7.476132 | 72.50701 |
| Costa Rica | Middle SDI | Central Latin America | 8.393911828 | 9.805763 | 16.81994 |
| El Salvador | Low-middle SDI | Central Latin America | 3.885235196 | 6.118145 | 57.47169 |
| Guatemala | Low-middle SDI | Central Latin America | 2.598785963 | 4.691259 | 80.51734 |
| Honduras | Low-middle SDI | Central Latin America | 3.605514322 | 4.808188 | 33.35651 |
| Mexico | Middle SDI | Central Latin America | 4.389615184 | 6.833577 | 55.676 |
| Nicaragua | Low-middle SDI | Central Latin America | 3.844400796 | 5.736012 | 49.20431 |
| Panama | Middle SDI | Central Latin America | 6.498400281 | 8.100047 | 24.64678 |
| Venezuela | Middle SDI | Central Latin America | 5.115080312 | 7.433098 | 45.31733 |
| Tropical Latin America |  |  | 4.971902779 | 6.578704 | 32.31764 |
| Brazil | Middle SDI | Tropical Latin America | 4.974337748 | 6.604853 | 32.77854 |
| Paraguay | Middle SDI | Tropical Latin America | 4.921539047 | 5.321812 | 8.133091 |
| North Africa and Middle East |  |  | 4.371863446 | 7.915754 | 81.06134 |
| Algeria | Middle SDI | North Africa and Middle East | 3.095772582 | 5.49762 | 77.58475 |
| Bahrain | High-middle SDI | North Africa and Middle East | 5.55780618 | 9.82524 | 76.78271 |
| Egypt | Low-middle SDI | North Africa and Middle East | 3.49486986 | 5.444404 | 55.78274 |
| Iran | High-middle SDI | North Africa and Middle East | 5.830623875 | 10.19753 | 74.89602 |
| Iraq | Low-middle SDI | North Africa and Middle East | 3.554780455 | 4.648583 | 30.76991 |
| Jordan | Middle SDI | North Africa and Middle East | 5.240102601 | 9.354156 | 78.51093 |
| Kuwait | High-middle SDI | North Africa and Middle East | 12.31839901 | 14.38516 | 16.77783 |
| Lebanon | High-middle SDI | North Africa and Middle East | 6.392521666 | 17.79151 | 178.3176 |
| Libya | High-middle SDI | North Africa and Middle East | 5.346611413 | 7.509156 | 40.44702 |
| Morocco | Low-middle SDI | North Africa and Middle East | 3.442751913 | 4.844031 | 40.7023 |
| Palestine | Low-middle SDI | North Africa and Middle East | 4.832641108 | 5.165749 | 6.892873 |
| Oman | High-middle SDI | North Africa and Middle East | 6.096749891 | 11.53909 | 89.26624 |
| Qatar | High-middle SDI | North Africa and Middle East | 7.390878726 | 15.62212 | 111.3703 |
| Saudi Arabia | High-middle SDI | North Africa and Middle East | 6.128258683 | 12.2432 | 99.7827 |
| Syria | Middle SDI | North Africa and Middle East | 4.776797821 | 7.273152 | 52.25999 |
| Tunisia | Middle SDI | North Africa and Middle East | 8.502702522 | 10.96961 | 29.01319 |
| Turkey | High-middle SDI | North Africa and Middle East | 4.529024545 | 10.41965 | 130.064 |
| United Arab Emirates | High-middle SDI | North Africa and Middle East | 5.354383442 | 8.972593 | 67.57471 |
| Yemen | Low SDI | North Africa and Middle East | 3.44203985 | 4.074838 | 18.38439 |
| South Asia |  |  | 2.731889877 | 3.132042 | 14.64745 |
| Afghanistan | Low SDI | North Africa and Middle East | 2.877105166 | 2.98554 | 3.768901 |
| Bangladesh | Low SDI | South Asia | 2.286389648 | 3.594467 | 57.2115 |
| Bhutan | Low-middle SDI | South Asia | 2.78801355 | 4.126188 | 47.99741 |
| India | Low-middle SDI | South Asia | 2.955019808 | 3.249148 | 9.953503 |
| Nepal | Low SDI | South Asia | 2.495893418 | 3.235485 | 29.63234 |
| Pakistan | Low-middle SDI | South Asia | 1.441716986 | 2.07144 | 43.67871 |
| Central Sub-Saharan Africa |  |  | 2.840578044 | 3.199958 | 12.65166 |
| Angola | Low-middle SDI | Central Sub‐Saharan Africa | 2.528135384 | 3.226999 | 27.64343 |
| Central African Republic |  | Central Sub‐Saharan Africa | 1.925798699 | 1.807248 | -6.15591 |
| Congo | Low-middle SDI | Central Sub‐Saharan Africa | 2.439343677 | 3.15257 | 29.23843 |
| Democratic Republic of the Congo | Low SDI | Central Sub‐Saharan Africa | 3.010236849 | 3.254544 | 8.115888 |
| Equatorial Guinea | Middle SDI | Central Sub‐Saharan Africa | 2.244846475 | 4.045596 | 80.21705 |
| Gabon | Middle SDI | Central Sub‐Saharan Africa | 3.265356638 | 3.894176 | 19.2573 |
| Eastern Sub-Saharan Africa |  |  | 2.370484762 | 3.038436 | 28.17783 |
| Burundi | Low SDI | Eastern Sub‐Saharan Africa | 1.969751474 | 2.703796 | 37.26586 |
| Comoros | Low SDI | Eastern Sub‐Saharan Africa | 2.342255852 | 3.101886 | 32.43157 |
| Djibouti | Low-middle SDI | Eastern Sub‐Saharan Africa | 2.744317804 | 3.044354 | 10.93299 |
| Eritrea | Low SDI | Eastern Sub‐Saharan Africa | 0.952044827 | 2.045113 | 114.8127 |
| Ethiopia | Low SDI | Eastern Sub‐Saharan Africa | 2.06666741 | 2.994207 | 44.88092 |
| Kenya | Low-middle SDI | Eastern Sub‐Saharan Africa | 3.255987217 | 3.452546 | 6.036851 |
| Madagascar | Low SDI | Eastern Sub‐Saharan Africa | 2.197846637 | 2.329237 | 5.978121 |
| Malawi | Low SDI | Eastern Sub‐Saharan Africa | 2.750826883 | 3.003927 | 9.200878 |
| Mauritius | High-middle SDI | Southeast Asia | 9.63009202 | 20.99674 | 118.0326 |
| Mozambique | Low SDI | Eastern Sub‐Saharan Africa | 2.585130416 | 2.796279 | 8.167822 |
| Rwanda | Low SDI | Eastern Sub‐Saharan Africa | 2.244381258 | 3.158149 | 40.71358 |
| Seychelles | Middle SDI | Southeast Asia | 6.488760493 | 16.87603 | 160.081 |
| Somalia | Low SDI | Eastern Sub‐Saharan Africa | 2.361198851 | 2.164512 | -8.32996 |
| Tanzania | Low SDI | Eastern Sub‐Saharan Africa | 3.149604509 | 3.472857 | 10.26326 |
| Uganda | Low SDI | Eastern Sub‐Saharan Africa | 2.975796547 | 3.13722 | 5.42453 |
| Zambia | Low-middle SDI | Eastern Sub‐Saharan Africa | 2.457266172 | 3.122939 | 27.08996 |
| Southern Sub-Saharan Africa |  |  | 4.635878047 | 4.640157 | 0.092297 |
| Botswana | Middle SDI | Southern Sub‐Saharan Africa | 3.857954549 | 5.322127 | 37.95205 |
| Lesotho | Low-middle SDI | Southern Sub‐Saharan Africa | 3.299169068 | 3.372137 | 2.211719 |
| Namibia | Middle SDI | Southern Sub‐Saharan Africa | 2.560862873 | 3.603105 | 40.69886 |
| South Africa | Middle SDI | Southern Sub‐Saharan Africa | 4.923993478 | 5.043458 | 2.426169 |
| Swaziland | Low-middle SDI | Southern Sub‐Saharan Africa | 3.634505841 | 3.449876 | -5.0799 |
| Zimbabwe | Low-middle SDI | Southern Sub‐Saharan Africa | 3.891073434 | 2.81315 | -27.7025 |
| Western Sub-Saharan Africa |  |  | 4.232307515 | 4.446112 | 5.051713 |
| Benin | Low SDI | Western Sub‐Saharan Africa | 4.029361972 | 4.383122 | 8.779544 |
| Burkina Faso | Low SDI | Western Sub‐Saharan Africa | 4.269227062 | 4.677126 | 9.554396 |
| Cameroon | Low-middle SDI | Western Sub‐Saharan Africa | 3.858662909 | 4.00533 | 3.80097 |
| Cape Verde | Low-middle SDI | Western Sub‐Saharan Africa | 3.65695916 | 5.097757 | 39.3988 |
| Chad | Low SDI | Western Sub‐Saharan Africa | 3.688288893 | 3.876329 | 5.098302 |
| Cote d'Ivoire | Low SDI | Western Sub‐Saharan Africa | 4.523861764 | 4.47124 | -1.1632 |
| The Gambia | Low SDI | Western Sub‐Saharan Africa | 3.943900306 | 3.76504 | -4.53512 |
| Ghana | Low-middle SDI | Western Sub‐Saharan Africa | 4.313866518 | 4.810745 | 11.51817 |
| Guinea | Low SDI | Western Sub‐Saharan Africa | 3.782915546 | 3.875915 | 2.458406 |
| Guinea-Bissau | Low SDI | Western Sub‐Saharan Africa | 3.666577929 | 3.907643 | 6.574667 |
| Liberia | Low SDI | Western Sub‐Saharan Africa | 4.102876557 | 3.822601 | -6.8312 |
| Mali | Low SDI | Western Sub‐Saharan Africa | 3.784615862 | 4.508977 | 19.13963 |
| Mauritania | Low-middle SDI | Western Sub‐Saharan Africa | 4.595686552 | 5.255135 | 14.3493 |
| Niger | Low SDI | Western Sub‐Saharan Africa | 3.706167458 | 3.929303 | 6.020656 |
| Nigeria | Low-middle SDI | Western Sub‐Saharan Africa | 4.475057003 | 4.60115 | 2.817687 |
| Sao Tome and Principe | Low-middle SDI | Western Sub‐Saharan Africa | 3.518240385 | 3.813636 | 8.396115 |
| Senegal | Low SDI | Western Sub‐Saharan Africa | 4.210113916 | 4.178166 | -0.75884 |
| Sierra Leone | Middle SDI | Western Sub‐Saharan Africa | 3.642584938 | 3.833621 | 5.244513 |
| Togo | Low SDI | Western Sub‐Saharan Africa | 3.829056092 | 3.940338 | 2.906239 |
| American Samoa | High-middle SDI | Oceania | 7.338373917 | 12.66138 | 72.53657 |
| Bermuda | High-middle SDI | Caribbean | 9.641581275 | 16.35539 | 69.63392 |
| Greenland | High-middle SDI | Western Europe | 7.968579843 | 11.08776 | 39.14353 |
| Guam | High-middle SDI | Oceania | 14.65284636 | 20.18169 | 37.73221 |
| Northern Mariana Islands | High-middle SDI |  | 14.40353502 | 26.03698 | 80.768 |
| Puerto Rico | High-middle SDI | Caribbean | 12.78490913 | 15.28801 | 19.57858 |
| Virgin Islands, U.S. |  |  | 7.11489205 | 9.768213 | 37.2925 |
| South Sudan | Low SDI | Eastern Sub‐Saharan Africa | 2.784609836 | 2.607971 | -6.34339 |
| Sudan | Low-middle SDI | North Africa and Middle East | 3.538834207 | 4.394452 | 24.17796 |
| High-middle SDI |  |  | 7.348489994 | 23.95164 | 225.9397 |
| High SDI |  |  | 41.18685161 | 55.49834 | 34.74771 |
| Low-middle SDI |  |  | 3.226325007 | 3.754396 | 16.36756 |
| Low SDI |  |  | 2.74349865 | 3.13911 | 14.41995 |
| Middle SDI |  |  | 4.629315306 | 12.0222 | 159.6971 |

| Table color guide |
| --- |
| Global |
| GBD regions |
| Countries |
| SDI quintiles |
