# Supplementary material for: Global, regional, and national quality of care of gallbladder and biliary tract cancer: a systematic analysis for the global burden of disease study 1990–2017
Source: Int J Equity Health. 2021 Dec 18;20:259. doi: 10.1186/s12939-021-01596-y (PMC8684179; doi:10.1186/s12939-021-01596-y)
Supplement: Supplementary file 4 — Additional file 4. Sex-specific age-standardized Quality of Care Index, and Gender Disparity Ratio in 21 GBD regions, 195 countries, and SDI quintiles in 1990 and 2017. [file 12939_2021_1596_MOESM4_ESM.docx]

| **Supplementary Table 3: Sex-specific age-standardized Quality of Care Index, and Gender Disparity Ratio in 21 GBD regions, 195 countries, and SDI quintiles in 1990 and 2017** | | | | | | |
| --- | --- | --- | --- | --- | --- | --- |
| **location_name** | **Female** | | **Male** | | **GDR** | |
|  | **1990** | **2017** | **1990** | **2017** | **1990** | **2017** |
| Global | 24.28805697 | 29.15370268 | 28.49785355 | 39.34996261 | 0.852276714 | 0.740882602 |
| East Asia | 6.76682985 | 29.4750665 | 8.393564327 | 29.81240841 | 0.806192648 | 0.988684513 |
| China | 5.427967942 | 28.46170208 | 7.342527211 | 28.718933 | 0.73925064 | 0.991043159 |
| North Korea | 13.81843079 | 15.91424626 | 8.764929305 | 12.25406376 | 1.576559297 | 1.298691321 |
| Taiwan | 34.38121495 | 59.81219875 | 30.84072602 | 56.79511572 | 1.114799143 | 1.053122227 |
| Southeast Asia | 4.683329915 | 14.58885271 | 5.749671148 | 15.54605901 | 0.814538744 | 0.93842772 |
| Cambodia | 2.474533926 | 4.589951725 | 4.758460371 | 6.696060636 | 0.520028272 | 0.685470454 |
| Indonesia | 3.566838089 | 6.563580379 | 5.572359723 | 8.194201732 | 0.640094729 | 0.801003025 |
| Laos | 2.425058157 | 4.175268344 | 4.282003902 | 6.208189845 | 0.566337213 | 0.672541989 |
| Malaysia | 6.306781298 | 20.0213822 | 6.652332028 | 19.48441254 | 0.9480557 | 1.027558935 |
| Maldives | 4.403007523 | 24.18769692 | 6.117255849 | 22.12679217 | 0.719768411 | 1.093140692 |
| Myanmar | 2.387486984 | 4.998376811 | 4.395689935 | 6.700862417 | 0.543142719 | 0.745930374 |
| Philippines | 5.592005368 | 8.350334123 | 6.323823689 | 9.073964644 | 0.884275976 | 0.920252001 |
| Sri Lanka | 6.74945059 | 24.21434293 | 6.553576932 | 22.00808365 | 1.029888053 | 1.100247678 |
| Thailand | 5.871567851 | 22.5509509 | 5.670209473 | 20.78463601 | 1.03551163 | 1.084981757 |
| Timor-Leste | 2.817741536 | 5.350550622 | 5.256669626 | 7.30796344 | 0.536031696 | 0.732153447 |
| Vietnam | 4.766519815 | 13.53784471 | 5.834957016 | 13.34035136 | 0.816890305 | 1.014804209 |
| Oceania | 3.117414908 | 3.752241372 | 4.663208782 | 5.885965263 | 0.668512832 | 0.637489554 |
| Fiji | 4.817796091 | 6.193980481 | 6.566233133 | 8.315107455 | 0.733722972 | 0.744906848 |
| Kiribati | 2.5383437 | 3.134222874 | 4.858054424 | 5.519750603 | 0.522502113 | 0.567819653 |
| Marshall Islands | 3.417194684 | 4.504069506 | 5.128979942 | 6.529653095 | 0.6662523 | 0.689786952 |
| Federated States of Micronesia | 2.974401665 | 4.553698372 | 5.325100526 | 6.920099511 | 0.558562538 | 0.658039435 |
| Papua New Guinea | 2.028235262 | 2.59503806 | 3.481815235 | 4.51577059 | 0.58252237 | 0.574661181 |
| Samoa | 4.558619209 | 6.564147736 | 6.594227814 | 8.527179453 | 0.691304477 | 0.769791204 |
| Solomon Islands | 2.399813945 | 2.990309727 | 4.454168793 | 5.389796299 | 0.5387793 | 0.554809414 |
| Tonga | 5.214676079 | 7.451102724 | 7.064650855 | 8.6074554 | 0.738136418 | 0.86565685 |
| Vanuatu | 2.466333075 | 2.539926129 | 3.787321031 | 4.275887621 | 0.651207821 | 0.594011432 |
| Central Asia | 3.271221692 | 2.985361916 | 4.653693616 | 5.309575004 | 0.70293018 | 0.562260052 |
| Armenia | 4.45878549 | 4.502976515 | 5.122954893 | 5.795205289 | 0.870354236 | 0.777017602 |
| Azerbaijan | 3.156140241 | 2.230797929 | 4.505645351 | 5.74764036 | 0.700485723 | 0.388124132 |
| Georgia | 4.095197487 | 2.557125018 | 5.345904134 | 4.52932039 | 0.766043944 | 0.564571458 |
| Kazakhstan | 3.546754029 | 3.127707226 | 4.368372222 | 4.672719512 | 0.811916624 | 0.669354798 |
| Kyrgyzstan | 3.395645852 | 2.400841821 | 5.204427791 | 4.572862235 | 0.652453255 | 0.525019495 |
| Mongolia | 1.468758029 | 2.154483258 | 4.054270524 | 4.07168301 | 0.362274303 | 0.529138259 |
| Tajikistan | 3.27220093 | 2.531416783 | 5.335648948 | 5.24090996 | 0.613271405 | 0.483010928 |
| Turkmenistan | 2.17782935 | 2.454554742 | 3.830802998 | 4.079226583 | 0.568504658 | 0.601720618 |
| Uzbekistan | 3.225951257 | 2.976017324 | 5.333652064 | 6.751051249 | 0.604829715 | 0.440822801 |
| Central Europe | 9.322944058 | 18.65220941 | 8.91823957 | 16.34384281 | 1.045379414 | 1.14123769 |
| Albania | 3.920452716 | 4.124189095 | 5.311062153 | 6.203507179 | 0.738167358 | 0.66481572 |
| Bosnia and Herzegovina | 1.974726939 | 3.441214047 | 4.105628884 | 5.839632156 | 0.480980379 | 0.589286098 |
| Bulgaria | 11.29277757 | 8.651939026 | 5.306663496 | 5.052723137 | 2.128037245 | 1.712331903 |
| Croatia | 23.62500231 | 40.09795317 | 11.75077813 | 21.20083816 | 2.010505352 | 1.89133811 |
| Czech Republic | 12.70523819 | 31.70919575 | 12.8179513 | 36.77624004 | 0.991206621 | 0.862219621 |
| Hungary | 4.90762012 | 4.781293251 | 4.523972388 | 5.917960326 | 1.084803288 | 0.807929251 |
| Macedonia | 4.123472516 | 2.946733602 | 5.309323949 | 5.734659093 | 0.776647377 | 0.513846343 |
| Montenegro | 7.577058041 | 5.062902206 | 5.973199232 | 6.187060551 | 1.268509177 | 0.818304939 |
| Poland | 1.430621653 | 3.214620601 | 5.224152032 | 6.469185391 | 0.27384763 | 0.496912734 |
| Romania | 3.630042517 | 3.587579211 | 4.13455108 | 5.059614342 | 0.877977426 | 0.709061792 |
| Serbia | 2.418116625 | 3.216866634 | 5.067143496 | 5.635733075 | 0.477214949 | 0.57079826 |
| Slovakia | 48.32877109 | 61.58052444 | 35.20502054 | 48.91049223 | 1.372780653 | 1.259045281 |
| Slovenia | 14.5640748 | 43.39062971 | 5.501379309 | 7.136744265 | 2.647349688 | 6.079891348 |
| Eastern Europe | 12.48449393 | 26.86996819 | 10.60613391 | 19.98882167 | 1.177101292 | 1.344249733 |
| Belarus | 8.492795405 | 19.4846656 | 6.954675086 | 12.77396394 | 1.221163505 | 1.525342149 |
| Estonia | 10.21137532 | 28.09537629 | 7.544809077 | 17.48136743 | 1.353430579 | 1.607161248 |
| Latvia | 9.566522907 | 20.95257129 | 7.344876983 | 15.03155476 | 1.30247558 | 1.393905796 |
| Lithuania | 12.64066562 | 26.20654778 | 7.927694526 | 14.07419326 | 1.594494538 | 1.862028416 |
| Moldova | 4.558699034 | 10.47791492 | 5.271048965 | 8.246394507 | 0.864856135 | 1.270605585 |
| Russian Federation | 11.10478154 | 24.41108024 | 9.68587856 | 18.6236071 | 1.146491924 | 1.310760054 |
| Ukraine | 18.15386507 | 35.68584924 | 13.63512578 | 25.572912 | 1.331404298 | 1.395455052 |
| High-income Asia Pacific | 33.29621466 | 53.92588219 | 31.8717744 | 58.9997252 | 1.044692845 | 0.91400226 |
| Brunei | 9.059451327 | 19.26116025 | 12.77397157 | 24.57613018 | 0.709211797 | 0.783734466 |
| Japan | 36.71908399 | 56.56447036 | 35.464599 | 61.76509436 | 1.035372879 | 0.915799951 |
| South Korea | 7.177415025 | 43.3530469 | 9.512994266 | 49.61453994 | 0.754485373 | 0.873797216 |
| Singapore | 15.75518155 | 44.92943438 | 18.89321288 | 52.66718625 | 0.833906951 | 0.85308211 |
| Australasia | 37.66815497 | 46.57231867 | 45.34082252 | 51.50210064 | 0.830777936 | 0.904279983 |
| Australia | 36.81047177 | 43.40518996 | 45.20978754 | 52.02465219 | 0.814214659 | 0.834319657 |
| New Zealand | 41.9829769 | 60.23426246 | 46.0796867 | 48.19780582 | 0.911095103 | 1.249730386 |
| Western Europe | 44.32676239 | 49.03581907 | 48.08809306 | 56.2176731 | 0.921782495 | 0.872249176 |
| Andorra | 41.86317733 | 35.64030667 | 58.59033585 | 52.1825114 | 0.714506526 | 0.682993319 |
| Austria | 34.25036577 | 51.26680731 | 38.87076978 | 56.57742592 | 0.881134229 | 0.90613538 |
| Belgium | 34.07884149 | 32.1773554 | 49.86381977 | 48.73283197 | 0.683438245 | 0.660280844 |
| Cyprus | 17.979791 | 30.06791962 | 28.4858982 | 42.63571195 | 0.631182169 | 0.70522851 |
| Denmark | 37.14408849 | 50.60459655 | 46.00165252 | 56.00703848 | 0.807451177 | 0.903539946 |
| Finland | 33.76801417 | 45.45659435 | 43.57339533 | 54.72632564 | 0.774968623 | 0.830616597 |
| France | 29.27646121 | 31.75033702 | 44.36483053 | 47.30984617 | 0.659902469 | 0.67111478 |
| Germany | 36.3056642 | 59.53993028 | 37.44535473 | 66.18464533 | 0.969563901 | 0.899603374 |
| Greece | 34.1643389 | 31.60877943 | 49.09794098 | 45.57325034 | 0.695840563 | 0.693581853 |
| Iceland | 47.57655743 | 39.21320103 | 62.34031279 | 55.65379353 | 0.763174827 | 0.704591701 |
| Ireland | 27.88981473 | 34.59899226 | 42.16122768 | 49.37068365 | 0.661503857 | 0.700800348 |
| Israel | 21.08734193 | 22.67839612 | 33.31924586 | 35.6290352 | 0.632887732 | 0.63651446 |
| Italy | 29.48755141 | 45.2415391 | 29.2681851 | 47.09974152 | 1.007495043 | 0.960547503 |
| Luxembourg | 35.74243252 | 37.18139247 | 51.83548847 | 54.74724349 | 0.689535945 | 0.679146384 |
| Malta | 26.48482822 | 28.73346378 | 40.06494667 | 43.03967622 | 0.661047385 | 0.667604088 |
| Netherlands | 40.40499806 | 37.18976804 | 56.48874873 | 53.73517457 | 0.715275147 | 0.692093556 |
| Norway | 39.6248332 | 46.62786255 | 63.47984381 | 78.10405599 | 0.624211258 | 0.596996685 |
| Portugal | 16.87778244 | 26.4121795 | 27.82509344 | 37.84187278 | 0.606566964 | 0.697961744 |
| Spain | 54.62583548 | 64.72052297 | 44.31929886 | 65.22657291 | 1.232551888 | 0.99224166 |
| Sweden | 100 | 21.94022121 | 100 | 44.00439884 | 1 | 0.498591545 |
| Switzerland | 46.56119299 | 36.35465942 | 61.98594096 | 51.74261371 | 0.751157315 | 0.702605779 |
| United Kingdom | 31.86667103 | 41.08827997 | 48.1202728 | 50.05891205 | 0.662229642 | 0.820798501 |
| Southern Latin America | 5.138455412 | 11.09302998 | 9.691342899 | 18.60814849 | 0.530210876 | 0.596138298 |
| Argentina | 5.275023166 | 8.4664055 | 9.335171344 | 14.96269662 | 0.565069774 | 0.565834202 |
| Chile | 4.686458712 | 13.77084686 | 9.881926844 | 23.57507804 | 0.474245437 | 0.584127308 |
| Uruguay | 6.723960962 | 9.316395066 | 11.01483375 | 16.64570871 | 0.610445978 | 0.559687498 |
| High-income North America | 52.34525623 | 67.57550836 | 64.50649265 | 78.53550104 | 0.811472676 | 0.860445372 |
| Canada | 34.79121483 | 34.66716953 | 44.36885424 | 49.73590217 | 0.784135976 | 0.697025047 |
| United States | 55.31990992 | 72.5673376 | 68.04828557 | 83.1942526 | 0.812950825 | 0.872263832 |
| Caribbean | 6.561173419 | 5.797905391 | 9.676465056 | 10.11851404 | 0.678054784 | 0.572999688 |
| Antigua and Barbuda | 6.419831137 | 6.50718009 | 9.100107428 | 11.22702298 | 0.705467621 | 0.579599784 |
| The Bahamas | 5.73651562 | 5.201884619 | 8.183724365 | 9.671590516 | 0.700966377 | 0.537852033 |
| Barbados | 6.698775242 | 6.966725137 | 10.15085008 | 12.28848549 | 0.659922587 | 0.566931144 |
| Belize | 3.729305273 | 3.49330567 | 7.446383668 | 7.185092876 | 0.500820994 | 0.486187963 |
| Cuba | 8.678720122 | 8.529265961 | 12.18794365 | 14.17869723 | 0.712074192 | 0.601554982 |
| Dominica | 4.571613922 | 4.73587196 | 7.237562883 | 8.797429367 | 0.631651012 | 0.538324522 |
| Dominican Republic | 3.837068219 | 4.513611897 | 6.40245302 | 8.057977553 | 0.599312202 | 0.560142029 |
| Grenada | 2.859790271 | 4.21336508 | 5.771034757 | 7.355561229 | 0.495542029 | 0.572813542 |
| Guyana | 2.849645676 | 2.624950453 | 5.186810146 | 5.736767398 | 0.549402349 | 0.457566129 |
| Haiti | 1.10661391 | 2.381441432 | 4.675503942 | 5.168325313 | 0.236683344 | 0.460776226 |
| Jamaica | 5.582208017 | 4.536192654 | 9.486603782 | 10.50842324 | 0.588430607 | 0.431672055 |
| Saint Lucia | 4.111088196 | 4.837055573 | 6.648311055 | 9.266498242 | 0.618365802 | 0.521993902 |
| Saint Vincent and the Grenadines | 4.186438204 | 3.433801364 | 7.206646323 | 6.991099702 | 0.580913509 | 0.491167557 |
| Suriname | 3.298726728 | 3.60140809 | 6.2439558 | 6.791548025 | 0.528307188 | 0.530277939 |
| Trinidad and Tobago | 4.264087367 | 4.546123195 | 7.153855022 | 8.840374239 | 0.596054485 | 0.514245559 |
| Andean Latin America | 1.914957715 | 4.029033647 | 5.510041041 | 8.573175726 | 0.347539646 | 0.469958132 |
| Bolivia | 0.292214118 | 2.246293678 | 3.811525855 | 5.427442529 | 0.076665915 | 0.413877009 |
| Ecuador | 2.409147757 | 4.698852246 | 6.080372374 | 9.494852595 | 0.396217141 | 0.49488417 |
| Peru | 2.399120985 | 4.570967813 | 5.940786149 | 9.618868809 | 0.403838974 | 0.475208458 |
| Central Latin America | 3.407303529 | 5.596334664 | 7.21330914 | 10.03437041 | 0.472363441 | 0.557716572 |
| Colombia | 3.426240701 | 6.320157448 | 6.867120445 | 10.13084986 | 0.498934121 | 0.623852641 |
| Costa Rica | 7.375746651 | 7.957189936 | 10.78653145 | 13.50979189 | 0.683792254 | 0.588994264 |
| El Salvador | 2.841626396 | 4.818383353 | 6.949851479 | 9.778923056 | 0.408875845 | 0.492731493 |
| Guatemala | 1.593411005 | 3.521289576 | 5.248452487 | 7.540768361 | 0.303596347 | 0.466966947 |
| Honduras | 2.786777315 | 4.006060063 | 6.00849276 | 7.346666553 | 0.463806386 | 0.545289491 |
| Mexico | 3.285390811 | 5.420451878 | 7.303075396 | 10.16144091 | 0.449864014 | 0.533433391 |
| Nicaragua | 2.890264915 | 4.790931285 | 6.487483956 | 8.27532345 | 0.445513998 | 0.578941876 |
| Panama | 5.485725511 | 6.624025244 | 8.85009034 | 11.21619767 | 0.619849663 | 0.590576721 |
| Venezuela | 4.121218152 | 5.992860515 | 7.562768575 | 10.29982532 | 0.544935114 | 0.581840985 |
| Tropical Latin America | 4.019157392 | 5.21144812 | 7.238228559 | 9.483211515 | 0.555268096 | 0.549544647 |
| Brazil | 4.018727035 | 5.230727283 | 7.247045203 | 9.520950477 | 0.554533182 | 0.549391292 |
| Paraguay | 4.094320771 | 4.2776217 | 6.82584428 | 7.590600717 | 0.599826278 | 0.56354192 |
| North Africa and Middle East | 3.431227644 | 5.983968087 | 6.515236618 | 11.25125834 | 0.526646666 | 0.531848786 |
| Algeria | 1.811811511 | 3.998283973 | 7.007641319 | 9.51095626 | 0.258547981 | 0.420387169 |
| Bahrain | 4.836094785 | 7.456115135 | 7.326078305 | 12.6842202 | 0.660120543 | 0.587826056 |
| Egypt | 2.792608201 | 4.286396073 | 5.297764058 | 7.566749442 | 0.527129591 | 0.566477866 |
| Iran | 5.450245161 | 8.303060742 | 7.135806297 | 13.32744042 | 0.763788272 | 0.623004904 |
| Iraq | 3.007774645 | 3.943156363 | 5.117093798 | 6.618598306 | 0.587789625 | 0.595769101 |
| Jordan | 4.520619278 | 7.638433201 | 7.004876714 | 12.43879784 | 0.645353154 | 0.614081304 |
| Kuwait | 11.33602506 | 11.86497196 | 14.05950615 | 17.61770877 | 0.806288993 | 0.673468504 |
| Lebanon | 5.395351309 | 14.82326151 | 8.694431349 | 23.01059951 | 0.620552523 | 0.644192756 |
| Libya | 4.425563282 | 5.888008085 | 7.533185424 | 11.41950164 | 0.587475687 | 0.515609899 |
| Morocco | 2.590597333 | 3.62357166 | 6.104100961 | 7.923968412 | 0.424402766 | 0.457292542 |
| Palestine | 4.560862761 | 4.305250724 | 5.992944913 | 6.950164952 | 0.761038659 | 0.619445834 |
| Oman | 5.422297804 | 9.143132142 | 7.467670146 | 13.98615296 | 0.726103014 | 0.653727452 |
| Qatar | 5.679998533 | 12.47858169 | 9.654681668 | 18.5093396 | 0.588315465 | 0.674177575 |
| Saudi Arabia | 5.013094563 | 9.478260461 | 8.243086245 | 15.41660173 | 0.608157481 | 0.614808674 |
| Syria | 4.341767011 | 5.376214983 | 6.344695817 | 10.11568438 | 0.684314447 | 0.531473184 |
| Tunisia | 7.459919953 | 9.599171273 | 10.94864843 | 14.05384758 | 0.681355329 | 0.683027991 |
| Turkey | 3.715391441 | 8.065229241 | 6.344792214 | 13.72272194 | 0.585581263 | 0.587728096 |
| United Arab Emirates | 4.513580643 | 6.502989821 | 6.820539686 | 11.18231825 | 0.661762976 | 0.581542188 |
| Yemen | 2.854592207 | 3.390780408 | 4.75439594 | 5.815142328 | 0.600411123 | 0.583094999 |
| South Asia | 1.868902928 | 2.013141554 | 4.806373817 | 5.785077353 | 0.388838446 | 0.347988701 |
| Afghanistan | 2.030376779 | 2.197070525 | 4.964980785 | 5.327328556 | 0.408939504 | 0.41241506 |
| Bangladesh | 1.366478064 | 2.62261408 | 4.446634708 | 5.947278275 | 0.307306121 | 0.440977193 |
| Bhutan | 1.757823102 | 3.014397681 | 5.117902321 | 6.450490827 | 0.343465544 | 0.467312916 |
| India | 2.163451179 | 2.114618303 | 4.872052505 | 5.83758065 | 0.444053338 | 0.362242242 |
| Nepal | 1.763446661 | 2.385981582 | 4.339434872 | 5.369969534 | 0.406377031 | 0.444319389 |
| Pakistan | 0.499400167 | 1.164801252 | 4.804940278 | 5.291007483 | 0.103934729 | 0.220147345 |
| Central Sub-Saharan Africa | 2.344975789 | 2.514346302 | 4.363091061 | 4.715223521 | 0.537457448 | 0.533240108 |
| Angola | 1.998330551 | 2.582152236 | 4.012099488 | 4.789427149 | 0.498076021 | 0.539135925 |
| Central African Republic | 1.546337288 | 1.279979911 | 2.979823191 | 3.008166033 | 0.51893592 | 0.42550175 |
| Congo | 1.886500407 | 2.403768274 | 3.691234871 | 4.86473786 | 0.51107569 | 0.494120823 |
| Democratic Republic of the Congo | 2.504689783 | 2.55453188 | 4.674588984 | 4.770684995 | 0.535809628 | 0.535464379 |
| Equatorial Guinea | 1.721224422 | 3.247675736 | 3.595018549 | 5.959106174 | 0.478780401 | 0.544993769 |
| Gabon | 2.543796785 | 3.325710103 | 4.559485339 | 5.021697047 | 0.557913141 | 0.662268168 |
| Eastern Sub-Saharan Africa | 1.702784814 | 2.400477102 | 4.212808226 | 4.638913867 | 0.40419234 | 0.517465332 |
| Burundi | 1.511996874 | 2.155464634 | 3.306610769 | 4.070645399 | 0.457264849 | 0.529514223 |
| Comoros | 1.833989067 | 2.501897426 | 3.819524786 | 4.661912187 | 0.480161583 | 0.536667643 |
| Djibouti | 2.08215878 | 2.404313843 | 4.216447758 | 4.58611482 | 0.493818233 | 0.524259409 |
| Eritrea | 1.058135667 | 1.957642687 | 1.328623532 | 2.167966426 | 0.796414967 | 0.902985703 |
| Ethiopia | 1.186909019 | 2.261877212 | 4.206476379 | 5.04122265 | 0.282162293 | 0.448676317 |
| Kenya | 2.487856876 | 2.673183118 | 5.50203666 | 5.122089038 | 0.452170174 | 0.521893137 |
| Madagascar | 1.880725589 | 1.939613211 | 3.690349943 | 3.560076294 | 0.5096334 | 0.544823496 |
| Malawi | 1.907990118 | 2.24454434 | 4.987368948 | 4.783240525 | 0.382564462 | 0.469251824 |
| Mauritius | 10.9598043 | 21.83885506 | 8.969444524 | 20.23197949 | 1.221904464 | 1.079422559 |
| Mozambique | 2.027527777 | 2.351708922 | 4.197865179 | 4.096495874 | 0.482990208 | 0.574078186 |
| Rwanda | 1.668989457 | 2.634567906 | 3.896190142 | 4.49276978 | 0.428364478 | 0.586401715 |
| Seychelles | 7.185998364 | 17.48312331 | 6.860031259 | 17.45914909 | 1.047516854 | 1.001373161 |
| Somalia | 2.041868376 | 1.773912693 | 3.637711137 | 3.379511884 | 0.561305804 | 0.524902043 |
| Tanzania | 2.4889613 | 2.833177774 | 4.91191569 | 5.046255007 | 0.50671906 | 0.561441657 |
| Uganda | 2.488681614 | 3.051924818 | 4.592556387 | 4.167070138 | 0.541894623 | 0.732391037 |
| Zambia | 1.914748163 | 2.537774645 | 4.176915149 | 4.554142582 | 0.458412032 | 0.557245321 |
| Southern Sub-Saharan Africa | 4.059567741 | 3.902972726 | 5.73318458 | 5.978925267 | 0.708082512 | 0.652788344 |
| Botswana | 3.697874691 | 4.341564791 | 4.342575359 | 7.197629892 | 0.851539555 | 0.60319367 |
| Lesotho | 3.058698583 | 3.113332997 | 3.992304595 | 3.48707011 | 0.766148602 | 0.892822025 |
| Namibia | 1.987373407 | 3.064370758 | 3.979532474 | 4.735285089 | 0.499398716 | 0.647135431 |
| South Africa | 4.259178585 | 4.22435352 | 6.16948119 | 6.508142556 | 0.690362521 | 0.649087429 |
| Swaziland | 3.191321247 | 3.342844836 | 4.547499299 | 3.377598769 | 0.701774984 | 0.989710461 |
| Zimbabwe | 3.234305806 | 2.25905631 | 5.508349142 | 4.209995615 | 0.587164271 | 0.536593507 |
| Western Sub-Saharan Africa | 3.378109296 | 3.701607139 | 6.087497813 | 6.261124954 | 0.554925751 | 0.591204802 |
| Benin | 3.266037883 | 3.487216916 | 5.887462134 | 6.3411909 | 0.55474461 | 0.549930915 |
| Burkina Faso | 3.408463656 | 3.788631899 | 6.398477163 | 6.731059146 | 0.532699199 | 0.562858209 |
| Cameroon | 3.173509126 | 3.375305164 | 5.618121002 | 5.542943878 | 0.564870199 | 0.608937279 |
| Cape Verde | 2.987523243 | 4.141106668 | 5.255379627 | 6.919081166 | 0.568469541 | 0.598505288 |
| Chad | 2.910696411 | 3.11980266 | 5.579181038 | 5.672724221 | 0.521706751 | 0.549965508 |
| Cote d'Ivoire | 3.181218908 | 3.329754639 | 6.935862904 | 6.635217502 | 0.458662311 | 0.501830518 |
| The Gambia | 3.180605351 | 2.861906059 | 5.650042897 | 5.639943524 | 0.562934726 | 0.507435233 |
| Ghana | 3.517428234 | 3.983822819 | 6.220690165 | 6.654727379 | 0.565440191 | 0.598645533 |
| Guinea | 2.981928051 | 3.139061076 | 5.757450995 | 5.607031008 | 0.517925042 | 0.559843716 |
| Guinea-Bissau | 3.166379829 | 3.360723408 | 5.146811813 | 5.302883589 | 0.615211891 | 0.633753948 |
| Liberia | 3.570131171 | 3.064166877 | 5.661854547 | 5.730605011 | 0.630558617 | 0.53470216 |
| Mali | 2.989213035 | 3.720819896 | 5.658769604 | 6.369193966 | 0.528244344 | 0.584190074 |
| Mauritania | 3.737341804 | 4.387979599 | 6.494316219 | 7.265490822 | 0.575478877 | 0.603948131 |
| Niger | 2.904674107 | 3.141187438 | 5.518101082 | 5.880041968 | 0.526390159 | 0.534211738 |
| Nigeria | 3.586892121 | 3.878244808 | 6.221833356 | 6.487931751 | 0.576500834 | 0.597762886 |
| Sao Tome and Principe | 2.645463937 | 3.005118784 | 5.450068573 | 5.579226025 | 0.48540012 | 0.538626464 |
| Senegal | 3.352202262 | 3.398903631 | 6.128476994 | 6.027062032 | 0.546987819 | 0.563940376 |
| Sierra Leone | 3.013408742 | 3.126294244 | 5.322368421 | 5.618693302 | 0.566178157 | 0.556409485 |
| Togo | 3.212865543 | 3.426039941 | 5.47171211 | 5.309243069 | 0.587177373 | 0.645297248 |
| American Samoa | 7.540790596 | 12.84850676 | 7.587668652 | 12.92742102 | 0.99382181 | 0.993895591 |
| Bermuda | 8.90928243 | 13.44772181 | 11.29563557 | 19.89697977 | 0.78873671 | 0.675867492 |
| Greenland | 6.392662045 | 8.566012601 | 10.71084163 | 14.36211392 | 0.59684031 | 0.596431183 |
| Guam | 17.52320888 | 21.24023889 | 12.52171247 | 20.2584994 | 1.399425911 | 1.048460622 |
| Northern Mariana Islands | 16.99244979 | 27.95358034 | 12.68205224 | 25.53295318 | 1.339881706 | 1.094804042 |
| Puerto Rico | 12.08641066 | 12.4017555 | 14.49157349 | 19.79043032 | 0.83403025 | 0.626654161 |
| Virgin Islands, U.S. | 6.851631201 | 7.81313564 | 8.539355008 | 12.41756777 | 0.802359335 | 0.629200161 |
| South Sudan | 2.341260318 | 2.335092306 | 4.059971244 | 3.881242545 | 0.576669187 | 0.601635244 |
| Sudan | 2.909355835 | 3.671912267 | 5.209012324 | 6.240657996 | 0.558523508 | 0.588385435 |
| High-middle SDI | 6.692112213 | 22.72866726 | 8.883048878 | 26.16566542 | 0.753357581 | 0.868644726 |
| High SDI | 39.55262094 | 52.36583742 | 43.64835756 | 59.18452853 | 0.906165161 | 0.884789298 |
| Low-middle SDI | 2.417718901 | 2.689050389 | 5.178050583 | 6.254157056 | 0.466916818 | 0.429962082 |
| Low SDI | 1.981547998 | 2.168297653 | 4.655282896 | 5.452745853 | 0.425655764 | 0.397652433 |
| Middle SDI | 3.924365127 | 10.76163272 | 6.386876611 | 14.34559081 | 0.614441982 | 0.75017006 |

| Table colors guide |
| --- |
| Global |
| GBD regions |
| Countries |
| SDI quintiles |
