# Supplementary figures and images for: Global, regional, and national quality of care of gallbladder and biliary tract cancer: a systematic analysis for the global burden of disease study 1990–2017
Source: Int J Equity Health. 2021 Dec 18;20:259. doi: 10.1186/s12939-021-01596-y (PMC8684179; doi:10.1186/s12939-021-01596-y)

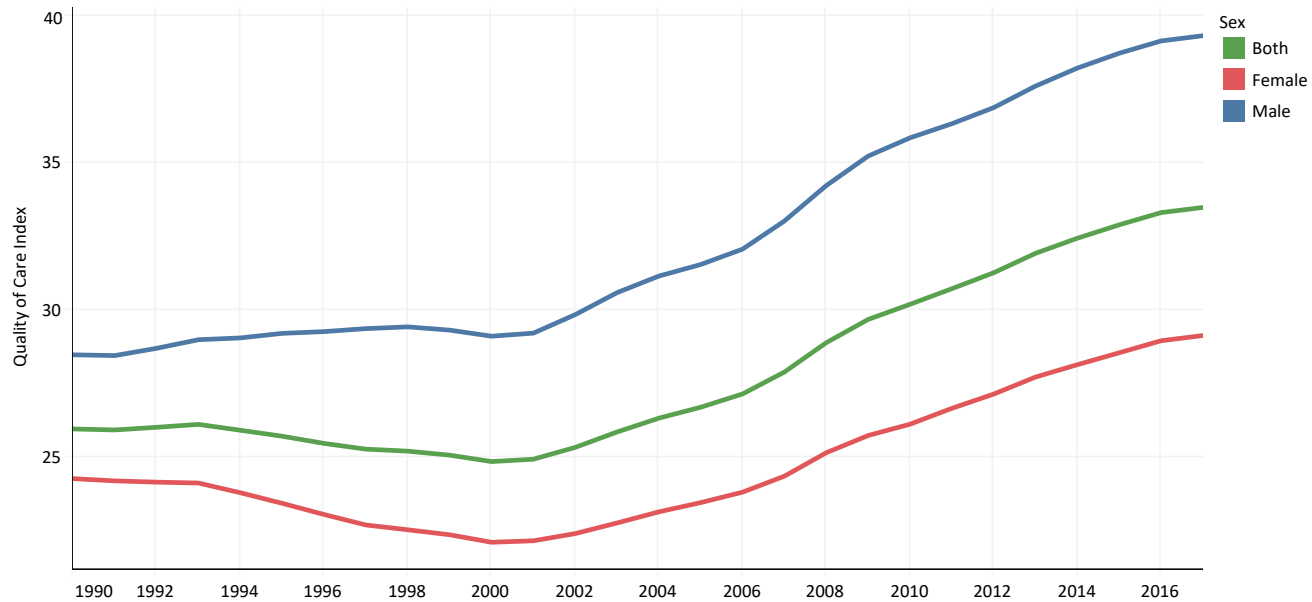

Supplement: Supplementary file 5 — Additional file 5. The global trend of QCI in female, male, and both from 1990 to 2017. [file 12939_2021_1596_MOESM5_ESM.pdf]
