# Supplementary material for: Global, regional, and national quality of care of gallbladder and biliary tract cancer: a systematic analysis for the global burden of disease study 1990–2017
Source: Int J Equity Health. 2021 Dec 18;20:259. doi: 10.1186/s12939-021-01596-y (PMC8684179; doi:10.1186/s12939-021-01596-y)
Supplement: Supplementary file 6 — Additional file 6. Age-specific Quality of Care Index in Global and SDI quintiles’ level in 1990 and 2017. [file 12939_2021_1596_MOESM6_ESM.docx]

| **Supplementary Table 4: Age-specific Quality of Care Index in Global and SDI quintiles’ level in 1990 and 2017** | | | | |
| --- | --- | --- | --- | --- |
| **Location name** | **year** | **Sex name** | **Age name** | **QCI score** |
| Global | 1990 | Both | 15 to 19 | 30.21528 |
| Global | 2017 | Both | 15 to 19 | 39.14201 |
| High-middle SDI | 1990 | Both | 15 to 19 | 25.00133 |
| High-middle SDI | 2017 | Both | 15 to 19 | 54.59847 |
| High SDI | 1990 | Both | 15 to 19 | 72.19704 |
| High SDI | 2017 | Both | 15 to 19 | 89.1588 |
| Low-middle SDI | 1990 | Both | 15 to 19 | 16.03539 |
| Low-middle SDI | 2017 | Both | 15 to 19 | 17.94324 |
| Low SDI | 1990 | Both | 15 to 19 | 11.73563 |
| Low SDI | 2017 | Both | 15 to 19 | 11.14666 |
| Middle SDI | 1990 | Both | 15 to 19 | 15.35389 |
| Middle SDI | 2017 | Both | 15 to 19 | 33.22501 |
| Global | 1990 | Both | 20 to 24 | 29.60722 |
| Global | 2017 | Both | 20 to 24 | 36.27326 |
| High-middle SDI | 1990 | Both | 20 to 24 | 25.81663 |
| High-middle SDI | 2017 | Both | 20 to 24 | 54.79029 |
| High SDI | 1990 | Both | 20 to 24 | 69.61726 |
| High SDI | 2017 | Both | 20 to 24 | 86.72197 |
| Low-middle SDI | 1990 | Both | 20 to 24 | 20.88742 |
| Low-middle SDI | 2017 | Both | 20 to 24 | 23.15482 |
| Low SDI | 1990 | Both | 20 to 24 | 14.57061 |
| Low SDI | 2017 | Both | 20 to 24 | 14.38988 |
| Middle SDI | 1990 | Both | 20 to 24 | 18.09015 |
| Middle SDI | 2017 | Both | 20 to 24 | 34.02495 |
| Global | 1990 | Both | 25 to 29 | 30.31955 |
| Global | 2017 | Both | 25 to 29 | 39.96515 |
| High-middle SDI | 1990 | Both | 25 to 29 | 26.09051 |
| High-middle SDI | 2017 | Both | 25 to 29 | 59.37703 |
| High SDI | 1990 | Both | 25 to 29 | 68.24412 |
| High SDI | 2017 | Both | 25 to 29 | 84.57953 |
| Low-middle SDI | 1990 | Both | 25 to 29 | 20.4726 |
| Low-middle SDI | 2017 | Both | 25 to 29 | 23.68971 |
| Low SDI | 1990 | Both | 25 to 29 | 13.07538 |
| Low SDI | 2017 | Both | 25 to 29 | 14.37792 |
| Middle SDI | 1990 | Both | 25 to 29 | 18.62888 |
| Middle SDI | 2017 | Both | 25 to 29 | 38.04671 |
| Global | 1990 | Both | 30 to 34 | 30.99966 |
| Global | 2017 | Both | 30 to 34 | 40.81837 |
| High-middle SDI | 1990 | Both | 30 to 34 | 21.88476 |
| High-middle SDI | 2017 | Both | 30 to 34 | 56.25903 |
| High SDI | 1990 | Both | 30 to 34 | 67.28619 |
| High SDI | 2017 | Both | 30 to 34 | 83.81969 |
| Low-middle SDI | 1990 | Both | 30 to 34 | 14.12232 |
| Low-middle SDI | 2017 | Both | 30 to 34 | 17.2218 |
| Low SDI | 1990 | Both | 30 to 34 | 10.04086 |
| Low SDI | 2017 | Both | 30 to 34 | 10.81294 |
| Middle SDI | 1990 | Both | 30 to 34 | 14.41244 |
| Middle SDI | 2017 | Both | 30 to 34 | 32.20106 |
| Global | 1990 | Both | 35 to 39 | 33.4738 |
| Global | 2017 | Both | 35 to 39 | 41.92943 |
| High-middle SDI | 1990 | Both | 35 to 39 | 22.29624 |
| High-middle SDI | 2017 | Both | 35 to 39 | 53.79185 |
| High SDI | 1990 | Both | 35 to 39 | 68.44156 |
| High SDI | 2017 | Both | 35 to 39 | 84.31985 |
| Low-middle SDI | 1990 | Both | 35 to 39 | 11.94107 |
| Low-middle SDI | 2017 | Both | 35 to 39 | 15.25021 |
| Low SDI | 1990 | Both | 35 to 39 | 9.544458 |
| Low SDI | 2017 | Both | 35 to 39 | 10.39676 |
| Middle SDI | 1990 | Both | 35 to 39 | 13.4844 |
| Middle SDI | 2017 | Both | 35 to 39 | 31.20548 |
| Global | 1990 | Both | 40 to 44 | 34.99154 |
| Global | 2017 | Both | 40 to 44 | 41.34354 |
| High-middle SDI | 1990 | Both | 40 to 44 | 21.74203 |
| High-middle SDI | 2017 | Both | 40 to 44 | 53.64476 |
| High SDI | 1990 | Both | 40 to 44 | 68.73745 |
| High SDI | 2017 | Both | 40 to 44 | 83.26049 |
| Low-middle SDI | 1990 | Both | 40 to 44 | 9.666262 |
| Low-middle SDI | 2017 | Both | 40 to 44 | 12.32697 |
| Low SDI | 1990 | Both | 40 to 44 | 6.602819 |
| Low SDI | 2017 | Both | 40 to 44 | 8.015416 |
| Middle SDI | 1990 | Both | 40 to 44 | 11.61547 |
| Middle SDI | 2017 | Both | 40 to 44 | 31.83779 |
| Global | 1990 | Both | 45 to 49 | 24.58346 |
| Global | 2017 | Both | 45 to 49 | 30.48647 |
| High-middle SDI | 1990 | Both | 45 to 49 | 11.69999 |
| High-middle SDI | 2017 | Both | 45 to 49 | 30.57061 |
| High SDI | 1990 | Both | 45 to 49 | 52.23333 |
| High SDI | 2017 | Both | 45 to 49 | 77.03596 |
| Low-middle SDI | 1990 | Both | 45 to 49 | 7.216727 |
| Low-middle SDI | 2017 | Both | 45 to 49 | 8.218608 |
| Low SDI | 1990 | Both | 45 to 49 | 6.172201 |
| Low SDI | 2017 | Both | 45 to 49 | 6.839954 |
| Middle SDI | 1990 | Both | 45 to 49 | 7.951247 |
| Middle SDI | 2017 | Both | 45 to 49 | 15.63592 |
| Global | 1990 | Both | 50 to 54 | 22.46593 |
| Global | 2017 | Both | 50 to 54 | 26.09228 |
| High-middle SDI | 1990 | Both | 50 to 54 | 11.82277 |
| High-middle SDI | 2017 | Both | 50 to 54 | 21.52269 |
| High SDI | 1990 | Both | 50 to 54 | 44.94933 |
| High SDI | 2017 | Both | 50 to 54 | 71.30638 |
| Low-middle SDI | 1990 | Both | 50 to 54 | 9.233917 |
| Low-middle SDI | 2017 | Both | 50 to 54 | 9.427267 |
| Low SDI | 1990 | Both | 50 to 54 | 8.575568 |
| Low SDI | 2017 | Both | 50 to 54 | 8.795303 |
| Middle SDI | 1990 | Both | 50 to 54 | 9.90736 |
| Middle SDI | 2017 | Both | 50 to 54 | 13.71436 |
| Global | 1990 | Both | 55 to 59 | 26.18322 |
| Global | 2017 | Both | 55 to 59 | 33.47703 |
| High-middle SDI | 1990 | Both | 55 to 59 | 11.66478 |
| High-middle SDI | 2017 | Both | 55 to 59 | 28.2781 |
| High SDI | 1990 | Both | 55 to 59 | 47.06489 |
| High SDI | 2017 | Both | 55 to 59 | 68.50656 |
| Low-middle SDI | 1990 | Both | 55 to 59 | 6.626397 |
| Low-middle SDI | 2017 | Both | 55 to 59 | 7.101821 |
| Low SDI | 1990 | Both | 55 to 59 | 5.800991 |
| Low SDI | 2017 | Both | 55 to 59 | 6.02396 |
| Middle SDI | 1990 | Both | 55 to 59 | 8.261727 |
| Middle SDI | 2017 | Both | 55 to 59 | 16.76215 |
| Global | 1990 | Both | 60 to 64 | 26.35644 |
| Global | 2017 | Both | 60 to 64 | 33.01512 |
| High-middle SDI | 1990 | Both | 60 to 64 | 9.834263 |
| High-middle SDI | 2017 | Both | 60 to 64 | 24.80518 |
| High SDI | 1990 | Both | 60 to 64 | 45.38377 |
| High SDI | 2017 | Both | 60 to 64 | 63.01158 |
| Low-middle SDI | 1990 | Both | 60 to 64 | 4.016327 |
| Low-middle SDI | 2017 | Both | 60 to 64 | 4.831645 |
| Low SDI | 1990 | Both | 60 to 64 | 3.577085 |
| Low SDI | 2017 | Both | 60 to 64 | 3.891954 |
| Middle SDI | 1990 | Both | 60 to 64 | 6.150932 |
| Middle SDI | 2017 | Both | 60 to 64 | 13.58339 |
| Global | 1990 | Both | 65 to 69 | 29.33755 |
| Global | 2017 | Both | 65 to 69 | 35.01993 |
| High-middle SDI | 1990 | Both | 65 to 69 | 8.282754 |
| High-middle SDI | 2017 | Both | 65 to 69 | 27.67257 |
| High SDI | 1990 | Both | 65 to 69 | 46.20405 |
| High SDI | 2017 | Both | 65 to 69 | 60.40228 |
| Low-middle SDI | 1990 | Both | 65 to 69 | 2.931077 |
| Low-middle SDI | 2017 | Both | 65 to 69 | 3.328028 |
| Low SDI | 1990 | Both | 65 to 69 | 2.764753 |
| Low SDI | 2017 | Both | 65 to 69 | 3.198417 |
| Middle SDI | 1990 | Both | 65 to 69 | 4.3976 |
| Middle SDI | 2017 | Both | 65 to 69 | 12.77561 |
| Global | 1990 | Both | 70 to 74 | 26.75583 |
| Global | 2017 | Both | 70 to 74 | 32.36162 |
| High-middle SDI | 1990 | Both | 70 to 74 | 5.516882 |
| High-middle SDI | 2017 | Both | 70 to 74 | 24.39399 |
| High SDI | 1990 | Both | 70 to 74 | 42.08611 |
| High SDI | 2017 | Both | 70 to 74 | 54.27613 |
| Low-middle SDI | 1990 | Both | 70 to 74 | 2.976071 |
| Low-middle SDI | 2017 | Both | 70 to 74 | 3.164621 |
| Low SDI | 1990 | Both | 70 to 74 | 3.004432 |
| Low SDI | 2017 | Both | 70 to 74 | 3.359195 |
| Middle SDI | 1990 | Both | 70 to 74 | 3.918344 |
| Middle SDI | 2017 | Both | 70 to 74 | 8.457283 |
| Global | 1990 | Both | 75 to 79 | 25.8795 |
| Global | 2017 | Both | 75 to 79 | 33.38101 |
| High-middle SDI | 1990 | Both | 75 to 79 | 4.967688 |
| High-middle SDI | 2017 | Both | 75 to 79 | 21.91191 |
| High SDI | 1990 | Both | 75 to 79 | 36.72861 |
| High SDI | 2017 | Both | 75 to 79 | 50.54862 |
| Low-middle SDI | 1990 | Both | 75 to 79 | 3.5126 |
| Low-middle SDI | 2017 | Both | 75 to 79 | 4.129444 |
| Low SDI | 1990 | Both | 75 to 79 | 3.509191 |
| Low SDI | 2017 | Both | 75 to 79 | 4.166433 |
| Middle SDI | 1990 | Both | 75 to 79 | 4.038563 |
| Middle SDI | 2017 | Both | 75 to 79 | 10.09152 |
| Global | 1990 | Both | 80 to 84 | 29.49122 |
| Global | 2017 | Both | 80 to 84 | 39.37788 |
| High-middle SDI | 1990 | Both | 80 to 84 | 6.6667 |
| High-middle SDI | 2017 | Both | 80 to 84 | 21.01865 |
| High SDI | 1990 | Both | 80 to 84 | 39.14392 |
| High SDI | 2017 | Both | 80 to 84 | 54.57692 |
| Low-middle SDI | 1990 | Both | 80 to 84 | 3.769508 |
| Low-middle SDI | 2017 | Both | 80 to 84 | 4.498475 |
| Low SDI | 1990 | Both | 80 to 84 | 3.756065 |
| Low SDI | 2017 | Both | 80 to 84 | 3.68319 |
| Middle SDI | 1990 | Both | 80 to 84 | 4.839992 |
| Middle SDI | 2017 | Both | 80 to 84 | 14.10224 |
| Global | 1990 | Both | 85 to 89 | 31.70533 |
| Global | 2017 | Both | 85 to 89 | 44.03173 |
| High-middle SDI | 1990 | Both | 85 to 89 | 7.099999 |
| High-middle SDI | 2017 | Both | 85 to 89 | 20.91099 |
| High SDI | 1990 | Both | 85 to 89 | 40.53199 |
| High SDI | 2017 | Both | 85 to 89 | 55.4204 |
| Low-middle SDI | 1990 | Both | 85 to 89 | 4.287989 |
| Low-middle SDI | 2017 | Both | 85 to 89 | 4.105411 |
| Low SDI | 1990 | Both | 85 to 89 | 4.309785 |
| Low SDI | 2017 | Both | 85 to 89 | 3.773509 |
| Middle SDI | 1990 | Both | 85 to 89 | 5.155698 |
| Middle SDI | 2017 | Both | 85 to 89 | 14.11339 |
| Global | 1990 | Both | 90 to 94 | 33.96764 |
| Global | 2017 | Both | 90 to 94 | 49.02534 |
| High-middle SDI | 1990 | Both | 90 to 94 | 7.068616 |
| High-middle SDI | 2017 | Both | 90 to 94 | 20.45247 |
| High SDI | 1990 | Both | 90 to 94 | 41.40239 |
| High SDI | 2017 | Both | 90 to 94 | 56.47953 |
| Low-middle SDI | 1990 | Both | 90 to 94 | 5.231137 |
| Low-middle SDI | 2017 | Both | 90 to 94 | 5.689658 |
| Low SDI | 1990 | Both | 90 to 94 | 5.384726 |
| Low SDI | 2017 | Both | 90 to 94 | 5.138227 |
| Middle SDI | 1990 | Both | 90 to 94 | 5.546976 |
| Middle SDI | 2017 | Both | 90 to 94 | 14.98956 |
| Global | 1990 | Both | 95 plus | 36.25923 |
| Global | 2017 | Both | 95 plus | 54.36391 |
| High-middle SDI | 1990 | Both | 95 plus | 10.20235 |
| High-middle SDI | 2017 | Both | 95 plus | 21.64297 |
| High SDI | 1990 | Both | 95 plus | 42.79959 |
| High SDI | 2017 | Both | 95 plus | 59.01451 |
| Low-middle SDI | 1990 | Both | 95 plus | 8.393756 |
| Low-middle SDI | 2017 | Both | 95 plus | 11.11741 |
| Low SDI | 1990 | Both | 95 plus | 8.120916 |
| Low SDI | 2017 | Both | 95 plus | 8.630449 |
| Middle SDI | 1990 | Both | 95 plus | 8.90899 |
| Middle SDI | 2017 | Both | 95 plus | 19.07809 |
